# Aza-Diels–Alder reaction between *N*-aryl-1-oxo-1*H*-isoindolium ions and *tert*-enamides: Steric effects on reaction outcome

**DOI:** 10.3762/bjoc.10.81

**Published:** 2014-04-14

**Authors:** Amitabh Jha, Ting-Yi Chou, Zainab ALJaroudi, Bobby D Ellis, T Stanley Cameron

**Affiliations:** 1 Department of Chemistry, Acadia University, Wolfville, NS, Canada; 2 Department of Chemistry, Dalhousie University, Halifax, NS, Canada

**Keywords:** alkene addition, cyclization, Diels–Alder, inverse electron demand, *N*-acyliminium ion, *tert*-enamide

## Abstract

The synthesis of 5-substituted 6,6a-dihydroisoindolo[2,1-*a*]quinolin-11(5*H*)-ones via [4 + 2] imino-Diels–Alder cyclization from *N*-aryl-3-hydroxyisoindolinones and *N-*vinyl lactams under Lewis acid-catalysed anhydrous conditions is reported. Reactions of *N*-(2-substituted-aryl)-3-hydroxyisoindolinones with *N*-vinylpyrrolidone under identical conditions resulted in the formation of 2-(2-substitued-aryl)-3-(2-(2-oxopyrrolidin-1-yl)vinyl)isoindolin-1-one analogues indicating steric hinderance as the cause of deviation. The probable mechanism of the reaction based on the results from X-ray crystallography and molecular modelling is discussed.

## Introduction

**Figure 1:**
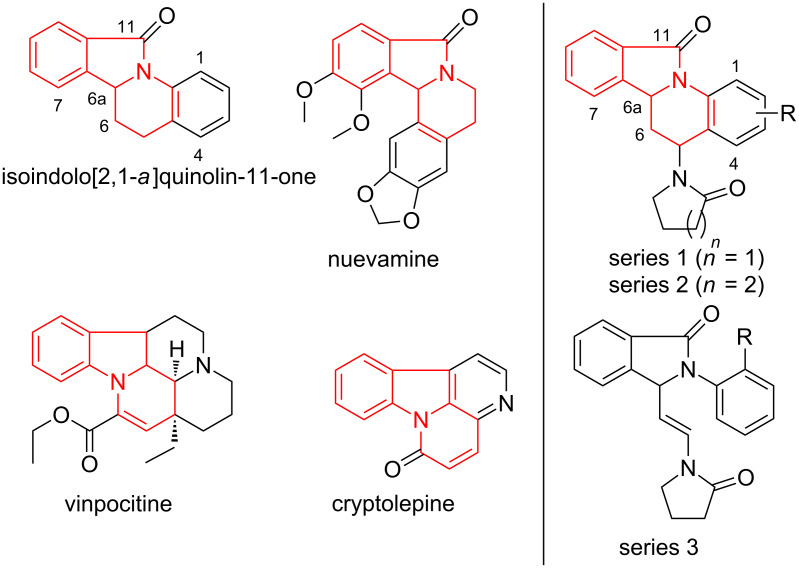
Pyridoisoindole frameworks (highlighted) in bioactive molecules and compounds under present investigation (series **1–3**).

Fused indoline, isoindoline, quinoline and isoquinoline substructures are found in many natural products and bioactive synthetic compounds (Figure 1). For example, nuevamine is a naturally-occurring isoindolo[1,2-*a*]isoquinolinone which has been isolated from *Berberis darwinii* [1–3]. Cryptolepine is an indolo[3,2-*b*]quinolone alkaloid found in west African shrub *Cryptolepis sanguinolenta,* a plant used in traditional medicine for the treatment of malaria [3]. This alkaloid has shown potent antiplasmodial [4] and anticancer [5] activities. The bioactive β-carboline alkaloids canthinone [6] and vinpocitine [7] also bear these substructures. Vinpocetine is a dehydrated derivative of the natural alkaloid of vincamine [8]. It is reported to have cerebral blood-flow enhancing [7] and neuroprotective effects [9], and is used as a drug in Eastern Europe for the treatment of cerebrovascular disorders and age-related memory impairment [10]. We are interested in the isoindolo[2,1-*a*]quinoline skeleton (Figure 1) due to its structural similarities with these alkaloids.

**Scheme 1:**
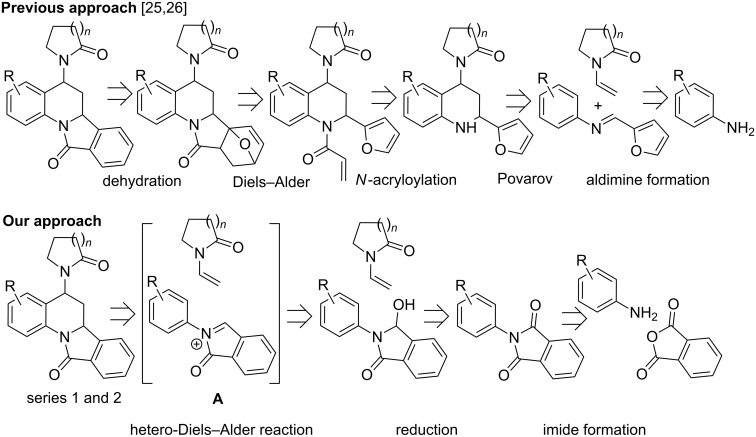
Comparison of the retro-synthetic approach for the synthesis of isoindoloquinoline skeleton reported in the literature and our strategy.

The most common method of synthesizing isoindolo[2,1-*a*]quinolone derivatives involves *N*-acyliminium ions or appropriate electron-deficient Schiff bases and subsequent [4 + 2] inverse-demand hetero-Diels–Alder cycloadditions with alkenes [11–17]. Vinylic systems from isoeugenol [11], cyclopentadiene [12], enones [13], vinyl ethers [14] and enolic 1,3-diketo compounds [15], have been reported to react with *N*-acyliminium ions obtained either from 2-formylbenzoic acid and anilines [11–12] or from *N*-acyliminium ions prepared from *N*-arylphthalimides [13–15]. Nucleophilic substitution of *N*-aryl-3-hydroxyisoindolinones from *N*-aryl-3-hydroxyisoindolinones with diethyl malonate and subsequent hydrolysis, decarboxylation and Friedel–Crafts acylation sequence also result in the formation of isoindolo[2,1-*a*]quinolones [16–17]. *N*-aryl-3-hydroxyisoindolinones with an aptly positioned alkene moiety at the *ortho* position of the *N*-aryl group undergo intramolecular electrophilic addition to the alkene yielding isoindolo[2,1-*a*]quinolines under acidic conditions [18]. Isoindolo[2,1-*a*]quinolone cores have also been prepared via an aldol-type intramolecular cyclization reaction of *N-*(2-acetylaryl)phthalimide under anhydrous and strongly basic conditions [19]. Kang et al*.* achieved a highly enantioselective synthesis of an isoindolo[2,1-*a*]quinoline derivative by affecting an intramolecular ring closure on (*E*)-3-(2-(isoindolin-2-yl)phenyl)acrylaldehyde using camphorsulfonic acid and a chiral pyrrolidine organocatalyst [20]. The reaction of 2-chloroquinoline with 2-formylphenylboronic acid yielded tetracyclic isoindoloquinoline using Suzuki cross-coupling conditions where the C–C bond formation is followed by nucleophilic attack of the pyridine lone pair on the neighbouring aldehyde and proton migration [21]. Several procedures involved the use of the Diels–Alder reaction on a furan ring to synthesize isoindoloquinoline derivatives [22–25]. Reaction of methallylmagnesium chloride with furyl aldimines produced furan-substituted *N*-aryl homoallylamines which reacted with maleic anhydride to undergo amide formation and Diels–Alder cascade. Concomitant electrophilic aromatic substitution and dehydration resulted in isoquinoloquinoline derivatives [22]. Similarly, isoindoloquinolines were also synthesized via classical Povarov chemistry between furyl aldimines and *tert*-enamides followed by a *N*-acryloylation, Diels–Alder reaction [23–24] and dehydration sequence [25–26] (Scheme 1).

As mentioned earlier, *N*-aryl-1-oxo-1*H*-isoindolium ions (**A**, Scheme 1) undergo [4 + 2] imino-Diels–Alder cyclization with electron-rich alkenes [11–17], the steric effects on the outcome of these reactions has not been studied. In continuation of our efforts on the development of novel synthetic methodologies [27–36], we report herein a systematic study of steric and electronic effects of aryl substituents leading to substituted 6,6a-dihydroisoindolo[2,1-*a*]quinolin-11(5*H*)-ones (series 1 and 2) from 1-oxo-2-aryl-1*H*-isoindolium ions (**A**) and appropriate *tert*-enamides (Scheme 1). Although this is the first report of the use of *tert*-enamides as a dienophile for the hetero-Diels–Alder reaction with *N*-acyliminium cations, formation of isoindoloquinolines (series 1 and 2) was rather expected based on literature reports [11–17]. The unpredicted formation of *E*-2-(2-substituted-aryl)-3-(2-(2-oxopyrrolidin-1-yl)vinyl)isoindolin-1-ones (series 3, Figure 1) from *N*-(2-substituted-aryl)-1-oxo-1*H*-isoindolium ions (**A**) and *tert*-enamides under identical conditions forms the major highlight of this work.

## Results and Discussion

Our literature search revealed that although *tert*-enamides have been used as dienophiles for the Povarov reaction to build 1,2,3,4-tetrahydroquinoline blocks [25–26,37], there were no reports of their usage in the inverse electron demand hetero-Diels–Alder reaction with an *N*-acyliminium cation. We envisaged that the isoindoloquinoline skeleton synthesized by Kouznetsov et al. [25] and Zaytsev et al. [26] in a 5-step sequence (Scheme 1) could be more conveniently synthesized (3-step sequence, Scheme 1) by employing *N*-aryl-3-hydroxy-isoindolinones as the *N*-acyliminium ion source and performing [4 + 2] imino-Diels–Alder reactions with *tert*-enamides. Our literature search also infuses confidence in the proposed scheme because the synthesis of *N*-aryl-3-hydroxyisoindolinones is well studied and standardized [13–15] and the reactions analogous to the final step of imino [4 + 2] Diels–Alder reactions between *N*-acyliminium ions and electron-rich dienophiles are reported [13–15].

We synthesized eight *N*-aryl-3-hydroxyisoindolinones from substituted anilines and phthalic anhydride following the reported procedure [13]. Previously reported reaction conditions for imino [4 + 2] Diels–Alder reactions [13–14] were employed here for the electrocyclization of *N*-acyliminium ions from *N*-aryl-3-hydroxyisoindolinones and *tert*-enamide analogues namely *N*-vinylpyrrolidone and *N*-vinylcaprolactam as the electron-rich dienophiles. Sixteen isoindoloquinoline derivatives were successfully synthesized and isolated following this protocol. The results are shown in Table 1.

**Table 1:** Reaction data for the synthesis of compounds **1a–h** and **2a–h**.

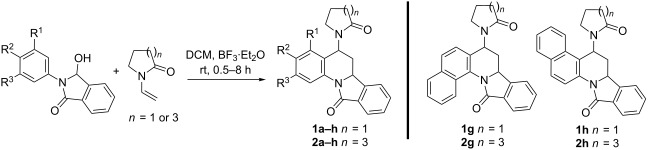								

Entry	R^1^	R^2^	R^3^	*n*	Product	Time^a^	% Yield^b^	Mp (°C)

1	H	H	H	1	**1a**	1 h	56	225–227
2	H	Me	H	1	**1b**	3 h	62	202–204
3	H	MeO	H	1	**1c**	3 h	82	227–230
4	H	Cl	H	1	**1d**	8 h	41	229–230
5	Me	H	Me	1	**1e**	8 h	59	232–235
6	H	F	Cl	1	**1f**	8 h	33	>260
7	1-naphthylamine			1	**1g**	8 h	47	197–199
8	2-naphthylamine			1	**1h**	8 h	50	242–244
9	H	H	H	3	**2a**	1 h	47	228–230
10	H	Me	H	3	**2b**	0.5 h	80	218–221
11	H	MeO	H	3	**2c**	1 h	48	216–217
12	H	Cl	H	3	**2d**	3 h	34	248–250
13	Me	H	Me	3	**2e**	8 h	33	204–207
14	H	F	Cl	3	**2f**	4 h	42	>260
15	1-naphthylamine			3	**2g**	8 h	34	260–262
16	2-naphthylamine			3	**2h**	8 h	32	238–240

^a^The entries with the reaction time less than 8 h formed pure precipitated products. ^b^Isolated yield based on *N*-aryl-1*H*-pyrrole-2,5-dione.

Among the series of reactions, reactions corresponding to entries 1–3, 9–12 and 14 formed the products as precipitates while others did not. The yields varied from 32–82% and appeared to depend on the functional groups on the aniline ring. Upon close investigation in a couple of cases (Table 1, entries 4 and 6), the main reason for the low yield was found to be the competing dimerization of the *tert*-enamides, which is known to happen under acidic conditions [38–39]. Electron donating groups at the *para* position of the aniline generally led to the formation of products in higher yields in comparison to those bearing electron withdrawing groups (e.g., R^2^ = –Cl, –F). Similar observations were made previously [13]. Furthermore, in the case of anilines with *meta* substitution, the reaction took longer and the yields of the final products were founds to be lower (Table 1, entries 5, 6, 13, 14). This might have been caused due to steric hindrance. It was also noted that the imino-Diels–Alder cyclization of *N-*acyliminium cations with *N*-vinylpyrrolidone generally afforded higher yields than with *N*-vinylcaprolactam. The only exception was entry 10 where the reaction appeared to be very fast (0.5 h) and the product precipitated in the reaction mixture. All compounds were completely characterized based on their spectral data.

**Scheme 2:**
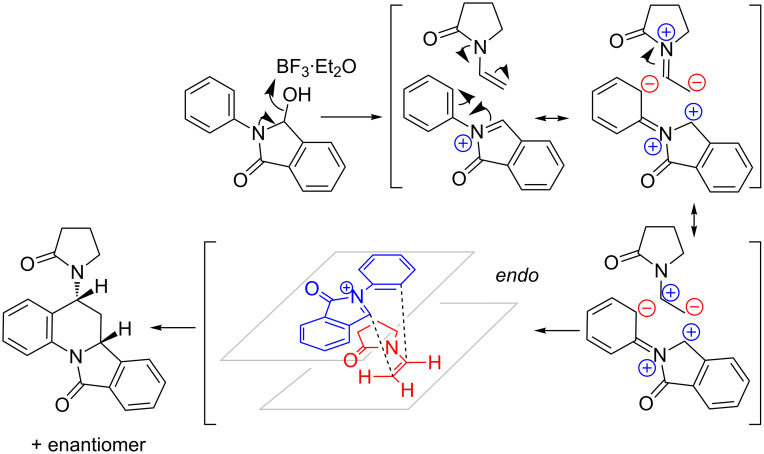
Mechanistic explanation for regio- and diastereoselectivity leading to (±)-6,6a-dihydroisoindolo[2,1-*a*]quinolin-11(5*H*)-one.

Owing to the unsymmetrical structures of the diene as well as the dienophile, at least two regioisomers can be visualized for these compounds. Based on our characterization results, only one regioisomer, where the substitution was at position 5 of the isoindoloquinoline ring, was exclusively formed in these reactions. Inverse-electron demand hetero-Diels–Alder reactions are known to exhibit this type of regioselectivity, and it can be explained based on charge control [40] (Scheme 2). The polarization of the iminium cation places the positive charge on the benzylic C of the isoindole ring and subsequently the *N*-aryl ring develops a negative charge at the *ortho* position. The polarized form of the *tert*-enamide dienophile, on the other hand, will have a positive and negative charge on carbons α and β to N, respectively. Such polarization can only lead to the regioisomer with the substitution at position 5 of the isoindoloquinoline ring.

Even with this regioselectivity, Table 1, entries 6 and 14 with 3-chloro-4-fluoroaniline and entries 8 and 15 with 2-naphthylamine as the starting materials were capable of forming additional regioisomers, but the reaction appeared to be regioselective again in these cases, each producing only one regioisomer. These observations can be explained based on steric and electronic factors. In the case derived from 3-chloro-4-fluoroaniline, the C–C bond formation occurred at position 6 of the aromatic ring as it was sterically favoured. In case of the 2-naphthylamine derivative, position 1 of the naphthalene ring is known to be more reactive as the resonance forms of the putative intermediate retain benzenoid character of one aromatic ring [41].

**Figure 2:**
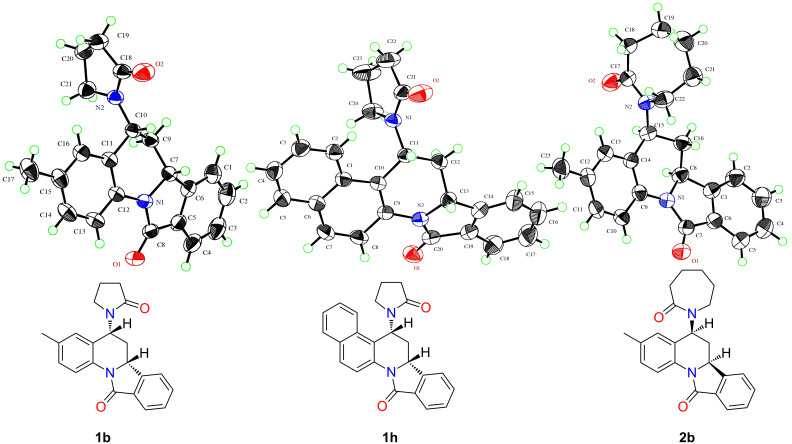
ORTEP diagrams and 2D structures for the isoindolo[2,1-*a*]quinolone derivatives **1b**, **1h** and **2b**.

Another observation that further supports the inverse-electron demand aza-Diels–Alder mechanism is the diastereoselectivity shown in these reactions. All compounds in series 1 and 2 have two stereogenic centres and are therefore capable of forming four diastereomers. Only *endo* products, where the hydrogen atoms on the chiral centres are in a *cis* orientation with respect to each other, as a racemic mixture, are exclusively formed in these reactions as confirmed by X-ray crystallography on single crystals of the three representative compounds **1b**, **1h** and **2b** (CCDC 952236, 951754 and 951755 respectively). Their ORTEP diagrams and corresponding 2D structures of these compounds are shown in Figure 2. ^1^H and ^13^C NMR spectra of all isolated products also indicate formation of a single diastereomer as a racemic mixture.

Under kinetic control, inverse-electron demand aza-Diels–Alder as well as normal Diels–Alder reactions are known to favour *endo* products since the *endo* approach maximizes secondary orbital overlap [42]. The “boat-type” transition state favours an *endo* orientation (“inside of the boat”) of the electron-donating substituents on the dienophile (Scheme 2).

To further understand the steric effects, we elected to study this transformation on *ortho*-substituted aniline-derived *N*-acyliminium cations. Nine *N*-(2-substituted-aryl)-3-hydroxyisoindolinones (Table 2) were prepared using the procedure identical to those reported in Table 1. In the subsequent BF_3_·Et_2_O-catalysed reaction with *N*-vinylpyrrolidin-2-one under conditions identical to the one employed earlier for series 1 and 2 cyclized products, irrespective of the size and electronic property of the substituent, no inverse-electron demand hetero-Diels–Alder reaction was observed. Instead, a C–C bond was formed between the terminal carbon on the double bond of *N*-vinylpyrrolidin-2-one and C3 of the *N*-acyliminium cation.

**Table 2:** Reaction data for the synthesis of compounds **3a–i**.

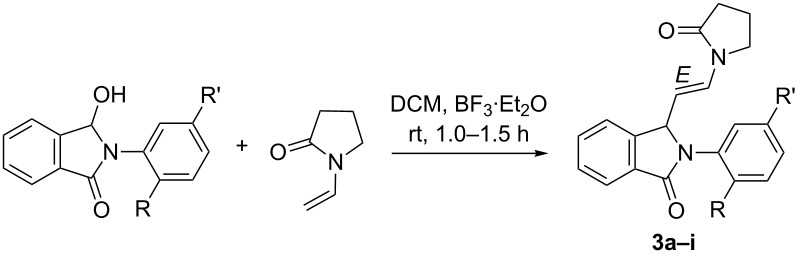						

Entry	R	R'	Product	Time	%Yield	Mp (°C)

1	F	H	**3a**	1 h	39	260–262
2	Cl	H	**3b**	1 h	51	97–101
3	I	H	**3c**	1 h	57	118–120
4	Br	Br	**3d**	1.5 h	45	248–250
5	NO_2_	H	**3e**	1 h	66	112–115
6	Et	H	**3f**	1 h	63	155–157
7	*t*-Bu	H	**3g**	1 h	56	246–248
8	OMe	H	**3h**	1 h	53	86–89
9	OCF_3_	H	**3i**	1 h	58	>260

**Figure 3:**
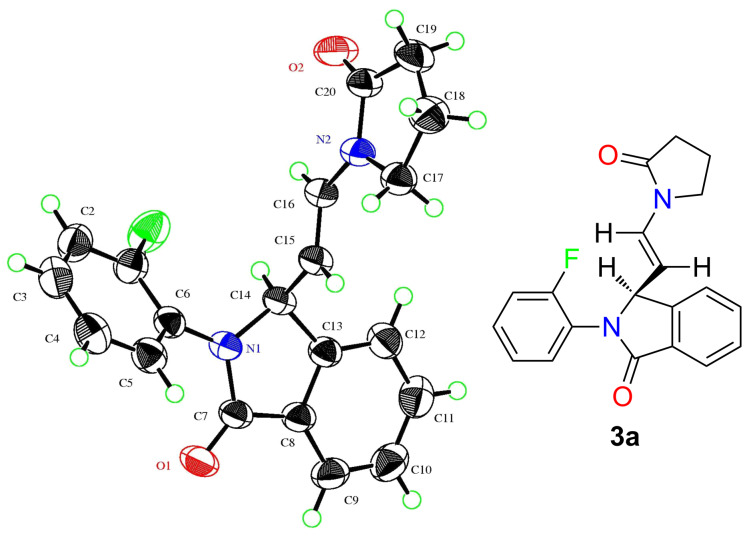
ORTEP diagram and 2D structure of *E*-2-(2-fluorophenyl)-3-(2-(2-oxopyrrolidin-1-yl)vinyl)isoindolin-1-one (**3a**).

All compounds of series 3 were characterized by spectroscopic means. To conclusively establish the structure and geometry around the double bond, single crystal X-ray crystallography was performed on a representative compound, **3a**. The NMR data as well as the X-ray structure indicate an *E-*configuration around the vinylic double bond. The ORTEP diagram of the molecular structure of **3a** is presented in Figure 3 (CCDC 951756).

**Scheme 3:**
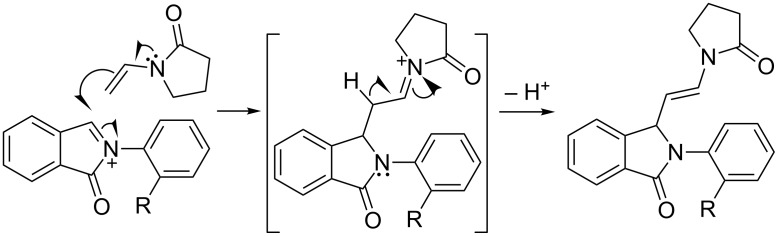
Most plausible mechamism for the formation of *E*-2-(2-substituted-phenyl)-3-(2-(2-oxopyrrolidin-1-yl)vinyl)isoindolin-1-ones (**3a–i**).

The mechanism of the formation of compounds **3a–i** is fairly straightforward (Scheme 3). Nucleophilic attack from the *tert*-enamide on the *N*-acyliminium cation leads to the formation of the first C–C bond. The resulting *N*-acyliminium cation undergoes β-proton elimination to yield the final products. The lack of cyclization in these cases is interesting from several standpoints. First, it becomes clear that *N*-acyliminium cations generated from *N*-aryl-3-hydroxyisoindolinones are capable of non-concerted reactions as previously reported [14–15]. Secondly, the required *cisoid* geometry of the *N*-acyliminium cation “diene” was presumably not maintained due to *ortho* substitution on the *N*-aryl. This potentially led to misalignment of the orbitals preventing the concerted cycloaddition. Thirdly, it is intriguing to ponder the reasons for the inability of the cationic intermediate to further cyclize with position 6 of the *N*-aryl ring in a non-concerted way.

**Figure 4:**
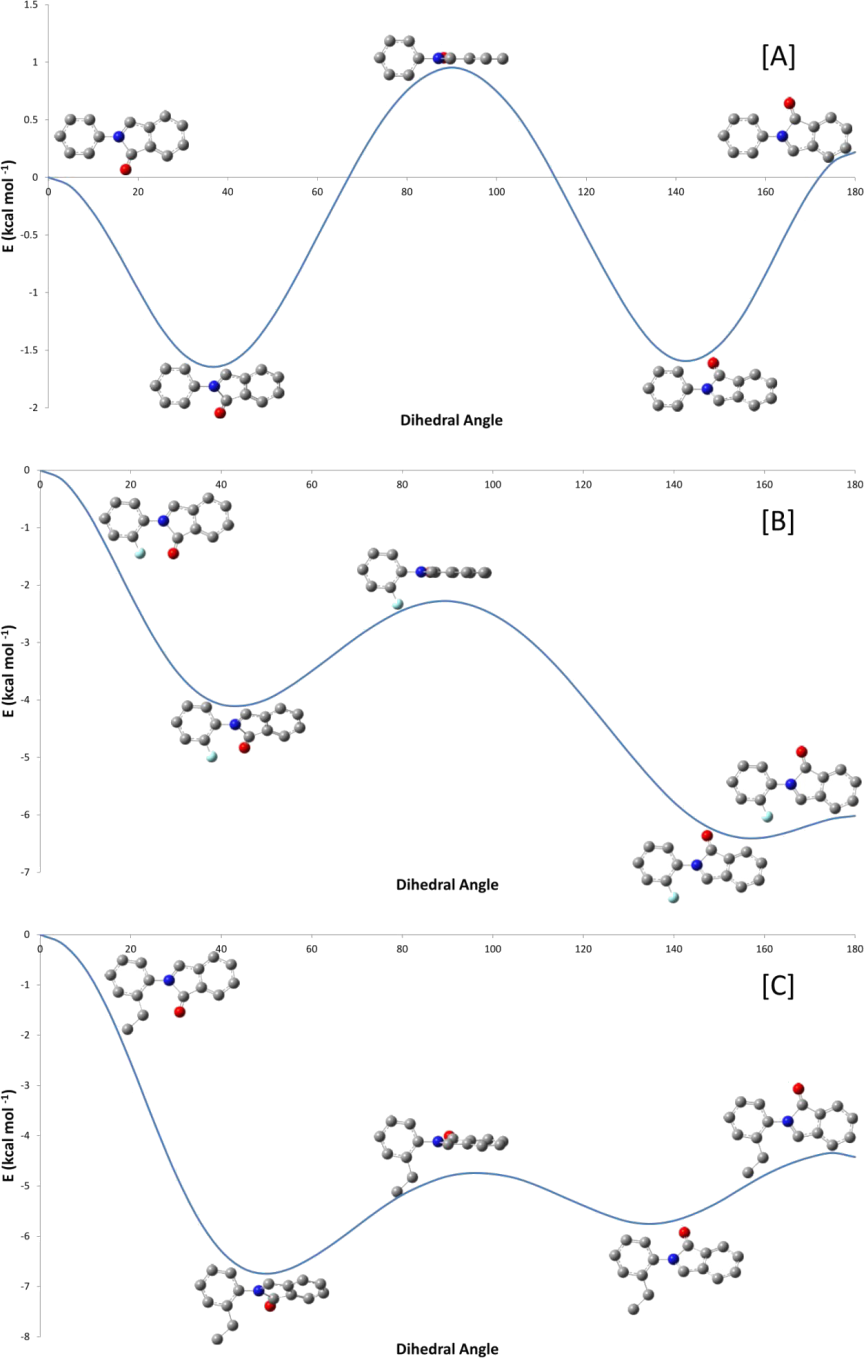
Rotational barrier calculation across *N*-aryl bond for the *N*-acyliminium ion intermediates of **1a** [A], **3a** [B] and **3f** [C].

The electronic influence of the substituents at position 2 of the *N*-aryl group does not appear to play a role as both strongly electron donating (–OCH_3_, entry 8, Table 2) as well as strongly electron withdrawing (–NO_2_, entry 5, Table 2) groups undergo the same fate of condensation without further electrophilic aromatic substitution. Lu et al. [43] have recently reported the synthesis of 3-(1-alkenyl)isoindolin-1-ones from *N*-acyliminium cations; however, their intermediates did not have the opportunity for intramolecular cyclization. The nitrogen atom in their case was either unsubstituted or substituted with methyl or benzyl groups. Steric impedance from the *ortho* substituent may be cited as a reason for the lack of cyclization with position 6 of the *N*-aryl ring. At this point, we decided to investigate the conformations of *N*-(2-substituted-phenyl)-1-oxo-1*H*-isoindolium ions by molecular modelling. Like biphenyl, the *N*-phenyl-1-oxo-1*H*-isoindolium ions were expected to show extended conjugation and not to acquire a mutually perpendicular conformation with respect to the *N*-phenyl and the oxoisoindolium moieties. Like biphenyl [44], the equilibrium torsional angle was found to be approximately 33° with a very small torsional barrier of approximately 1.5 kcal/mol [45]. Thus, a *cisoid* conformation is easily possible for *N*-phenyl-1-oxo-1*H*-isoindolium ions. Two *cisoid* conformations can be drawn for *N*-(2-substituted-phenyl)-1-oxo-1*H*-isoindolium ions – the *cisoid* conformation where the substituent at position 2 is at the vicinity of the carbonyl group is the one that can produce the electrocyclic (or non-concerted) reaction product(s). These *cisoid* conformations for *N*-(2-fluorophenyl) and *N*-(2-ethylphenyl) substituted 1-oxo-1*H*-isoindolium ions corresponding to a dihedral angle of 0° on the dihedral driver chart are shown in Figure 4. The torsional barrier in the case of 2-fluoro and 2-ethyl substituted ions were found to be approximately 6.5–7 kcal/mol. Thus, it becomes abundantly clear as to why *N*-vinylpyrrolidone does not cyclize (in a concerted fashion or otherwise) with *N*-(2-substituted phenyl)-1-oxo-1*H*-isoindolium ions.

## Conclusion

6,6a-Dihydroisoindolo[2,1-*a*]quinolin-11(5*H*)-one derivatives were synthesized from *N*-aryl-3-hydroxyisoindolinones and *N-*vinyl lactams under Lewis acid-catalysed anhydrous conditions in fair to good yields. As expected, the reaction appeared to proceed via the formation of an *N*-acyliminium ion which underwent an inverse-electron demand imino-Diels–Alder reaction. While reactions of this type are known to occur, this is the first report of the use of *N*-vinyl lactams as a dienophile for the inverse-electron demand imino-Diels–Alder reaction with *N-*acyliminium cations as dienes. The most important aspect of this investigation is the dissimilar outcome of the reactions of *N*-(2-substituted-aryl)-3-hydroxyisoindolinones with *N*-vinylpyrrolidone under identical conditions which resulted in the electrophilic addition of an *N*-acyliminium ion on the vinyl group followed by β-elimination leading to 2-(2-substitued-aryl)-3-(2-(2-oxopyrrolidin-1-yl)vinyl)isoindolin-1-one analogues. This indicated that the *ortho* substituent on the *N*-acyliminium ion became detrimental for cyclization to occur.

## Experimental

Seventeen *N*-arylphthalimides were synthesized by reacting phthalic anhydride with appropriate anilines following a literature procedure [46]. Borohydride reduction [13] of these *N*-arylphthalimides afforded seventeen *N-*aryl-3-hydroxyisoindolinones.

**General procedure for the synthesis of 5-(2-oxopyrrolidin-1-yl)-6,6a-dihydroisoindolo[2,1-*a*]quinolin-11(5*H*)-ones (1a–h); 5-(2-oxoazepan-1-yl)-6,6a-dihydroisoindolo[2,1-*a*]quinolin-11(5*H*)-ones (2a–h) and *(E)*-2-(2-substitued-phenyl)-3-(2-(2-oxopyrrolidin-1-yl)vinyl)isoindolin-1-ones (3a–i)**

Appropriate *N-*aryl-3-hydroxyisoindolinones (500 mg) from the previous step were dissolved in dichloromethane (10 mL) in a 50 mL round bottom flask which was then sealed with a rubber septum. BF_3_:Et_2_O (1.5 equiv) was added to the mixture slowly through the septum by a syringe. This resulted in the formation of a transparent solution. Appropriate *tert*-enamide (1.5 equiv) dissolved in dichloromethane (5 mL) was slowly added to the flask through a syringe over a period of 5 minutes. The reaction was then allowed to stir at room temperature until precipitates formed. The reaction progress was monitored by TLC. Upon completion of the reaction, the residue was suction filtered and the solid obtained was extracted with ethyl acetate (60 mL) and washed with water (30 mL × 3). The organic layer was dried over anhydrous Na_2_SO_4_ and evaporated in vacuum to dryness to obtain pure products. In the cases where precipitates did not form, the solvents were evaporated to dryness under reduced pressure. The residue was extracted by ethyl acetate (60 mL) and washed with water (30 mL × 3). The organic layer was dried over anhydrous Na_2_SO_4_ and evaporated in vacuum to dryness. The purification of the crude product was achieved by column chromatography (silica gel mesh size 230–240; eluent 50–100% EtOAc/hexane). Characterization data for one representative compound from each series is presented here.

5-(2-Oxopyrrolidin-1-yl)-6,6a-dihydroisoindolo[2,1-*a*]quinolin-11(5*H*)-one (**1a**): Yield: 56%. White solid. mp: 225–227 °C; ^1^H NMR (CD_3_OD) δ 1.68–1.84 (m, 1H), 1.98–2.14 (m, 2H), 2.57–2.65 (m, 2H), 2.68–2.78 (m, 1H), 3.03–3.18 (m, 1H), 3.22–3.32 (m, 1H), 5.04 (d, *J* = 12.0 Hz, 1H), 5.73–5.75 (m, 1H), 7.10–7.25 (m, 2H), 7.32 (d, *J* = 6.6 Hz, 1H), 7.50–7.70 (m, 3H), 7.83 (d, *J* = 7.2 Hz, 1H), 8.45 (d, *J* = 8.1 Hz, 1H); ^13^C NMR (CD_3_OD) δ 18.9, 32.0, 32.2, 44.3, 50.0, 59.7, 121.5, 123.5, 124.7, 125.2, 125.7, 127.9, 129.3, 130.0, 133.0, 133.8, 137.8, 145.6, 167.8, 178.9; FTIR *ν*_max_ (NaCl): 3051, 2953, 2921, 1689, 1490, 1456, 1380, 1282, 1213, 1094, 906, 759, 730 cm^−1^; HRMS *m*/*z*: [M + Na]^+^ calcd for C_20_H_18_N_2_O_2_Na, 341.1260; found, 341.1252.

5-(2-Oxoazepan-1-yl)-6,6a-dihydroisoindolo[2,1-*a*]quinolin-11(5*H*)-one (**2a**): Yield: 47%. White solid. mp: 228–230 °C; ^1^H NMR (CD_3_OD) δ 1.30–1.40 (m, 1H), 1.48–1.90 (m, 6H), 2.50–2.67 (m, 1H), 2.68–2.85 (m, 2H), 2.90–3.34 (m, 2H), 5.03 (d, *J* = 12.0 Hz, 1H), 6.15–6.23 (m, 1H), 7.15 (d, *J* = 3.9 Hz, 2H), 7.28–7.37 (m, 1H), 7.52–7.60 (m, 1H), 7.68 (d, *J* = 3.9 Hz, 2H), 7.85 (d, *J* = 7.5 Hz, 1H), 8.43 (d, *J* = 8.7 Hz, 1H); ^13^C NMR (CD_3_OD) δ 24.5, 30.3, 30.7, 33.2, 38.1, 46.3, 53.0, 60.0, 121.7, 123.4, 124.8, 125.6, 126.5, 128.5, 129.1, 130.0, 133.2, 133.8, 138.4, 146.0, 167.8, 179.4; FTIR *ν*_max_ (NaCl): 3047, 2929, 2851, 1695, 1683, 1597, 1489, 1454, 1380, 1198, 1070, 759 cm^−1^; HRMS *m*/*z*: [M + Na]^+^ calcd for C_22_H_22_N_2_O_2_Na, 369.1573; found, 369.1560.

(*E*)-2-(2-Fluorophenyl)-3-(2-(2-oxopyrrolidin-1-yl)vinyl)isoindolin-1-one (**3a**): Yield: 39%. Colorless solid, mp 260–262 °C; ^1^H NMR (CDCl_3_) δ 1.98–2.10 (m, 2H), 2.41–2.48 (m, 2H), 3.24–3.44 (m, 2H), 4.62 (dd, *J* = 9.6, 14.1 Hz, 1H), 5.55 (d, *J* = 9.6 Hz, 1H), 7.12–7.33 (m, 4H), 7.40–7.47 (m, 1H), 7.57 (dt, *J* = 7.2, 26.4 Hz, 2H), 7.94 (d, *J* = 7.2 Hz, 1H); ^13^C NMR (CDCl_3_) δ 17.5, 31.1, 45.2, 64.2, 107.5, 116.6, 116.9, 123.4, 124.4, 124.6, 125.0, 125.2, 128.9 (2C), 129.9, 131.7, 132.4, 145.8, 156.6, 159.9, 167.3, 173.3; FTIR *ν*_max_ (NaCl): 3068, 2937, 1701, 1655, 1503, 1407, 1375, 1269, 758 cm^−1^; HRMS *m*/*z*: [M + Na]^+^ calcd for C_20_H_17_FN_2_O_2_Na, 359.1166; found, 359.1149.

## Supporting Information

File 1 Synthetic procedure and characterization data for all compounds and crystallographic data for **1a**, **1h**, **2b** and **3a**.
