# Supplementary material for: Aza-Diels–Alder reaction between N-aryl-1-oxo-1H-isoindolium ions and tert-enamides: Steric effects on reaction outcome
Source: Beilstein J Org Chem. 2014 Apr 14;10:848–57. doi: 10.3762/bjoc.10.81 (PMC3999823; doi:10.3762/bjoc.10.81)
Supplement: File 1 — Synthetic procedure and characterization data for all compounds and crystallographic data for 1a, 1h, 2b and 3a. [file Beilstein_J_Org_Chem-10-848-s001.pdf]

Supporting Information

for

Aza-Diels–Alder reaction between *N*-aryl-1-oxo-1*H*-isoindolium ions and *tert*-enamides: Steric effects on reaction outcome

Amitabh Jha[1,*§], Ting-Yi Chou[1], Zainab ALJaroudi[1], Bobby D. Ellis[1] and T. Stanley Cameron[2]

Address: [1]Department of Chemistry, Acadia University, Wolfville, NS, Canada and [2]Department of Chemistry, Dalhousie University, Halifax, NS, Canada

* Corresponding author

[§]Phone: +1-902-585-1515; Fax: +1-902-585-1114

Email: Amitabh Jha - ajha@acadiau.ca

Synthetic procedure and characterization data for all compounds and crystallographic data for 1a, 1h, 2b and 3a

Contents

1. Experimental S2
2. General procedure for the synthesis of *N*-aryl-*1H*-pyrrole-2,5-diones and their spectral data S2
3. General procedure for the synthesis of *N*-aryl-3-hydroxyisoindolinones and their spectral data S5
4. General procedure for the synthesis of 5-(2-oxopyrrolidin-1-yl)-6,6a-dihydroisoindolo[2,1-*a*]quinolin-11(5*H*)-ones (**1a**–**h**), 5-(2-oxoazepan-1-yl)-6,6a-dihydroisoindolo[2,1-*a*]quinolin-11(5*H*)-ones (**2a**–**h**) and *(E)*-2-(2-substitued-phenyl)-3-(2-(2-oxopyrrolidin-1-yl)vinyl)isoindolin-1-ones (**3a**–**i**), and their spectral data S10
5. ^{1}H and ^{13}C NMR spectra of 5-(2-oxopyrrolidin-1-yl)-6,6a-dihydro-

isoindolo[2,1-*a*]quinolin-11(5*H*)-ones (**1a**–**h**) S23
6. ^{1}H and ^{13}C NMR spectra of 5-(2-oxoazepan-1-yl)-6,6a-dihydro-isoindolo[2,1-*a*]quinolin-11(5*H*)-ones (**2a**–**h**) S31
7. ^{1}H and ^{13}C NMR spectra of *(E)*-2-(2-substitued-phenyl)-3-(2-(2-oxopyrrolidin-1-yl)vinyl) isoindolin-1-ones (**3a**–**i**) S39
8. ^{1}H and ^{13}C NMR spectra of intermediate *N*-aryl-1*H*-pyrrole-2,5-diones S48
9. ^{1}H and ^{13}C NMR spectra of intermediate *N*-aryl-3-hydroxy-isoindolinones S65
10. X-ray crystal structure of compound **1b** S82
11. X-ray crystal structure of compound **1h** S86
12. X-ray crystal structure of compound **2b** S90
13. X-ray crystal structure of compound **3a** S94
14. References S98

Experimental

General information

All reagents were purchased from Aldrich Chemical Co. and were used without purification. Melting points were recorded on a MEL-TEMP II apparatus and are uncorrected. TLCs were performed on pre-coated Merck silica gel $60F_{254}$ plates with the spots detected under UV light. Silica gel (100–200 mesh) was used for column chromatography. ^{1}H and ^{13}C NMR spectra were recorded on Bruker AC-300 Avance spectrometer at 300 MHz and 75 MHz respectively in $CDCl_3$, Acetone-d_6, CD_3OD or DMSO-d_6. ^{1}H NMR spectra were reported relative to residual $CHCl_3$ (δ 7.26), acetone (δ 2.05), methanol (δ 3.31) or DMSO (δ 2.50). ^{13}C NMR spectra were reported relative to $CHCl_3$ (δ 77.16), acetone (δ 29.92), methanol (δ 49.15) or DMSO (δ 39.52). IR spectra were recorded on Nicolet Avatar 330FT-IT spectrophotometer. High-Resolution Mass Spectra (HRMS) were recorded in positive ion mode on an Ion Spec Fourier transform mass spectrometer.

General procedure for the synthesis of *N*-aryl-*1H*-pyrrole-2,5-diones

Phthalic anhydride (10 mmol) was mixed with the corresponding anilines (12 mmol) in

dichloromethane (50 ml) for 30 minutes at room temperature to form the corresponding 2-(arylcarbamoyl)benzoic acid. Vacuum filtration gave excellent yields of the products. The solids were then dried, dissolved in acetic anhydride and were heated at 90 °C for 2–4 hours with freshly fused anhydrous sodium acetate as a catalyst (15 mol %). The solvent was evaporated under low pressure and the crude solids were dissolved in dichloromethane to assist in removal of insoluble sodium acetate by filtration. The organic solvent was evaporated under vacuum and the solid thus obtained was recrystallized from hot methanol to yield pure *N*-aryl-*1H*-pyrrole-2,5-diones.

2-Phenylisoindoline-1,3-dione: Yield: 88%. Mp: 201-203 ^{o}C (lit. mp 208-210 ^{o}C [1]). Its ^{1}H and ^{13}C NMR spectra were found to be identical to the literature report [1].

2-*p*-Tolylisoindoline-1,3-dione: Yield: 95%. Mp: 199-201 ^{o}C (lit. mp 207-209 ^{o}C [1]). Its ^{1}H and ^{13}C NMR spectra were found to be identical to the literature report [1].

2-(4-Methoxyphenyl)isoindoline-1,3-dione: Yield: 72%. Mp: 160-162 ^{o}C (lit. mp 143-145 ^{o}C [1]). Its ^{1}H and ^{13}C NMR spectra were found to be identical to the literature report [1].

2-(4-Chlorophenyl)isoindoline-1,3-dione: Yield: 78%. Mp: 194-195 ^{o}C (lit. mp 194-196 ^{o}C [1]). Its ^{1}H and ^{13}C NMR spectra were found to be identical to the literature report [1].

2-(3,5-Dimethylphenyl)isoindoline-1,3-dione: Yield: 96%. Mp: 132-133 ^{o}C (lit. mp 133 ^{o}C [2]). Its ^{1}H NMR spectra were found to be identical to the literature report [2].

2-(3-Chloro-4-fluorophenyl)isoindoline-1,3-dione: Yield: 73%. Mp: 185-186°C. ^{1}H NMR

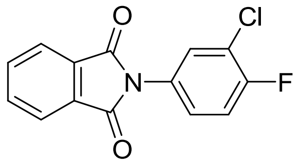

($CDCl_3$): δ 7.22-7.32 (m, 1H), 7.33-7.40 (m, 1H), 7.56 (dd, *J*= 2.4, 6.5Hz, 1H), 7.81 (dd, *J*= 3.0, 5.4Hz, 2H), 7.96 (dd, *J*= 3.0, 5.4Hz, 2H). ^{13}C NMR ($CDCl_3$): δ 117.0 (d, *J*=22.5 Hz), 121.8 (d, *J*=18.7 Hz), 124.1, 126.4 (d, *J*=7.5 Hz), 128.65, 129.0, 131.9, 134.8, 157.7 (d, *J*=249.0 Hz), 166.9.

2-(Naphthalen-1-yl)isoindoline-1,3-dione: Yield: 77%. Mp: 183-185 °C (lit. mp 159-161 °C [3]). Its ^{1}H and ^{13}C NMR spectra were found to be identical to the literature report [3].

2-(Naphthalen-2-yl)isoindoline-1,3-dione: Yield: 75%. Mp: 213-215 °C (lit. mp 214-216 °C [3]). Its ^{1}H and ^{13}C NMR spectra were found to be identical to the literature report [3].

2-(2-Fluorophenyl)isoindoline-1,3-dione: Yield: 74%. Mp: 188-190 °C (lit. mp 194-195 °C [4]). Its ^{1}H and ^{13}C NMR spectra were found to be identical to the literature report [4].

2-(2-Chlorophenyl)isoindoline-1,3-dione: Yield: 70%. Mp: 141-142 °C (lit. mp 143-143.6 °C [4]). Its ^{1}H and ^{13}C NMR spectra were found to be identical to the literature report [4].

2-(2-Iodophenyl)isoindoline-1,3-dione: Yield: 83%. Mp: 188-190°C. ^{1}H NMR ($CDCl_3$): δ 7.20 (t,

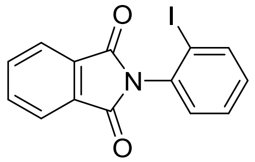

J= 7.5Hz, 1H), 7.32 (d, *J*= 7.5Hz, 1H), 7.51 (t, *J*= 7.5Hz, 1H), 7.79-7.85 (m, 2H), 7.96-8.02 (m, 3H). ^{13}C NMR ($CDCl_3$): δ 99.0, 124.0, 129.4, 130.4, 131.0, 132.2, 134.6, 135.4, 140.0, 166.5.

2-(2,5-Dibromophenyl)isoindoline-1,3-dione: Yield: 69%. Mp: 214-215°C. ^{1}H NMR ($CDCl_3$): δ

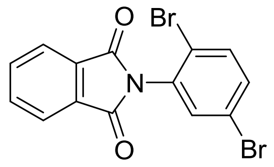

7.51 (d, *J*= 8.1Hz, 2H), 7.62 (d, *J*= 8.4Hz, 1H), 7.85 (dd, *J*= 3.0, 5.4Hz, 2H), 8.00 (dd, *J*= 3.3, 4.8Hz, 2H). ^{13}C NMR ($CDCl_3$): δ 121.7, 122.6, 124.3, 124.3, 132.2, 133.3, 134.2, 134.9, 134.9, 166.3.

2-(2-Nitrophenyl)isoindoline-1,3-dione: Yield: 81%. Mp: 195-197 °C (lit. mp 198-199 °C [2]). Its ^{1}H spectrum was found to be identical to the literature report [2].

2-(2-Ethylphenyl)isoindoline-1,3-dione: Yield: 69%. Mp: 133-134 °C (lit. mp 132-133 °C [2]). Its ^{1}H spectrum was found to be identical to the literature report [2].

2-(2-*tert*-Butylphenyl)isoindoline-1,3-dione: Yield: 55%. Mp: 119-121 °C (lit. mp 123 °C [2]). Its ^{1}H spectrum was found to be identical to the literature report [2].

2-(2-Methoxyphenyl)isoindoline-1,3-dione: Yield: 81%. Mp: 150-151 °C (lit. mp 158 °C [2]). Its ^{1}H spectrum was found to be identical to the literature report [2].

2-(2-(Trifluoromethoxy)phenyl)isoindoline-1,3-dione: Yield: 71%. Mp: 90-93°C. ^{1}H NMR

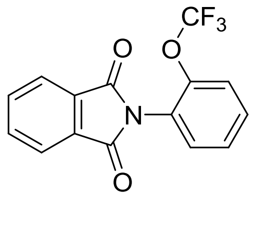

($CDCl_3$): δ 7.38-7.58 (m, 4H), 7.79-7.85 (m, 2H), 7.93-8.02 (m, 2H). ^{13}C NMR ($CDCl_3$): δ 119.0, 122.4, 124.2, 124.8, 127.5, 130.7, 130.9, 132.4, 134.7, 146.0, 166.6.

General procedure for the synthesis of *N*-aryl-3-hydroxyisoindolinones

Appropriate *N*-aryl-*1H*-pyrrole-2,5-diones (700 mg) from the previous step were taken in MeOH (75 ml) and the mixture was stirred in an ice bath for 10 minutes followed by the addition of powdered $NaBH_4$ (1.0 equiv) in portions over 10 minutes. The reaction progress was monitored by TLC. The reduction reaction took approximately 1 hour to go to completion. Once the TLC showed only one single spot which was more polar than the starting material, the remaining $NaBH_4$ was quenched by adding diluted HCl solution (0.5 N, 10 ml). The solvent was evaporated under reduced pressure. The residue was extracted with ethyl acetate (100 ml) and water (50 ml × 3). The organic layer was dried over anhydrous Na_2SO_4 and vacuum evaporated to dryness, yielding pure reduced products.

3-Hydroxy-2-phenylisoindolin-1-one: Yield: 78%. Mp: 167-168°C (lit. mp 167-168 ^{o}C [5]). Its ^{1}H NMR spectrum was found to be identical to the literature report [5].

3-Hydroxy-2-*p*-tolylisoindolin-1-one: Yield: 99%. Mp: 166-168°C. ^{1}H NMR ($CDCl_3$): δ 2.35 (s, 3H), 5.70-5.77 (m, 1H), 6.45-6.62 (m, 1H), 7.24 (d, *J*= 7.5Hz, 2H), 7.68-7.76 (m, 5H). ^{13}C NMR ($CDCl_3$): δ 20.3, 82.9, 122.7, 123.2, 123.7, 129.4, 129.9, 132.5, 132.7, 134.4, 136.0, 144.6, 165.9.

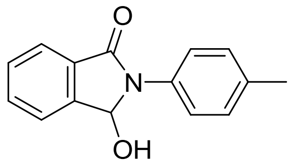

3-Hydroxy-2-(4-methoxyphenyl)isoindolin-1-one: Yield: 89%. Mp: 135-137 ^{o}C (lit. mp 147-149 ^{o}C [5]). Its ^{1}H NMR spectrum was found to be identical to the literature report [5].

2-(4-Chlorophenyl)-3-hydroxyisoindolin-1-one: Yield: 85%. Mp: 198-200 ^{o}C (lit. mp 198-200 ^{o}C [6]). Its ^{1}H NMR spectrum was found to be identical to the literature report [6].

2-(3,5-Dimethylphenyl)-3-hydroxyisoindolin-1-one: Yield: 85%. Mp: 133-135°C. ^{1}H NMR

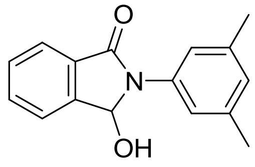

(CDCl$_3$): δ 2.10 (s, 3H), 2.33 (s, 3H), 3.02 (br s, 1H), 6.34 (m, 1H), 6.88 (s, 1H), 7.30 (s, 2H), 7.50 (t, *J*= 7.2Hz, 1H), 7.57-7.69 (m, 2H), 7.76 (d, *J*= 7.5Hz, 1H). ^{13}C NMR (CDCl$_3$): δ 21.6, 83.2, 122.2, 123.4, 124.2, 127.8, 130.5, 132.3, 132.9, 137.3, 138.1, 148.2, 166.4.

2-(3-Chloro-4-fluorophenyl)-3-hydroxyisoindolin-1-one: Yield: 87%. Mp: 163-165°C. ^{1}H NMR

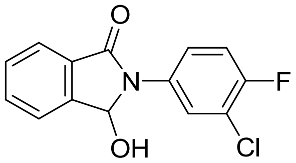

(DMSO-d$_6$): δ 3.62 (br s, 1H), 6.28 (m, 1H), 7.15 (t, *J*= 8.7Hz, 1H), 7.47 (t, *J*= 7.2Hz, 1H), 7.60-7.70 (m, 3H), 7.89 (d, *J*= 4.5Hz, 1H), 8.09 (s, 1H). ^{13}C NMR (DMSO-d$_6$): δ 83.1, 117.5 (d, *J*=21.8 Hz), 120.2 (d, *J*=18.0 Hz), 123.4 (d, *J*=6.5 Hz), 123.8, 124.4, 124.6, 130.5, 131.7, 133.8, 135.7, 145.1, 155.1 (d, *J*=243.0 Hz), 166.4.

3-Hydroxy-2-(naphthalen-1-yl)isoindolin-1-one: Yield: 96%. Mp: 178-180°C (lit. mp 179-181 ^{o}C [7]). Its ^{1}H NMR spectrum was found to be identical to the literature report [7].

3-Hydroxy-2-(naphthalen-2-yl)isoindolin-1-one: Yield: 90%. Mp: 209-210°C. ^{1}H NMR (CD$_3$OD):

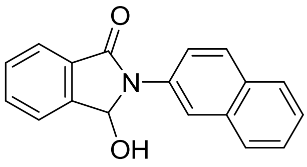

δ 6.61 (s, 1H), 7.45-7.56 (m, 2H), 7.60-7.69 (m, 1H), 7.72-7.78 (m, 2H), 7.86-7.99 (m, 6H), 8.20 (br s, 1H). ^{13}C NMR (CD$_3$OD): δ 83.5,

121.5, 122.7, 123.3, 123.6, 125.8, 126.4, 127.6, 127.8, 128.6, 130.0, 131.9, 133.1, 134.2, 134.9, 138.0, 144.2, 167.8.

2-(2-Fluorophenyl)-3-hydroxyisoindolin-1-one: Yield: 98%. Mp: 173-175°C. ^{1}H NMR

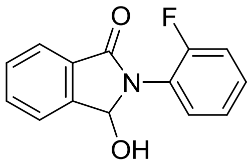

(DMSO-d_6): δ 4.71 (br s, 1H), 6.34 (d, *J*= 5.7Hz, 1H), 7.20-7.30 (m, 2H), 7.33-7.36 (m, 1H), 7.50-7.68 (m, 4H), 7.90 (d, *J*= 6.9Hz, 1H). ^{13}C NMR (DMSO-d_6): δ 63.3, 115.3 (d, *J*=19.2 Hz), 124.3 (d, *J*=15.8 Hz), 125.6 (d, *J*=7.5 Hz), 127.1, 127.9, 129.1, 130.0, 131.0, 135.7, 136.1, 140.0, 154.8 (d, *J*=249.0 Hz), 167.7.

2-(2-Chlorophenyl)-3-hydroxyisoindolin-1-one: Yield: 67%. Mp: 172-173°C. ^{1}H NMR

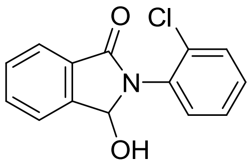

(DMSO-d_6): δ 6.17 (d, *J*= 8.7Hz, 1H), 6.88 (d, *J*= 7.8Hz, 1H), 7.40-7.46 (m, 3H), 7.55-7.75 (m, 5H). ^{13}C NMR (DMSO-d_6): δ 83.7, 123.7, 124.7, 128.6, 130.4, 130.6, 131.7, 132.5, 133.5, 135.0, 137.0, 139.2, 146.0, 166.3.

3-Hydroxy-2-(2-iodophenyl)isoindolin-1-one: Yield: 88%. Mp: 176-177°C. ^{1}H NMR ($CDCl_3$): δ

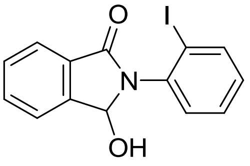

2.96 (br s, 1H), 6.19 (d, *J*= 3.9Hz, 1H), 7.15 (t, *J*= 7.5Hz, 1H), 7.32 (d, *J*= 7.5Hz, 1H), 7.44 (t, *J*= 7.5Hz, 1H), 7.54-7.62 (m, 1H), 7.64-7.70 (m, 2H), 7.86 (d, *J*= 7.2Hz, 1H), 7.96 (d, *J*= 7.8Hz, 1H). ^{13}C NMR ($CDCl_3$): δ 84.1, 99.0, 123.7, 124.5, 129.5, 130.4, 131.3, 131.8, 133.0, 137.2, 139.2, 140.1, 144.0, 166.2.

2-(2,5-Dibromophenyl)-3-hydroxyisoindolin-1-one: Yield: 77%. Mp: 161-163°C. ^{1}H NMR

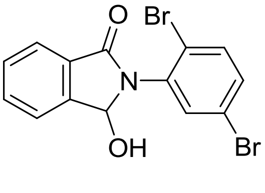

($CDCl_3$): δ 6.24 (br s, 1H), 6.96 (d, *J*= 9.3Hz, 1H), 7.57-7.85 (m, 5H),

7.94-8.03 (m, 2H). ^{13}C NMR ($CDCl_3$): δ 83.7, 121.4, 123.6, 124.7, 130.5, 131.4, 132.3, 133.5, 135.0, 135.2, 136.0, 138.2, 145.9, 166.7.

3-Hydroxy-2-(2-nitrophenyl)isoindolin-1-one: Yield: 68%. Mp: 177-179°C. ^{1}H NMR (CD_3OD): δ

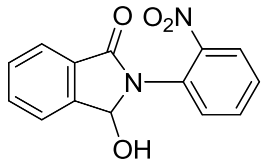

3.32 (m, 1H), 6.32 (m, 1H), 7.60-7.88 (m, 7H), 8.12 (d, *J*= 8.1Hz, 1H). ^{13}C NMR (CD_3OD): δ 84.6, 123.5, 123.8, 125.4, 129.0, 130.0, 130.2, 130.8, 131.0, 133.5, 134.0, 145.3, 147.5, 168.1.

2-(2-Ethylphenyl)-3-hydroxyisoindolin-1-one: Yield: 68%. Mp: 147-148°C. ^{1}H NMR

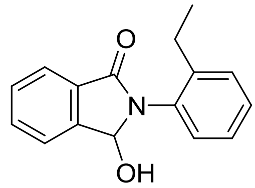

(Acetone-d_6): δ 1.19 (t, *J*= 7.2Hz, 3H), 2.63 (q, *J*= 7.2Hz, 2H), 5.81 (m, 1H), 6.29 (br s, 1H), 7.19-7.80 (m, 8H). ^{13}C NMR (Acetone-d_6): δ 14.1, 24.2, 84.6, 123.2, 124.0, 126.4, 126.5, 128.3, 129.0, 129.6, 129.8, 132.4, 135.8, 143.8, 145.6, 166.2.

2-(2-*tert*-Butylphenyl)-3-hydroxyisoindolin-1-one: Yield: 68%. Mp: 126-128°C. ^{1}H NMR

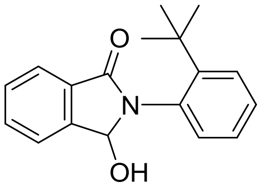

(Acetone-d_6): δ 1.41 (s, 9H), 4.77 (m, 1H), 5.15 (m, 1H), 7.23-7.30 (m, 2H), 7.35-7.39 (m, 1H), 7.41-7.57 (m, 3H), 7.78-7.84 (m, 1H), 9.49 (br s, 1H). ^{13}C NMR (Acetone-d_6): δ 31.2, 35.1, 63.4, 126.7, 127.0, 127.3, 127.9, 128.4, 130.0, 130.9, 131.4, 136.2, 136.5, 140.8, 146.5, 168.1.

3-Hydroxy-2-(2-methoxyphenyl)isoindolin-1-one: Yield: 89%. Mp: 178-180°C (lit. mp 178-179 °C [5]). Its ^{1}H NMR spectrum was found to be identical to the literature report [5].

3-Hydroxy-2-(2-(trifluoromethoxy)phenyl)isoindolin-1-one: Yield: 55%. Semi-solid. ^{1}H NMR

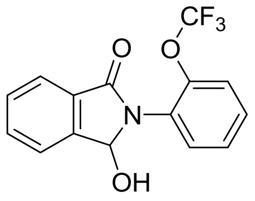

($CDCl_3$): δ 3.57 (br s, 1H), 6.22 (s, 1H), 7.25-7.55 (m, 5H), 7.60-7.62 (m, 2H), 7.69 (d, *J*= 7.2Hz, 1H). ^{13}C NMR ($CDCl_3$): δ 83.8, 120.8 (q, *J*=265.5Hz), 121.4, 123.8, 124.3, 127.5, 128.8, 129.4, 130.4, 131.3, 133.1, 134.2, 144.3, 146.0, 166.8.

General procedure for the synthesis of 5-(2-oxopyrrolidin-1-yl)-6,6a-dihydroisoindolo[2,1-*a*]quinolin-11(5*H*)-ones (1a–h); 5-(2-oxoazepan-1-yl)-6,6a-dihydroisoindolo[2,1-*a*]quinolin-11(5*H*)-ones (2a–h) and *(E)*-2-(2-substitued-phenyl)-3-(2-(2-oxopyrrolidin-1-yl)vinyl)isoindolin-1-ones (3a–i)

Appropriate *N*-aryl-3-hydroxyisoindolinones (500 mg) from the previous step were dissolved in dichloromethane (10 ml) in a 50 ml round bottom flask which was then sealed with a rubber septum. BF_3:Et_2O (1.5 equiv) was added to the mixture slowly through the septum by a syringe. This resulted in the formation of a transparent solution. Appropriate *tert*-enamide (1.5 equiv) dissolved in dichloromethane (5 ml) was slowly added to the flask through a syringe over a period of 5 minutes. The reaction was then allowed to stir at room temperature until the precipitates formed. The reaction progress was monitored by TLC. Upon completion of the reaction, the residue was suction filtered and the solid obtained was extracted with ethyl acetate (60 ml) and washed with water (30 ml × 3). The organic layer was dried over anhydrous Na_2SO_4 and evaporated in vacuum to dryness to obtain pure products. In the cases where precipitates did not form, the solvents were evaporated to dryness under reduced pressure. The residue was extracted by ethyl acetate (60 ml) and washed with water (30 ml × 3). The organic layer was dried over

anhydrous Na_2SO_4 and evaporated in vacuum to dryness. The purification of the crude product was achieved by column chromatography (silica gel mesh size 230–240; eluent 50–100% EtOAc/hexane).

5-(2-Oxopyrrolidin-1-yl)-6,6a-dihydroisoindolo[2,1-*a*]quinolin-11(5*H*)-one (**1a**): Yield: 56%.

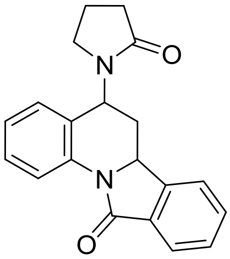

White solid. Mp: 225-227°C. ^{1}H NMR (CD_3OD): δ 1.68-1.84(m, 1H), 1.98-2.14(m, 2H), 2.57-2.65(m, 2H), 2.68-2.78(m, 1H), 3.03-3.18(m, 1H), 3.22-3.32(m, 1H), 5.04(d, *J*= 12.0Hz, 1H), 5.73-5.75(m, 1H), 7.10-7.25(m, 2H), 7.32(d, *J*= 6.6Hz, 1H), 7.50-7.70(m, 3H), 7.83 (d, *J*= 7.2Hz, 1H), 8.45 (d, *J*= 8.1Hz, 1H). ^{13}C NMR (CD_3OD): δ 18.9, 32.0, 32.2, 44.3, 50.0, 59.7, 121.5, 123.5, 124.7, 125.2, 125.7, 127.9, 129.3, 130.0, 133.0, 133.8, 137.8, 145.6, 167.8, 178.9. FT-IR ν_{max} (NaCl): 3051, 2953, 2921, 1689, 1490, 1456, 1380, 1282, 1213, 1094, 906, 759, 730 cm^{-1}. HRMS (amu): calcd for $C_{20}H_{18}N_2O_2$ $[M+Na]^+$: 341.1260; found $[M+Na]^+$: 341.1252.

3-Methyl-5-(2-oxopyrrolidin-1-yl)-6,6a-dihydroisoindolo[2,1-*a*]quinolin-11(5*H*)-one (**1b**):

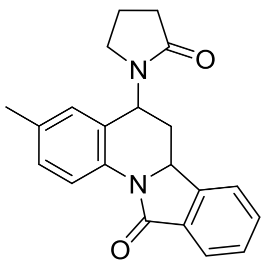

Yield: 62%. White solid. Mp: 202-204°C. ^{1}H NMR ($CDCl_3$): δ 1.68-1.78(m, 1H), 2.01-2.10(m, 2H), 2.35(s, 3H), 2.50-2.61(m, 2H), 2.62-2.71(m, 1H), 3.11(t, *J*= 6.9Hz , 2H), 4.92(d, *J*= 12.0Hz, 1H), 5.80 (dd, *J*= 6.3, 12.0Hz, 1H), 6.90(s, 1H), 7.20(d, *J*= 8.4Hz, 1H), 7.48-7.66(m, 3H), 7.96(d, *J*= 7.5Hz, 1H), 8.45(d, *J*= 8.4Hz, 1H). ^{13}C NMR ($CDCl_3$): δ 18.3, 21.2, 31.4, 31.8, 42.5, 48.2, 58.4, 120.8, 122.0, 123.6, 124.5, 127.2, 129.0, 129.4, 132.3, 132.9, 134.2, 134.6, 143.9, 165.8, 175.9. FT-IR ν_{max} (NaCl): 3047, 2921, 1682, 1499, 1458, 1421, 1383, 1343, 1286, 1213,

1168, 1098, 730 cm^{-1}. HRMS (amu): calcd for $C_{21}H_{20}N_2O_2$ [M+Na]$^+$: 355.1422; found [M+Na]$^+$: 355.1418.

3-Methoxy-5-(2-oxopyrrolidin-1-yl)-6,6a-dihydroisoindolo[2,1-*a*]quinolin-11(5*H*)-one (**1c**):

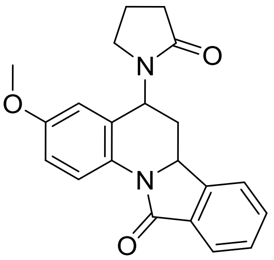

Yield: 82%. White solid. Mp: 227-230°C. ^{1}H NMR (CDCl$_3$): δ 1.65-1.81(m, 1H), 1.95-2.10(m, 2H), 2.40-2.60(m, 2H), 2.61-2.70(m, 1H), 3.07-3.15(m, 2H), 3.79(s, 3H), 4.88(d, *J*= 12.0Hz, 1H), 5.78(dd, *J*= 5.7, 11.1Hz, 1H), 6.62(s, 1H), 6.92(d, *J*= 7.5Hz, 1H), 7.51(dd, *J*= 7.2, 15.3Hz, 2H), 7.60(d, *J*= 7.2Hz, 1H), 7.92(d, *J*= 6.9Hz, 1H), 8.48(d, *J*= 9.0Hz, 1H). ^{13}C NMR (CDCl$_3$): δ 18.3, 31.2, 31.7, 42.5, 48.3, 55.7, 58.3, 112.4, 113.9, 121.9, 122.1, 124.4, 125.4, 129.0, 130.5, 132.2, 132.8, 143.7, 156.6, 165.6, 175.8. FT-IR v_{max} (NaCl): 3098, 2921, 1682, 1497, 1417, 1384, 1283, 1232, 1098, 1066, 906, 728 cm^{-1}. HRMS (amu): calcd for $C_{21}H_{20}N_2O_3$ [M+Na]$^+$: 371.1366; found [M+Na]$^+$: 371.1361.

3-Chloro-5-(2-oxopyrrolidin-1-yl)-6,6a-dihydroisoindolo[2,1-*a*]quinolin-11(5*H*)-one (**1d**): Yield:

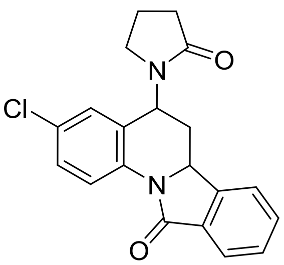

41%. White solid. Mp: 229-230°C. ^{1}H NMR (CDCl$_3$): δ 1.77-1.91(m, 1H), 2.02-2.13(m, 2H), 2.46-2.64(m, 2H), 2.71-2.78(m, 1H), 3.07-3.17(m, 1H), 3.29-3.37(m, 1H), 5.13(d, *J*= 12.3Hz, 1H), 5.75(dd, *J*= 6.3, 11.8Hz, 1H), 7.12(s, 1H), 7.37(d, *J*= 9.0Hz, 1H), 7.56-7.64(m, 1H), 7.70-7.74(m, 2H), 7.89(d, *J*= 7.5Hz, 1H), 8.52(d, *J*= 8.7Hz, 1H). ^{13}C NMR (CDCl$_3$): δ 18.4, 31.2, 31.4, 42.5, 48.1, 58.3, 122.1, 122.2, 124.7, 125.8, 128.9, 129.2, 130.0, 132.4, 132.7, 135.6, 143.7, 166.0, 176.0. FT-IR v_{max} (NaCl): 3043, 2953, 2917, 1686, 1593, 1482, 1380, 1343, 1282, 1164, 1094,

1025, 902, 755, 730, 698 cm^{-1}. HRMS (amu): calcd for $C_{20}H_{17}ClN_2O_2$ $[M+Na]^+$: 375.0871; found $[M+Na]^+$: 375.0878.

2,4-Dimethyl-5-(2-oxopyrrolidin-1-yl)-6,6a-dihydroisoindolo[2,1-*a*]quinolin-11(5*H*)-one (**1e**):

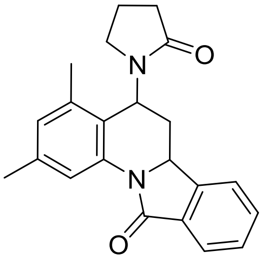

Yield: 59%. White solid. Mp: 232-235°C. ^1H NMR ($CDCl_3$): δ 1.55-1.69(m, 1H), 1.88-1.94(m, 2H), 2.14(s, 3H), 2.32-2.50(m, 5H), 2.67-2.77(m, 1H), 2.87-3.03(m, 2H), 4.75(d, *J*= 12.6Hz, 1H), 5.73(t, *J*= 9.0Hz, 1H), 6.82(s, 1H), 7.44-7.7.64(m, 3H), 7.95(d, *J*= 7.5Hz, 1H), 8.19(s, 1H). ^{13}C NMR ($CDCl_3$): δ 17.9, 19.5, 21.1, 30.8, 33.0, 42.0, 46.2, 57.8, 119.2, 119.6, 121.9, 124.3, 128.0, 128.7, 132.1, 132.8, 137.7(2C), 138.3, 143.9, 165.2, 174.2. FT-IR ν_{max} (NaCl): 3043, 2953, 2921, 1698, 1605, 1572, 1420, 1379, 1282, 1213, 1090, 1033, 853, 759, 735, 702 cm^{-1}. HRMS (amu): calcd for $C_{22}H_{22}N_2O_2$ $[M+Na]^+$: 369.1573; found $[M+Na]^+$: 369.1563.

2-Chloro-3-fluoro-5-(2-oxopyrrolidin-1-yl)-6,6a-dihydroisoindolo[2,1-*a*]quinolin-11(5*H*)-one (**1f**):

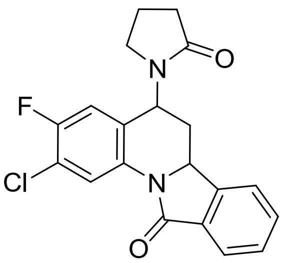

Yield: 33%. White solid. Mp: >260°C. ^1H NMR ($CDCl_3$): δ 1.63-1.82(m, 1H), 2.00-2.19(m, 2H), 2.40-2.62(m, 2H), 2.63-2.72(m, 1H), 3.00-3.20(m, 2H), 4.92(d, *J*= 12.3Hz, 1H), 5.77(t, *J*= 5.4Hz, 1H), 6.87(d, *J*= 9.0Hz, 1H), 7.45-7.61(m, 2H), 7.64(d, *J*= 7.2Hz, 1H), 7.94(d, *J*= 6.9Hz, 1H), 8.79(d, *J*= 6.6Hz, 1H). ^{13}C ($CDCl_3$) NMR: δ 18.5, 31.2, 31.4, 42.6, 48.2, 58.4, 114.4, 114.7, 121.2, 121.5, 122.2, 122.9, 124.9, 129.5, 132.3, 132.9, 133.7, 143.7, 166.0, 176.0. FT-IR ν_{max} (NaCl): 3041, 2962, 2892, 1680, 1484, 1409, 1384, 1288, 1215, 1080, 1027, 890, 726, 681 cm^{-1}. HRMS (amu): calcd for $C_{20}H_{16}ClFN_2O_2$ $[M+Na]^+$: 393.0777; found $[M+Na]^+$: 393.0770.

7-(2-Oxopyrrolidin-1-yl)-8,8a-dihydrobenzo[*h*]isoindolo[2,1-*a*]quinolin-13(7*H*)-one (**1g**): Yield:

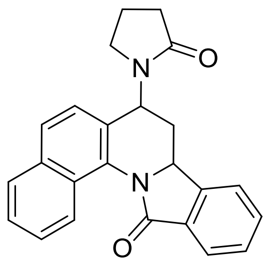

47%. Pale purple needle-like crystal. Mp: 197-199°C. ^{1}H NMR ($CDCl_3$): δ 1.64-1.79(m, 1H), 1.95-2.10(m, 2H), 2.40-2.65(m, 2H), 2.76-2.87(m, 1H), 3.08-3.17(t, *J*= 6.9Hz, 2H), 5.09(d, *J*= 10.5Hz, 1H), 5.93(dd, *J*= 3.3Hz, 10.6Hz, 1H), 7.13-7.28(m, 2H), 7.42-7.70(m, 5H), 7.75(d, *J*= 8.7Hz, 1H), 7.81-7.86(m, 1H), 8.02(d, *J*= 6.9Hz, 1H). ^{13}C NMR ($CDCl_3$): δ 18.4, 31.4, 35.1, 42.7, 48.6, 59.8, 122.2, 124.0, 124.1, 125.2, 125.8, 126.7, 126.8, 127.3, 127.6, 128.0, 129.1, 132.2, 132.5, 133.5, 134.4, 146.5, 166.0, 176.3. FT-IR ν_{max} (NaCl): 3051, 2925, 1681, 1613, 1470, 1397, 1282, 1209, 1123, 1025, 820, 755, 730cm^{-1}. HRMS (amu): calcd for $C_{24}H_{20}N_2O_2$ [M+Na]$^+$: 391.1417; found [M+Na]$^+$: 391.1398.

7-(2-Oxopyrrolidin-1-yl)-8,8a-dihydrobenzo[*f*]isoindolo[2,1-*a*]quinolin-13(7*H*)-one (**1h**): Yield:

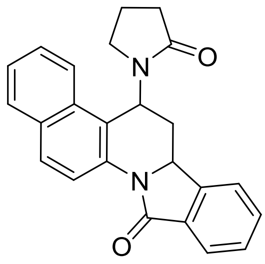

50%. White solid. Mp: 242-244°C. ^{1}H NMR ($CDCl_3$): δ 1.69-1.83(m, 3H), 2.31-2.45(m, 3H), 2.88-2.98(m, 1H), 3.09-3.19(m, 1H), 4.86(dd, *J*=2.7, 13.0Hz, 1H), 6.25(t, *J*= 9.0Hz, 1H), 7.43-7.60(m, 4H), 7.62-7.69(m, 1H), 7.76(d, *J*= 8.4Hz, 1H), 7.83-7.93(m, 2H), 7.99(d, *J*= 7.5Hz, 1H), 8.72(d, *J*= 9.0Hz, H). ^{13}C NMR ($CDCl_3$): δ 18.2, 31.2, 33.4, 42.3, 46.0, 58.0, 117.6, 120.0, 122.2, 123.2, 124.8, 125.3, 127.5, 129.0, 129.9, 131.1, 131.8, 132.6, 132.9, 136.8, 144.1, 165.8, 174.9. FT-IR ν_{max} (NaCl): 3047, 2974, 2921, 2876, 1677, 1617, 1515, 1470, 1404, 1356, 1298, 1210, 1172, 824, 747, 686 cm^{-1}. HRMS (amu): calcd for $C_{24}H_{20}N_2O_2$ [M+Na]$^+$: 391.1417; found [M+Na]$^+$: 391.1409.

5-(2-Oxoazepan-1-yl)-6,6a-dihydroisoindolo[2,1-*a*]quinolin-11(5*H*)-one (**2a**): Yield: 47%.

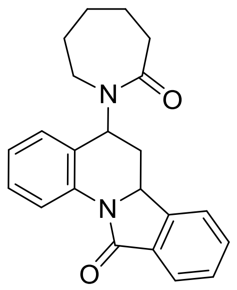

White solid. Mp: 228-230°C. ^{1}H NMR (CD_3OD): δ 1.30-1.40(m, 1H), 1.48-1.90(m, 6H), 2.50-2.67(m, 1H), 2.68-2.85(m, 2H), 2.90-3.34(m, 2H), 5.03(d, *J*= 12.0Hz, 1H), 6.15-6.23(m, 1H), 7.15(d, *J*= 3.9Hz, 2H),7.28-7.37(m, 1H), 7.52-7.60(m, 1H), 7.68(d, *J*= 3.9Hz, 2H), 7.85(d, *J*= 7.5Hz, 1H), 8.43(d, *J*= 8.7Hz, 1H). ^{13}C NMR (CD_3OD): δ 24.5, 30.3, 30.7, 33.2, 38.1, 46.3, 53.0, 60.0, 121.7, 123.4, 124.8, 125.6, 126.5, 128.5, 129.1, 130.0, 133.2, 133.8, 138.4, 146.0, 167.8, 179.4. FT-IR ν_{max} (NaCl): 3047, 2929, 2851, 1695, 1683, 1597, 1489, 1454, 1380, 1198, 1070, 759 cm^{-1}. HRMS (amu): calcd for $C_{22}H_{22}N_2O_2$ [M+Na]$^+$: 369.1573; found [M+Na]$^+$: 369.1560.

3-Methyl-5-(2-oxoazepan-1-yl)-6,6a-dihydroisoindolo[2,1-*a*]quinolin-11(5*H*)-one (**2b**): Yield:

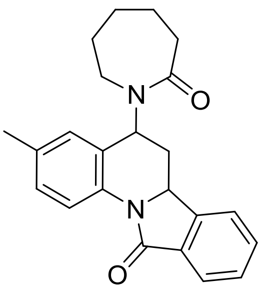

80%. White solid. Mp: 218-221°C. ^{1}H NMR (CD_3OD): δ 1.16-1.30(m, 1H), 1.40-1.85(m, 6H), 2.28(s, 3H), 2.45-2.55(m, 2H), 2.61-2.99(m, 3H), 3.08-3.20(m, 1H), 5.13(d, *J*= 11.7Hz, 1H), 6.09(s, 1H), 6.92(s, 1H), 7.14(d, *J*= 8.1Hz, 1H), 7.57(t, *J*= 7.2Hz, 1H), 7.69(t, *J*= 7.2Hz, 1H), 7.79(t, *J*= 6.6Hz, 2H), 8.33(d, *J*= 8.4Hz, 1H). ^{13}C NMR (CD_3OD): δ 21.0, 24.5, 30.3, 30.7, 33.3, 38.1, 46.3, 53.0, 60.0, 121.6, 123.5, 124.8, 126.3, 128.7, 129.8, 130.0, 133.3, 133.7, 135.6, 135.9, 145.9, 167.7, 179.4. FT-IR ν_{max} (NaCl): 3051, 2925, 2855, 1694, 1639, 1499, 1383, 1294, 1192, 1029, 972, 820, 726 cm^{-1}. HRMS (amu): calcd for $C_{23}H_{24}N_2O_2$ [M+H]$^+$: 361.1911; found [M+H]$^+$: 361.1894.

3-Methoxy-5-(2-oxoazepan-1-yl)-6,6a-dihydroisoindolo[2,1-*a*]quinolin-11(5*H*)-one (**2c**): Yield:

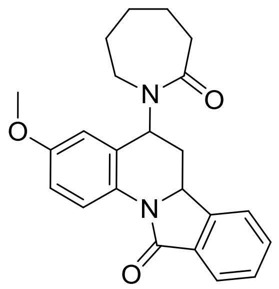

48%. White solid. Mp: 216-217°C. ^{1}H NMR (CD_3OD): δ 1.25-1.36(m, 1H), 1.56-1.90(m, 6H), 2.60-2.80(m, 3H), 2.90-3.31(m, 1H), 3.10-3.21(m, 1H), 3.82(s, 3H), 4.88(d, *J*= 12.0Hz, 1H), 6.29(dd, *J*= 6.0, 11.7Hz, 1H), 6.70(s, 1H), 6.94(d, *J*= 9.0Hz, 1H), 7.54(dd, *J*= 7.5, 14.4Hz, 3H), 7.62(d, *J*= 14.4Hz, 1H), 7.96(d, *J*= 7.5Hz, 1H), 8.48(d, *J*= 9.0Hz, 1H). ^{13}C NMR (CD_3OD): δ 23.7, 29.7, 30.1, 32.6, 37.7, 45.2, 51.6, 55.7, 58.5, 112.9, 113.8, 122.0, 122.1, 124.4, 126.6, 129.0, 131.0, 132.2, 132.9, 144.0, 156.6, 165.6, 176.9. FT-IR ν_{max} (NaCl): 3047, 2929, 2851, 1692, 1639, 1497, 1384, 1280, 1232, 1188, 1070, 1030, 976, 735 cm^{-1}. HRMS (amu): calcd for $C_{23}H_{24}N_2O_3$ $[M+Na]^+$: 399.1679; found $[M+Na]^+$: 399.1689.

3-Chloro-5-(2-oxoazepan-1-yl)-6,6a-dihydroisoindolo[2,1-*a*]quinolin-11(5*H*)-one (**2d**): Yield:

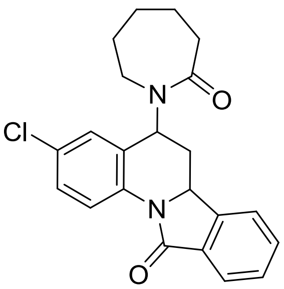

34%. White solid. Mp: 248-250°C. ^{1}H NMR ($CDCl_3$): δ 1.22-1.38(m, 1H), 1.52-1.78(m, 4H), 1.79-1.98(m, 2H), 2.65-2.81(m, 3H), 2.88-3.00(m, 1H), 3.12-3.25(m, 1H), 4.91(d, *J*= 12.3Hz, 1H), 6.25-6.36(m, 1H), 7.12(s, 1H), 7.28-7.38(m, 1H), 7.55(dd, *J*= 7.2, 13.5Hz, 2H), 7.65(d, *J*= 7.2Hz, 1H), 7.96(d, *J*= 7.2Hz, 1H), 8.52(d, *J*= 8.7Hz, 1H). ^{13}C NMR ($CDCl_3$): δ 23.7, 29.7, 30.0, 32.2, 37.6, 45.2, 51.2, 58.3, 122.1(2C), 124.6, 127.0, 127.2, 128.6, 129.2, 129.8, 132.4, 132.6, 136.0, 143.9, 166.0, 176.9. FT-IR ν_{max} (NaCl): 3047, 2929, 2851, 1692, 1639, 1497, 1384, 1280, 1232, 1190, 1070, 1029, 976, 820, 735 cm^{-1}. HRMS (amu): calcd for $C_{22}H_{21}ClN_2O_2$ $[M+Na]^+$: 403.1184; found $[M+Na]^+$: 403.1182.

2,4-Dimethyl-5-(2-oxoazepan-1-yl)-6,6a-dihydroisoindolo[2,1-*a*]quinolin-11(5*H*)-one (**2e**):

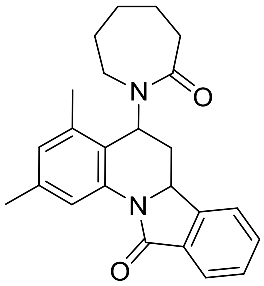

Yield: 33%. White solid. After three chromatographic purification attempts, the product still contained small amount of an impurity (apparent in its NMR spectra). ^{1}H NMR ($CDCl_3$): δ 1.23-1.98 (m, 7H), 2.20 (s, 3H), 2.28 (s, 3H), 2.33-2.38 (m, 3H), 2.56 (bs, 1H), 2.90-2.97 (m, 1H), 4.71(1H, d, *J*=12.6 Hz), 6.19-6.24 (m, 1H), 7.18-7.28 (m, 2H), 7.47-7.70 (m, 4H), 7.95-7.98 (m, 1H). ^{13}C NMR ($CDCl_3$): δ 19.5, 21.6, 23.5, 29.2, 30.2, 34.2, 37.7, 44.3, 49.9, 58.6, 119.4, 121.4, 122.2, 124.7, 127.4, 129.0, 130.3, 132.6, 132.9, 138.1, 138.6, 144.4, 165.1, 175.3. HRMS (amu): calcd for $C_{24}H_{26}N_2O_2$ $[M+Na]^+$: 397.1892; found $[M+Na]^+$: 397.1865.

2-Chloro-3-fluoro-5-(2-oxoazepan-1-yl)-6,6a-dihydroisoindolo[2,1-*a*]quinolin-11(5*H*)-one (**2f**):

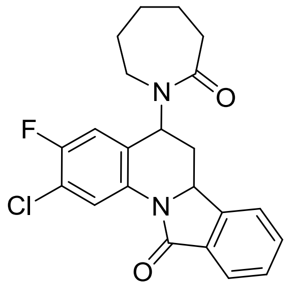

Yield: 42%. White solid. Mp: >260°C. ^{1}H NMR ($CDCl_3$): δ 1.21-1.34(m, 1H), 1.56-1.75(m, 4H), 1.77-1.95(m, 2H), 2.69(s, 3H), 2.84-2.98(m, 1H), 3.05-3.17(m, 1H), 4.88(d, *J*= 12.3Hz, 1H), 6.22-6.30(m, 1H), 6.91(d, *J*= 9.6Hz, 1H), 7.54(dd, *J*= 7.2, 12.0Hz, 2H), 7.65(t, *J*= 7.5Hz, 1H), 7.93(d, *J*= 7.2Hz, 1H), 8.65(d, *J*= 6.9Hz, 1H). ^{13}C NMR ($CDCl_3$): δ 23.6, 29.7, 30.0, 32.0, 37.5, 45.2, 51.1, 58.4, 114.6, 114.9, 120.8, 121.0, 122.1, 122.7, 124.7, 125.8, 129.3, 132.1, 132.8, 133.9, 143.7, 153.3, 156.6, 165.8, 177.0. FT-IR ν_{max} (NaCl): 3047, 2929, 2851, 1700, 1639, 1489, 1409, 1380, 1188, 1078, 914, 730 cm^{-1}. HRMS (amu): calcd for $C_{22}H_{20}ClFN_2O_2$ $[M+Na]^+$: 421.1090; found $[M+Na]^+$: 421.1471.

7-(2-Oxoazepan-1-yl)-8,8a-dihydrobenzo[*h*]isoindolo[2,1-*a*]quinolin-13(7*H*)-one (**2g**): Yield: 34%. White solid. Mp: 260-262°C. ^{1}H NMR ($CDCl_3$): δ 1.21-1.39(m, 2H), 1.50-1.98(m, 5H), 2.59-2.80(m, 2H), 2.80-3.00(m, 1H), 2.98-3.22(m, 2H), 5.08(d, *J*=11.7Hz, 1H), 6.37-6.52(m, 1H), 7.29(d, *J*= 7.8Hz, 2H), 7.50-7.65(m, 3H), 7.65-7.69(m, 1H), 7.75(d, *J*= 8.4Hz, 1H), 7.83-7.88(m, 1H), 8.05(d, *J*= 6.3Hz, 2H). ^{13}C NMR ($CDCl_3$): δ 14.4, 23.8, 29.8, 30.1, 36.0, 45.2, 52.0, 60.2, 122.1, 124.7, 125.1, 125.2, 125.8, 126.6, 126.7, 127.1, 127.6, 128.0, 129.0, 132.2, 132.4, 133.9, 134.3, 146.6, 165.9, 176.9. FT-IR ν_{max} (NaCl): 3047, 2929, 2851, 1701, 1637, 1470, 1397, 1196, 1127, 1033, 976, 755, 730 cm^{-1}. HRMS (amu): calcd for $C_{26}H_{24}N_2O_2$ $[M+Na]^+$: 419.1730; found $[M+Na]^+$: 419.1725.

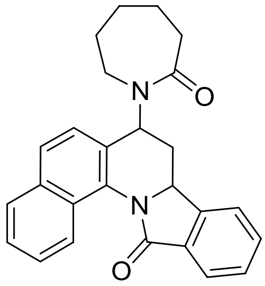

7-(2-Oxoazepan-1-yl)-8,8a-dihydrobenzo[*f*]isoindolo[2,1-*a*]quinolin-13(7*H*)-one (**2h**): Yield: 32%. White solid. Mp: 238-240°C. ^{1}H NMR ($CDCl_3$): δ 0.60-0.80(m, 1H), 0.93-1.12(m, 1H), 1.44(s, 2H), 1.50-1.76(m, 3H), 2.50-2.78(m, 3H), 2.78-3.91(m, 1H), 3.14(t, *J*= 9.3Hz, 1H), 4.79(d, *J*= 12.6Hz, 1H), 6.72(t, *J*= 9.0Hz, 1H), 7.40-7.59(m, 4H), 7.60-7.65(m, 1H), 7.78-7.85(m, 3H), 7.98(d, *J*= 7.2Hz, 1H), 8.68(d, *J*= 9.0Hz, 1H). ^{13}C NMR ($CDCl_3$): δ 24.0, 28.7, 30.6, 34.8, 38.4, 45.4, 49.8, 59.8, 120.7(2C), 123.7, 125.1, 125.3, 126.4, 128.0, 129.8, 130.0, 130.5, 132.7, 133.1, 133.4, 134.0, 138.3, 146.1, 167.7, 178.3. FT-IR ν_{max} (NaCl): 3056, 2925, 2855, 1696, 1631, 1511, 1470, 1403, 1348, 1298, 1263, 1200, 1172, 1029, 816, 751, 726 cm^{-1}. HRMS (amu): calcd for $C_{26}H_{24}N_2O_2$ $[M+Na]^+$: 419.1730; found $[M+Na]^+$: 419.1719.

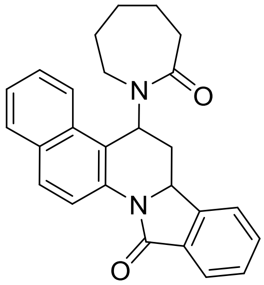

(E)-2-(2-Fluorophenyl)-3-(2-(2-oxopyrrolidin-1-yl)vinyl)isoindolin-1-one (**3a**): Yield: 39%.

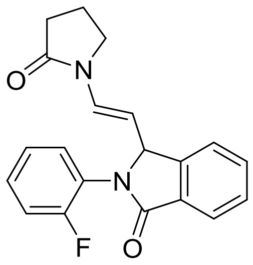

Colorless solid, mp 260-262°C. ^{1}H NMR ($CDCl_3$): δ 1.98-2.10(m, 2H), 2.41-2.48(m, 2H), 3.24-3.44(m,2H), 4.62(dd, J= 9.6, 14.1Hz, 1H), 5.55(d, J= 9.6Hz, 1H), 7.12-7.33(m, 4H), 7.40-7.47(m, 1H), 7.57(dt, J= 7.2, 26.4Hz, 2H), 7.94(d, J= 7.2Hz, 1H). ^{13}C NMR ($CDCl_3$): δ 17.5, 31.1, 45.2, 64.2, 107.5, 116.6, 116.9, 123.4, 124.4, 124.6, 125.0, 125.2, 128.9 (2C), 129.9, 131.7, 132.4, 145.8, 156.6, 159.9, 167.3, 173.3. FT-IR ν_{max} (NaCl): 3068, 2937, 1701, 1655, 1503, 1407, 1375, 1269, 758 cm^{-1}. HRMS (amu): calcd for $C_{20}H_{17}FN_2O_2$ [M+Na]$^+$: 359.1166; found [M+Na]$^+$: 359.1149.

(E)-2-(2-Chlorophenyl)-3-(2-(2-oxopyrrolidin-1-yl)vinyl)isoindolin-1-one (**3b**): Yield: 51%.

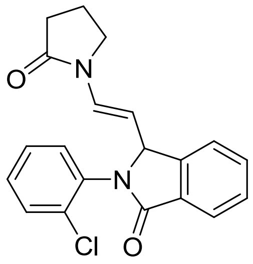

Colorless solid, mp 97-101°C. ^{1}H NMR ($CDCl_3$): δ 2.04-2.12(m, 2H), 2.42-2.50(m, 2H), 3.36-3.47(m,2H), 4.61-4.80(m, 1H), 5.50(bs, 1H), 7.17(d, J=14.3Hz, 1H), 7.27-7.35(m, 2H), 7.43(d, J=7.4Hz, 1H), 7.50-7.60 (m, 2H), 7.63(t, J=7.4Hz, 1H), 7.96(d, J=7.4Hz, 1H). ^{13}C NMR ($CDCl_3$): δ 20.7, 34.3, 48.4, 67.7, 110.5, 126.6, 127.7, 130.9, 132.0, 132.1, 132.7, 133.9, 134.4, 134.8, 135.6, 137.0, 138.1, 149.0, 170.6, 176.6. FT-IR ν_{max} (NaCl): 3068, 2962, 2242, 1704, 1655, 1482, 1407, 1375, 1265, 758 cm^{-1}. HRMS (amu): calcd for $C_{20}H_{17}ClN_2O_2$ [M+Na]$^+$: 375.0876; found [M+Na]$^+$: 375.0856.

(E)-2-(2-Iodophenyl)-3-(2-(2-oxopyrrolidin-1-yl)vinyl)isoindolin-1-one (**3c**). Yield: 57%.

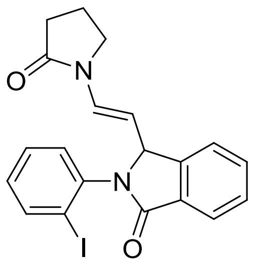

Colorless solid, mp: 118-120°C. ^{1}H NMR ($CDCl_3$): δ 1.72-1.75 (m, 1H), 2.01-2.13 (m, 2H), 2.40-2.51 (m, 2H), 3.34-3.47 (m, 2H), 4.85 (m, 1H), 5.38

(m, 1H), 7.06-7.25 (m, 2H), 7.20 (d, *J*= 7.5Hz, 1H), 7.33-7.46 (m, 2H), 7.48-7.58 (m, 1H), 7.62 (t, *J*= 7.5Hz, 1H), 7.93 (d, *J*= 7.5Hz, 1H). ^{13}C NMR ($CDCl_3$): δ 17.4, 31.1, 45.2, 63.5, 66.6, 99.3, 102.1, 107.3, 123.5, 124.5, 128.4, 129.0, 130.2, 131.8, 132.1, 132.4, 140.1, 145.5, 167.5, 173.5. FT-IR ν_{max} (NaCl): 3047, 2974, 1700, 1655, 1470, 1407, 1372, 1270, 1143, 759, 690 cm^{-1}. HRMS (amu): calcd for $C_{20}H_{17}IN_2O_2$ $[M+Na]^+$: 467.0227; found $[M+Na]^+$: 467.0219.

(*E*)-2-(2,5-Dibromophenyl)-3-(2-(2-oxopyrrolidin-1-yl)vinyl)isoindolin-1-one (**3d**). Yield: 45%.

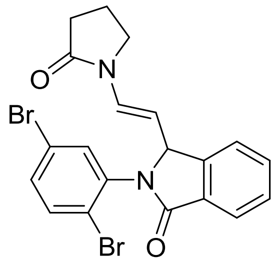

Colorless solid, mp: 248-250°C. ^{1}H NMR ($CDCl_3$): δ 2.02-2.18 (m, 3H), 2.49 (t, *J*= 8.1Hz, 2H), 4.63-4.85 (m, 1H), 5.35-5.57 (m, 1H), 7.18 (d, *J*= 14.1Hz, 1H). 7.33-7.45 (m, 2H), 7.50-7.57 (m, 2H), 7.63 (t, *J*= 7.5Hz, 1H), 7.94 (d, *J*= 7.5Hz, 1H). ^{13}C NMR ($CDCl_3$): δ 17.5, 31.1, 45.3, 60.5, 106.8, 121.4, 123.5, 124.6, 129.0 (2C), 131.2, 132.7, 133.0, 134.8, 137.8, 145.6, 167.0, 173.6. FT-IR ν_{max} (NaCl): 3043, 2925, 1702, 1655, 1467, 1404, 1269, 1030, 751, 686 cm^{-1}. HRMS (amu): calcd for $C_{20}H_{16}BrN_2O_2$ $[M+Na]^+$: 496.9471; found $[M+Na]^+$: 496.9452.

(*E*)-2-(2-Nitrophenyl)-3-(2-(2-oxopyrrolidin-1-yl)vinyl)isoindolin-1-one (**3e**): Yield: 66%. Pale

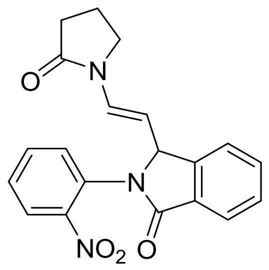

yellow solid, mp: 112-115°C. ^{1}H NMR (DMSO-d_6): δ 1.61 (m, 1H), 2.00-2.13 (m, 2H), 2.47 (t, *J*= 9.0 Hz, 2H), 3.30-3.49 (m, 2H), 4.83 (dd, *J*= 9.6, 14.2Hz, 1H), 5.55 (d, *J*= 9.6Hz, 1H), 7.30-7.37 (m, 1H), 7.41-7.49 (m, 2H), 7.53 (t, *J*= 7.5Hz, 1H), 7.60-7.70 (m, 2H), 7.90 (d, *J*= 7.5Hz, 1H), 8.10 (dd, *J*= 1.2, 8.1Hz, 1H). ^{13}C NMR (DMSO-d_6): δ 16.6, 30.3, 44.4, 63.0, 105.8, 123.2, 123.5, 124.9 (2C), 127.4, 128.4, 128.7 (2C), 128.8, 129.8, 133.7, 145.8, 165.9, 172.9. FT-IR

ν_{max} (NaCl): 3047, 2921, 1702, 1655, 1601, 1529, 1405, 1373, 1262, 1139, 739 cm^{-1}. HRMS (amu): calcd for $C_{20}H_{17}N_3O_4$ $[M+Na]^+$: 386.1106; found $[M+Na]^+$: 386.1101.

(*E*)-2-(2-Ethylphenyl)-3-(2-(2-oxopyrrolidin-1-yl)vinyl)isoindolin-1-one (**3f**): Yield: 63%. Colourless solid, mp 155-157°C. ^{1}H NMR ($CDCl_3$): δ 1.22(t, J=7.6Hz, 3H), 2.05-2.10(m, 2H), 2.43-2.56(m, 4H), 3.28-3.42(m, 2H), 4.63-4.72(m, 1H), 4.67-5.44(AB quartet, 1H), 7.08-7.38(m, 4H), 7.43(d, J=7.3Hz, 1H), 7.54(dt, J=7.3, 1.2Hz, 1H), 7.60(dt, J=7.4, 1.2Hz, 1H), 7.94(d, J= J=7.3Hz, 1H). ^{13}C NMR ($CDCl_3$): δ 14.2, 17.2, 23.9, 30.8, 44.9, 123.1, 124.0, 126.4, 128.3, 128.6, 128.9, 131.9, 134.9, 142.6, 145.3, 173.2. FT-IR ν_{max} (NaCl): 3053, 2962, 2929, 1689, 1655, 1492, 1413, 1284, 1151, 760 cm^{-1}. HRMS (amu): calcd for $C_{22}H_{22}N_2O_2$ $[M+Na]^+$: 369.1579; found $[M+Na]^+$: 369.1559.

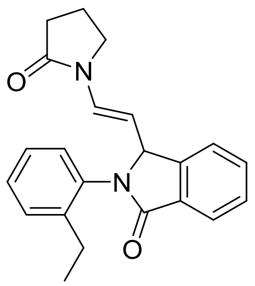

(*E*)-2-(2-*tert*-Butylphenyl)-3-(2-(2-oxopyrrolidin-1-yl)vinyl)isoindolin-1-one (**3g**): Yield: 56%. Colorless solid, mp: 246-248°C. ^{1}H NMR ($CDCl_3$): δ 1.36(s, 9H), 2.09(t, J=7.4 Hz, 2H), 2.47(t, J=7.4 Hz, 2H), 3.35-3.49 (m, 2H), 4.69(dd, J=14.2, 9.5 Hz, 1H), 5.31 (d, J=9.5 Hz, 1H), 6.91 (dd, J=7.7, 1.3 Hz, 1H), 7.05 (d, J=14.2Hz, 1H), 7.20 (t, J=7.7 Hz, 1H), 7.33 (t, J=7.7Hz, 1H), 7.43, d, J=7.4Hz, 1H), 7.53-7.62(m, 3H), 7.94(d, J=7.5Hz, 1H). ^{13}C NMR ($CDCl_3$): δ 17.2, 30.8, 32.0, 32.5, 35.9, 45.0, 66.0, 108.2, 123.2, 124.1, 126.3, 128.1, 128.6, 128.9, 131.9, 133.6, 134.2, 145.4, 149.6, 168.5, 173.1. FT-IR ν_{max} (NaCl): 3054, 2959, 2917, 1688, 1655, 1489, 1437, 1241, 1145, 758 cm^{-1}. HRMS (amu): calcd for $C_{24}H_{26}N_2O_2$ $[M+Na]^+$: 397.1892; found $[M+Na]^+$: 397.1872.

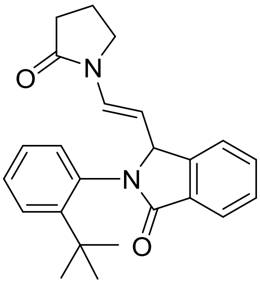

(*E*)-2-(2-Methoxyphenyl)-3-(2-(2-oxopyrrolidin-1-yl)vinyl)isoindolin-1-one (**3h**): Yield: 53%.

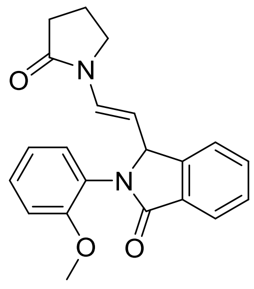

mp: 86-89°C. ^{1}H NMR ($CDCl_3$): δ 1.92-2.02(m, 2H), 2.32-2.40(m, 2H), 3.17-3.38(m, 2H), 3.75(s, 3H), 4.59(dd, *J*= 9.6, 14.1Hz, 1H), 5.56(d, *J*= 9.6Hz, 1H), 6.89-6.96(m, 2H), 7.12(d, *J*= 15.0Hz, 1H), 7.18-7.26(m, 2H), 7.36(d, *J*= 7.5Hz), 7.44(t, *J*= 7.2Hz, 1H), 7.53(t, *J*= 7.2Hz, 1H), 7.85(d, *J*= 7.5Hz, 1H). ^{13}C NMR ($CDCl_3$): δ 17.1, 30.8, 44.9, 55.6, 63.4, 107.7, 112.1, 120.6, 123.0, 123.7, 125.4, 128.0, 128.2, 128.7, 130.0, 131.8(2C), 145.7, 155.3, 167.3, 173.1. FT-IR ν_{max} (NaCl): 3051, 2929, 2839, 1697, 1655, 1503, 1407, 1261, 1143, 747 cm^{-1}. HRMS (amu): calcd for $C_{21}H_{20}N_2O_3$ $[M+Na]^+$: 371.1366; found $[M+Na]^+$: 371.1353.

(*E*)-2-(2-(Trifluoromethoxy)phenyl)-3-(2-(2-oxopyrrolidin-1-yl)vinyl)isoindolin-1-one (**3i**): Yield:

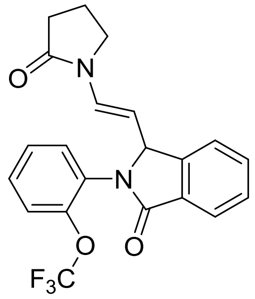

58%. Colorless solid, mp: >260°C. ^{1}H NMR ($CDCl_3$): δ 1.20-1.40 (m, 1H), 2.00-2.13 (m, 2H), 2.47 (t, *J*= 8.4Hz, 2H), 3.23-3.47 (m, 2H), 4.62 (dd, *J*= 9.6, 14.3Hz, 1H), 5.30 (d, *J*= 9.6Hz, 1H), 7.30 (d, *J*=14.4Hz, 1H), 7.30-7.43 (m, 3H), 7.48 (d, *J*= 7.8Hz, 1H), 7.55 (t, *J*= 7.5Hz, 1H), 7.64 (t, *J*= 7.5Hz, 1H), 7.96 (d, *J*= 7.5Hz, 1H). ^{13}C NMR ($CDCl_3$): δ 17.4, 18.1, 31.0, 45.0, 64.2, 107.2, 121.3, 123.4, 124.4, 127.3, 128.7, 128.9(2C), 129.5, 130.5, 131.2, 132.5, 145.6, 167.3, 173.4. FT-IR ν_{max} (NaCl): 2917, 1705, 1655, 1503, 1405, 1366, 1249, 1169, 759 cm^{-1}. HRMS (amu): calcd for $C_{21}H_{17}F_3N_2O_3$ $[M+Na]^+$: 425.1083; found $[M+Na]^+$: 425.1100.

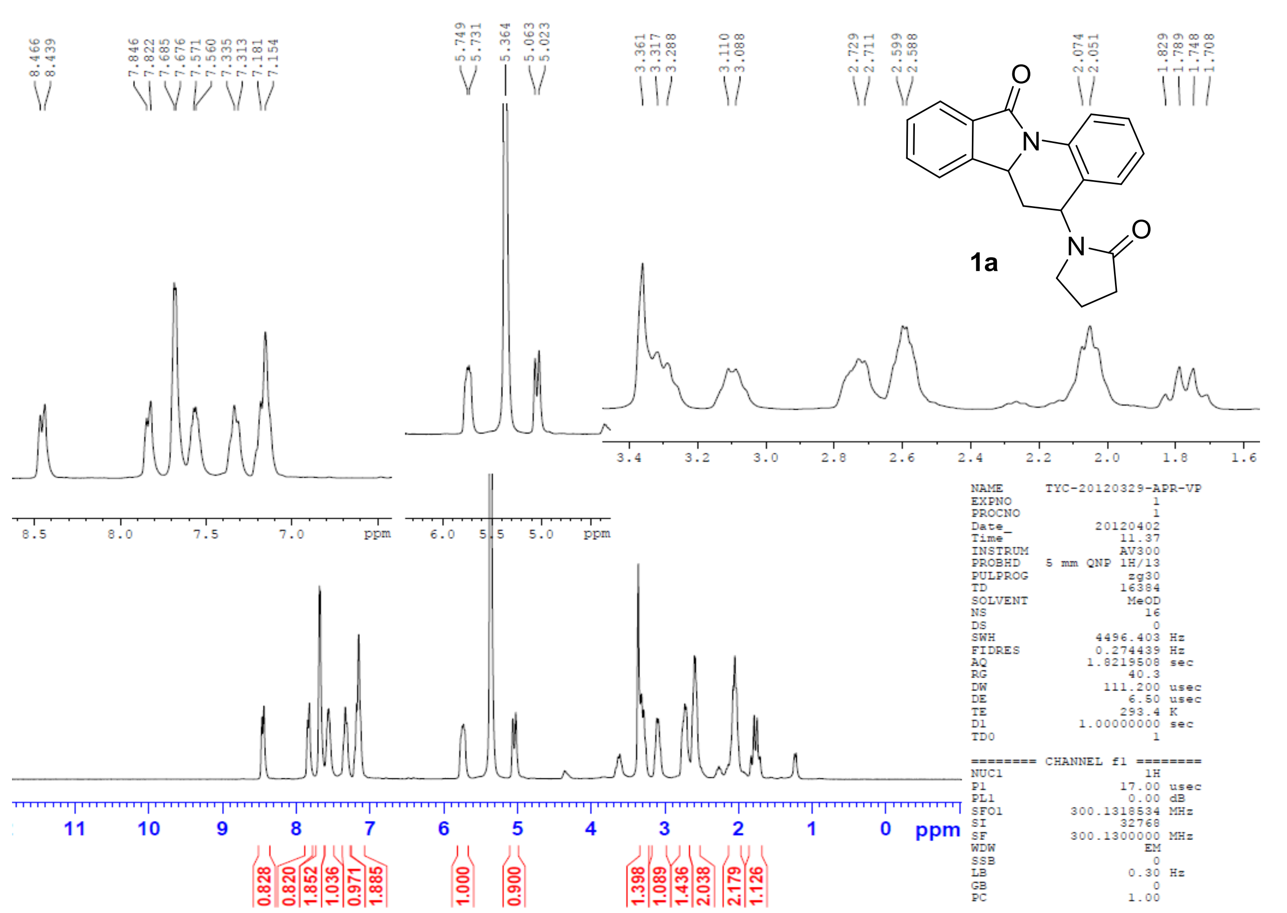
8.466
8.439
7.846
7.822
7.685
7.676
7.571
7.560
7.335
7.313
7.181
7.154
5.749
5.731
5.364
5.063
5.023
3.361
3.317
3.288
3.110
3.088
2.729
2.711
2.599
2.588
2.074
2.051
1.829
1.789
1.748
1.708
1a
NAME TYC-20120329-APR-VP
EXPNO 1
PROCNO 1
Date_ 20120402
Time 11.37
INSTRUM AV300
PROBHD 5 mm QNP 1H/13
PULPROG zg30
TD 16384
SOLVENT MeOD
NS 16
DS 0
SWH 4496.403 Hz
FIDRES 0.274439 Hz
AQ 1.8219508 sec
RG 40.3
DW 111.200 usec
DE 6.50 usec
TE 293.4 K
D1 1.00000000 sec
TD0 1
======== CHANNEL f1 ========
NUC1 1H
P1 17.00 usec
PL1 0.00 dB
SFO1 300.1318534 MHz
SI 32768
SF 300.1300000 MHz
WDW EM
SSB 0
LB 0.30 Hz
GB 0
PC 1.00
0.828
0.820
1.852
1.036
0.971
1.885
1.000
0.900
1.398
1.089
1.436
2.038
2.179
1.126
ppm

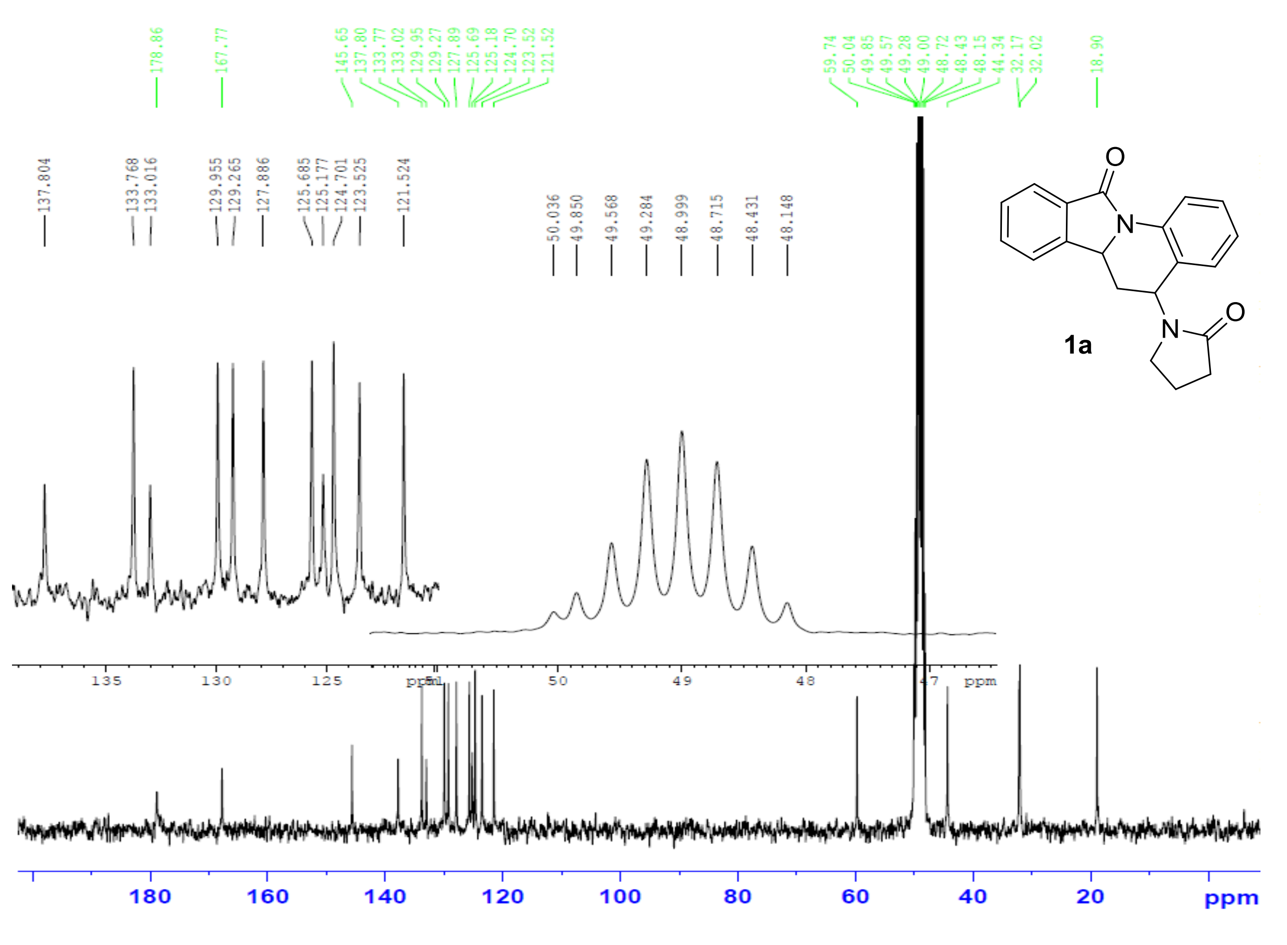
178.86
167.77
145.65
137.80
133.77
133.02
129.95
129.27
127.89
125.69
125.18
124.70
123.52
121.52
59.74
50.04
49.85
49.57
49.28
49.00
48.72
48.43
48.15
44.34
32.17
32.02
18.90
137.804
133.768
133.016
129.955
129.265
127.886
125.685
125.177
124.701
123.525
121.524
50.036
49.850
49.568
49.284
48.999
48.715
48.431
48.148
1a
ppm

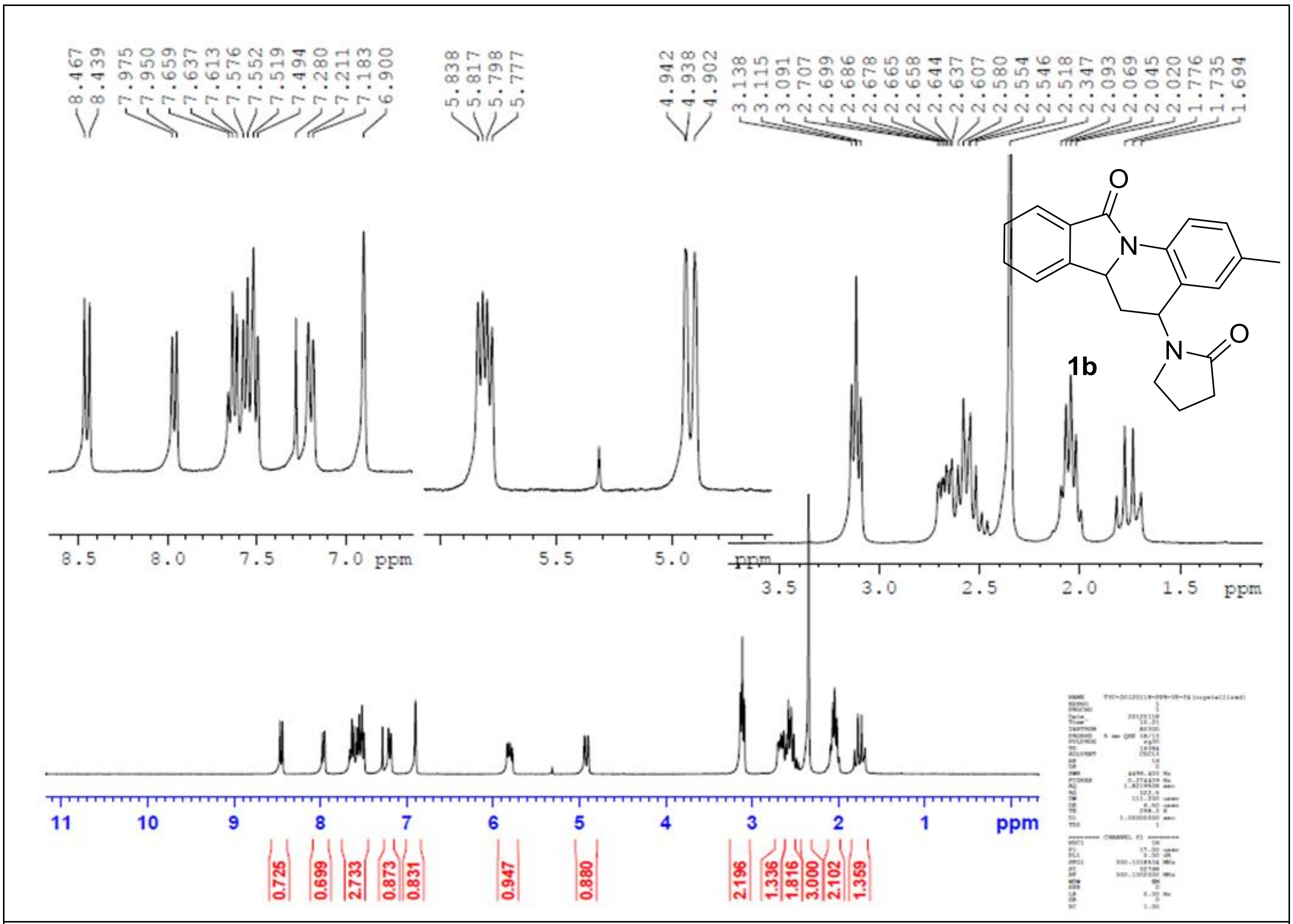
8.467
8.439
7.975
7.950
7.659
7.637
7.613
7.576
7.552
7.519
7.494
7.280
7.211
7.183
6.900
5.838
5.817
5.798
5.777
4.942
4.938
4.902
3.138
3.115
3.091
2.707
2.699
2.686
2.678
2.665
2.658
2.644
2.637
2.607
2.580
2.554
2.546
2.518
2.347
2.093
2.069
2.045
2.020
1.776
1.735
1.694
O
N
N
O
1b
8.5 8.0 7.5 7.0 ppm
5.5 5.0 ppm
3.5 3.0 2.5 2.0 1.5 ppm
11 10 9 8 7 6 5 4 3 2 1 ppm
0.725
0.699
2.733
0.873
0.831
0.947
0.880
2.196
1.336
1.816
3.000
2.102
1.359

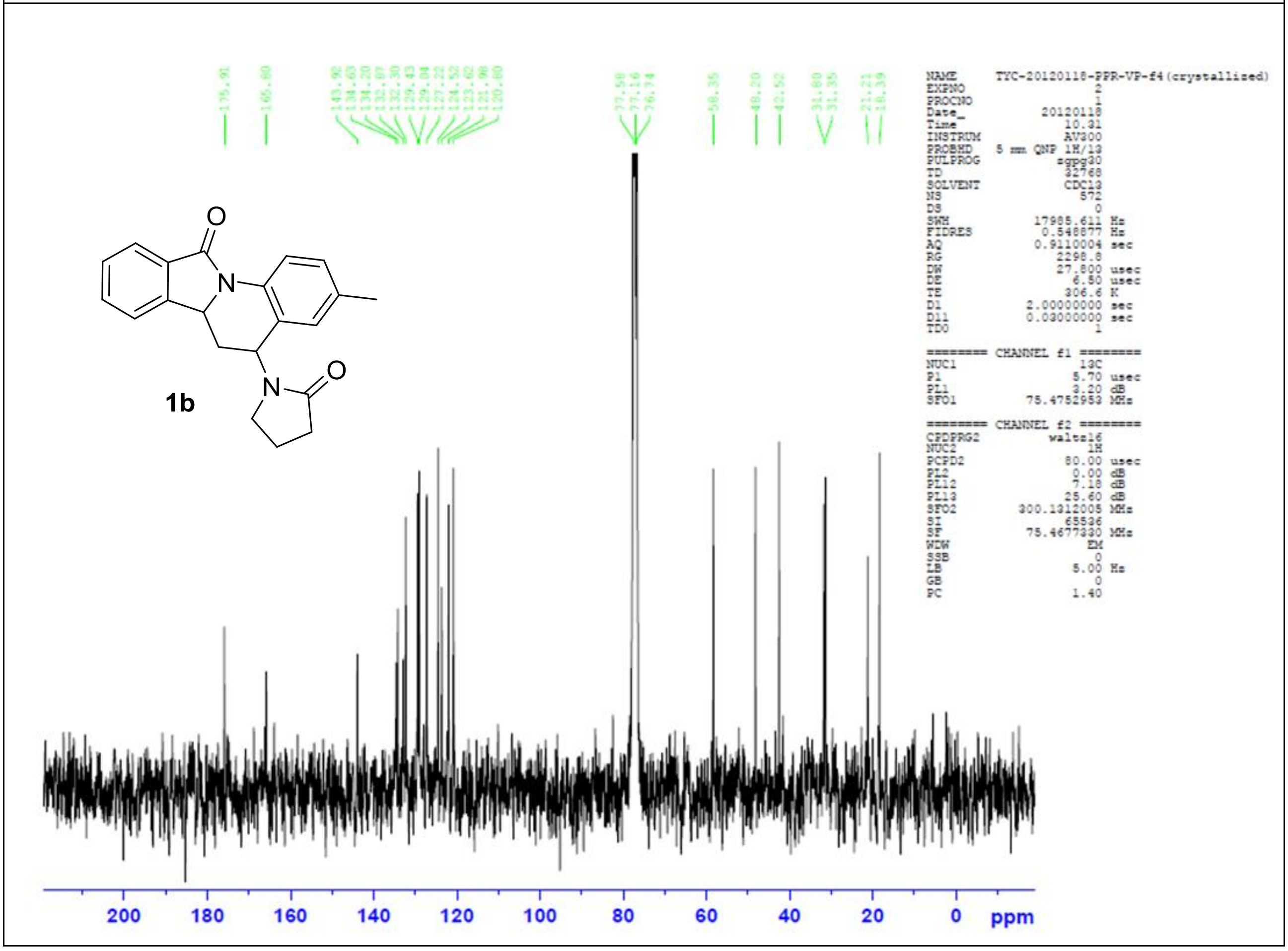
175.91
165.80
143.92
134.63
134.20
132.87
132.30
129.43
129.04
127.22
124.52
123.62
121.96
120.80
77.58
77.16
76.74
58.35
48.20
42.52
31.80
31.35
21.21
18.39
NAME TYC-20120118-PPR-VP-f4(crystallized)
EXPNO 2
PROCNO 1
Date_ 20120118
Time 10.31
INSTRUM AV300
PROBHD 5 mm QNP 1H/13
PULPROG zgpg30
TD 32768
SOLVENT CDCl3
NS 572
DS 0
SWH 17985.611 Hz
FIDRES 0.548877 Hz
AQ 0.9110004 sec
RG 2298.8
DW 27.800 usec
DE 6.50 usec
TE 306.6 K
D1 2.00000000 sec
D11 0.03000000 sec
TD0 1
======== CHANNEL f1 ========
NUC1 13C
P1 5.70 usec
PL1 3.20 dB
SFO1 75.4752953 MHz
======== CHANNEL f2 ========
CPDPRG2 waltz16
NUC2 1H
PCPD2 80.00 usec
PL2 0.00 dB
PL12 7.18 dB
PL13 25.60 dB
SFO2 300.1312005 MHz
SI 65536
SF 75.4677330 MHz
WDW EM
SSB 0
LB 5.00 Hz
GB 0
PC 1.40
O
N
N
O
1b
200 180 160 140 120 100 80 60 40 20 0 ppm

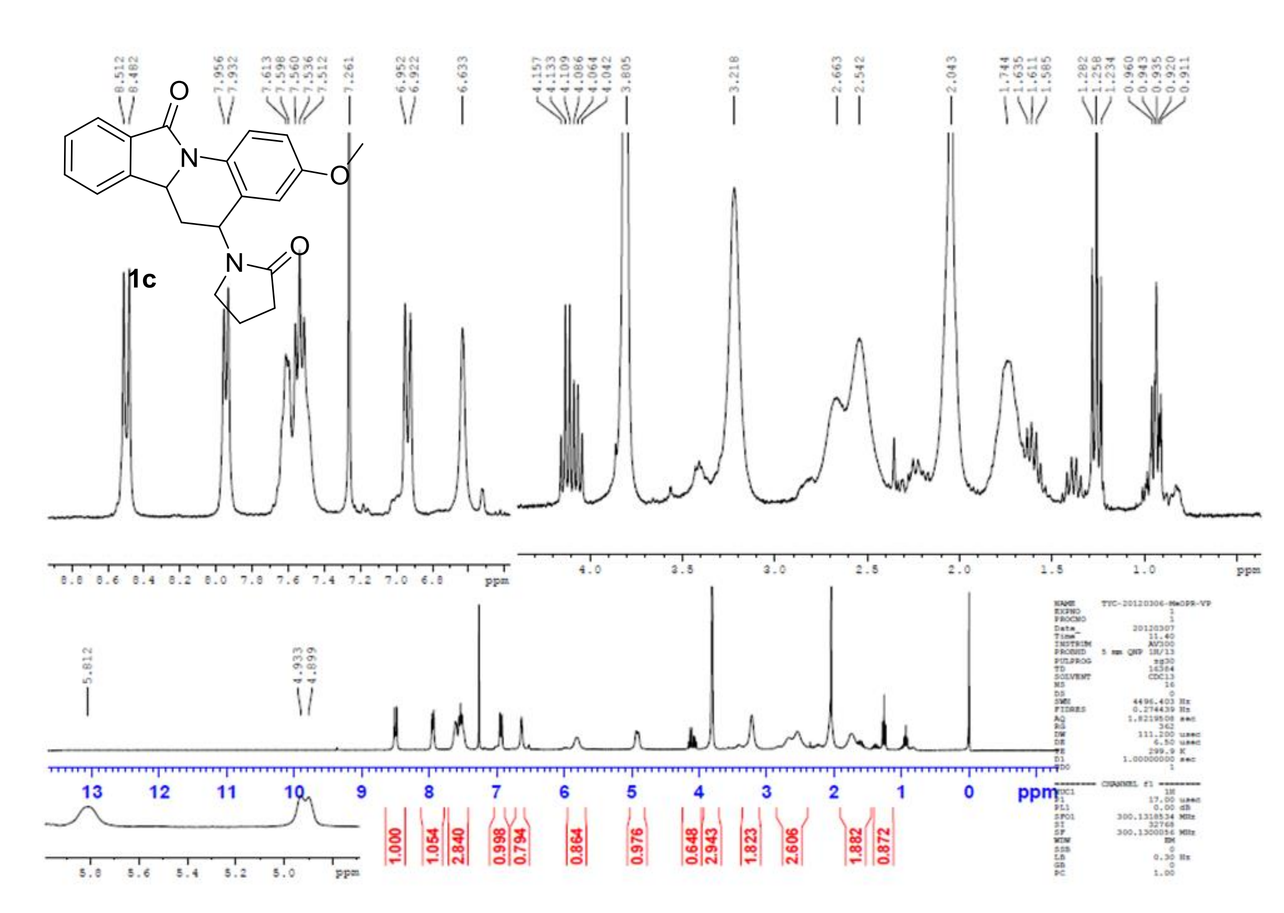
1c
8.512
8.482
7.956
7.932
7.613
7.598
7.560
7.536
7.512
7.261
6.952
6.922
6.633
4.157
4.133
4.109
4.086
4.064
4.042
3.805
3.218
2.663
2.542
2.043
1.744
1.635
1.611
1.585
1.282
1.258
1.234
0.960
0.943
0.935
0.920
0.911
5.812
4.933
4.899
1.000
1.054
2.840
0.998
0.794
0.864
0.976
0.648
2.943
1.823
2.606
1.882
0.872
ppm

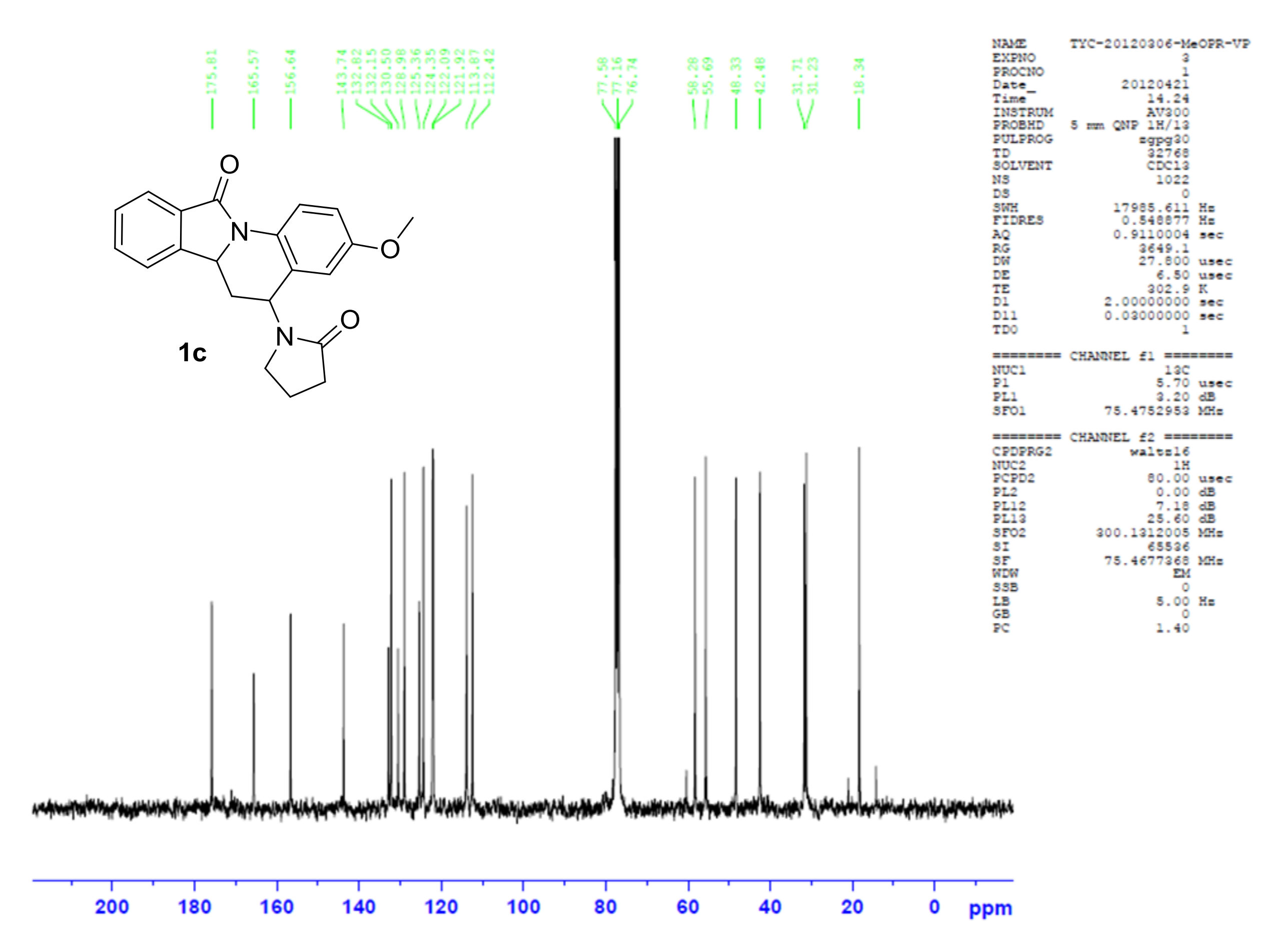
175.81
165.57
156.64
143.74
132.82
132.15
130.50
128.98
125.36
124.35
122.09
121.92
113.87
112.42
77.58
77.16
76.74
58.28
55.69
48.33
42.40
31.71
31.23
18.34
1c
NAME TYC-20120306-MeOPR-VP
EXPNO 3
PROCNO 1
Date_ 20120421
Time 14.24
INSTRUM AV300
PROBHD 5 mm QNP 1H/13
PULPROG zgpg30
TD 32768
SOLVENT CDCl3
NS 1022
DS 0
SWH 17985.611 Hz
FIDRES 0.548877 Hz
AQ 0.9110004 sec
RG 3649.1
DW 27.800 usec
DE 6.50 usec
TE 302.9 K
D1 2.00000000 sec
D11 0.03000000 sec
TD0 1
======== CHANNEL f1 ========
NUC1 13C
P1 5.70 usec
PL1 3.20 dB
SFO1 75.4752953 MHz
======== CHANNEL f2 ========
CPDPRG2 waltz16
NUC2 1H
PCPD2 80.00 usec
PL2 0.00 dB
PL12 7.18 dB
PL13 25.60 dB
SFO2 300.1312005 MHz
SI 65536
SF 75.4677368 MHz
WDW EM
SSB 0
LB 5.00 Hz
GB 0
PC 1.40
200
180
160
140
120
100
80
60
40
20
0
ppm

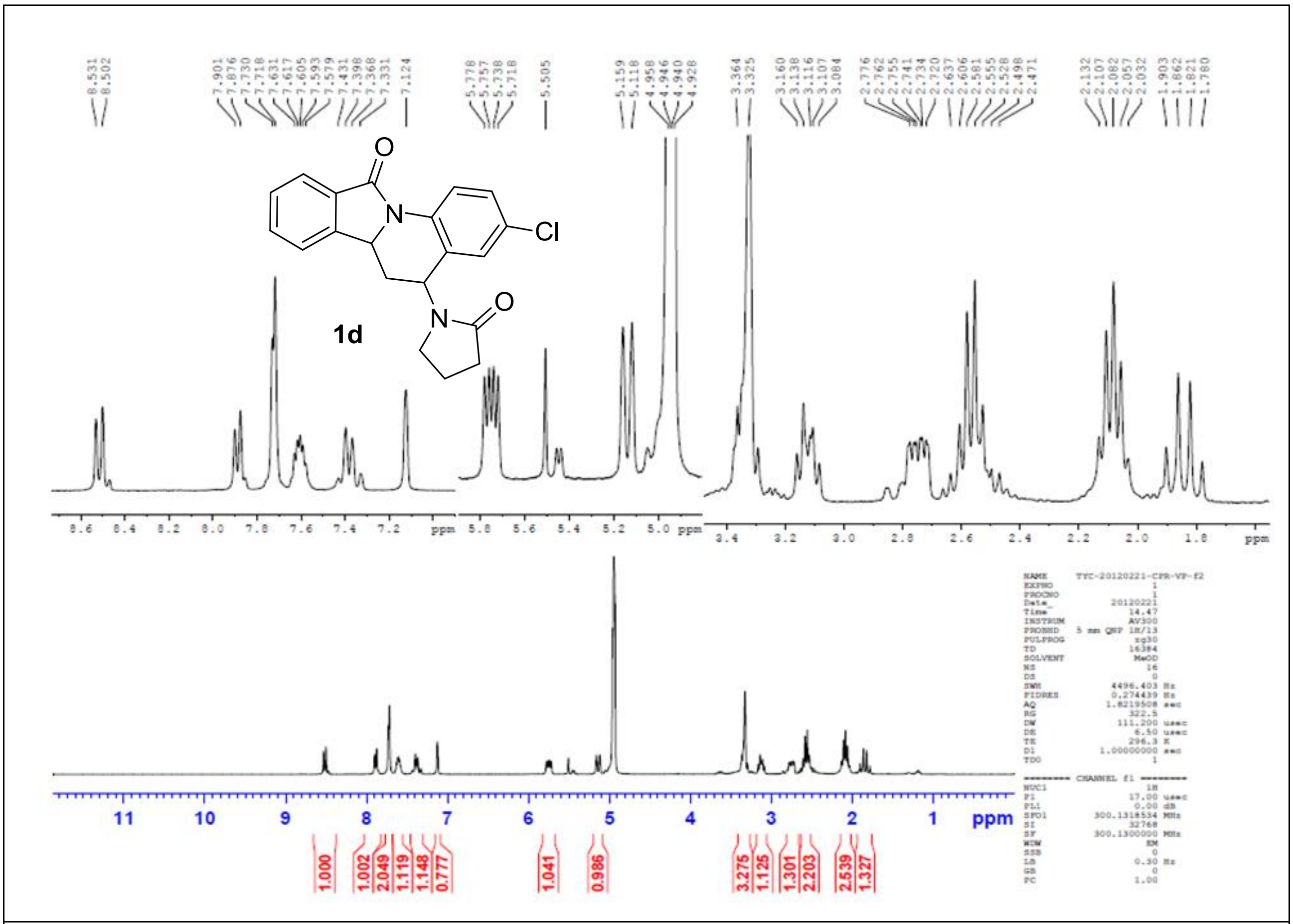
1d
ppm
11
10
9
8
7
6
5
4
3
2
1
ppm
1.000
1.002
2.049
1.119
1.148
0.777
1.041
0.986
3.275
1.125
1.301
2.203
2.539
1.327

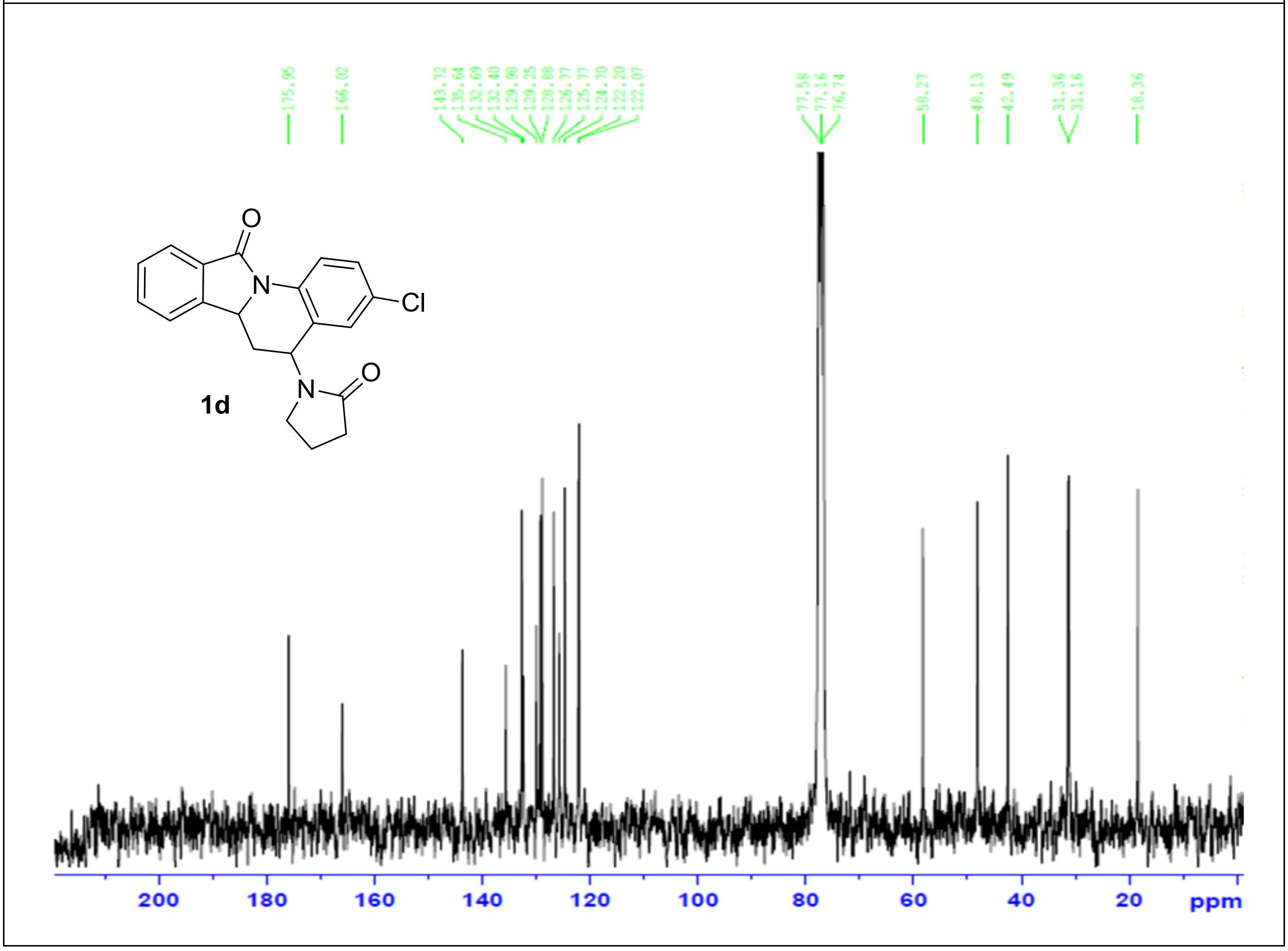
1d
175.96
166.02
143.72
135.64
132.69
132.40
129.98
129.25
128.88
126.77
125.77
124.70
122.20
122.07
77.58
77.16
76.74
58.27
48.13
42.49
31.36
31.16
18.36
200
180
160
140
120
100
80
60
40
20
ppm

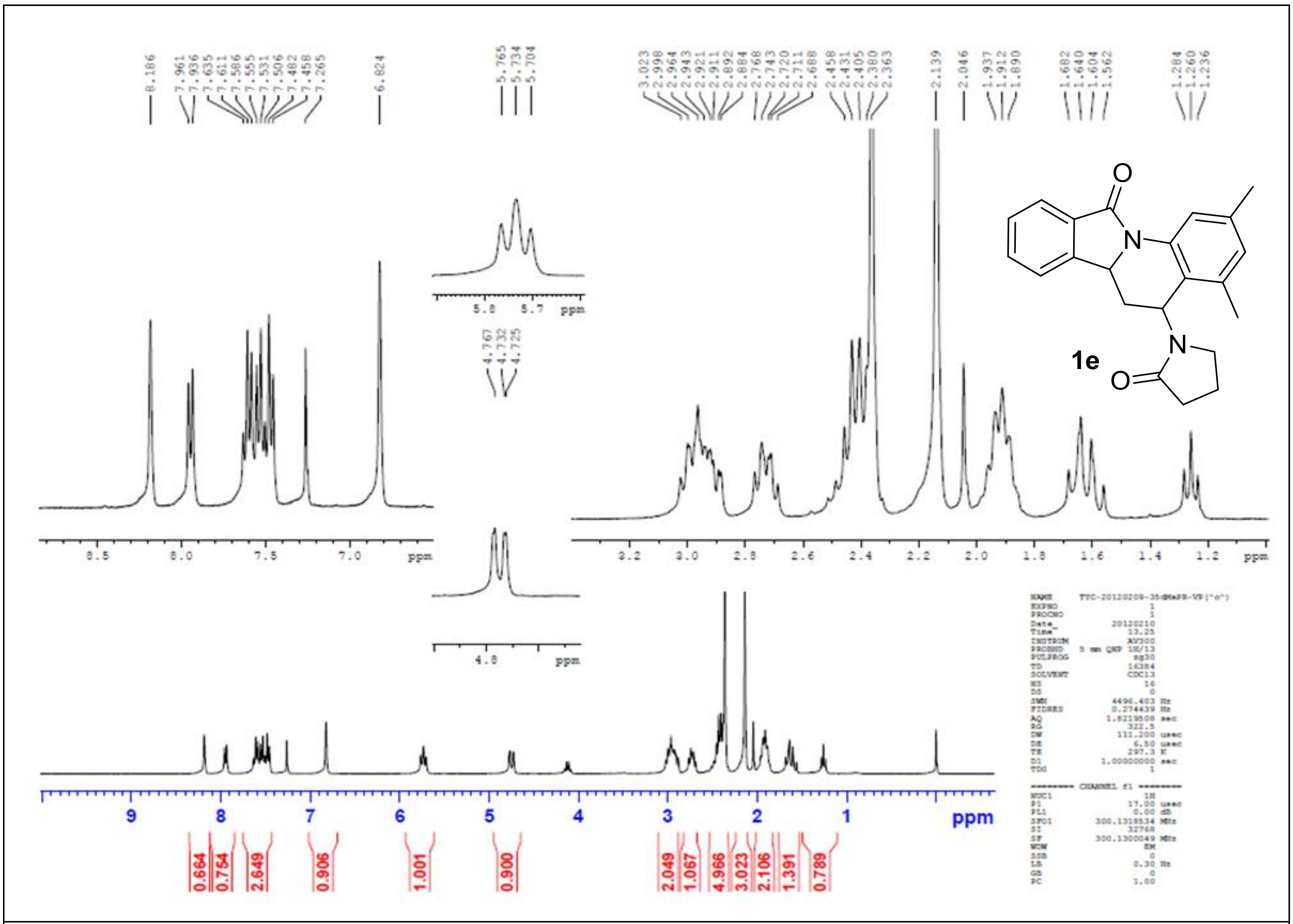

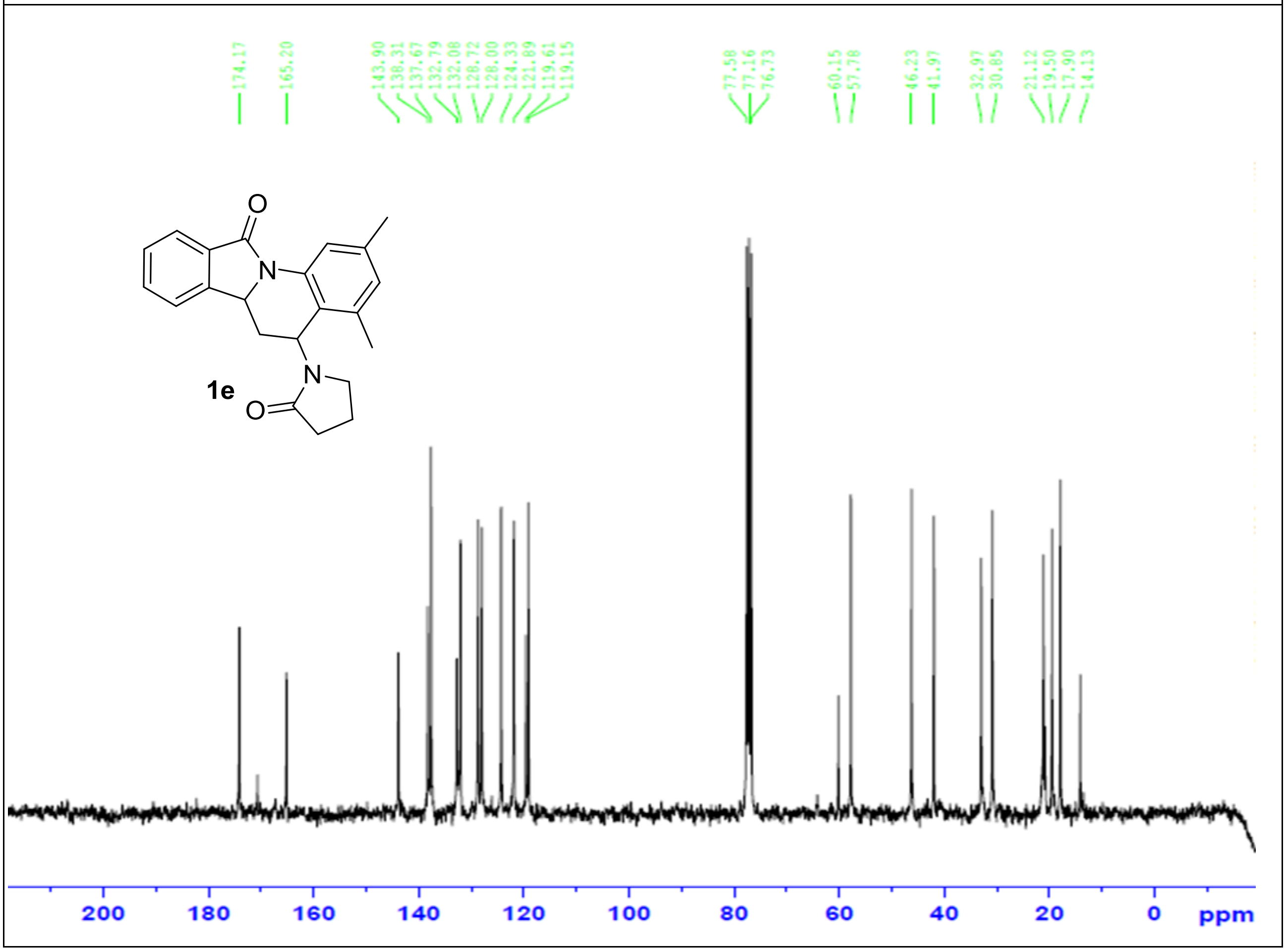

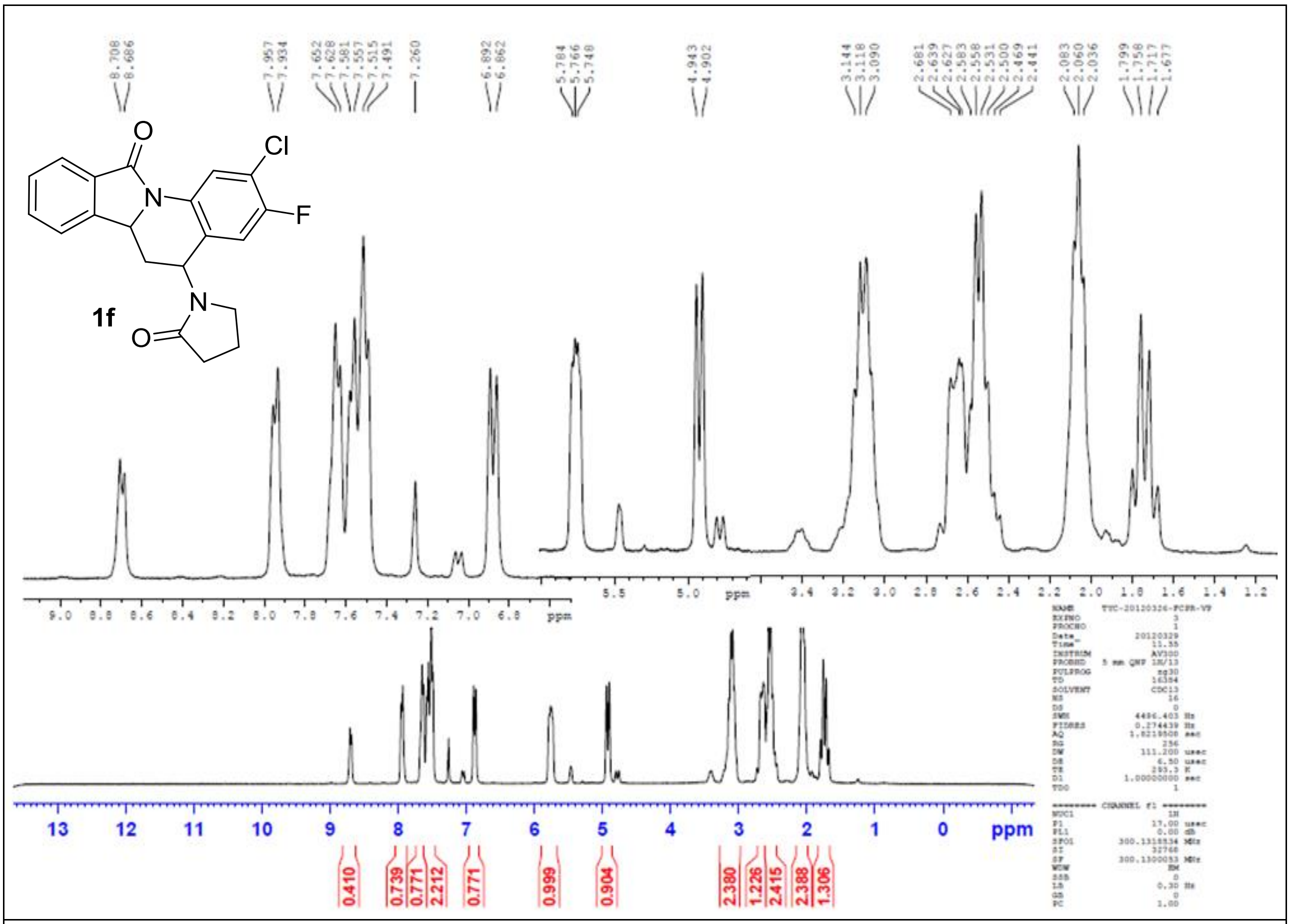
Cl
F
N
O
1f
ppm

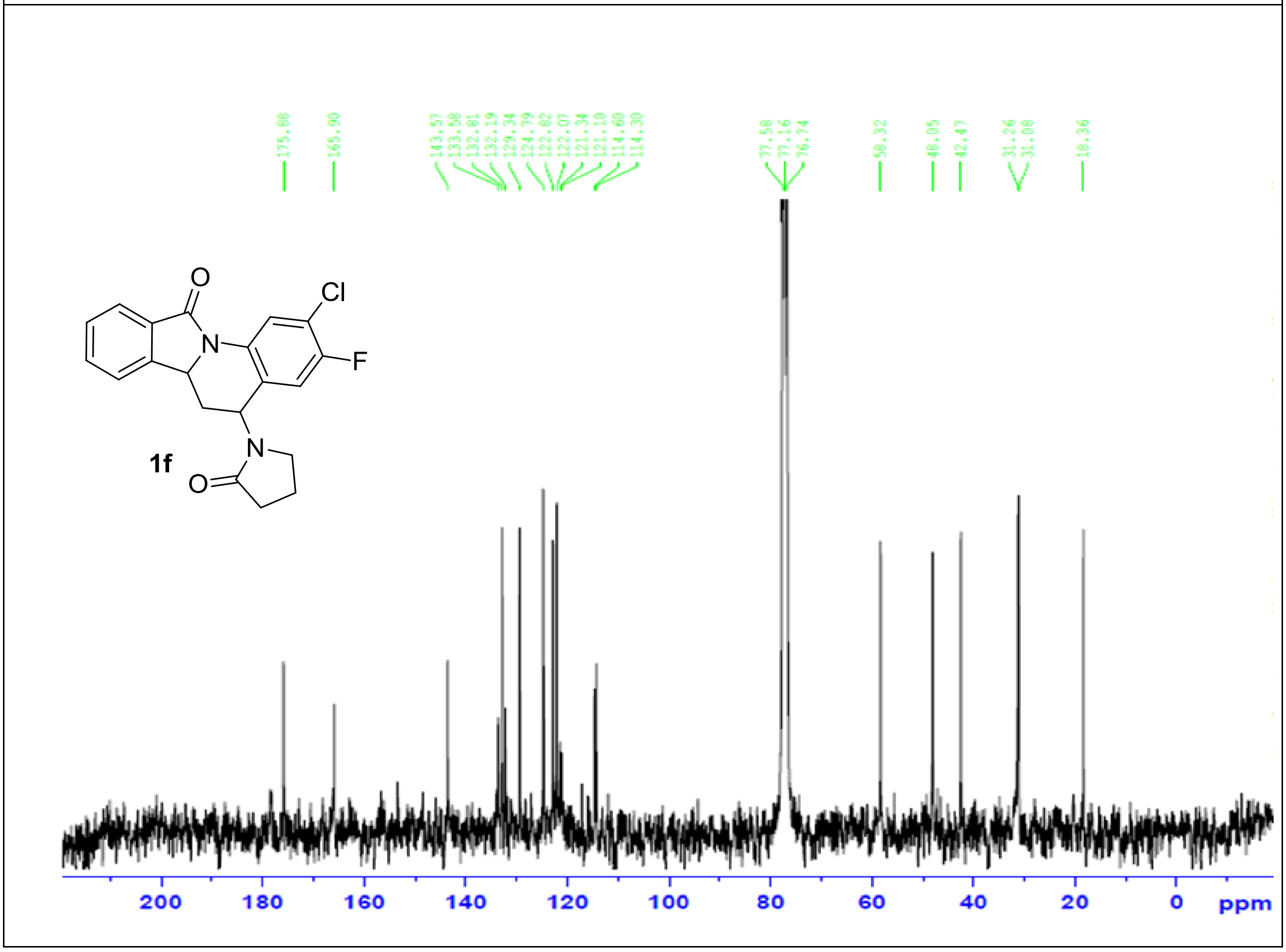
Cl
F
N
O
1f
ppm

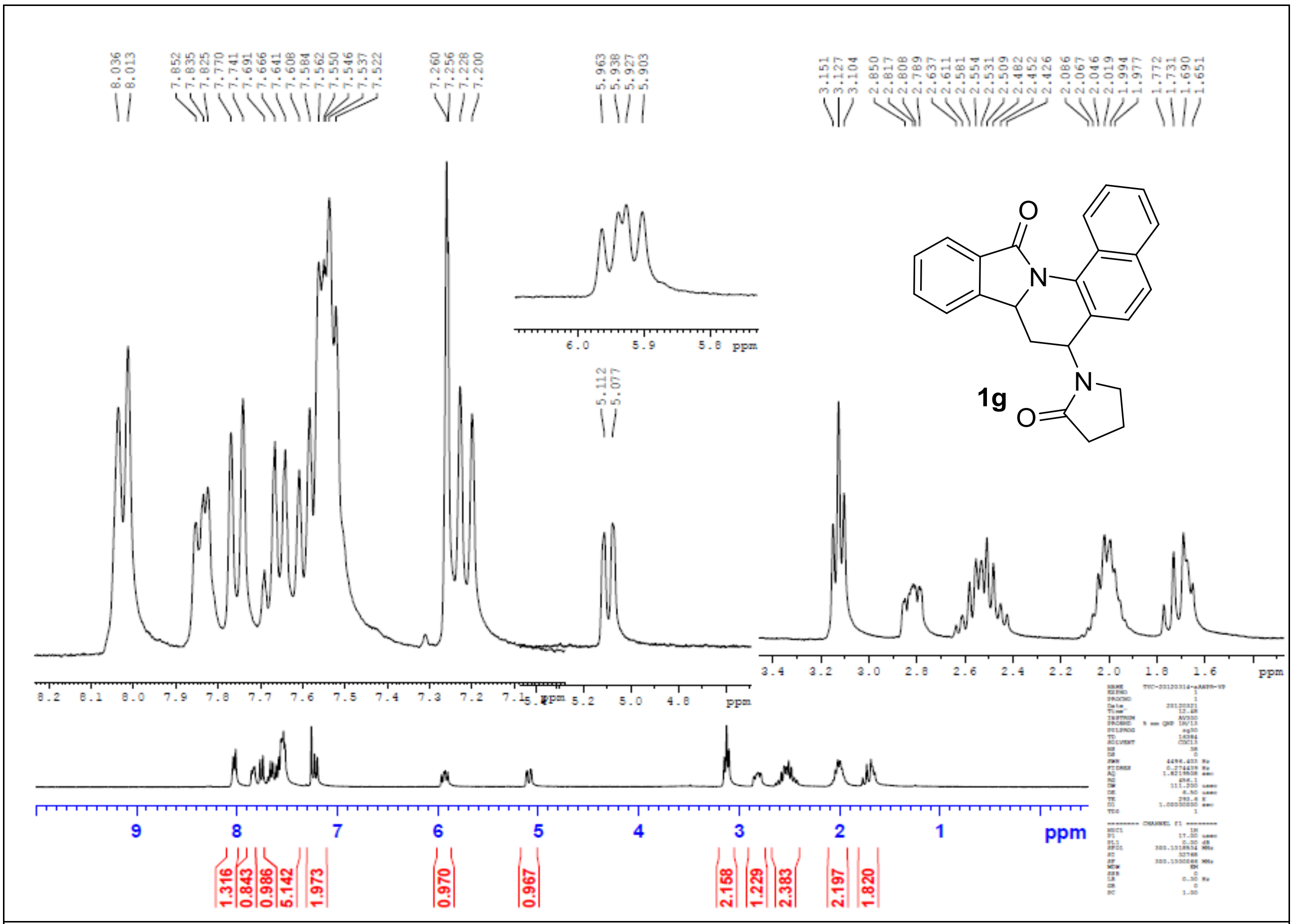

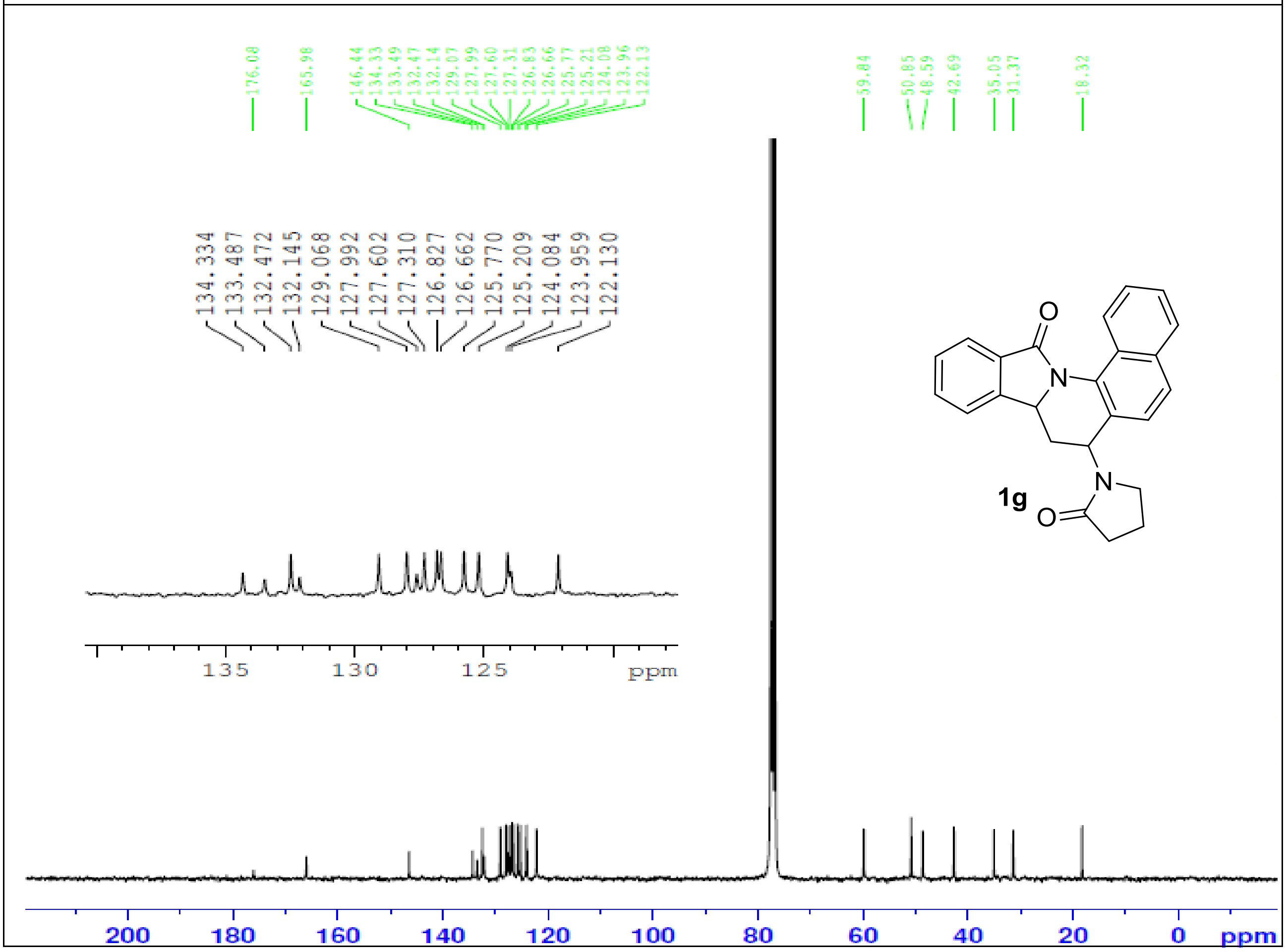

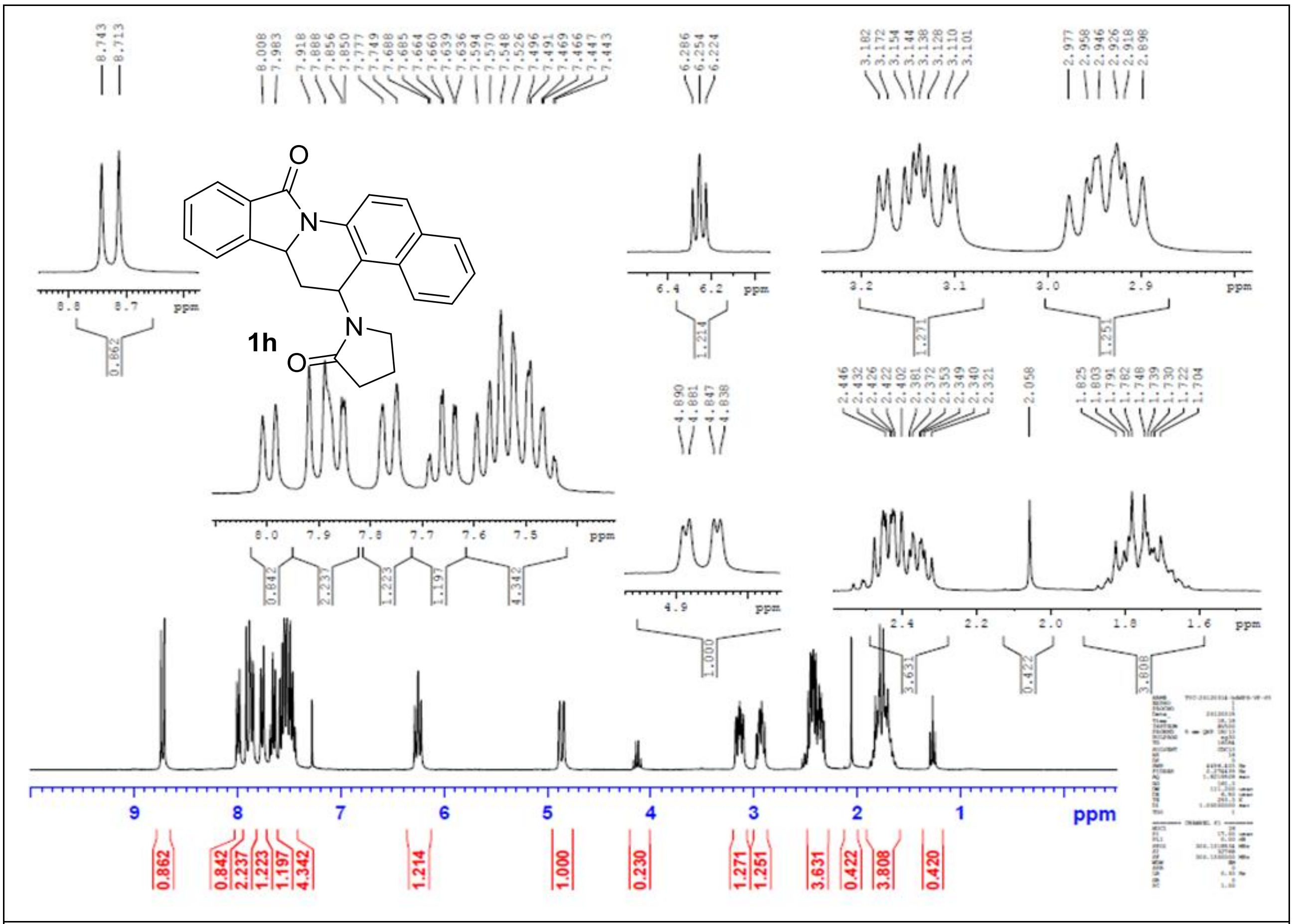

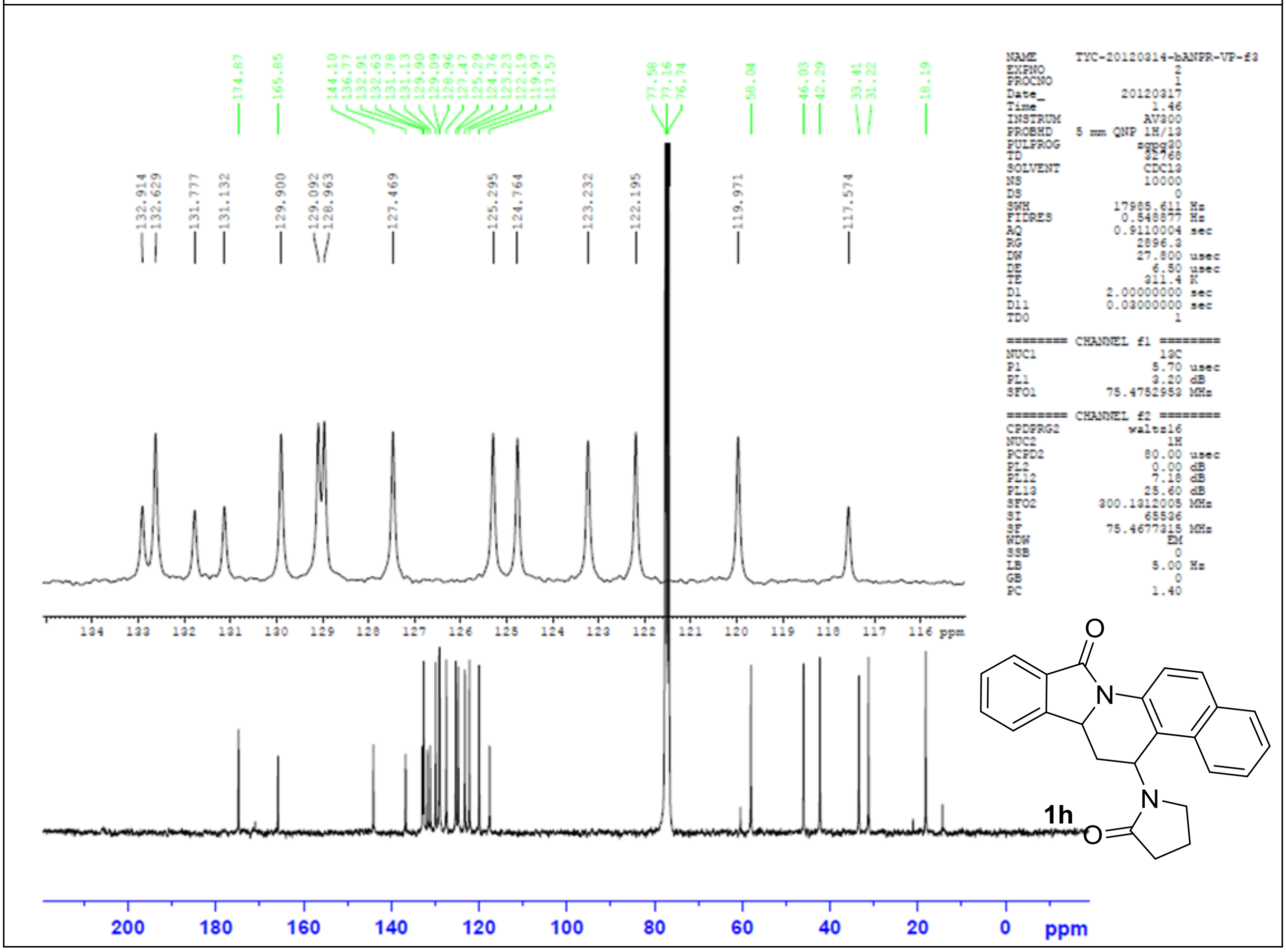

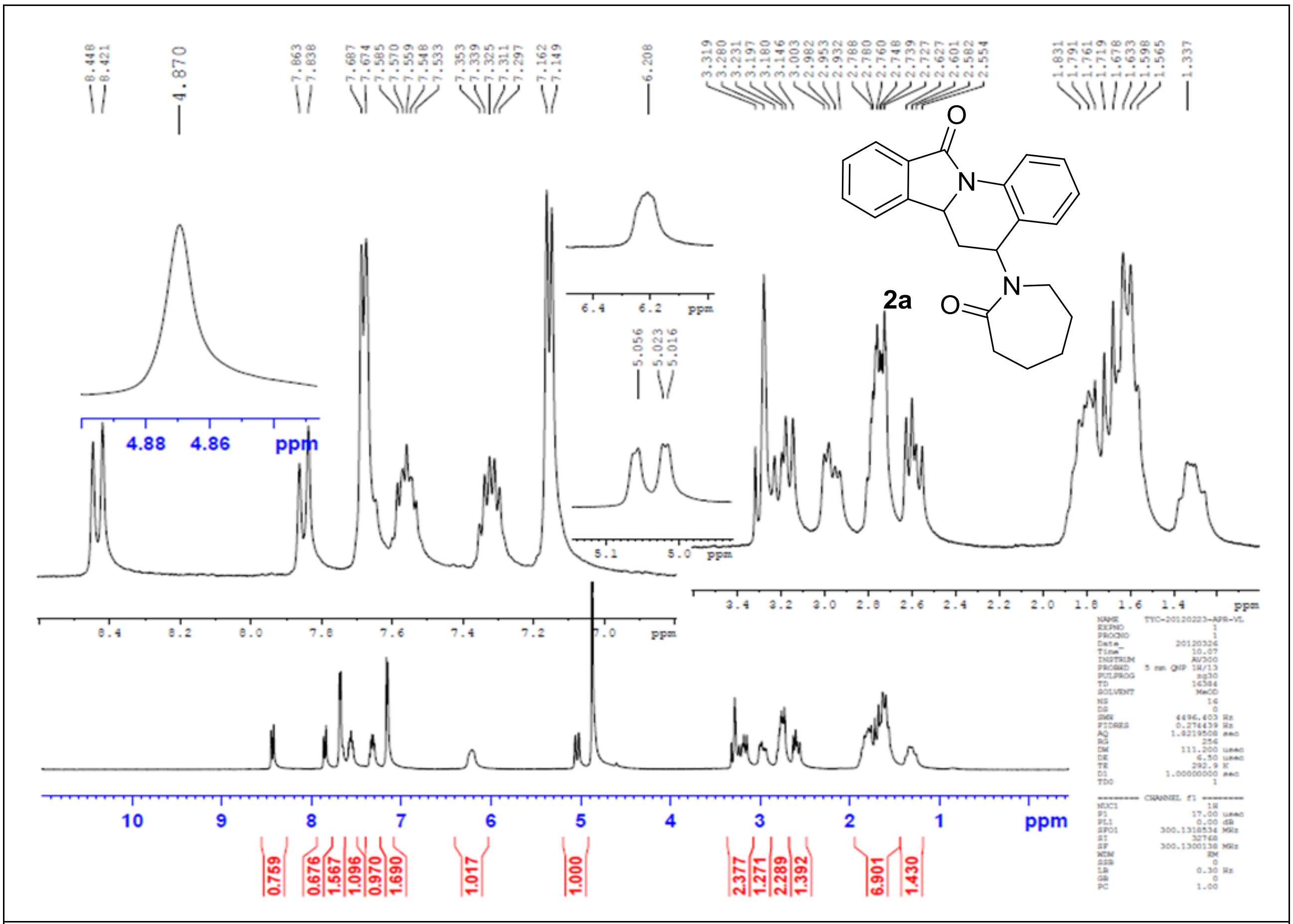
2a

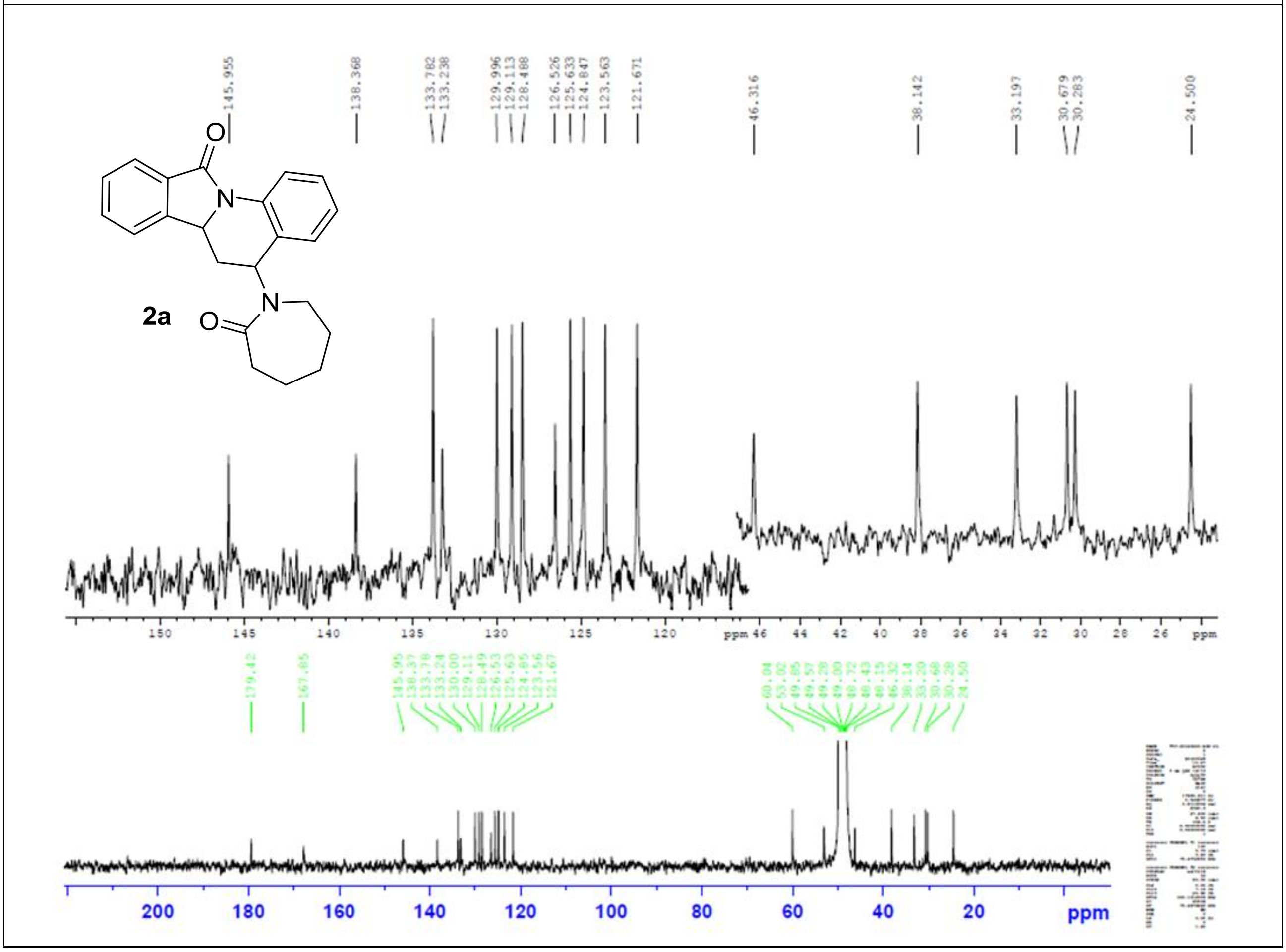
2a

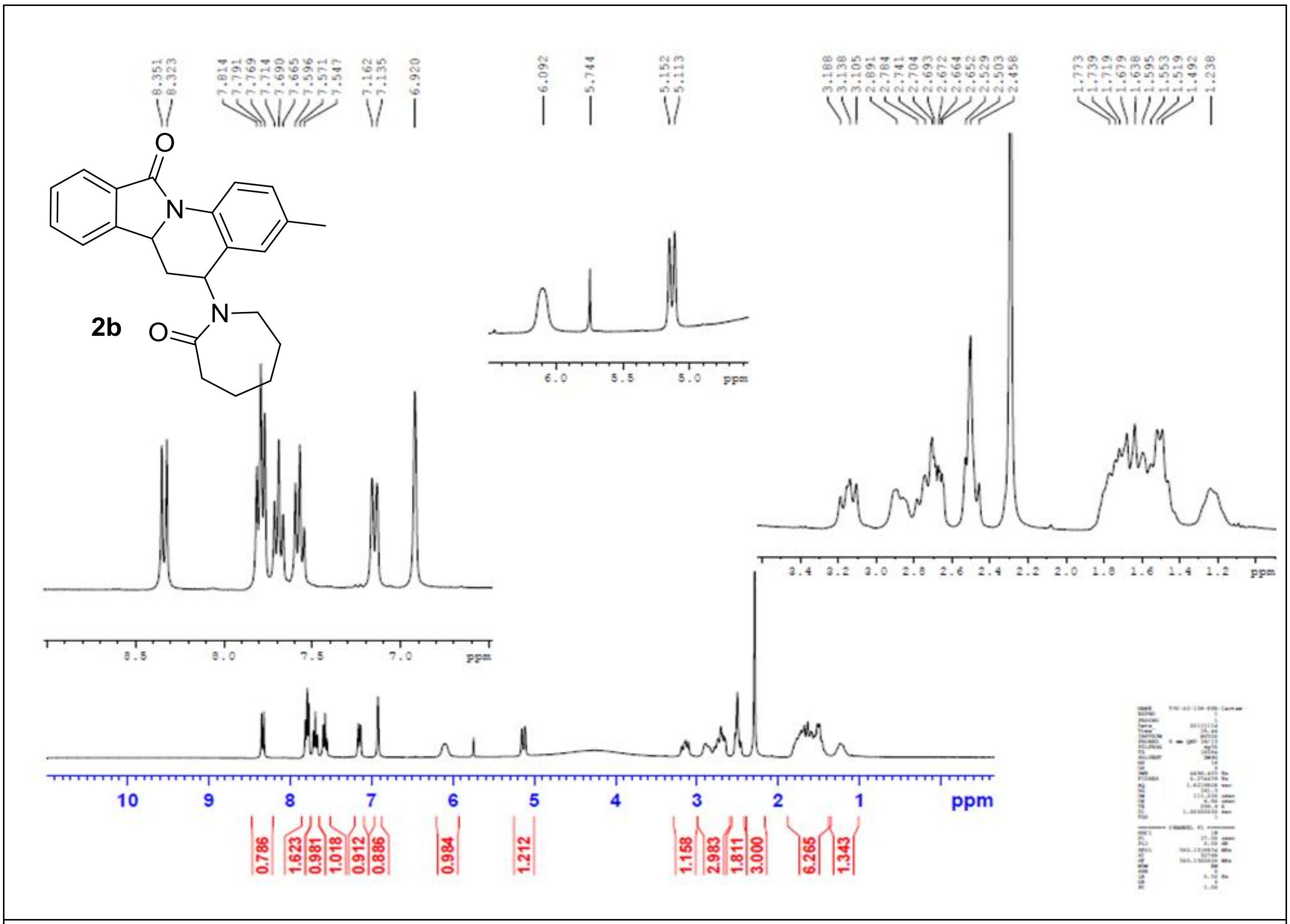
8.351
8.323
7.814
7.791
7.769
7.714
7.690
7.665
7.596
7.571
7.547
7.162
7.135
6.920
6.092
5.744
5.152
5.113
3.188
3.138
3.105
2.891
2.784
2.741
2.704
2.693
2.672
2.664
2.652
2.529
2.503
2.458
1.773
1.739
1.719
1.679
1.638
1.595
1.553
1.519
1.492
1.238
2b
ppm
0.786
1.623
0.981
1.018
0.912
0.886
0.984
1.212
1.158
2.983
1.811
3.000
6.265
1.343

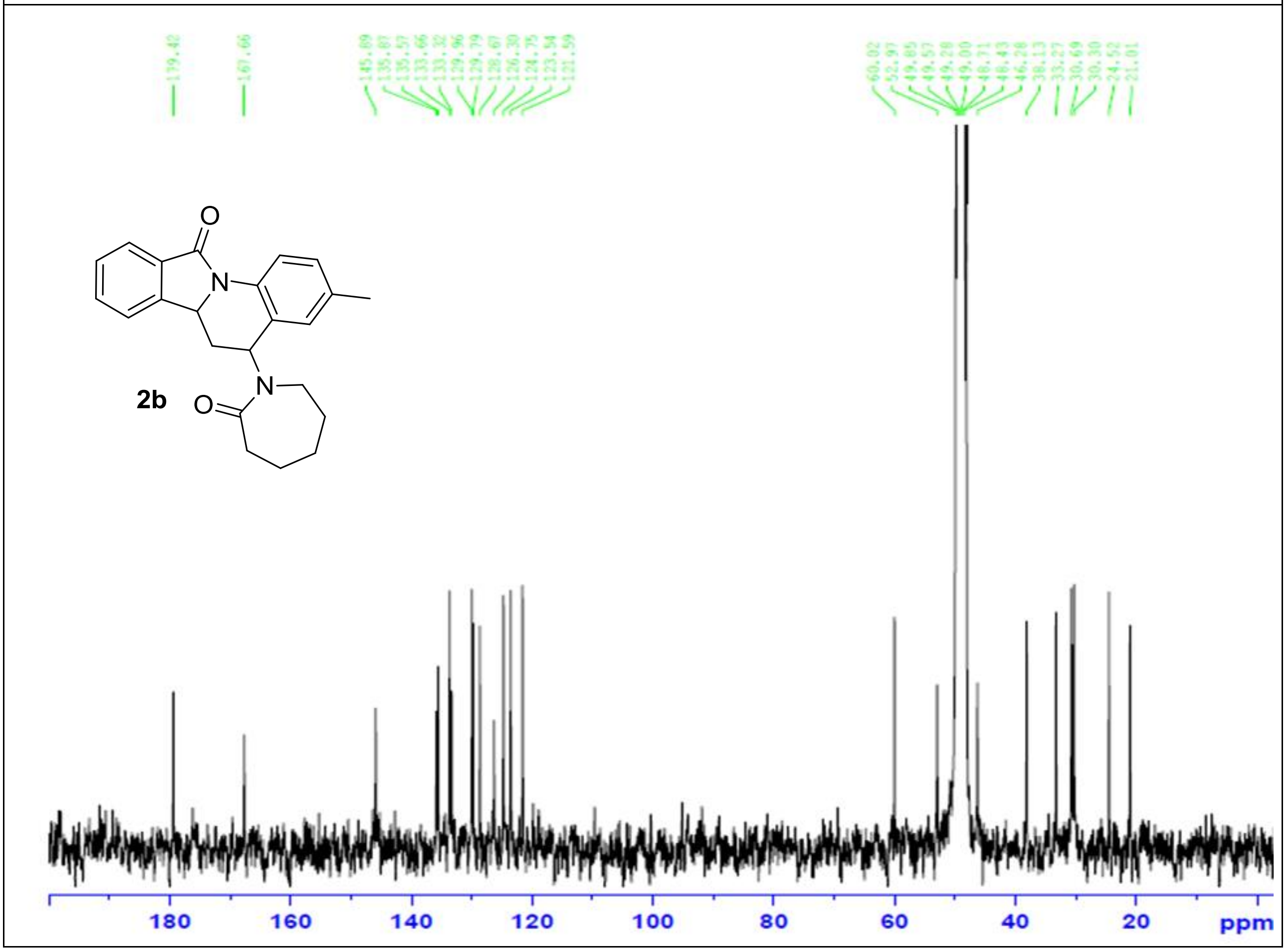
179.42
167.66
145.89
135.87
135.57
133.66
133.32
129.96
129.79
128.67
126.30
124.75
123.54
121.59
60.02
52.97
49.85
49.57
49.28
49.00
48.71
48.43
46.28
38.13
33.27
30.69
30.30
24.52
21.01
2b
ppm

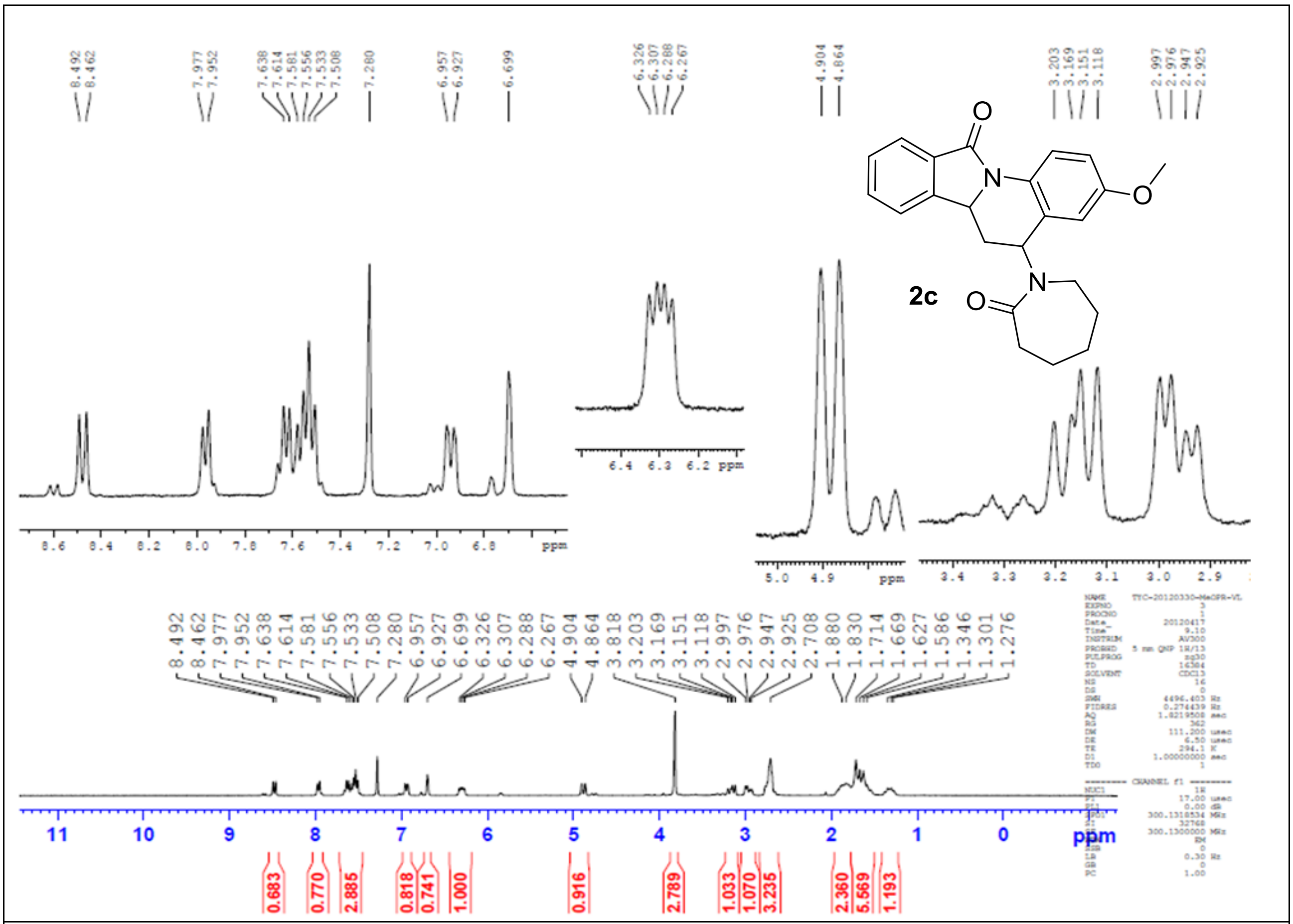
8.492
8.462
7.977
7.952
7.638
7.614
7.581
7.556
7.533
7.508
7.280
6.957
6.927
6.699
6.326
6.307
6.288
6.267
4.904
4.864
3.818
3.203
3.169
3.151
3.118
2.997
2.976
2.947
2.925
2.708
1.880
1.830
1.714
1.669
1.627
1.586
1.346
1.301
1.276
2c
0.683
0.770
2.885
0.818
0.741
1.000
0.916
2.789
1.033
1.070
3.235
2.360
5.569
1.193
ppm

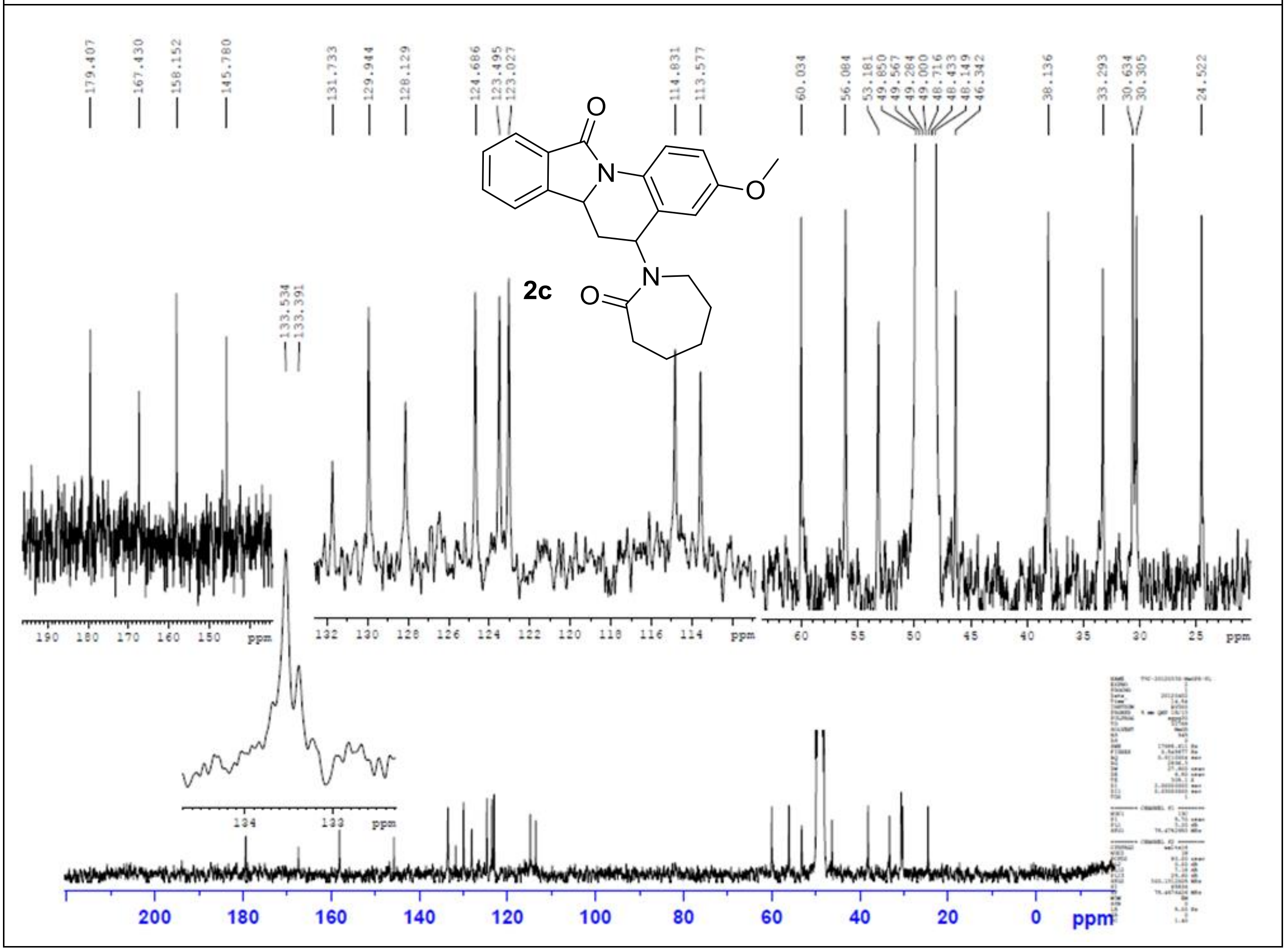
179.407
167.430
158.152
145.780
131.733
129.944
128.129
124.686
123.495
123.027
114.831
113.577
60.034
56.084
53.181
49.850
49.567
49.284
49.000
48.716
48.433
48.149
46.342
38.136
33.293
30.634
30.305
24.522
133.534
133.391
2c
ppm

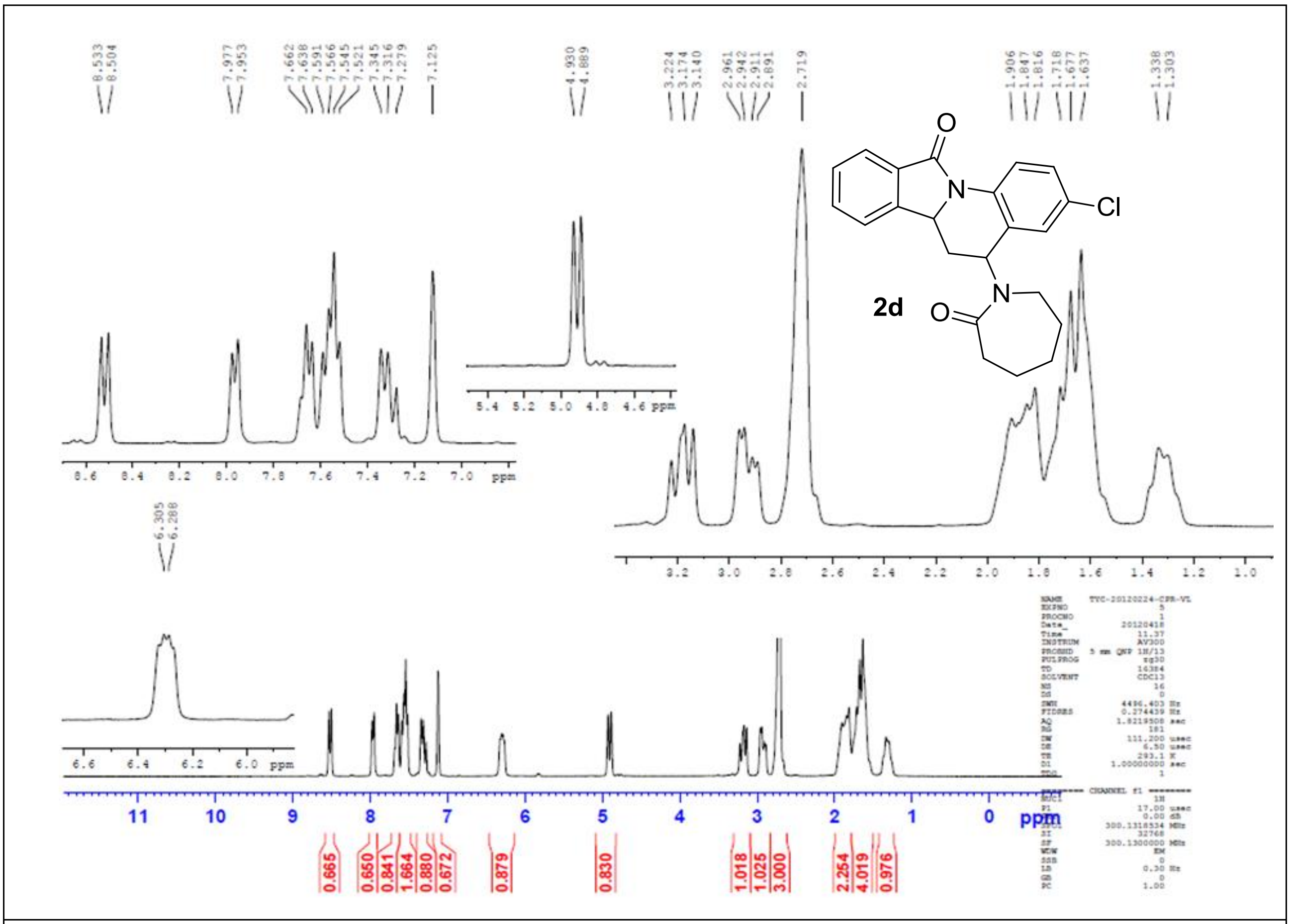

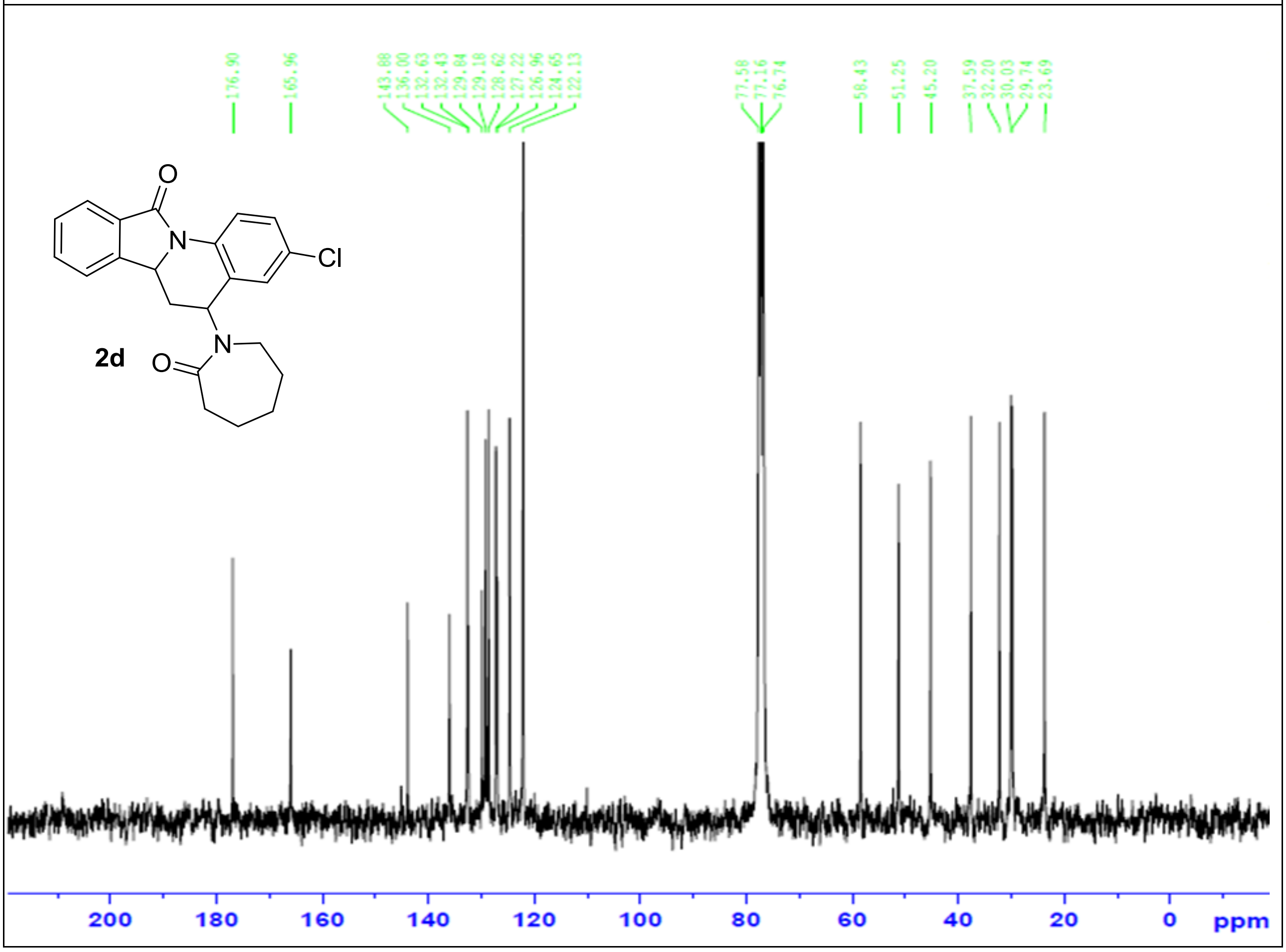

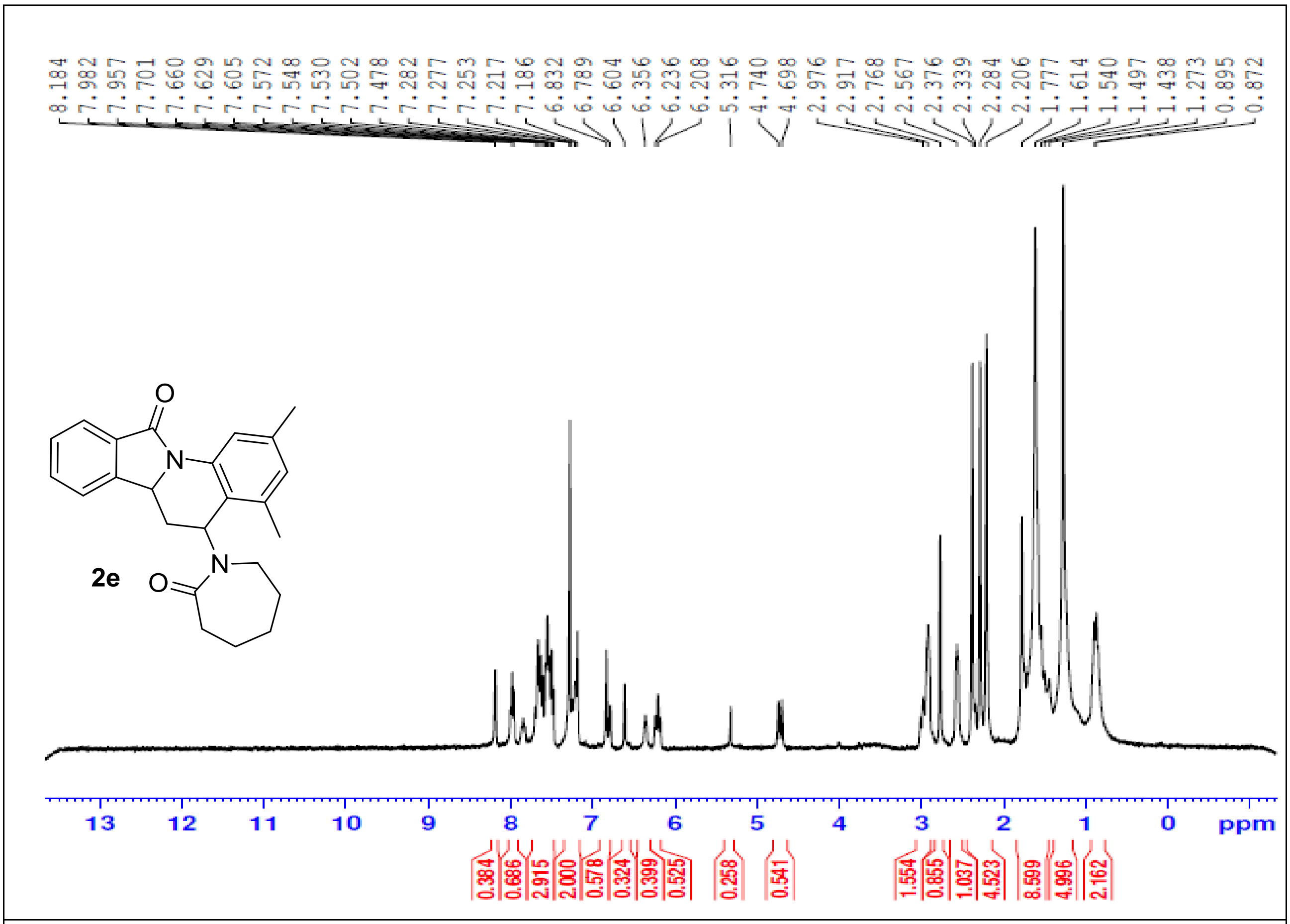
8.184
7.982
7.957
7.701
7.660
7.629
7.605
7.572
7.548
7.530
7.502
7.478
7.282
7.277
7.253
7.217
7.186
6.832
6.789
6.604
6.356
6.236
6.208
5.316
4.740
4.698
2.976
2.917
2.768
2.567
2.376
2.339
2.284
2.206
1.777
1.614
1.540
1.497
1.438
1.273
0.895
0.872
O
N
N
O
2e
13
12
11
10
9
8
7
6
5
4
3
2
1
0
ppm
0.384
0.686
2.915
2.000
0.578
0.324
0.399
0.525
0.258
0.541
1.554
0.855
1.037
4.523
8.599
4.996
2.162

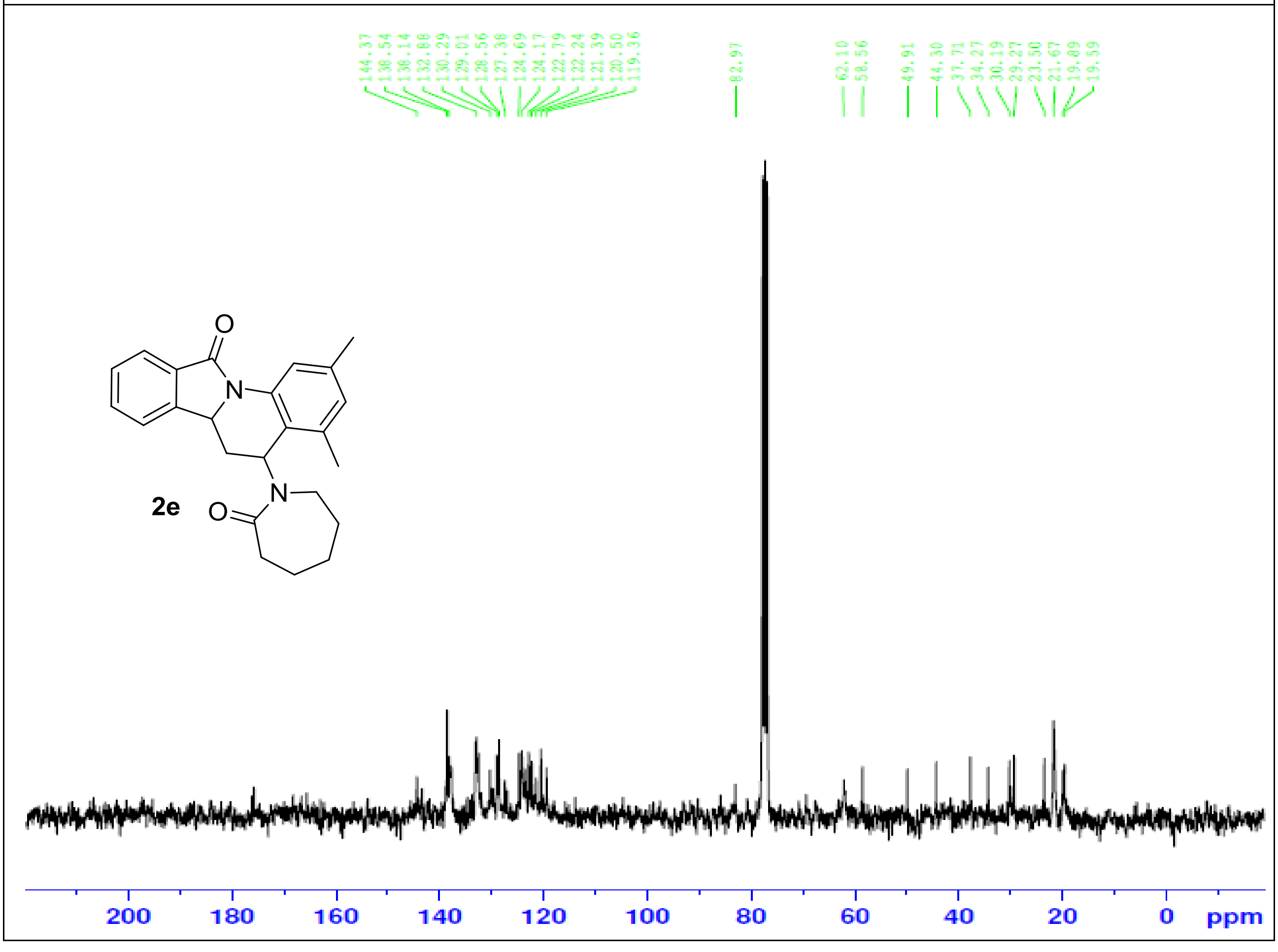
144.37
138.54
138.14
132.88
130.29
129.01
128.56
127.38
124.69
124.17
122.79
122.24
121.39
120.50
119.36
82.97
62.10
58.56
49.91
44.30
37.71
34.27
30.19
29.27
23.50
21.67
19.89
19.59
O
N
N
O
2e
200
180
160
140
120
100
80
60
40
20
0
ppm

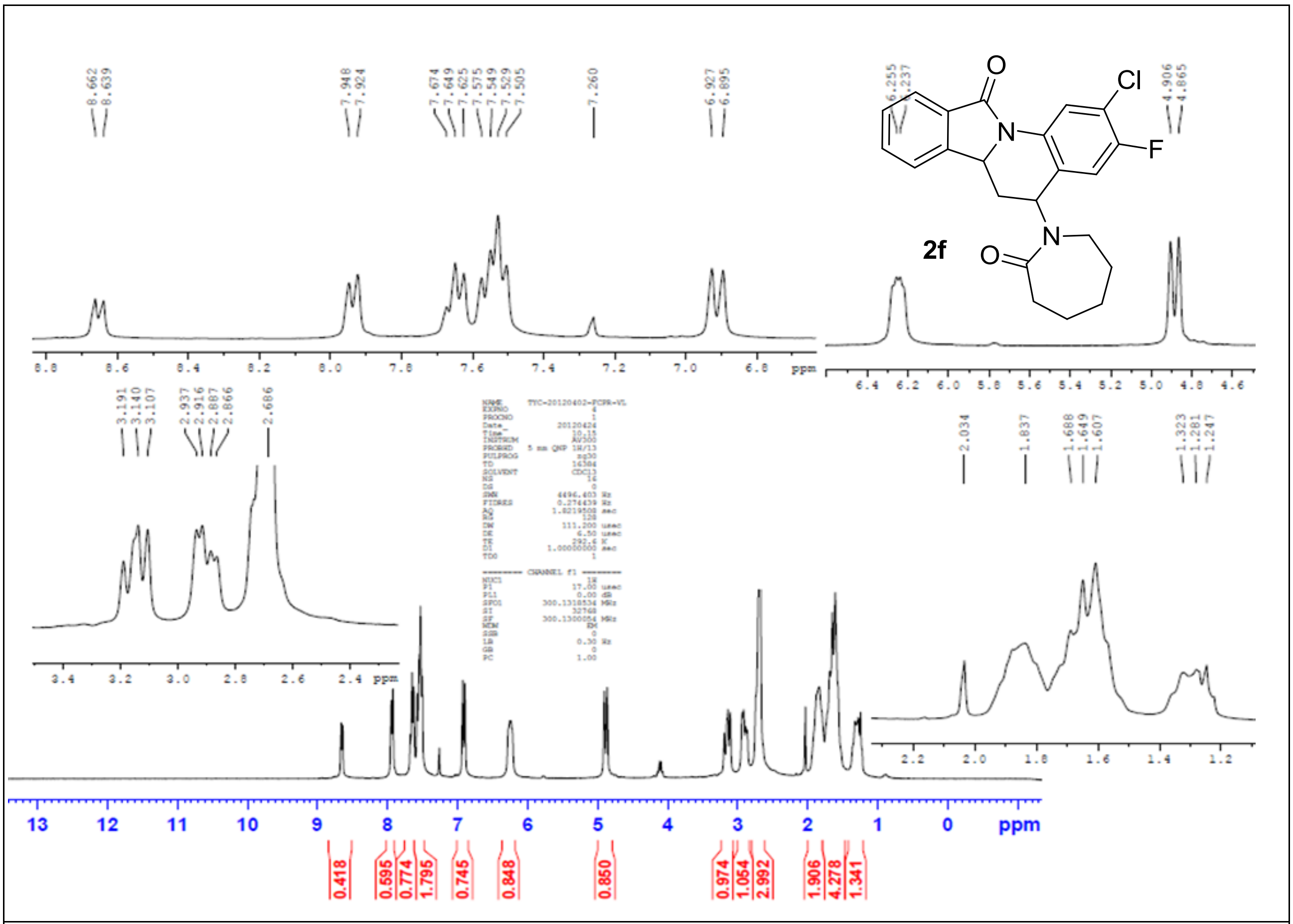

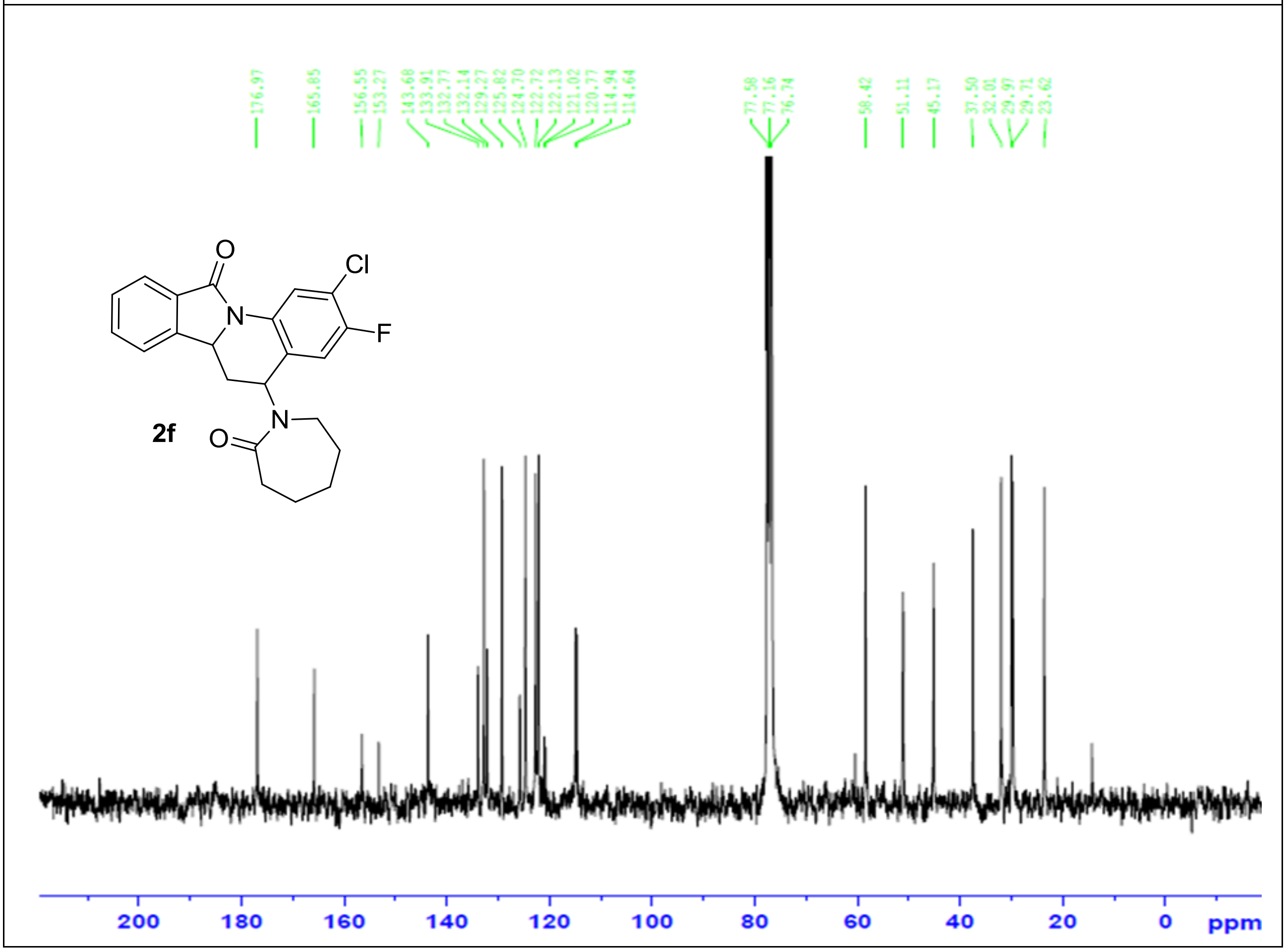

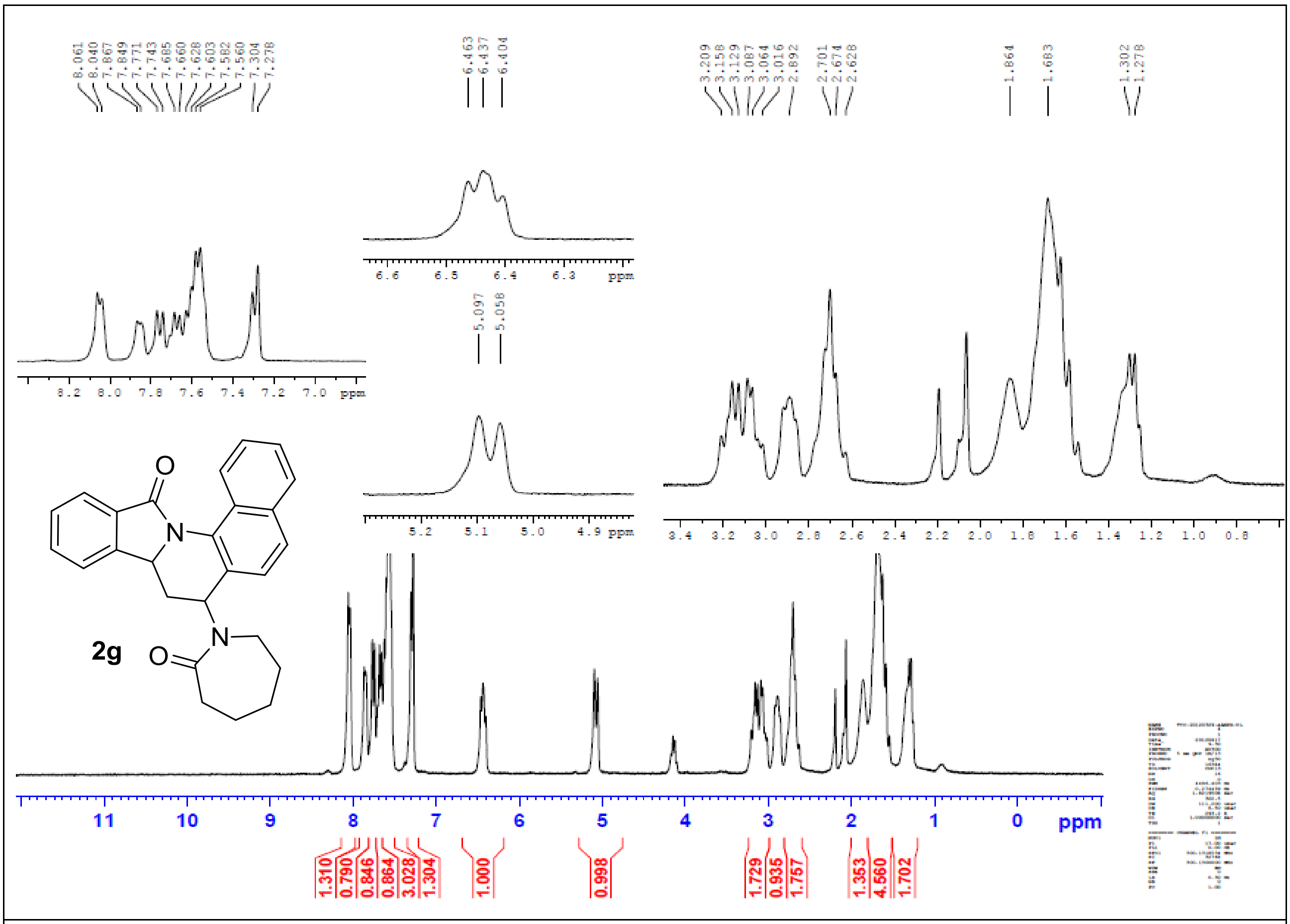
8.061 8.040 7.867 7.849 7.771 7.743 7.685 7.660 7.628 7.603 7.582 7.560 7.304 7.278
6.463 6.437 6.404
5.097 5.058
3.209 3.158 3.129 3.087 3.064 3.016 2.892 2.701 2.674 2.628
1.864
1.683
1.302 1.278
2g
N
O
ppm
1.310 0.790 0.846 0.864 3.028 1.304 1.000 0.998 1.729 0.935 1.757 1.353 4.560 1.702

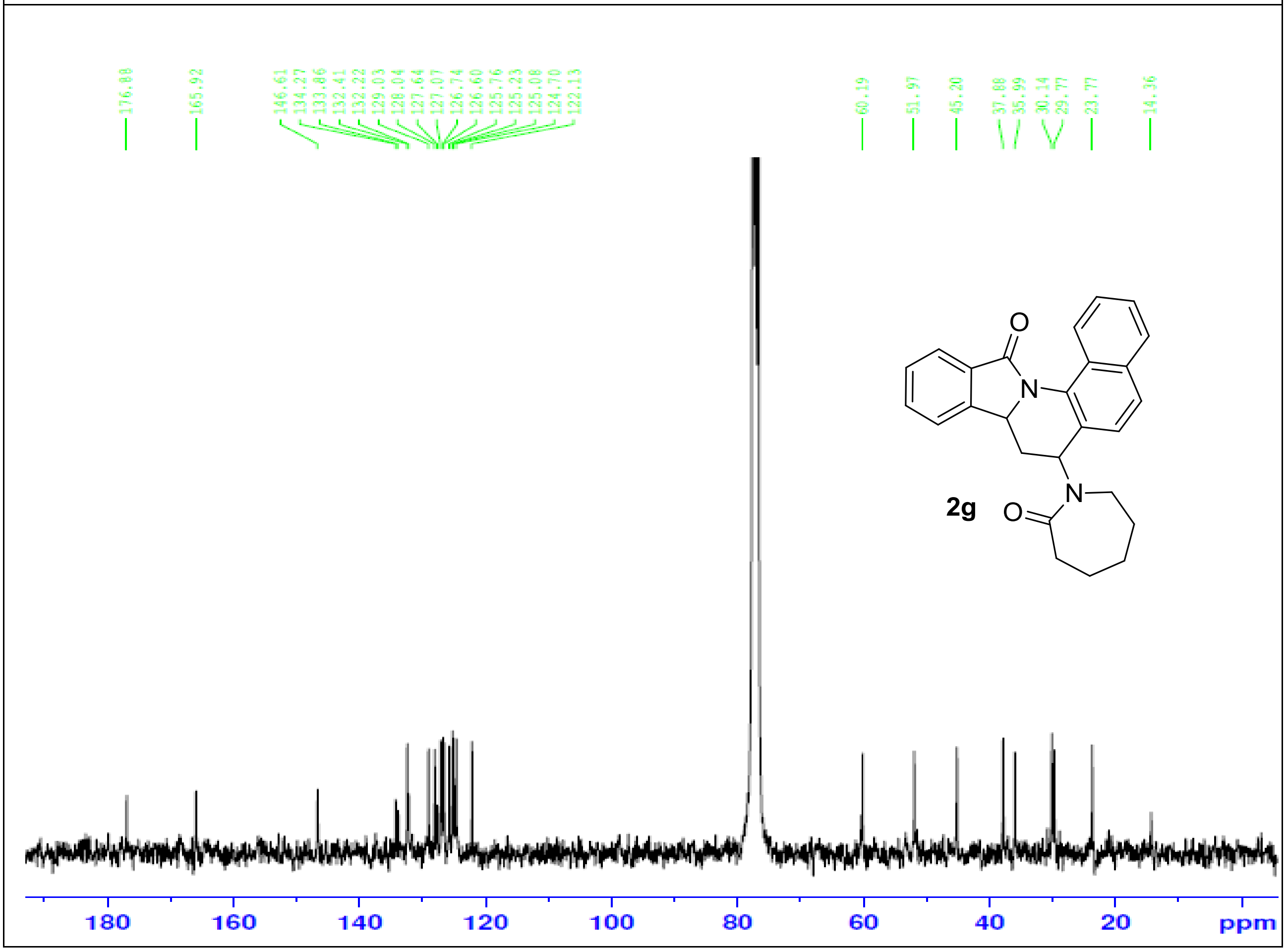
176.88
165.92
146.61 134.27 133.86 132.41 132.22 129.03 128.04 127.64 127.07 126.74 126.60 125.76 125.23 125.08 124.70 122.13
60.19
51.97
45.20
37.88 35.99
30.14 29.77
23.77
14.36
2g
N
O
ppm

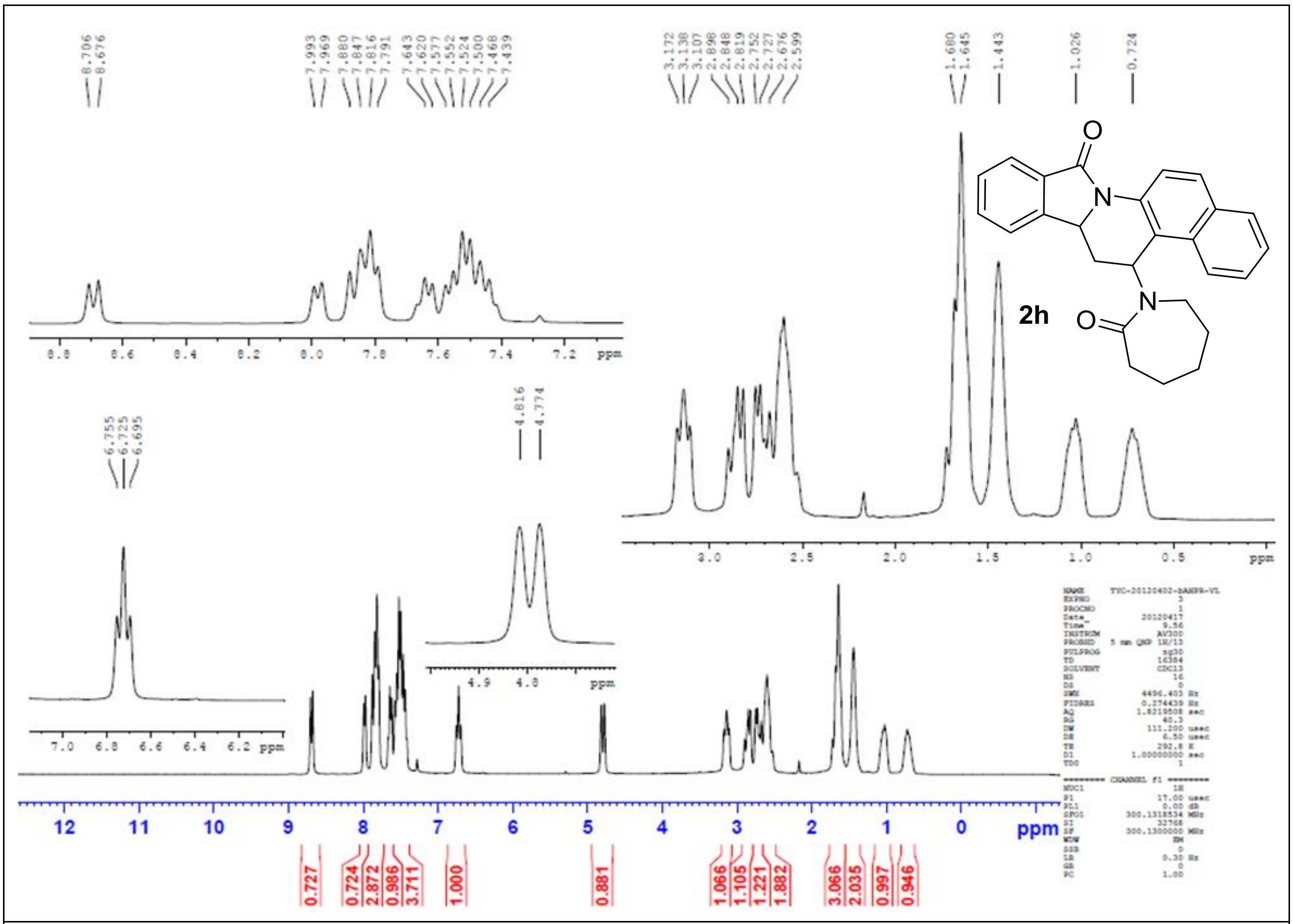
2h
NAME TYC-20120402-bANPR-VL
SOLVENT CDCl3
ppm

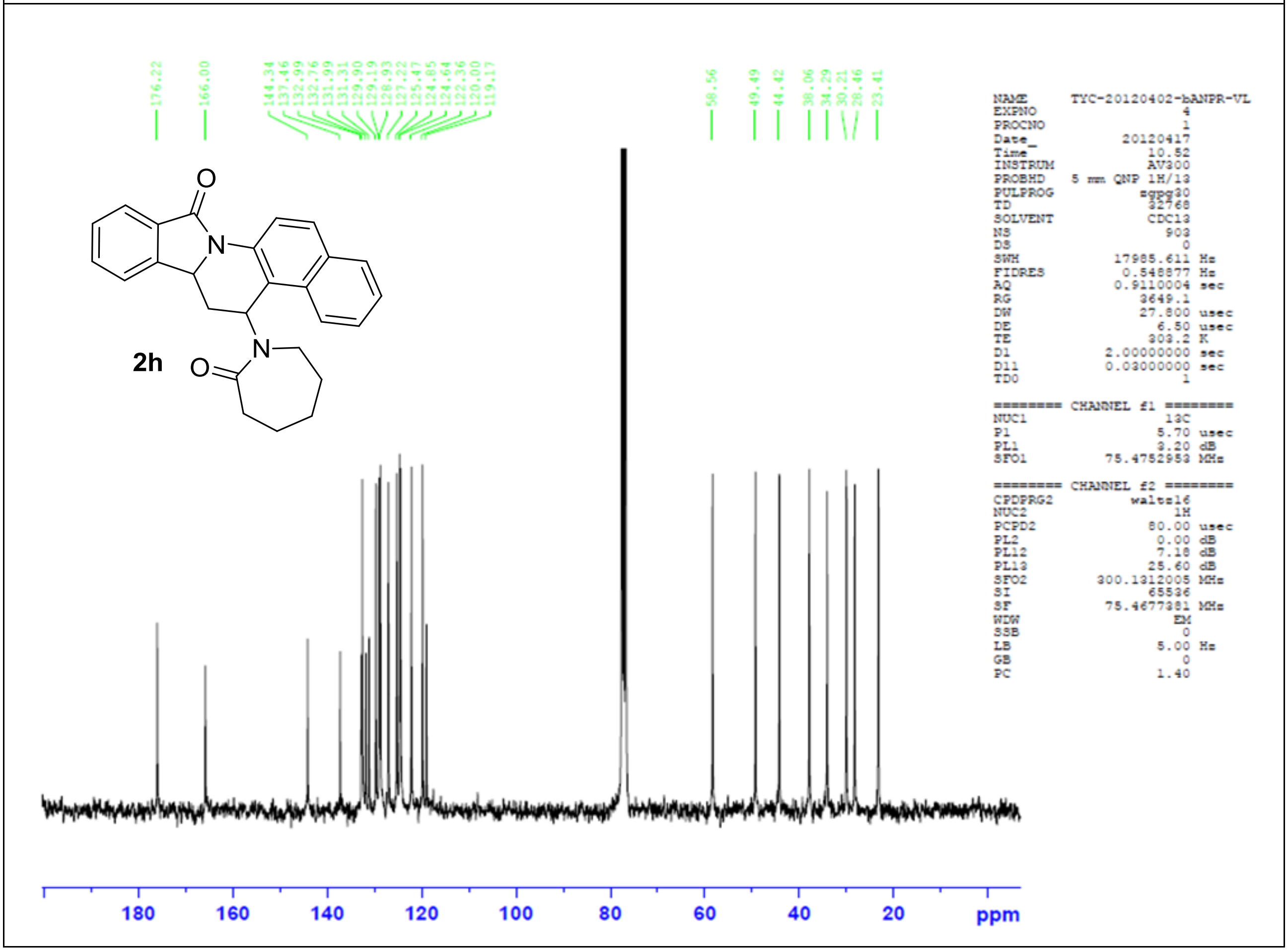
176.22
166.00
144.34
137.46
132.99
132.76
131.99
131.31
129.90
129.19
128.93
127.22
125.47
124.85
124.64
122.36
120.00
119.17
58.56
49.49
44.42
38.06
34.29
30.21
28.46
23.41
2h
NAME TYC-20120402-bANPR-VL
EXPNO 4
PROCNO 1
Date_ 20120417
Time 10.52
INSTRUM AV300
PROBHD 5 mm QNP 1H/13
PULPROG zgpg30
TD 32768
SOLVENT CDCl3
NS 903
DS 0
SWH 17985.611 Hz
FIDRES 0.548877 Hz
AQ 0.9110004 sec
RG 3649.1
DW 27.800 usec
DE 6.50 usec
TE 303.2 K
D1 2.00000000 sec
D11 0.03000000 sec
TD0 1
======== CHANNEL f1 ========
NUC1 13C
P1 5.70 usec
PL1 3.20 dB
SFO1 75.4752953 MHz
======== CHANNEL f2 ========
CPDPRG2 waltz16
NUC2 1H
PCPD2 80.00 usec
PL2 0.00 dB
PL12 7.18 dB
PL13 25.60 dB
SFO2 300.1312005 MHz
SI 65536
SF 75.4677381 MHz
WDW EM
SSB 0
LB 5.00 Hz
GB 0
PC 1.40
ppm

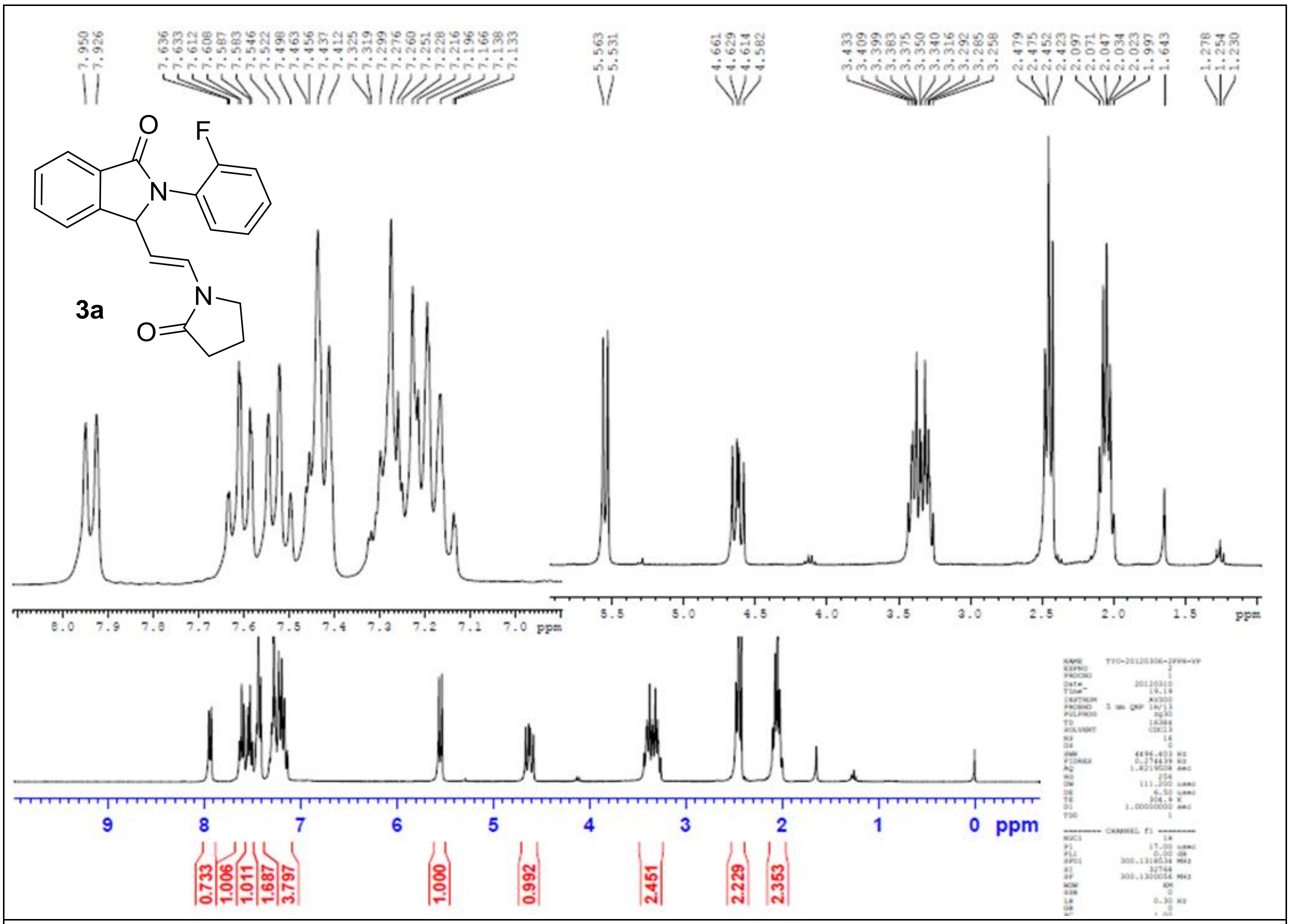
3a
7.950
7.926
7.636
7.633
7.612
7.608
7.587
7.583
7.546
7.522
7.498
7.463
7.456
7.437
7.412
7.325
7.319
7.299
7.276
7.260
7.251
7.228
7.216
7.196
7.166
7.138
7.133
5.563
5.531
4.661
4.629
4.614
4.582
3.433
3.409
3.399
3.383
3.375
3.350
3.340
3.316
3.292
3.285
3.258
2.479
2.475
2.452
2.423
2.097
2.071
2.047
2.034
2.023
1.997
1.643
1.278
1.254
1.230
0.733
1.006
1.011
1.687
3.797
1.000
0.992
2.451
2.229
2.353
ppm

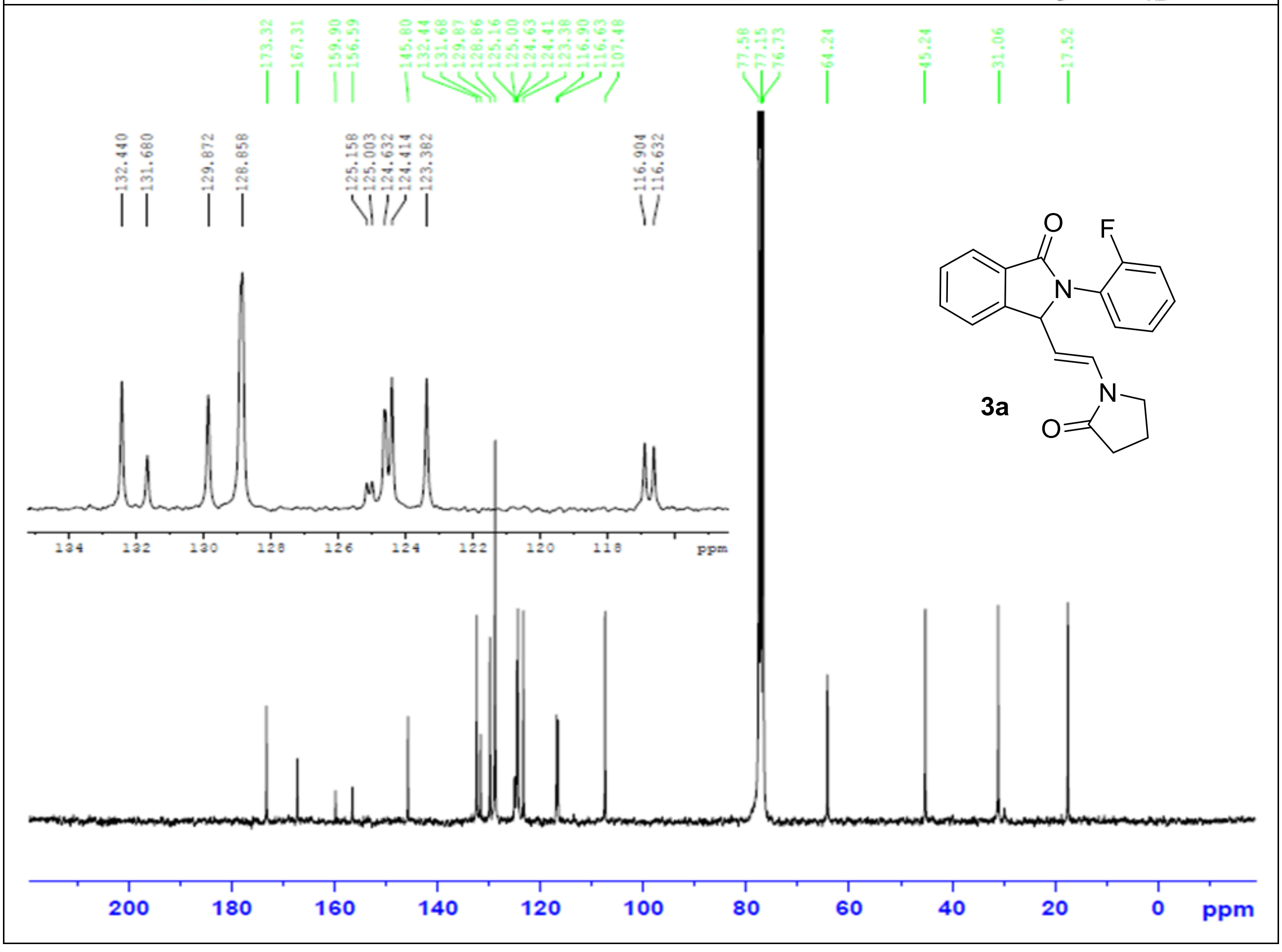
173.32
167.31
159.90
156.59
145.80
132.44
131.68
129.87
128.86
125.16
125.00
124.63
124.41
123.38
116.90
116.63
107.48
77.58
77.15
76.73
64.24
45.24
31.06
17.52
132.440
131.680
129.872
128.858
125.158
125.003
124.632
124.414
123.382
116.904
116.632
3a
ppm

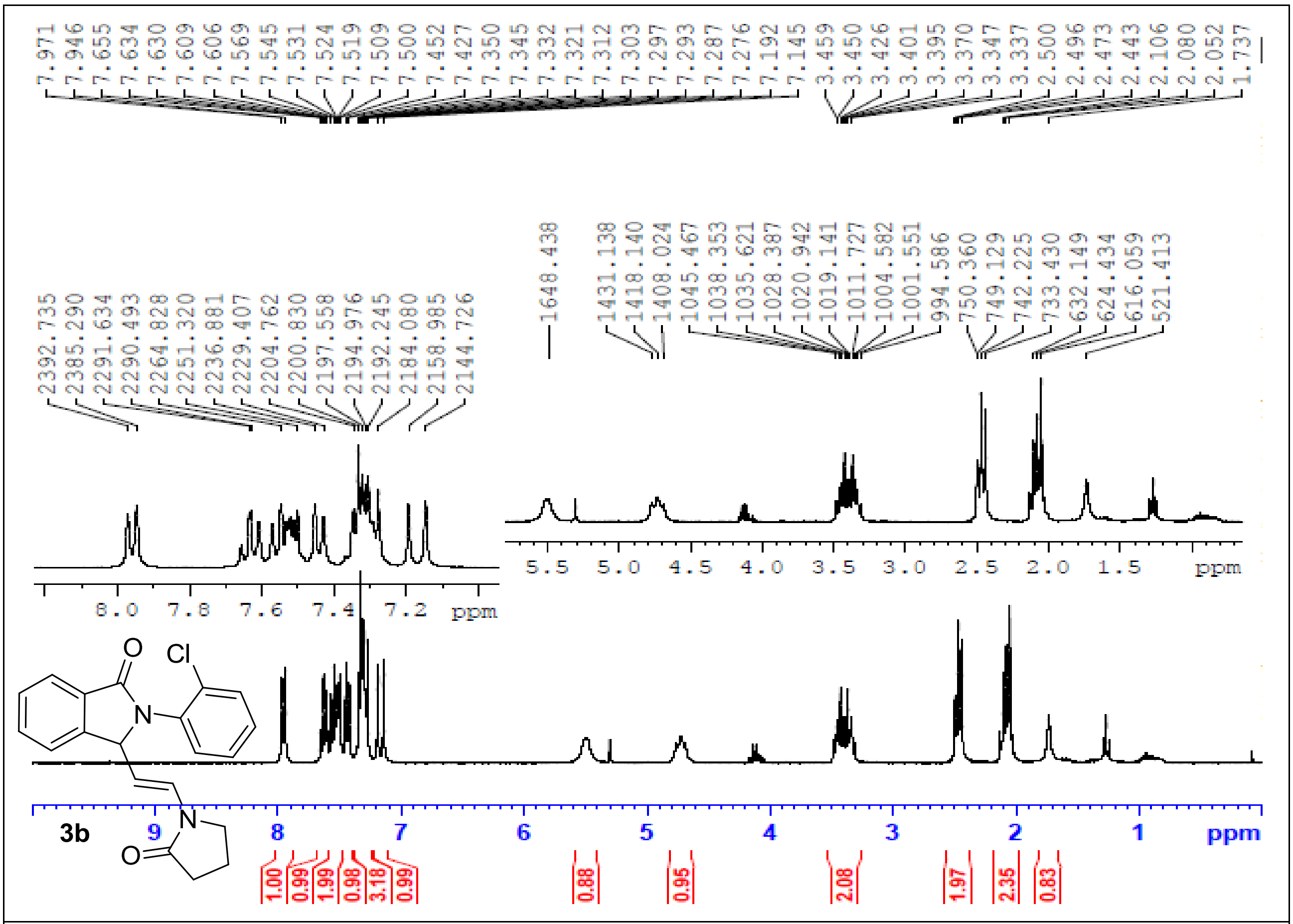
7.971 7.946 7.655 7.634 7.630 7.609 7.606 7.569 7.545 7.531 7.524 7.519 7.509 7.500 7.452 7.427 7.350 7.345 7.332 7.321 7.312 7.303 7.297 7.293 7.287 7.276 7.192 7.145 3.459 3.450 3.426 3.401 3.395 3.370 3.347 3.337 2.500 2.496 2.473 2.443 2.106 2.080 2.052 1.737
2392.735 2385.290 2291.634 2290.493 2264.828 2251.320 2236.881 2229.407 2204.762 2200.830 2197.558 2194.976 2192.245 2184.080 2158.985 2144.726
1648.438 1431.138 1418.140 1408.024 1045.467 1038.353 1035.621 1028.387 1020.942 1019.141 1011.727 1004.582 1001.551 994.586 750.360 749.129 742.225 733.430 632.149 624.434 616.059 521.413
8.0 7.8 7.6 7.4 7.2 ppm
5.5 5.0 4.5 4.0 3.5 3.0 2.5 2.0 1.5 ppm
O
Cl
N
N
O
3b
9 8 7 6 5 4 3 2 1 ppm
1.00 0.99 1.99 0.98 3.18 0.99 0.88 0.95 2.08 1.97 2.35 0.83

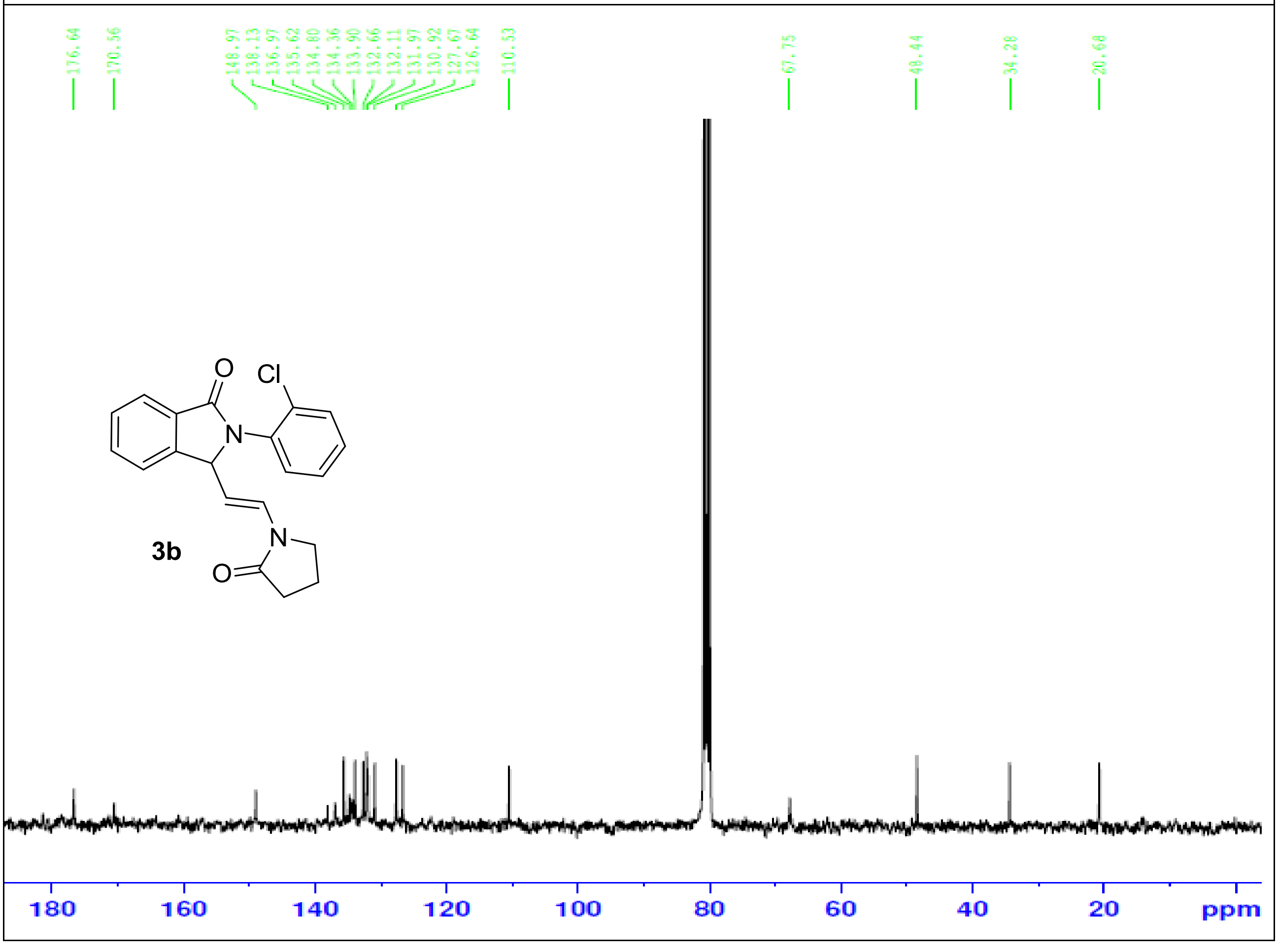
176.64 170.56 148.97 138.13 136.97 135.62 134.80 134.36 133.90 132.66 132.11 131.97 130.92 127.67 126.64 110.53 67.75 48.44 34.28 20.68
O
Cl
N
N
O
3b
180 160 140 120 100 80 60 40 20 ppm

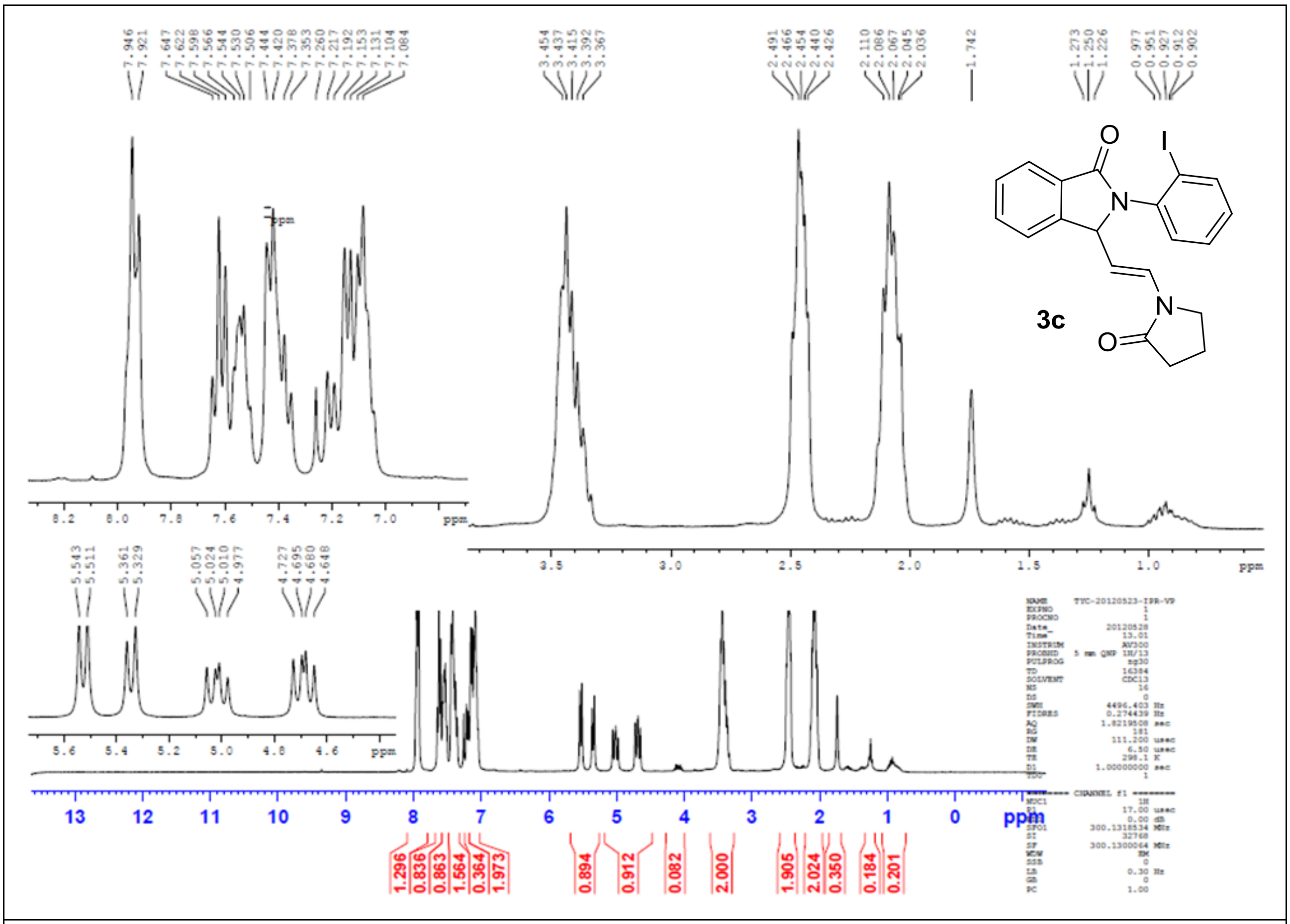
7.946
7.921
7.647
7.622
7.598
7.566
7.544
7.530
7.506
7.444
7.420
7.378
7.353
7.260
7.217
7.192
7.153
7.131
7.104
7.084
3.454
3.437
3.415
3.392
3.367
2.491
2.466
2.454
2.440
2.426
2.110
2.086
2.067
2.045
2.036
1.742
1.273
1.250
1.226
0.977
0.951
0.927
0.912
0.902
5.543
5.511
5.361
5.329
5.057
5.024
5.010
4.977
4.727
4.695
4.680
4.648
3c
1.296
0.836
0.863
1.564
0.364
1.973
0.894
0.912
0.082
2.000
1.905
2.024
0.350
0.184
0.201
ppm

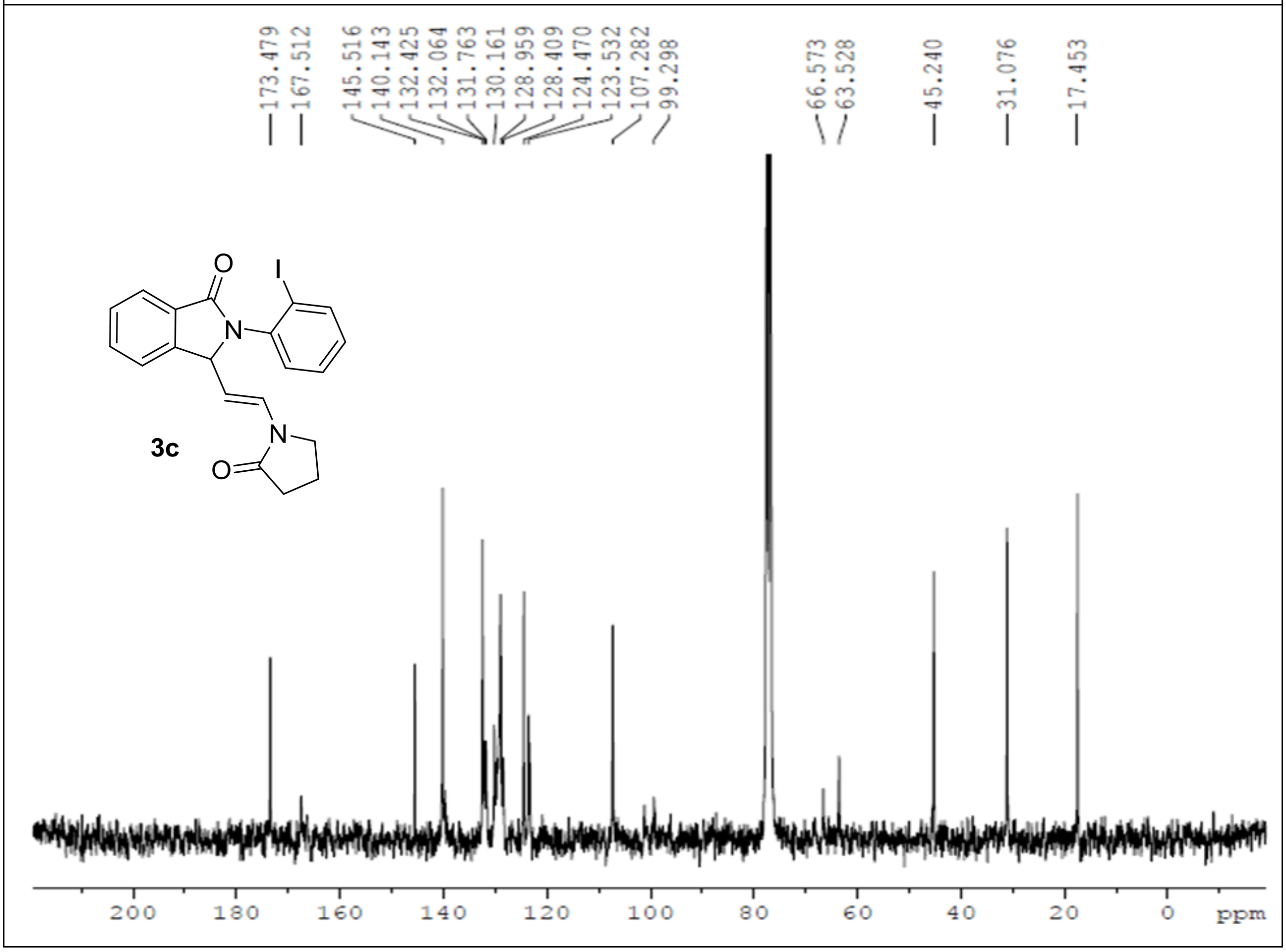
173.479
167.512
145.516
140.143
132.425
132.064
131.763
130.161
128.959
128.409
124.470
123.532
107.282
99.298
66.573
63.528
45.240
31.076
17.453
3c
ppm

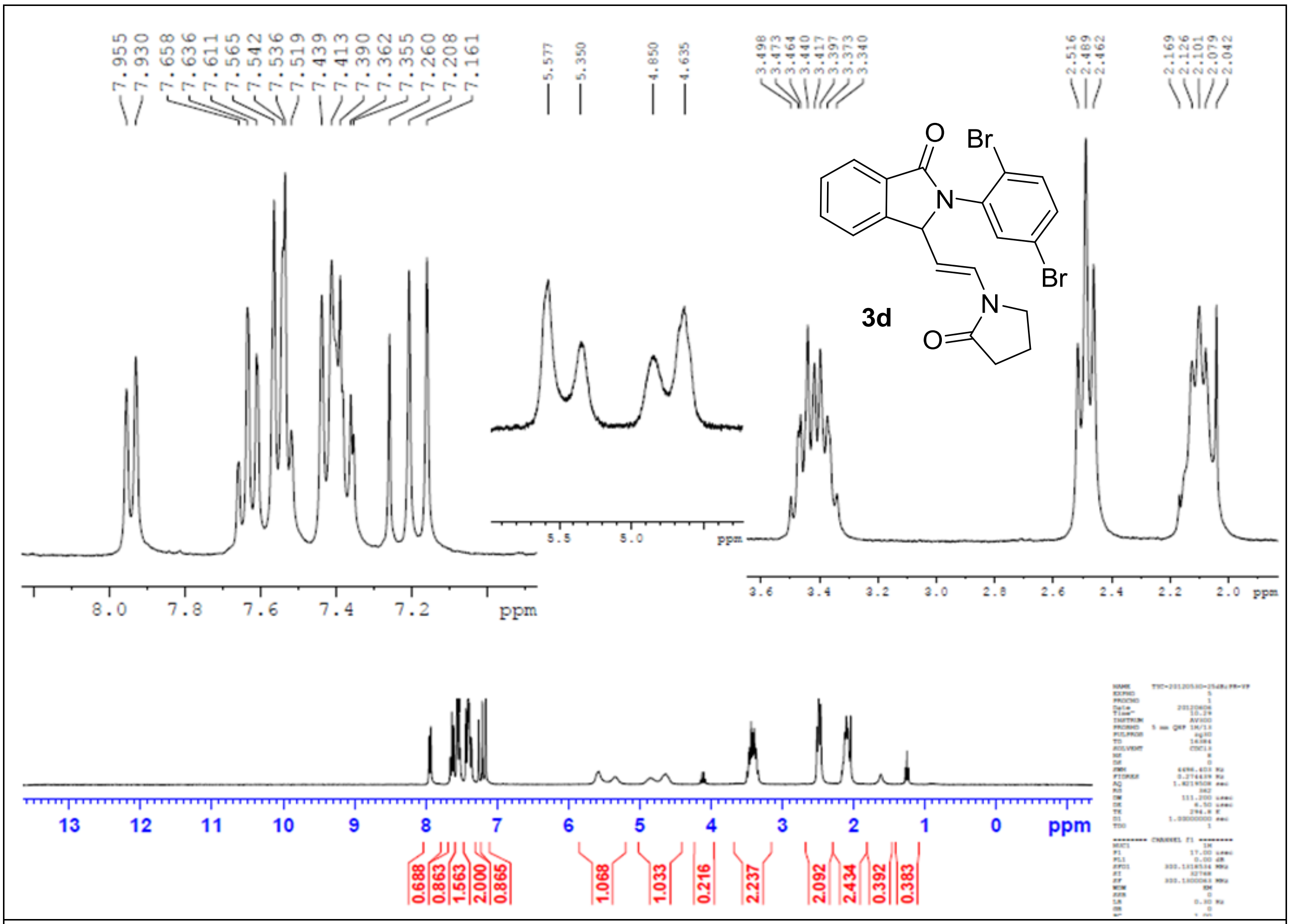
3d
7.955
7.930
7.658
7.636
7.611
7.565
7.542
7.536
7.519
7.439
7.413
7.390
7.362
7.355
7.260
7.208
7.161
5.577
5.350
4.850
4.635
3.498
3.473
3.464
3.440
3.417
3.397
3.373
3.340
2.516
2.489
2.462
2.169
2.126
2.101
2.079
2.042
0.688
0.863
1.563
2.000
0.865
1.068
1.033
0.216
2.237
2.092
2.434
0.392
0.383
ppm

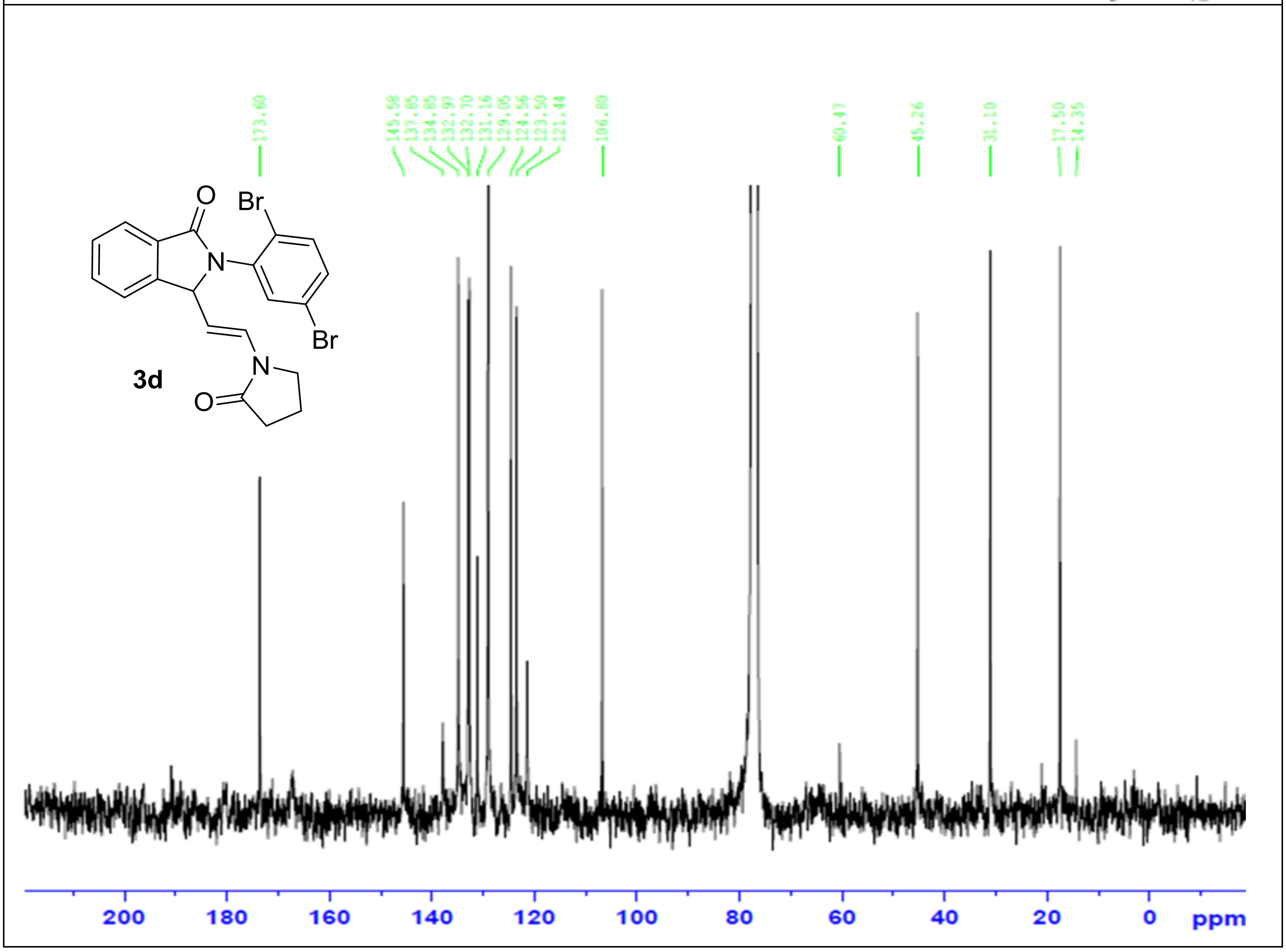
3d
173.60
145.58
137.85
134.85
132.97
132.70
131.16
129.05
124.56
123.50
121.44
106.80
60.47
45.26
31.10
17.50
14.35
ppm

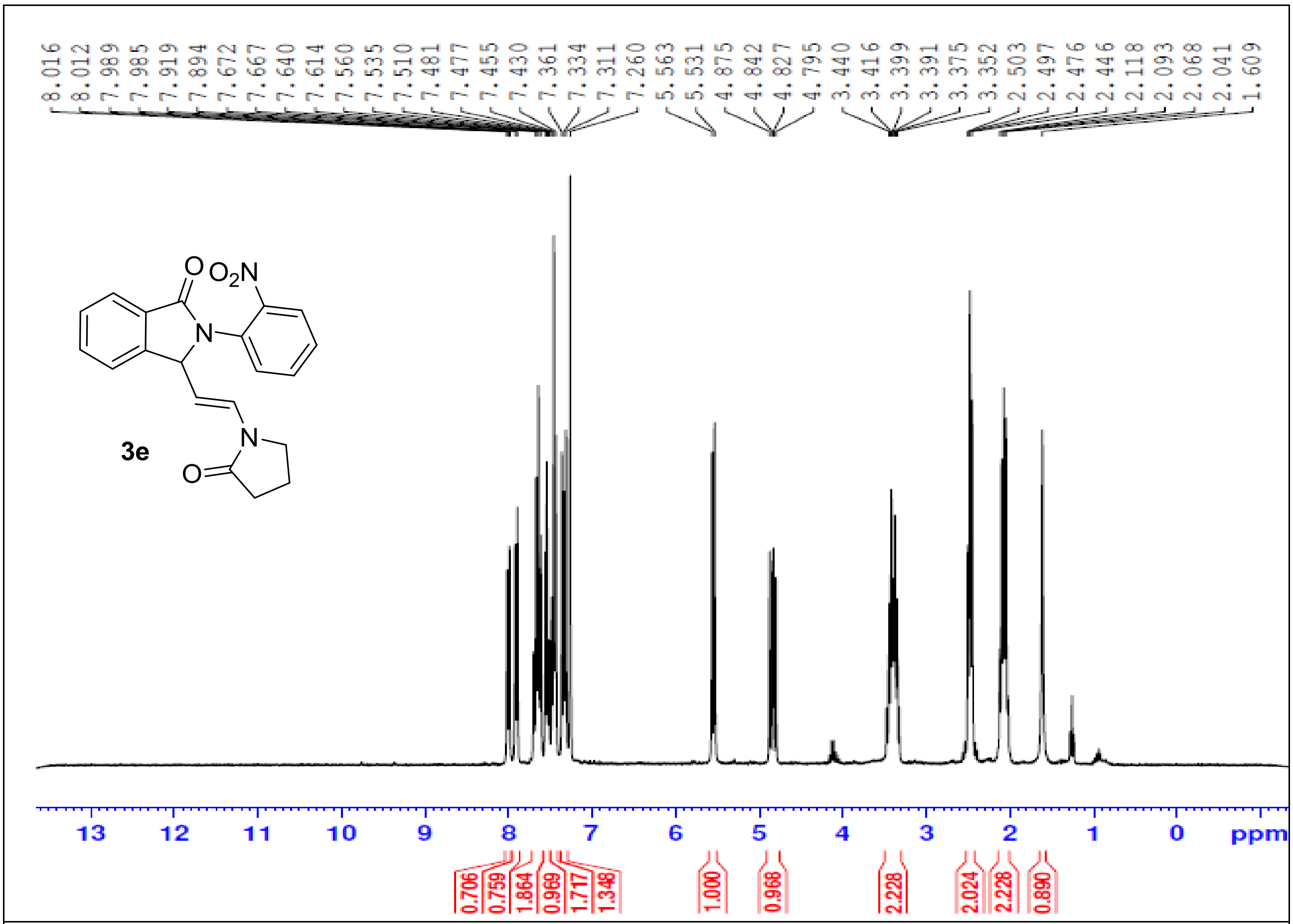
8.016 8.012 7.989 7.985 7.919 7.894 7.672 7.667 7.640 7.614 7.560 7.535 7.510 7.481 7.477 7.455 7.430 7.361 7.334 7.311 7.260 5.563 5.531 4.875 4.842 4.827 4.795 3.440 3.416 3.399 3.391 3.375 3.352 2.503 2.497 2.476 2.446 2.118 2.093 2.068 2.041 1.609
O
O_2N
N
3e
N
O
13 12 11 10 9 8 7 6 5 4 3 2 1 0 ppm
0.706 0.759 1.864 0.969 1.717 1.348 1.000 0.968 2.228 2.024 2.228 0.890

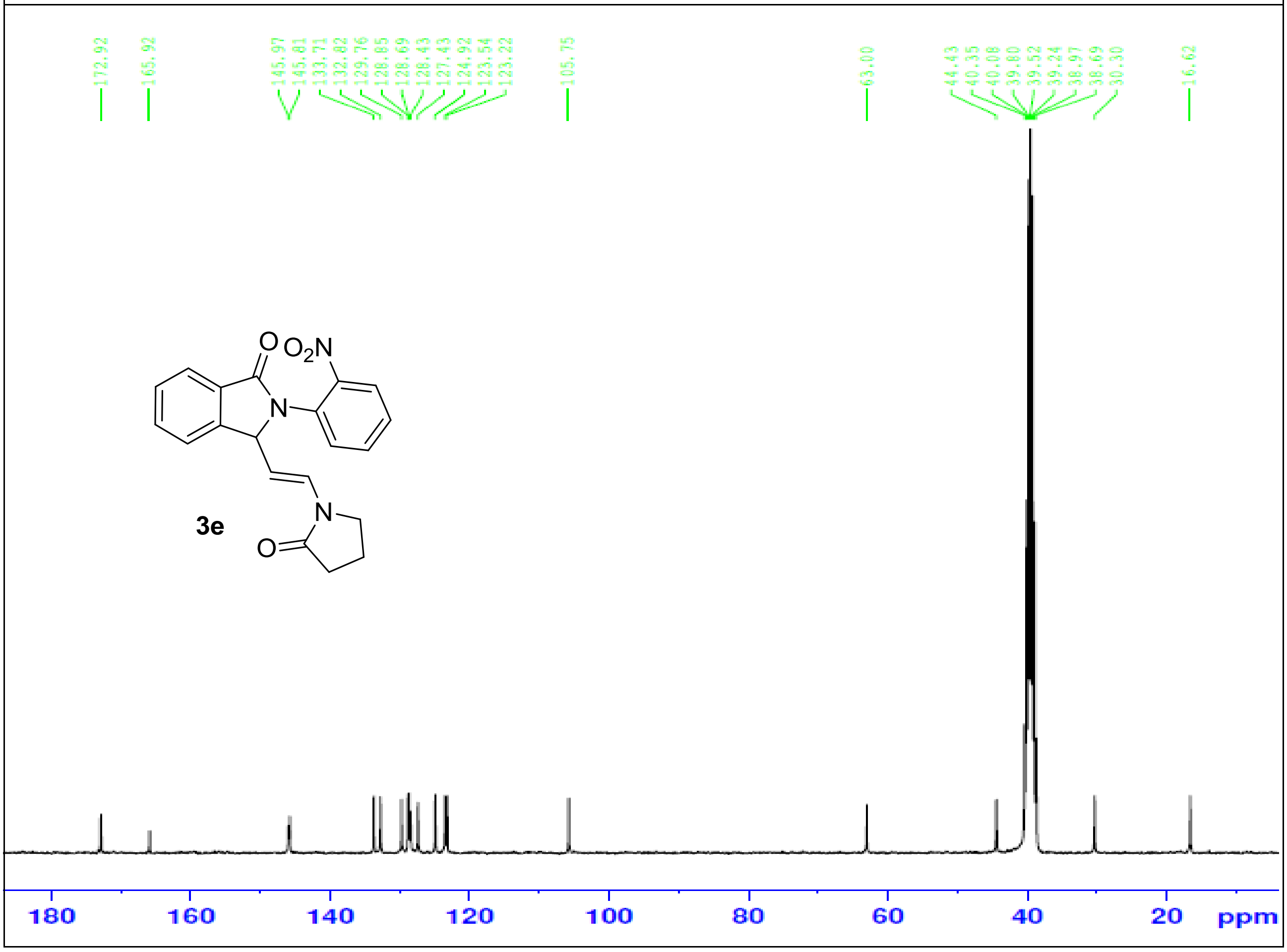
172.92 165.92 145.97 145.81 133.71 132.82 129.76 128.85 128.69 128.43 127.43 124.92 123.54 123.22 105.75 63.00 44.43 40.35 40.08 39.80 39.52 39.24 38.97 38.69 30.30 16.62
O
O_2N
N
3e
N
O
180 160 140 120 100 80 60 40 20 ppm

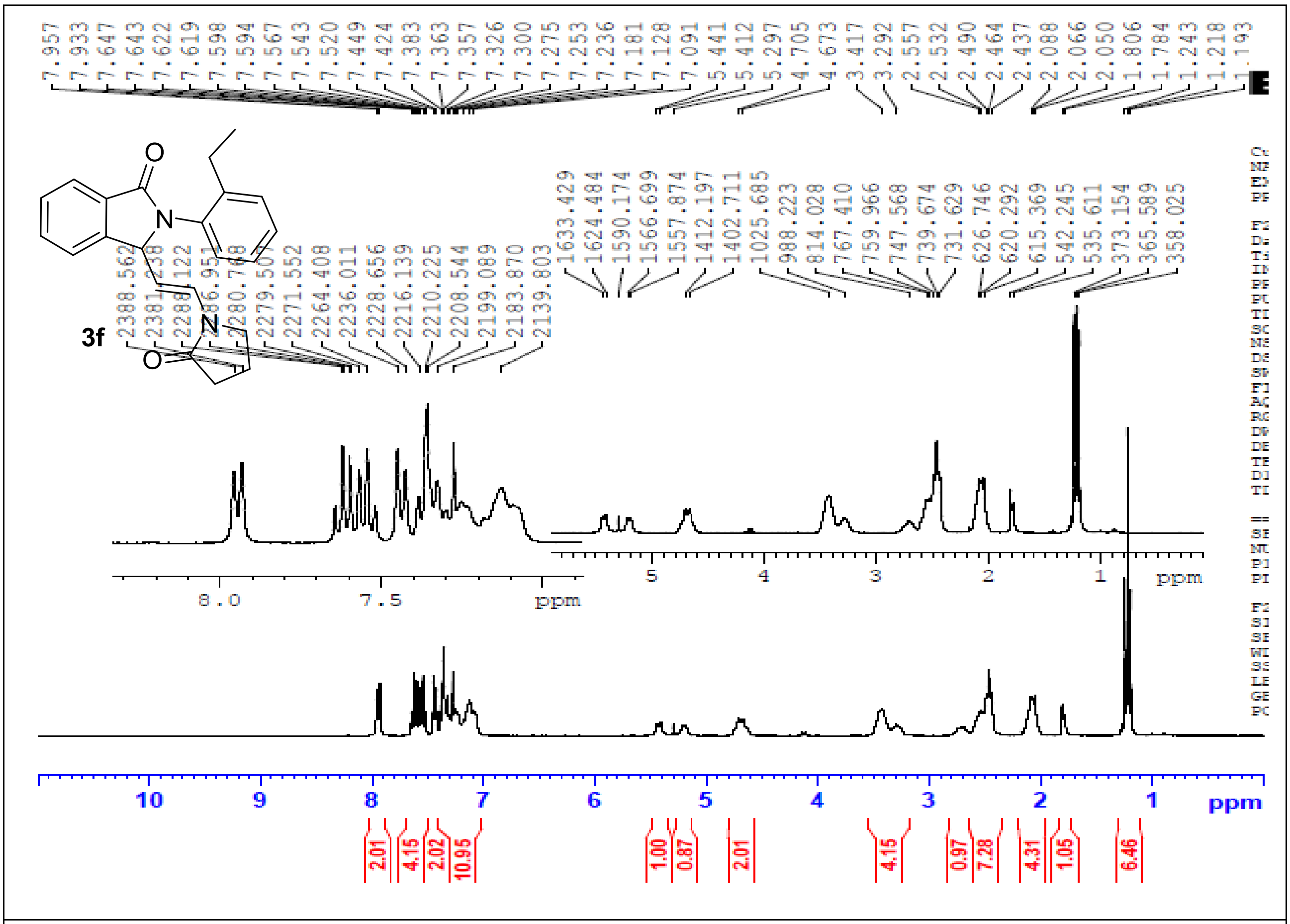
7.957 7.933 7.647 7.643 7.622 7.619 7.598 7.594 7.567 7.543 7.520 7.449 7.424 7.383 7.363 7.357 7.326 7.300 7.275 7.253 7.236 7.181 7.128 7.091 5.441 5.412 5.297 4.705 4.673 3.417 3.292 2.557 2.532 2.490 2.464 2.437 2.088 2.066 2.050 1.806 1.784 1.243 1.218 1.193
3f
ppm
2.01 4.15 2.02 10.95 1.00 0.87 2.01 4.15 0.97 7.28 4.31 1.05 6.46

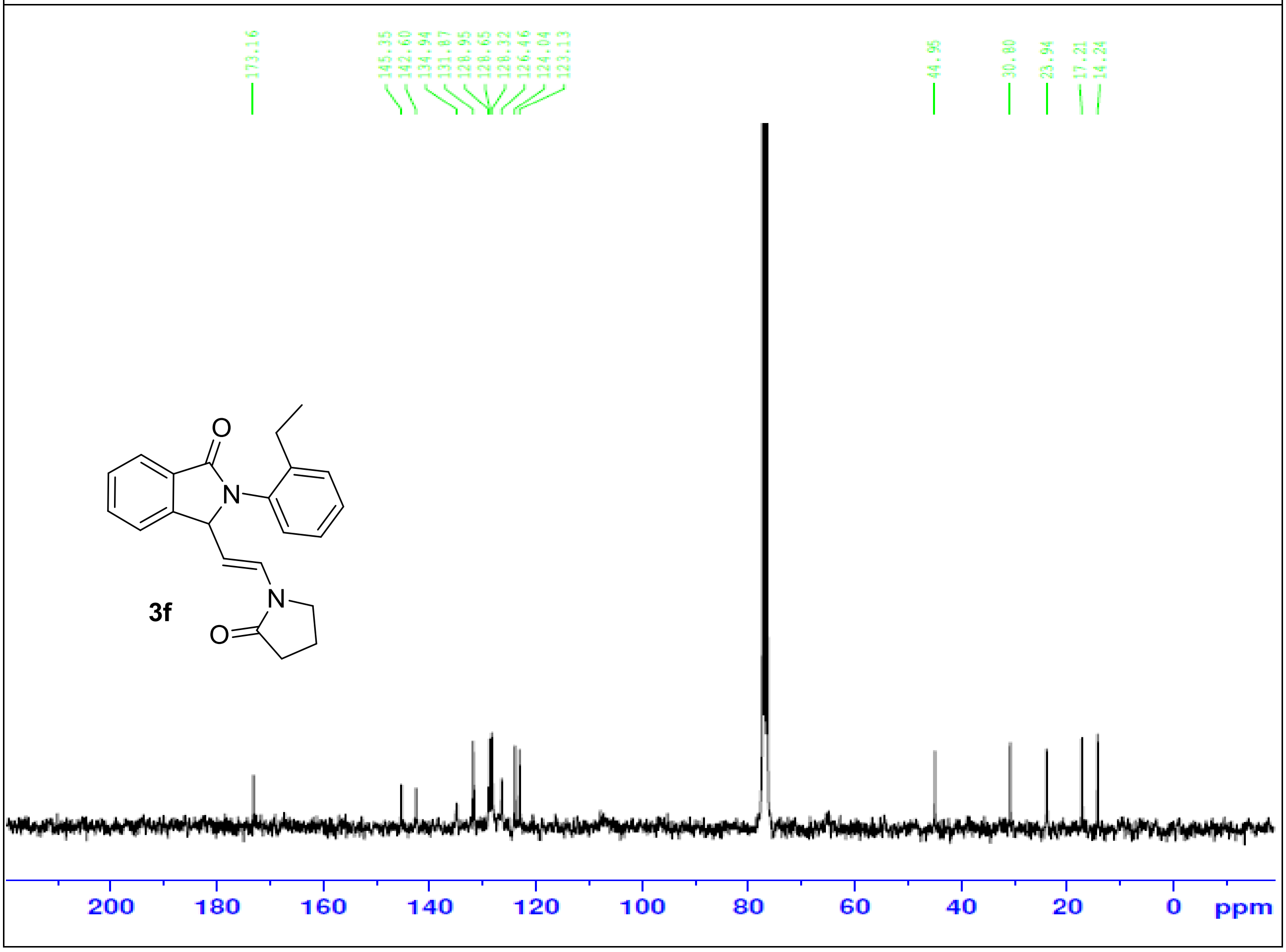
173.16
145.35 142.60 134.94 131.87 128.95 128.65 128.32 126.46 124.04 123.13
44.95 30.80 23.94 17.21 14.24
3f
ppm

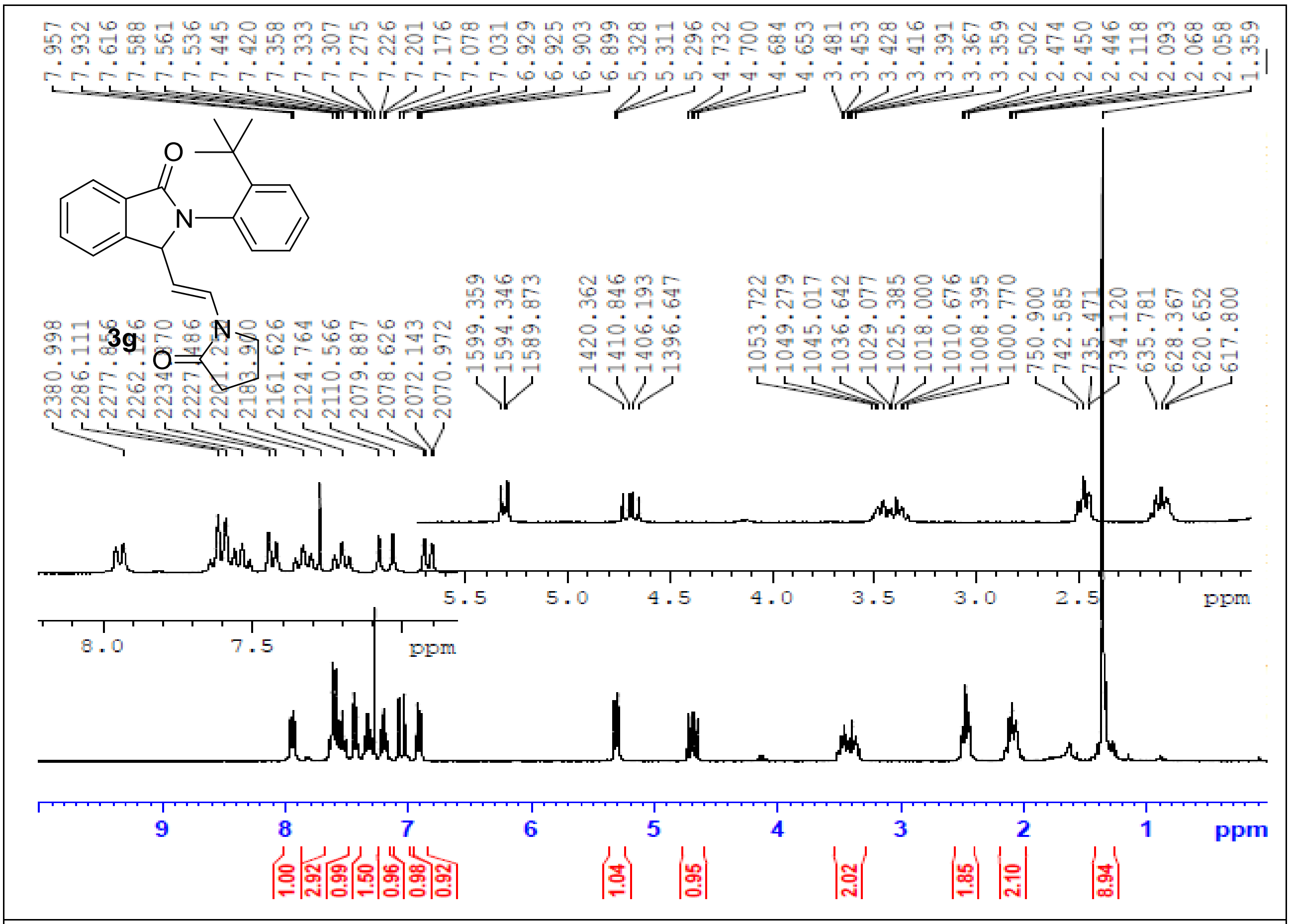
7.957 7.932 7.616 7.588 7.561 7.536 7.445 7.420 7.358 7.333 7.307 7.275 7.226 7.201 7.176 7.078 7.031 6.929 6.925 6.903 6.899 5.328 5.311 5.296 4.732 4.700 4.684 4.653 3.481 3.453 3.428 3.416 3.391 3.367 3.359 2.502 2.474 2.450 2.446 2.118 2.093 2.068 2.058 1.359
O
N
3g
2380.998 2286.111 2227.486 2161.626 2124.764 2110.566 2079.887 2078.626 2072.143 2070.972
1599.359 1594.346 1589.873
1420.362 1410.846 1406.193 1396.647
1053.722 1049.279 1045.017 1036.642 1029.077 1025.385 1018.000 1010.676 1008.395 1000.770
750.900 742.585 735.471 734.120
635.781 628.367 620.652 617.800
ppm
1.00 2.92 0.99 1.50 0.96 0.98 0.92 1.04 0.95 2.02 1.85 2.10 8.94

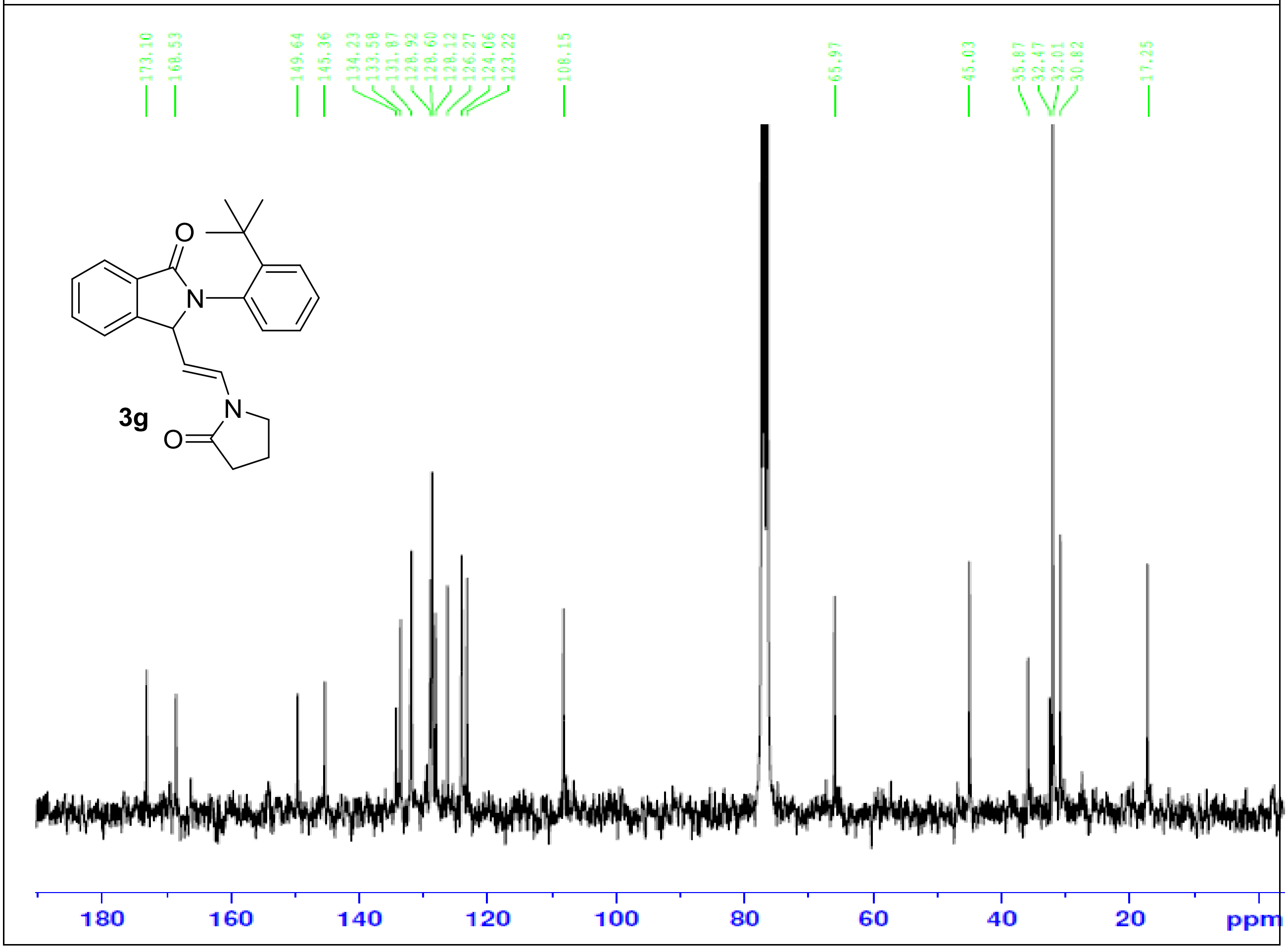
173.10 168.53 149.64 145.36 134.23 133.58 131.87 128.92 128.60 128.12 126.27 124.06 123.22 108.15 65.97 45.03 35.87 32.47 32.01 30.82 17.25
O
N
3g
180 160 140 120 100 80 60 40 20 ppm

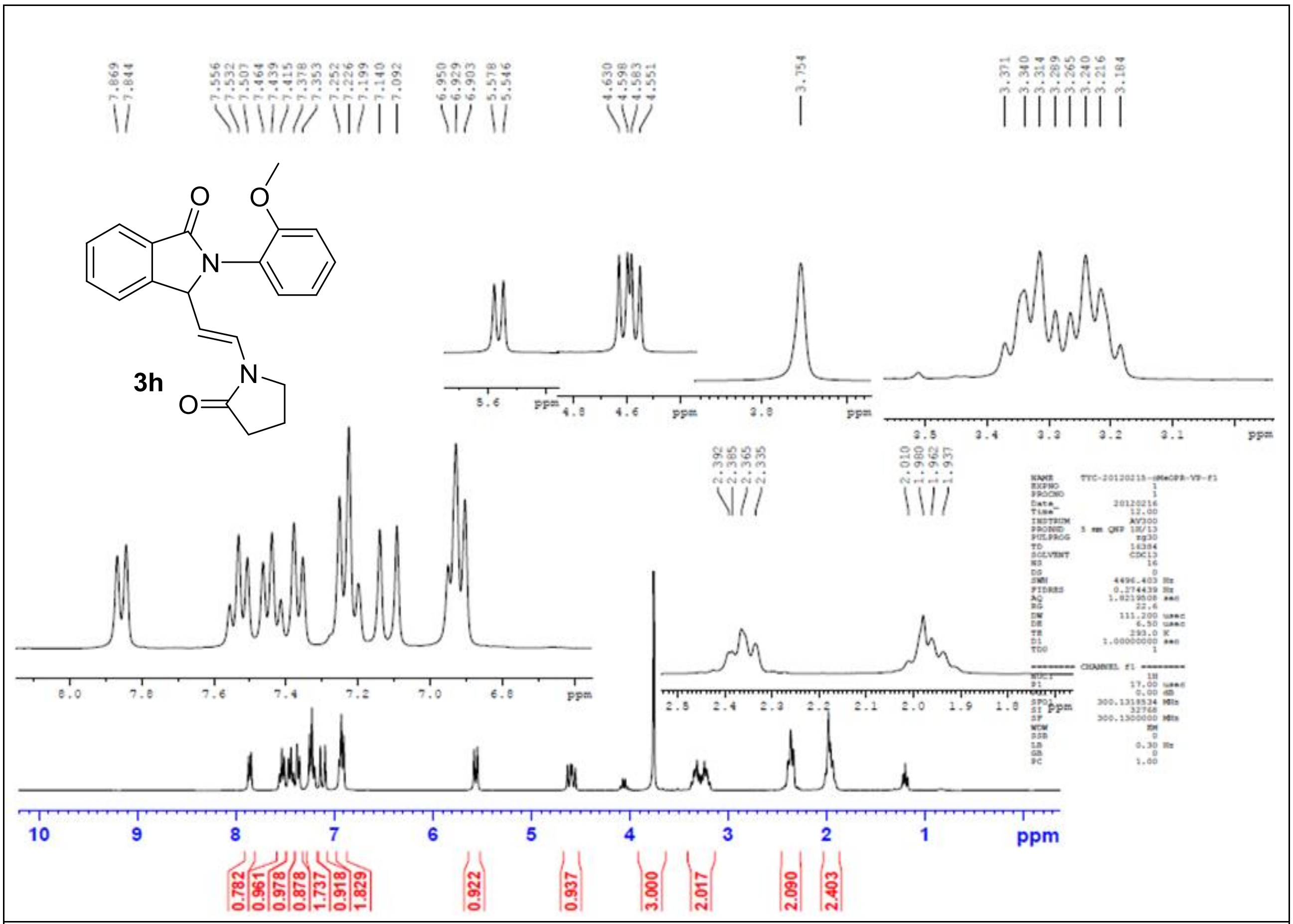
3h

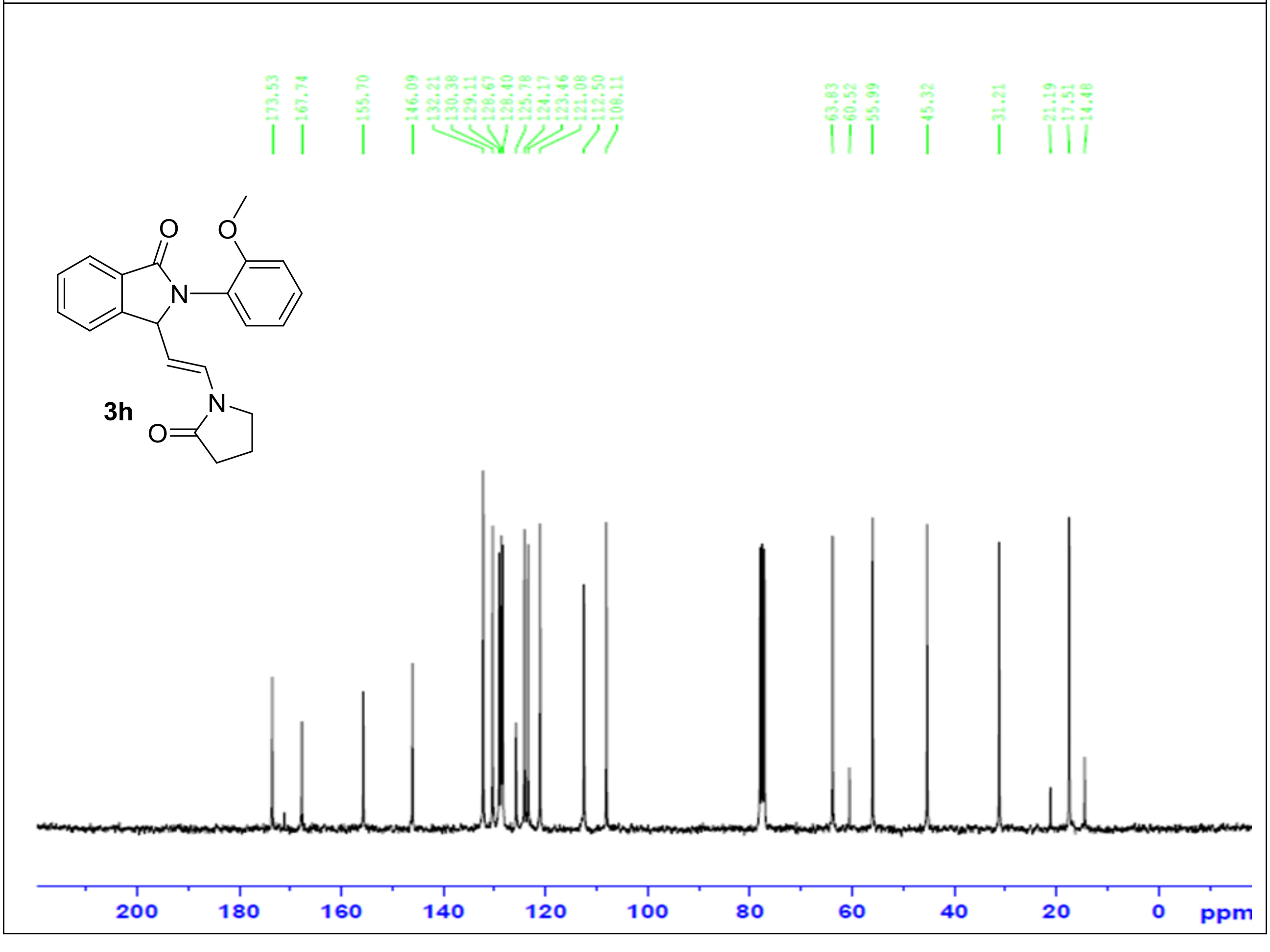
3h

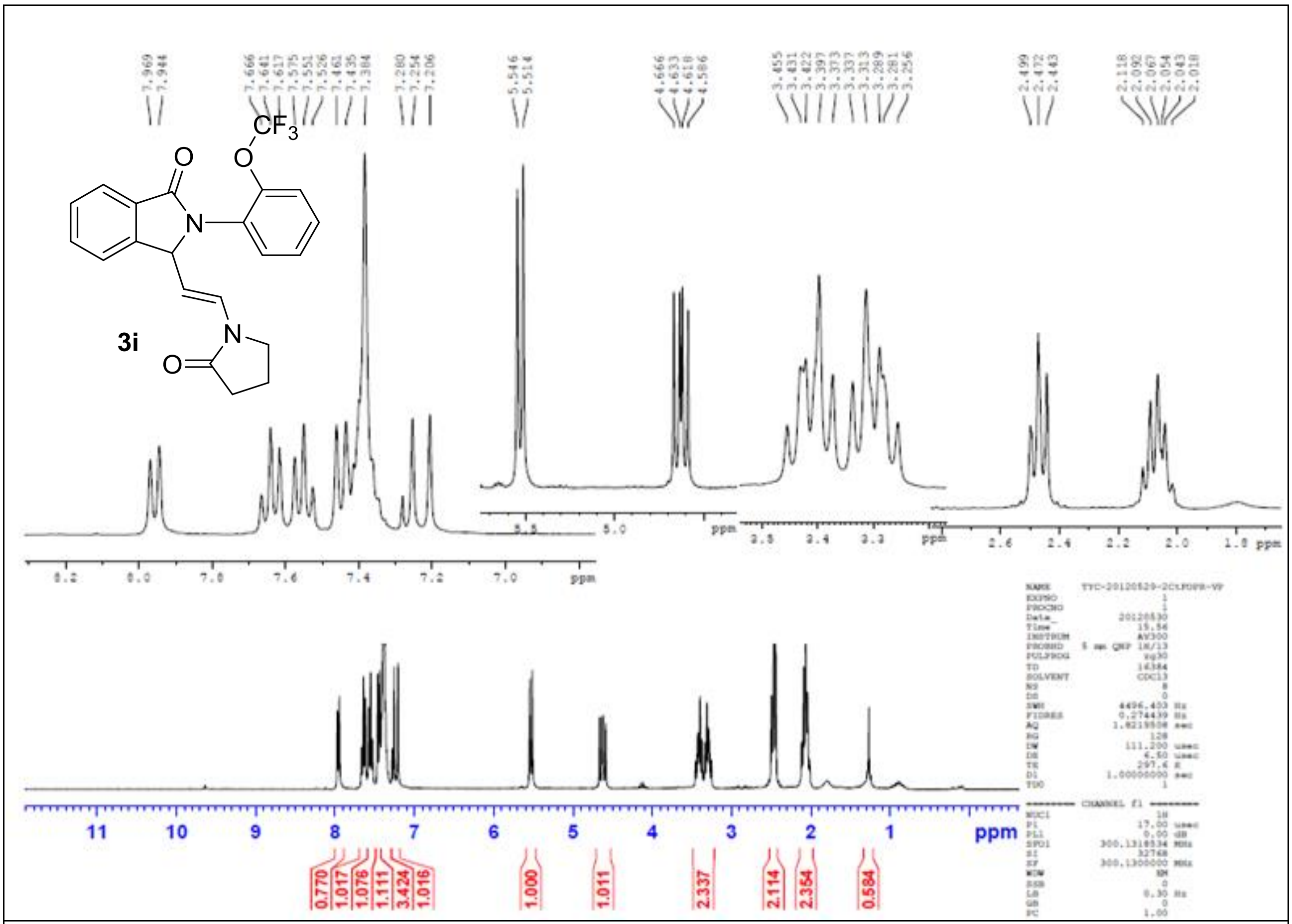
3i
ppm

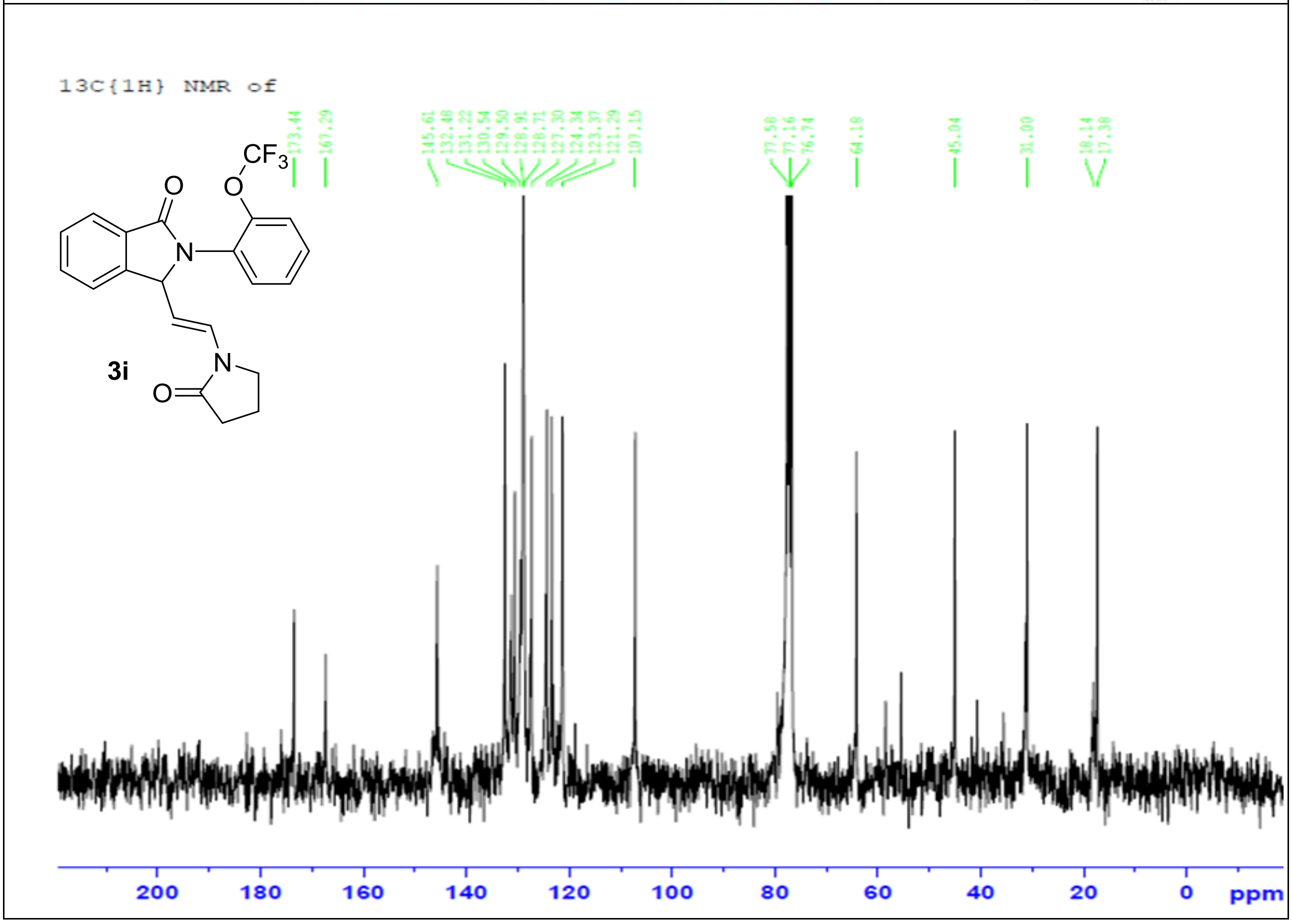
13C{1H} NMR of
173.44
167.29
145.61
132.48
131.22
130.54
129.50
128.91
128.71
127.30
124.34
123.37
121.29
107.15
77.58
77.16
76.74
64.18
45.04
31.00
18.14
17.38
3i
ppm

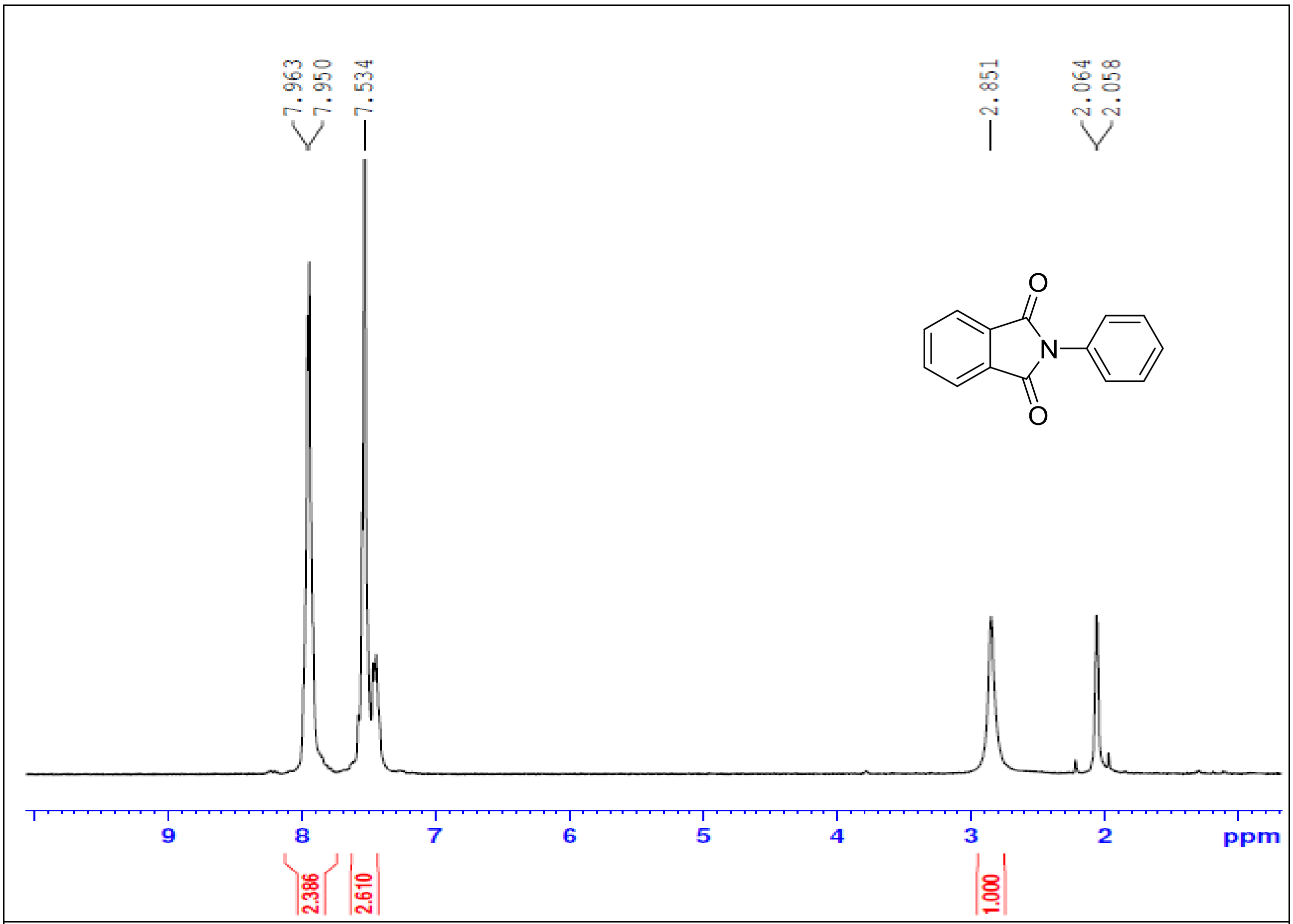
7.963
7.950
7.534
2.851
2.064
2.058
O
N
O
9
8
7
6
5
4
3
2
ppm
2.386
2.610
1.000

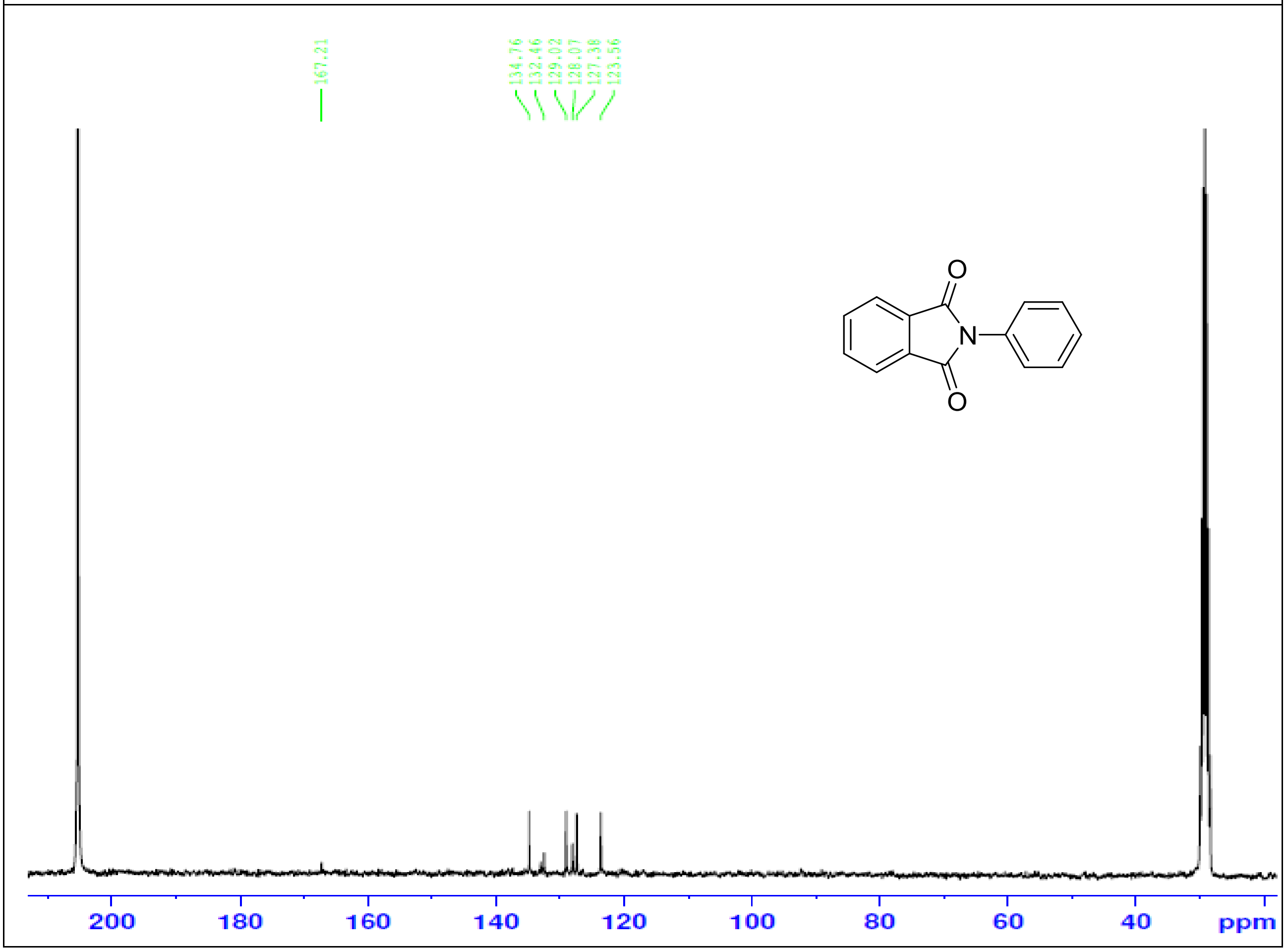
167.21
134.76
132.46
129.02
128.07
127.38
123.56
O
N
O
200
180
160
140
120
100
80
60
40
ppm

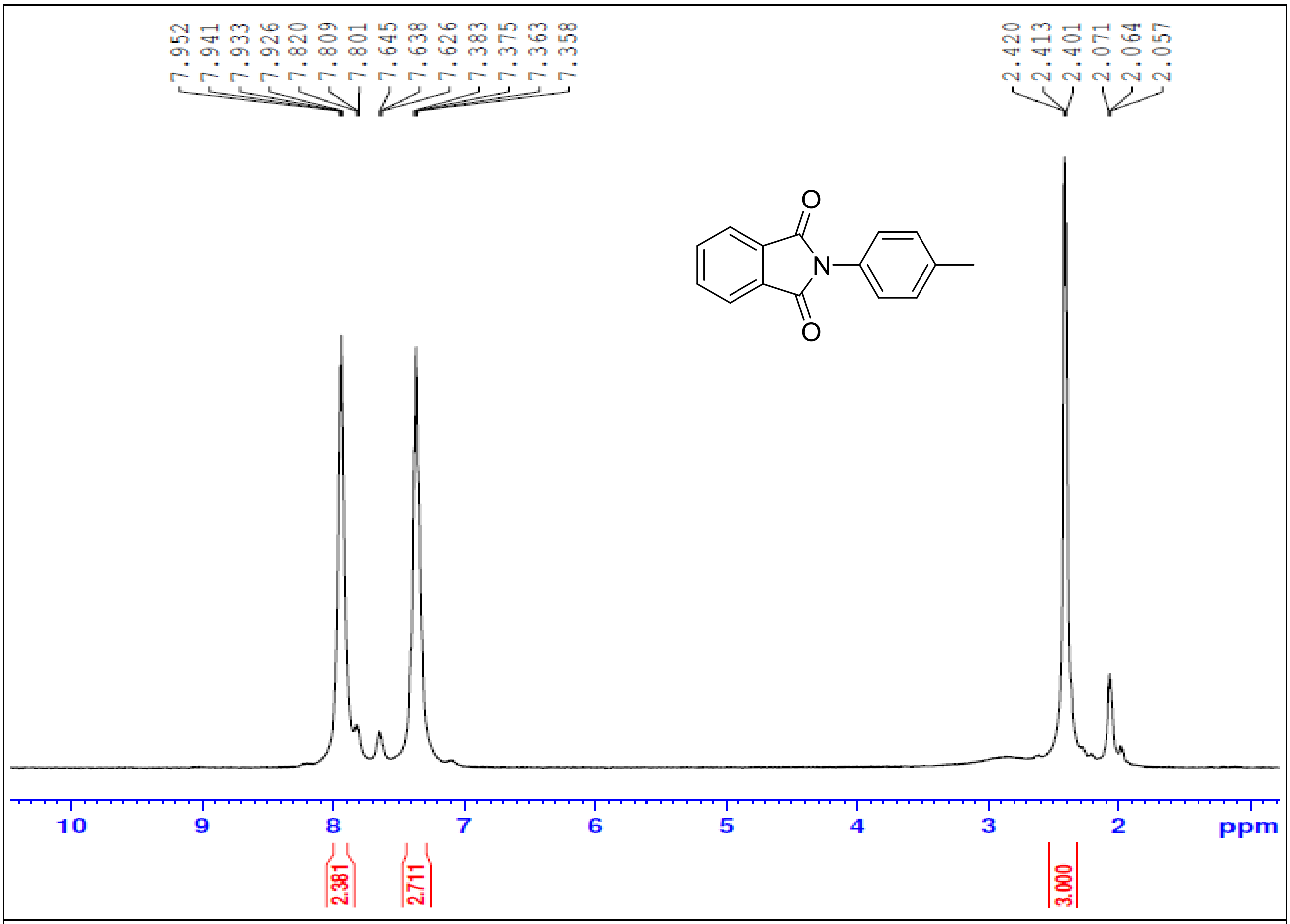
7.952
7.941
7.933
7.926
7.820
7.809
7.801
7.645
7.638
7.626
7.383
7.375
7.363
7.358
2.420
2.413
2.401
2.071
2.064
2.057
O
N
O
10
9
8
7
6
5
4
3
2
ppm
2.381
2.711
3.000

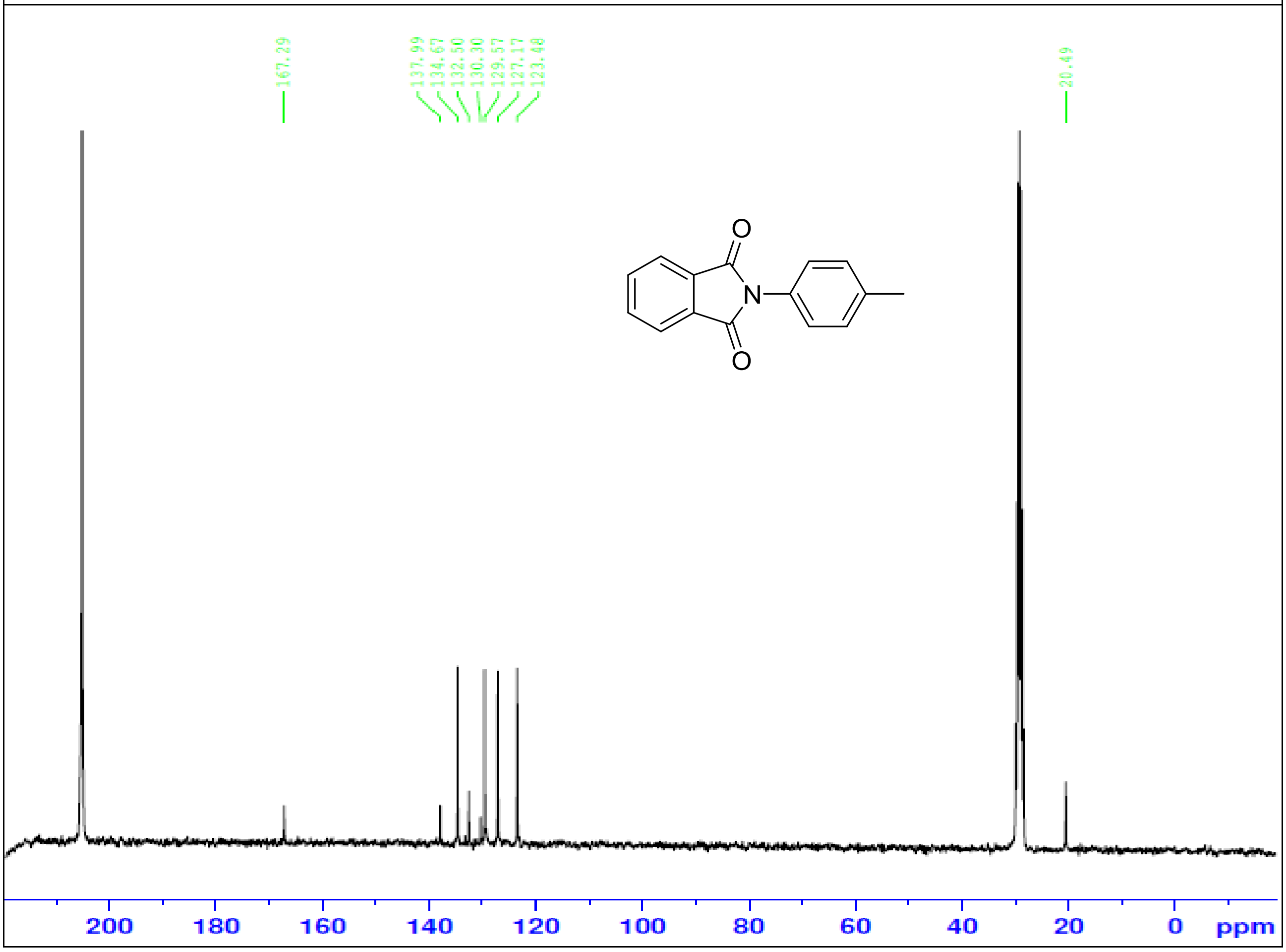
167.29
137.99
134.67
132.50
130.30
129.57
127.17
123.48
20.49
O
N
O
200
180
160
140
120
100
80
60
40
20
0
ppm

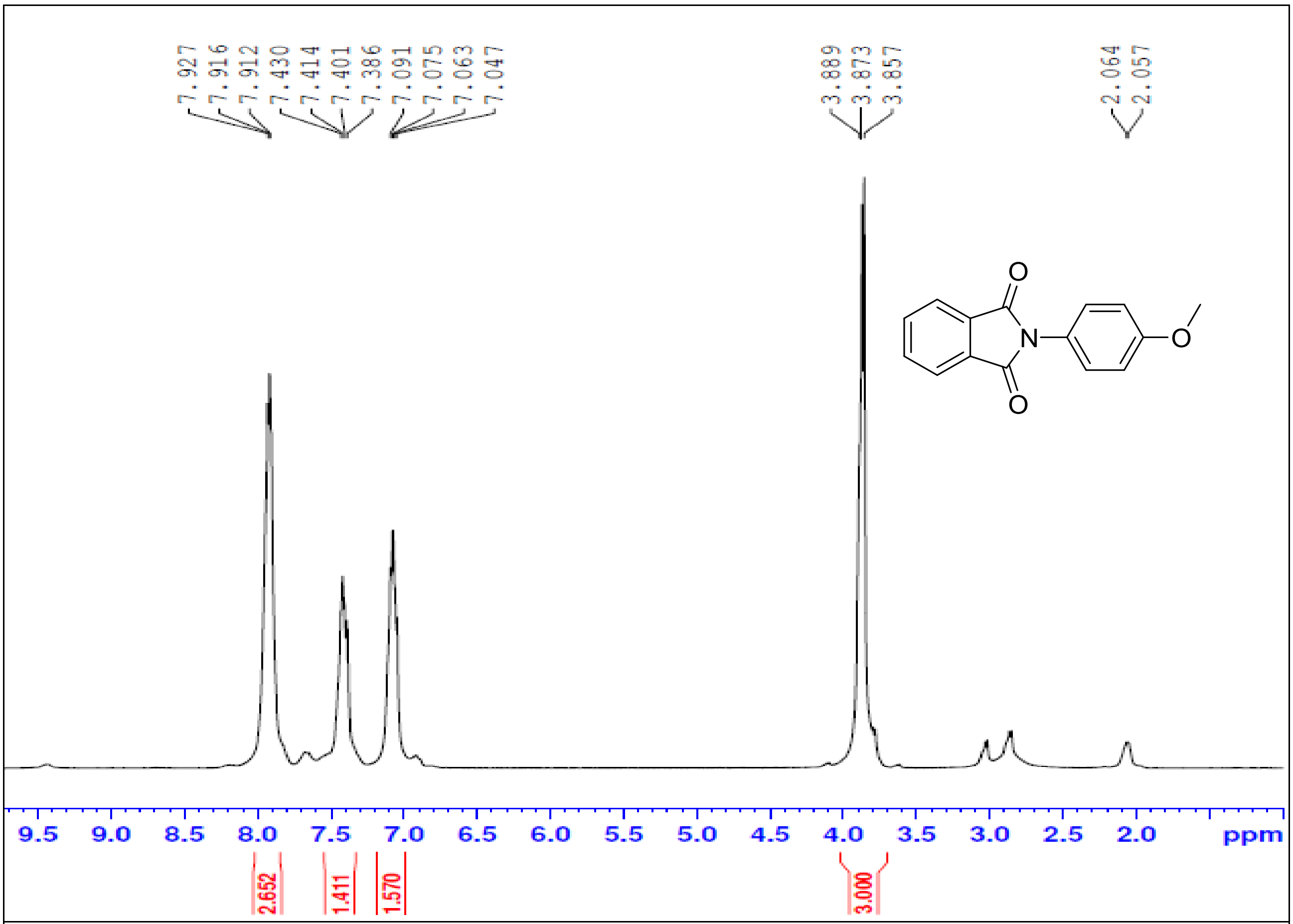
7.927
7.916
7.912
7.430
7.414
7.401
7.386
7.091
7.075
7.063
7.047
3.889
3.873
3.857
2.064
2.057
O
N
O
O
9.5
9.0
8.5
8.0
7.5
7.0
6.5
6.0
5.5
5.0
4.5
4.0
3.5
3.0
2.5
2.0
ppm
2.652
1.411
1.570
3.000

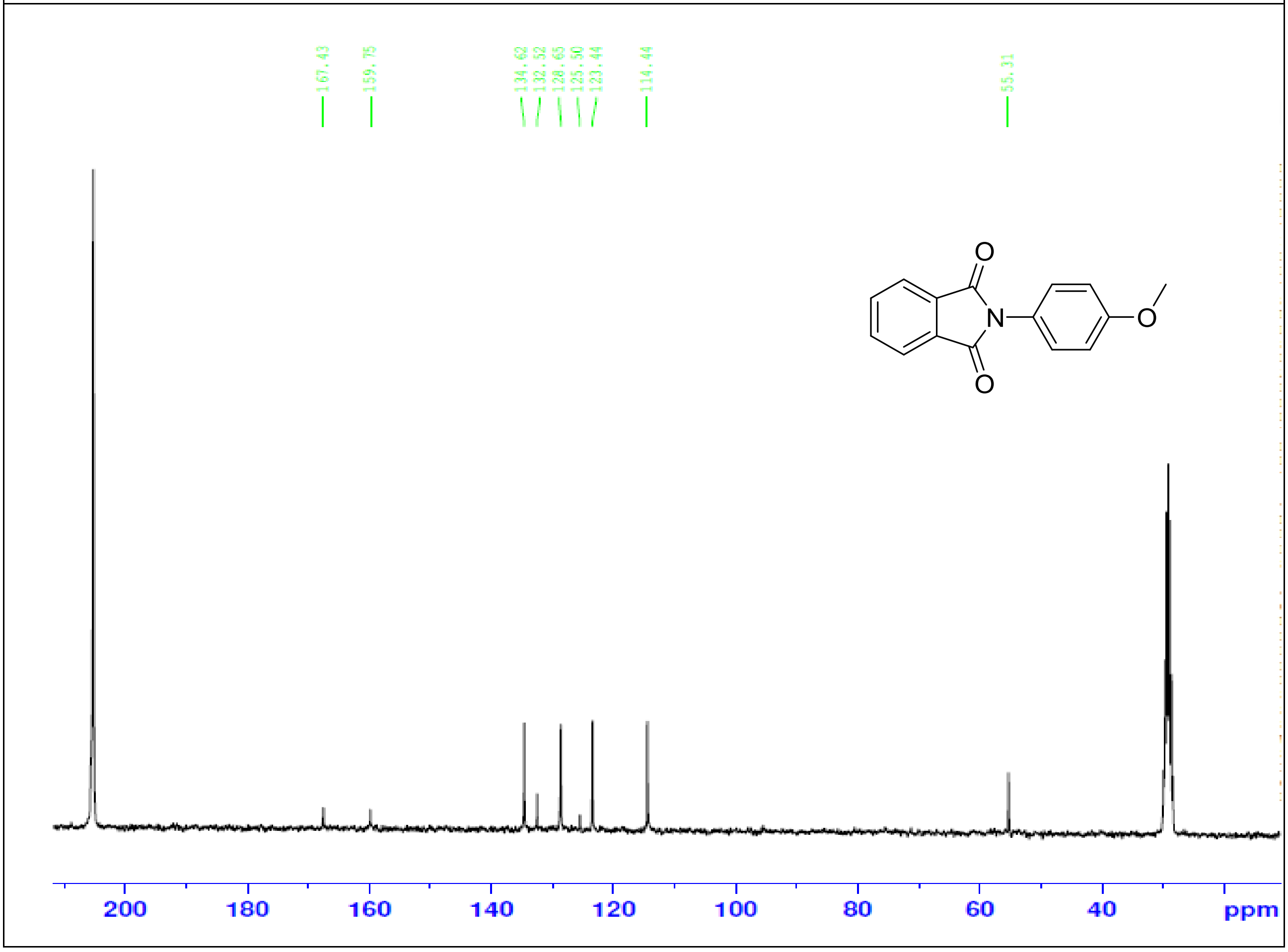
167.43
159.75
134.62
132.52
128.65
125.50
123.44
114.44
55.31
O
N
O
O
200
180
160
140
120
100
80
60
40
ppm

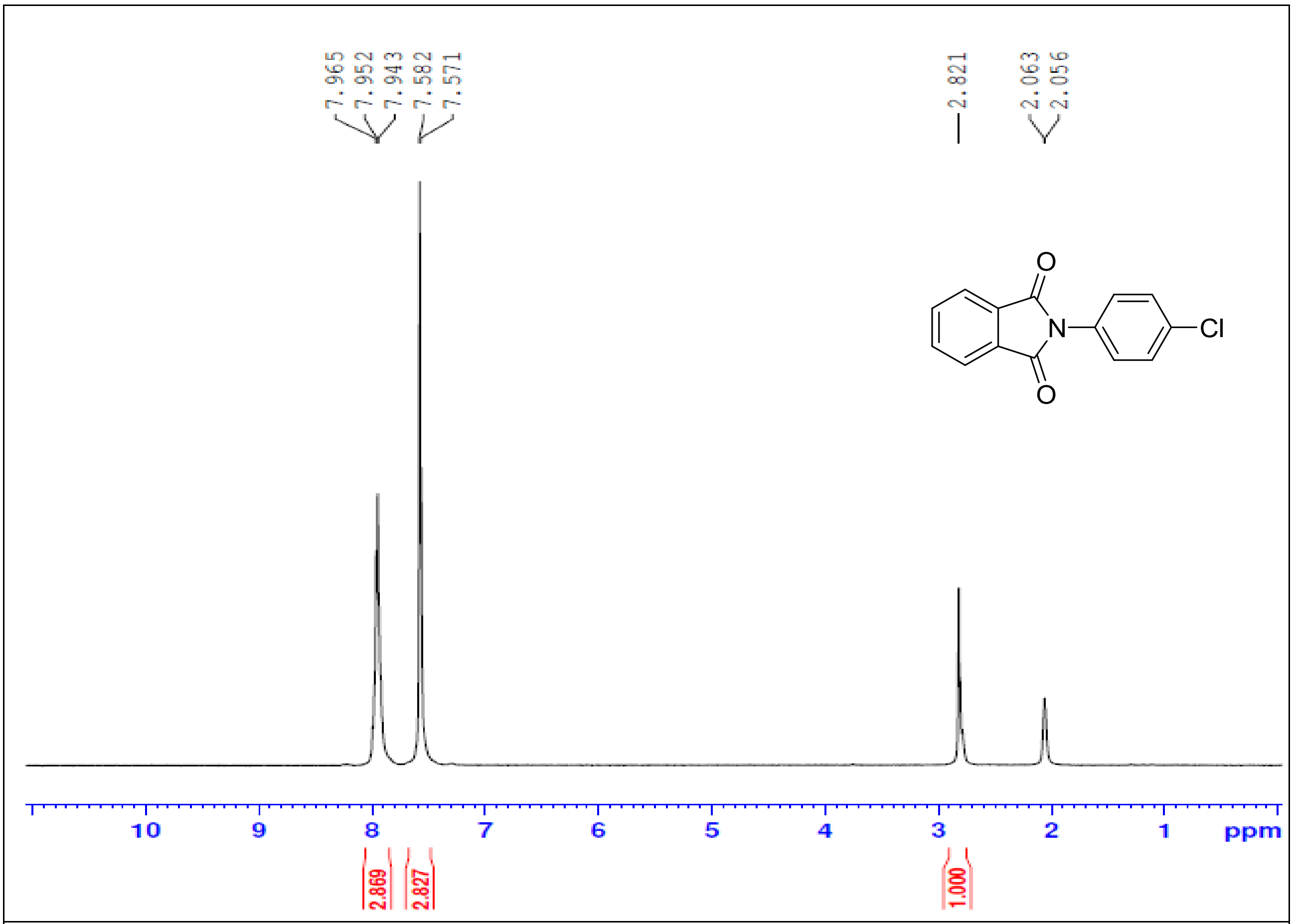
7.965
7.952
7.943
7.582
7.571
2.821
2.063
2.056
O
N
Cl
O
10
9
8
7
6
5
4
3
2
1
ppm
2.869
2.827
1.000

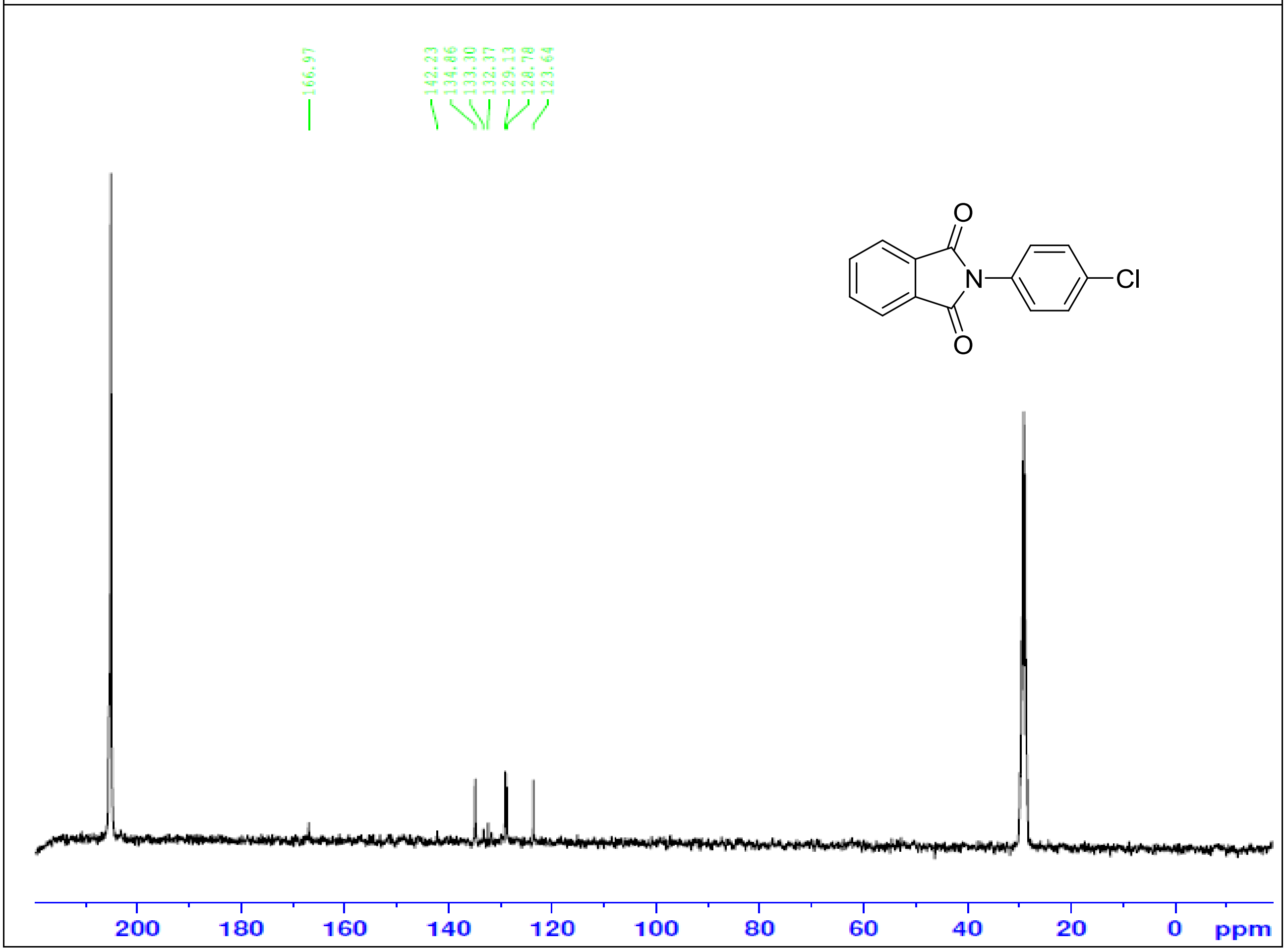
166.97
142.23
134.86
133.30
132.37
129.13
128.78
123.64
O
N
Cl
O
200
180
160
140
120
100
80
60
40
20
0
ppm

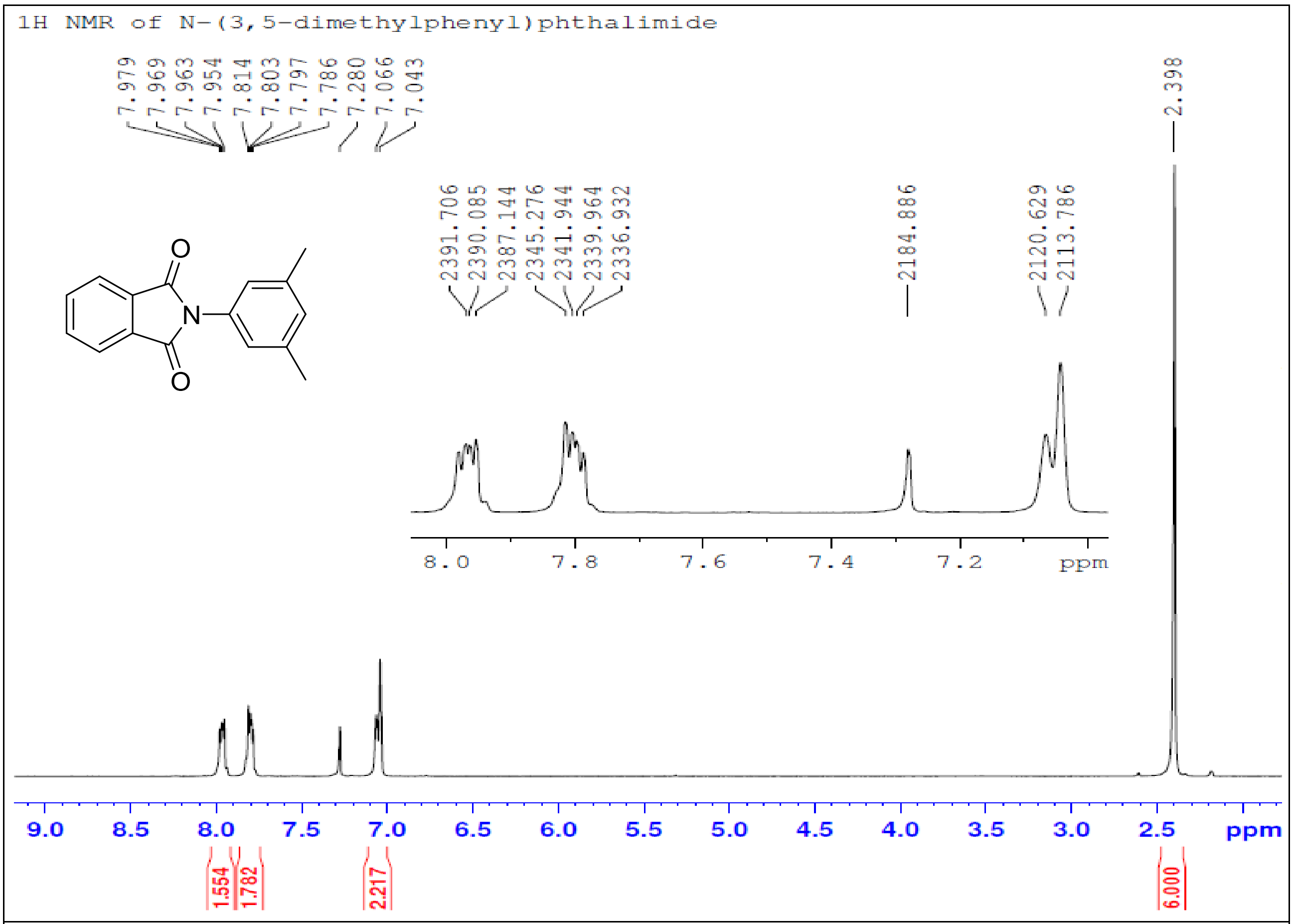
1H NMR of N-(3,5-dimethylphenyl)phthalimide
7.979
7.969
7.963
7.954
7.814
7.803
7.797
7.786
7.280
7.066
7.043
2.398
2391.706
2390.085
2387.144
2345.276
2341.944
2339.964
2336.932
2184.886
2120.629
2113.786
8.0
7.8
7.6
7.4
7.2
ppm
O
N
O
9.0
8.5
8.0
7.5
7.0
6.5
6.0
5.5
5.0
4.5
4.0
3.5
3.0
2.5
ppm
1.554
1.782
2.217
6.000

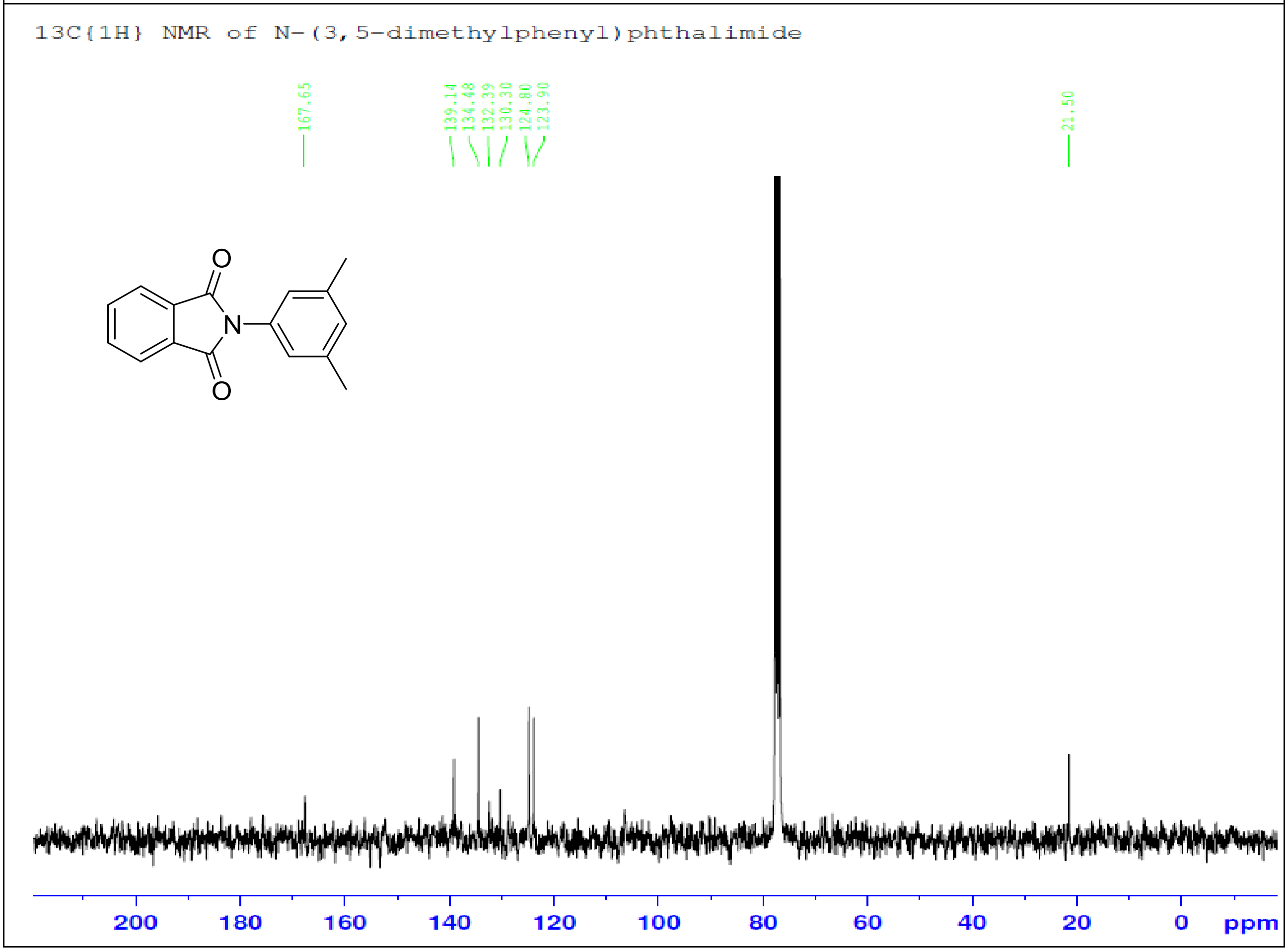
13C{1H} NMR of N-(3,5-dimethylphenyl)phthalimide
167.65
139.14
134.48
132.39
130.30
124.80
123.90
21.50
O
N
O
200
180
160
140
120
100
80
60
40
20
0
ppm

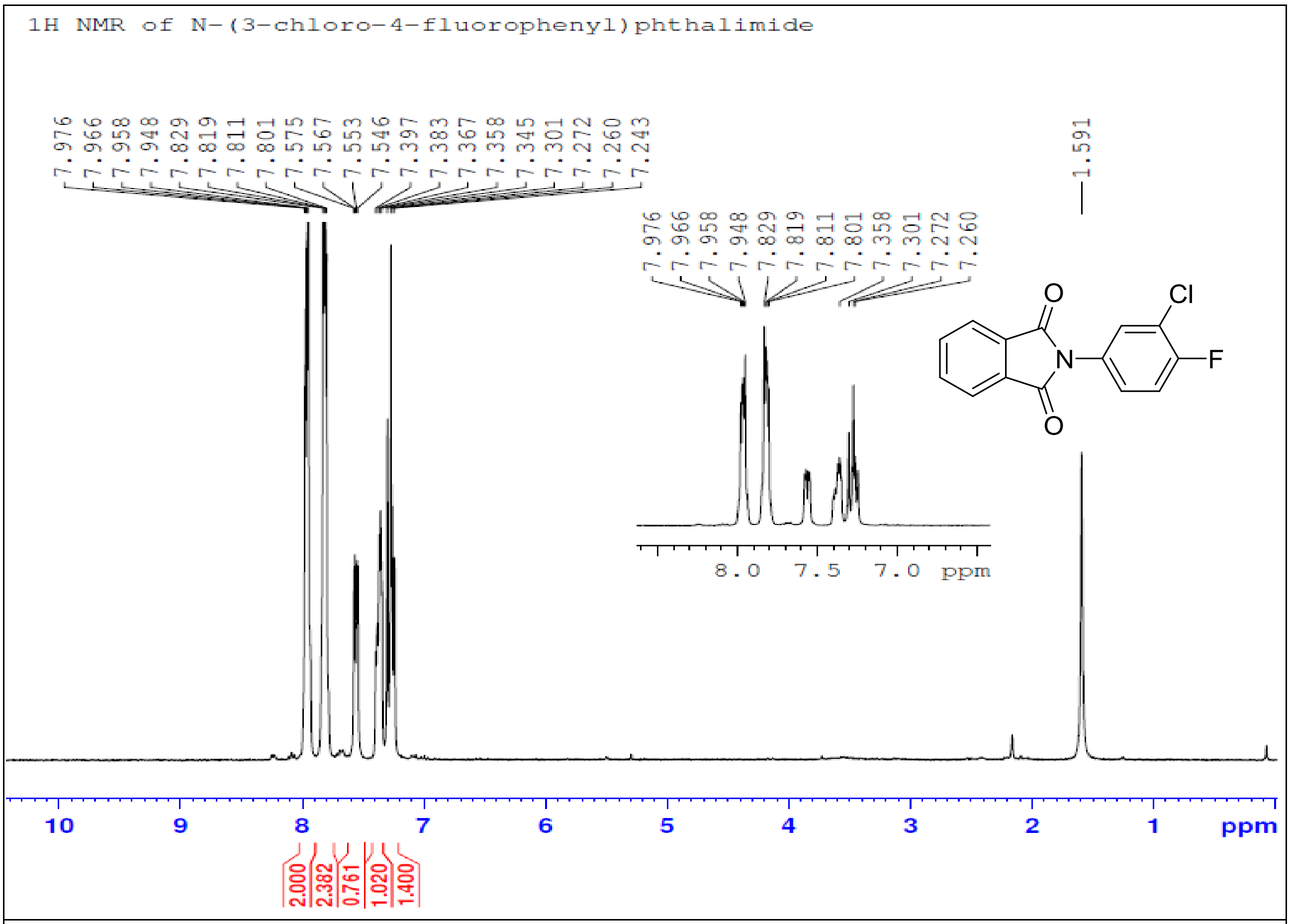
1H NMR of N-(3-chloro-4-fluorophenyl)phthalimide
7.976 7.966 7.958 7.948 7.829 7.819 7.811 7.801 7.575 7.567 7.553 7.546 7.397 7.383 7.367 7.358 7.345 7.301 7.272 7.260 7.243
1.591
7.976 7.966 7.958 7.948 7.829 7.819 7.811 7.801 7.358 7.301 7.272 7.260
8.0 7.5 7.0 ppm
O
Cl
N
F
O
10 9 8 7 6 5 4 3 2 1 ppm
2.000 2.382 0.761 1.020 1.400

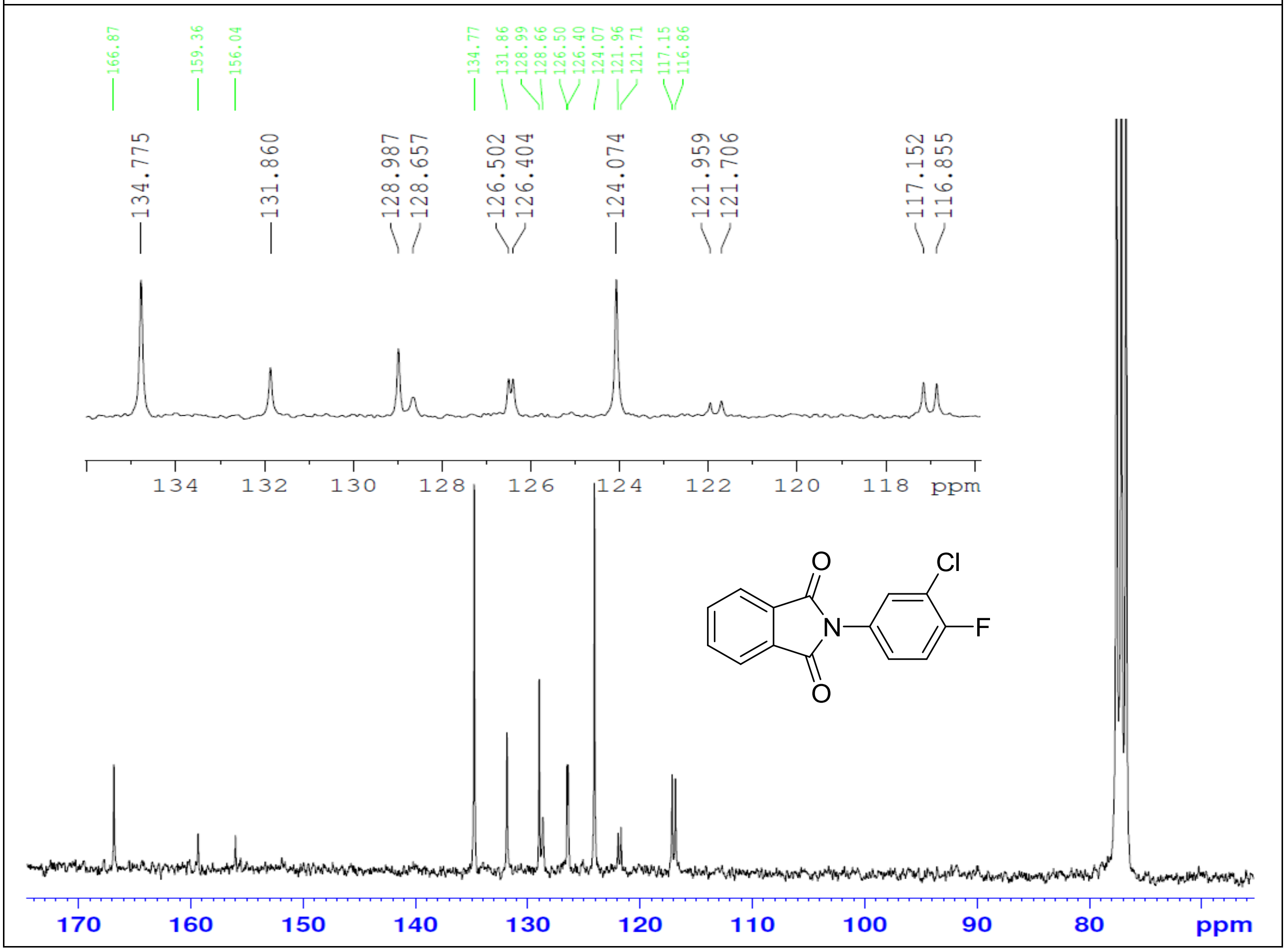
166.87 159.36 156.04 134.77 131.86 128.99 128.66 126.50 126.40 124.07 121.96 121.71 117.15 116.86
134.775 131.860 128.987 128.657 126.502 126.404 124.074 121.959 121.706 117.152 116.855
134 132 130 128 126 124 122 120 118 ppm
O
Cl
N
F
O
170 160 150 140 130 120 110 100 90 80 ppm

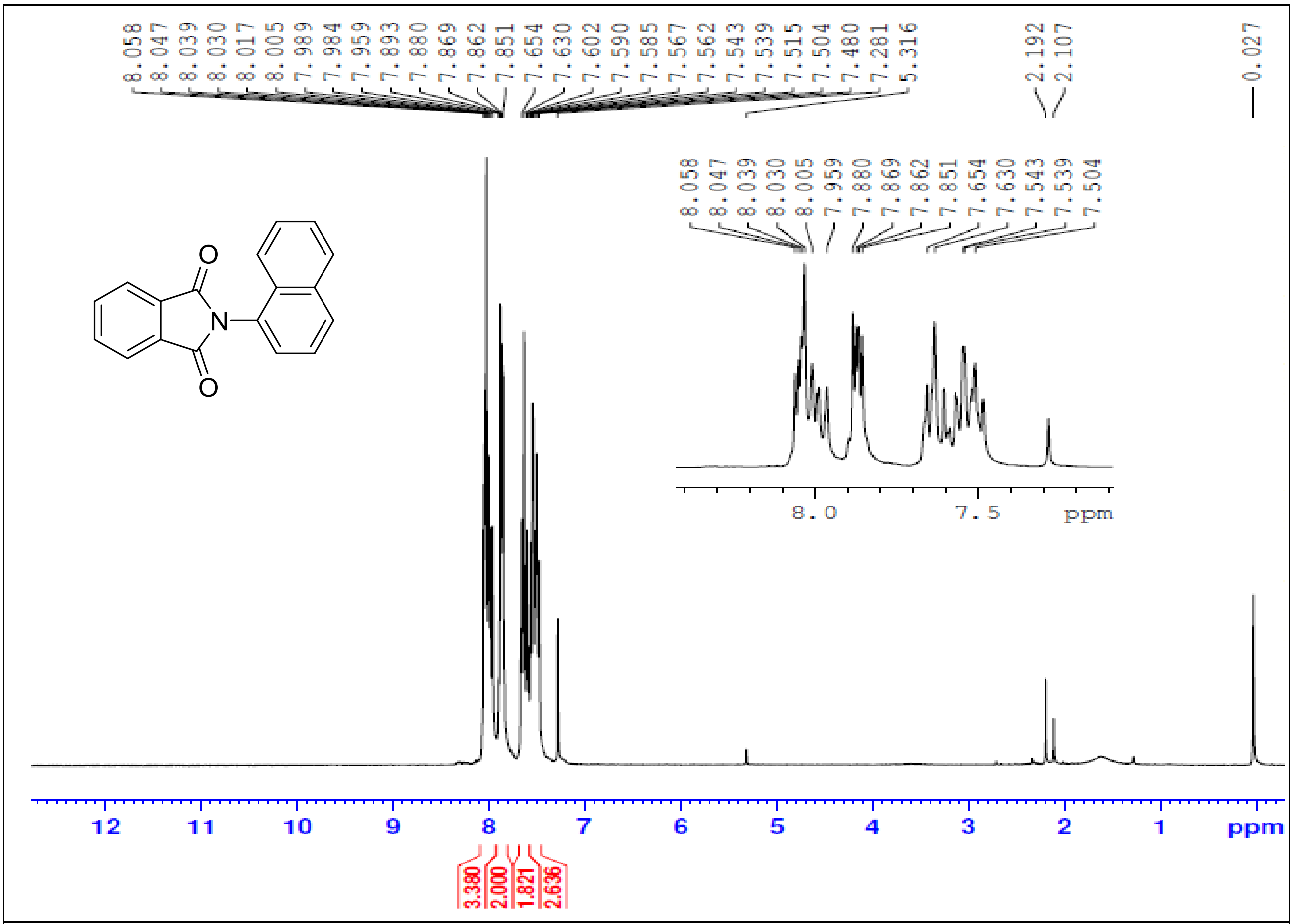
8.058
8.047
8.039
8.030
8.017
8.005
7.989
7.984
7.959
7.893
7.880
7.869
7.862
7.851
7.654
7.630
7.602
7.590
7.585
7.567
7.562
7.543
7.539
7.515
7.504
7.480
7.281
5.316
2.192
2.107
0.027
O
N
O
8.0
7.5
ppm
12
11
10
9
8
7
6
5
4
3
2
1
ppm
3.380
2.000
1.821
2.636

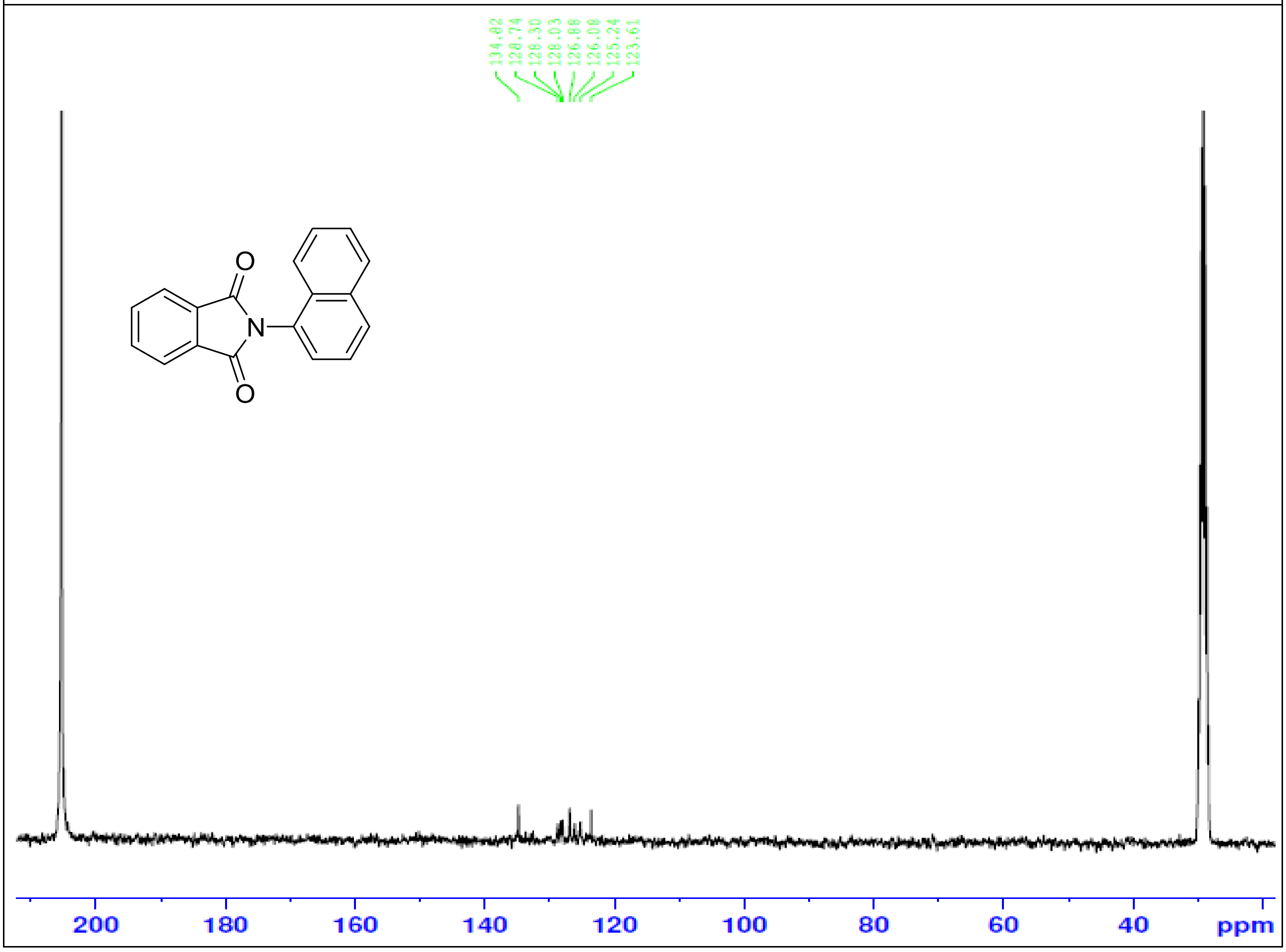
134.82
128.74
128.30
128.03
126.88
126.08
125.24
123.61
O
N
O
200
180
160
140
120
100
80
60
40
ppm

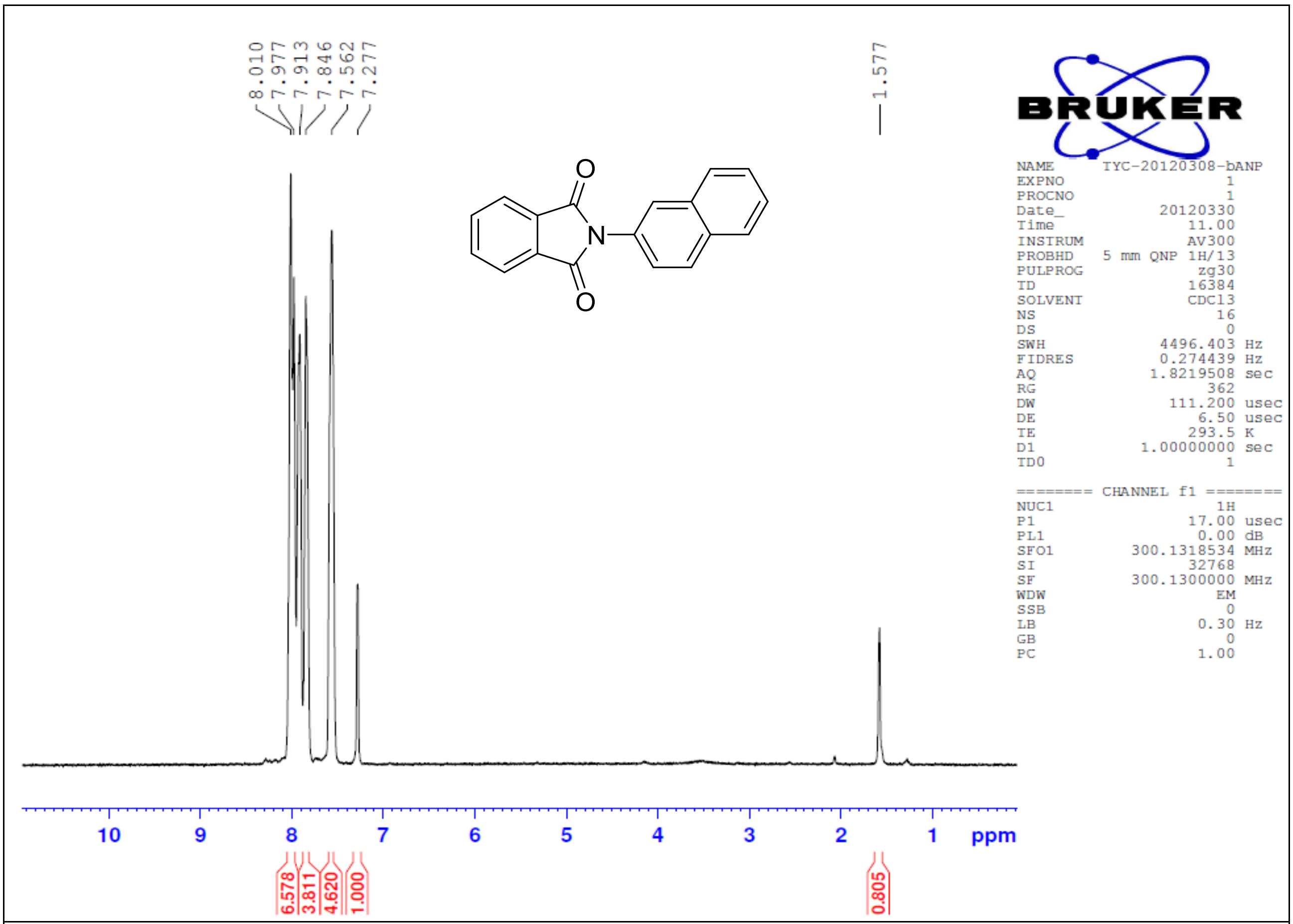
8.010
7.977
7.913
7.846
7.562
7.277
1.577
O
N
O
BRUKER
NAME TYC-20120308-bANP
EXPNO 1
PROCNO 1
Date_ 20120330
Time 11.00
INSTRUM AV300
PROBHD 5 mm QNP 1H/13
PULPROG zg30
TD 16384
SOLVENT CDCl3
NS 16
DS 0
SWH 4496.403 Hz
FIDRES 0.274439 Hz
AQ 1.8219508 sec
RG 362
DW 111.200 usec
DE 6.50 usec
TE 293.5 K
D1 1.00000000 sec
TD0 1
======== CHANNEL f1 ========
NUC1 1H
P1 17.00 usec
PL1 0.00 dB
SFO1 300.1318534 MHz
SI 32768
SF 300.1300000 MHz
WDW EM
SSB 0
LB 0.30 Hz
GB 0
PC 1.00
10 9 8 7 6 5 4 3 2 1 ppm
6.578
3.811
4.620
1.000
0.805

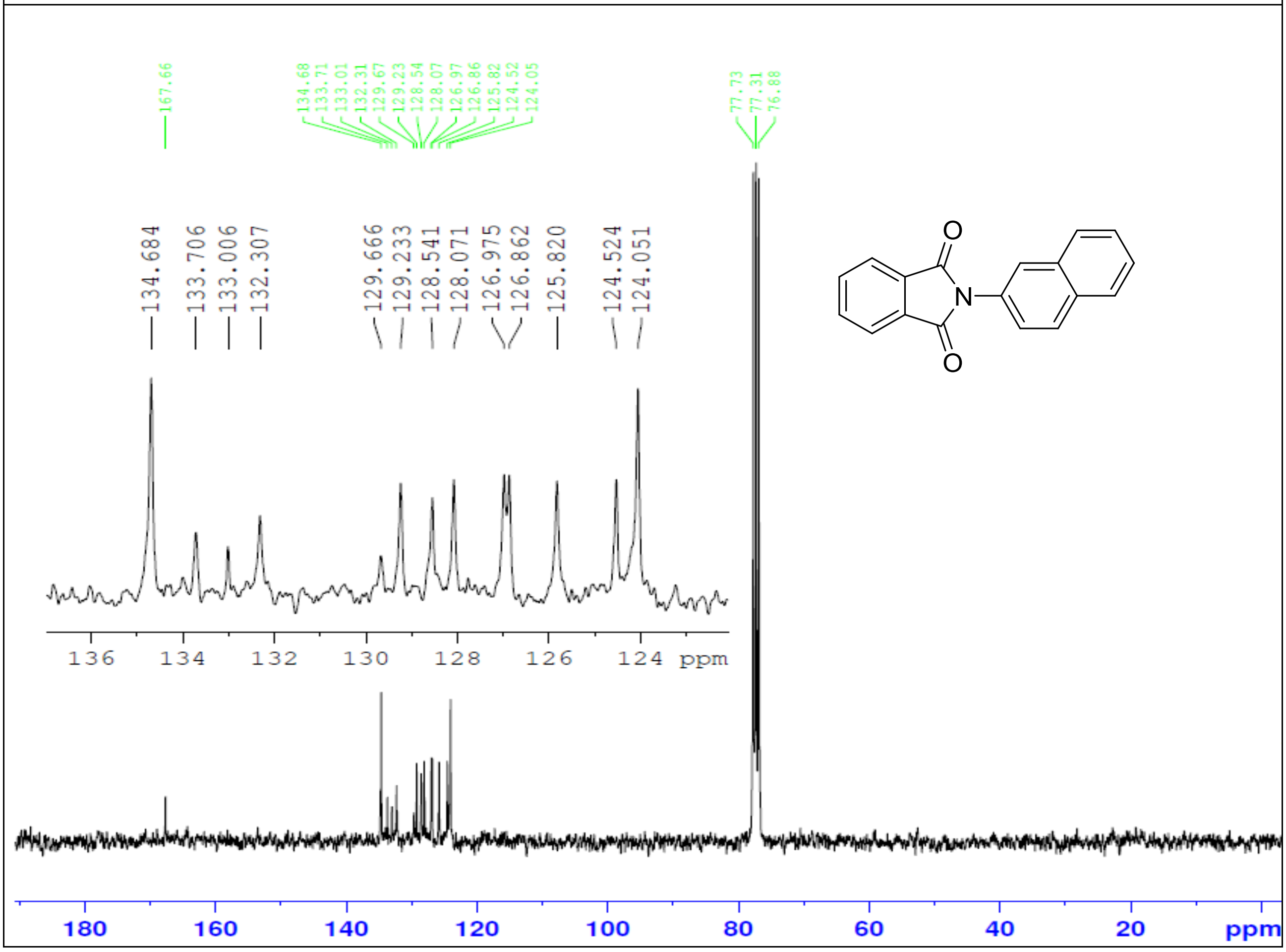
167.66
134.68
133.71
133.01
132.31
129.67
129.23
128.54
128.07
126.97
126.86
125.82
124.52
124.05
77.73
77.31
76.88
134.684
133.706
133.006
132.307
129.666
129.233
128.541
128.071
126.975
126.862
125.820
124.524
124.051
O
N
O
136 134 132 130 128 126 124 ppm
180 160 140 120 100 80 60 40 20 ppm

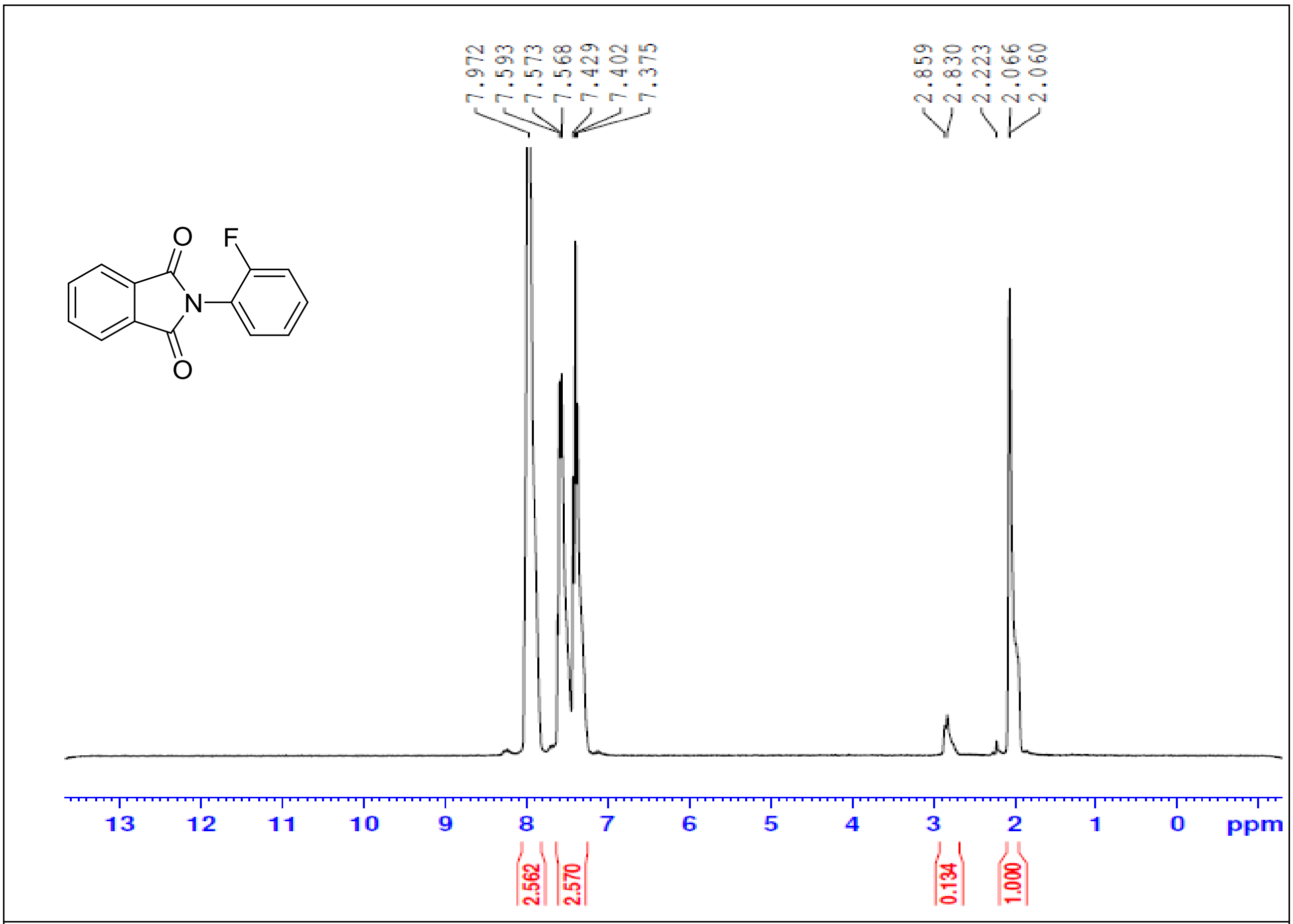
7.972
7.593
7.573
7.568
7.429
7.402
7.375
2.859
2.830
2.223
2.066
2.060
O
F
N
O
13 12 11 10 9 8 7 6 5 4 3 2 1 0 ppm
2.562
2.570
0.134
1.000

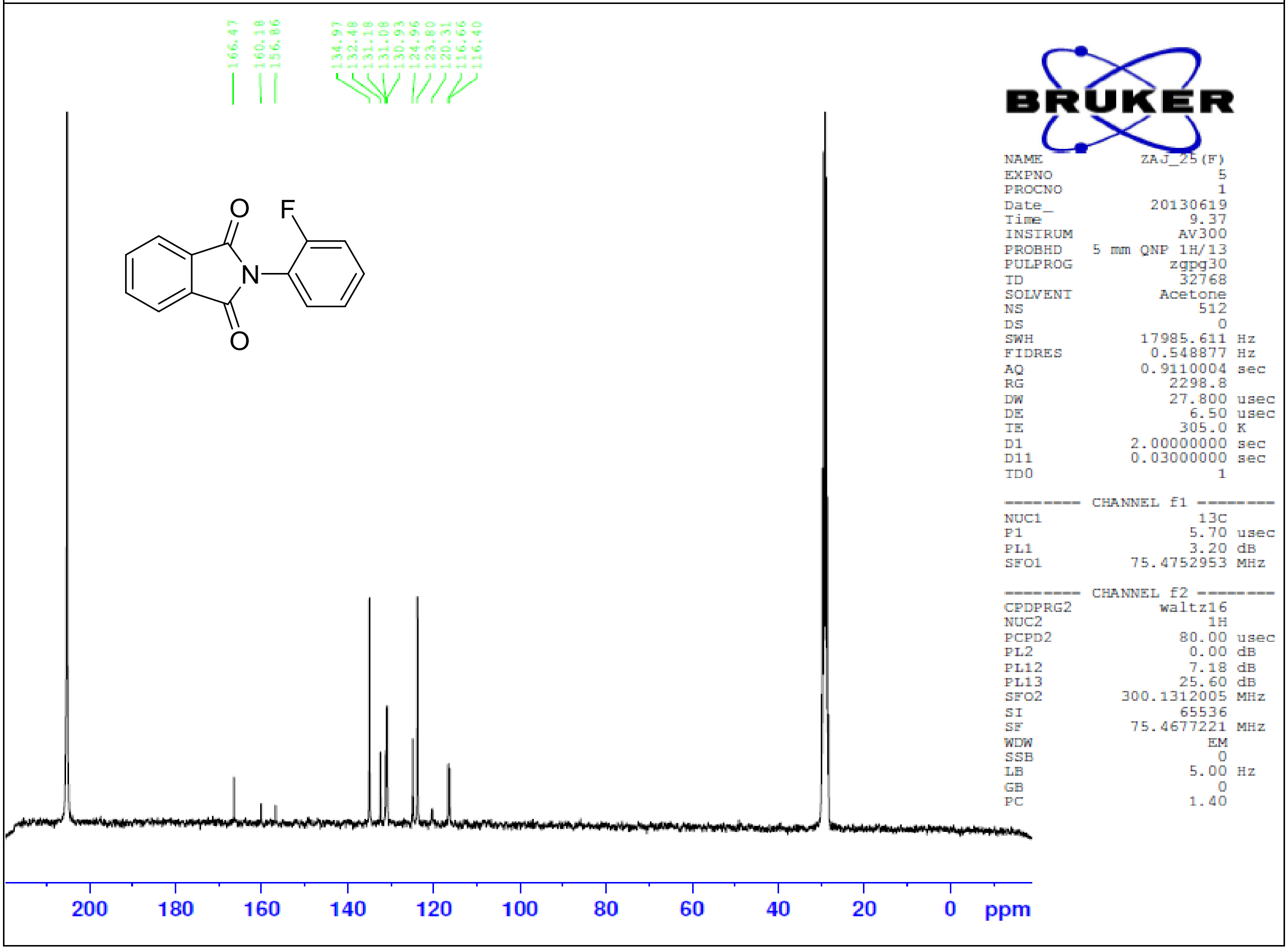
166.47
160.18
156.86
134.97
132.48
131.18
131.08
130.93
124.96
123.80
120.31
116.66
116.40
O
F
N
O
BRUKER
NAME ZAJ_25(F)
EXPNO 5
PROCNO 1
Date_ 20130619
Time 9.37
INSTRUM AV300
PROBHD 5 mm QNP 1H/13
PULPROG zgpg30
TD 32768
SOLVENT Acetone
NS 512
DS 0
SWH 17985.611 Hz
FIDRES 0.548877 Hz
AQ 0.9110004 sec
RG 2298.8
DW 27.800 usec
DE 6.50 usec
TE 305.0 K
D1 2.00000000 sec
D11 0.03000000 sec
TD0 1
======== CHANNEL f1 ========
NUC1 13C
P1 5.70 usec
PL1 3.20 dB
SFO1 75.4752953 MHz
======== CHANNEL f2 ========
CPDPRG2 waltz16
NUC2 1H
PCPD2 80.00 usec
PL2 0.00 dB
PL12 7.18 dB
PL13 25.60 dB
SFO2 300.1312005 MHz
SI 65536
SF 75.4677221 MHz
WDW EM
SSB 0
LB 5.00 Hz
GB 0
PC 1.40
200 180 160 140 120 100 80 60 40 20 0 ppm

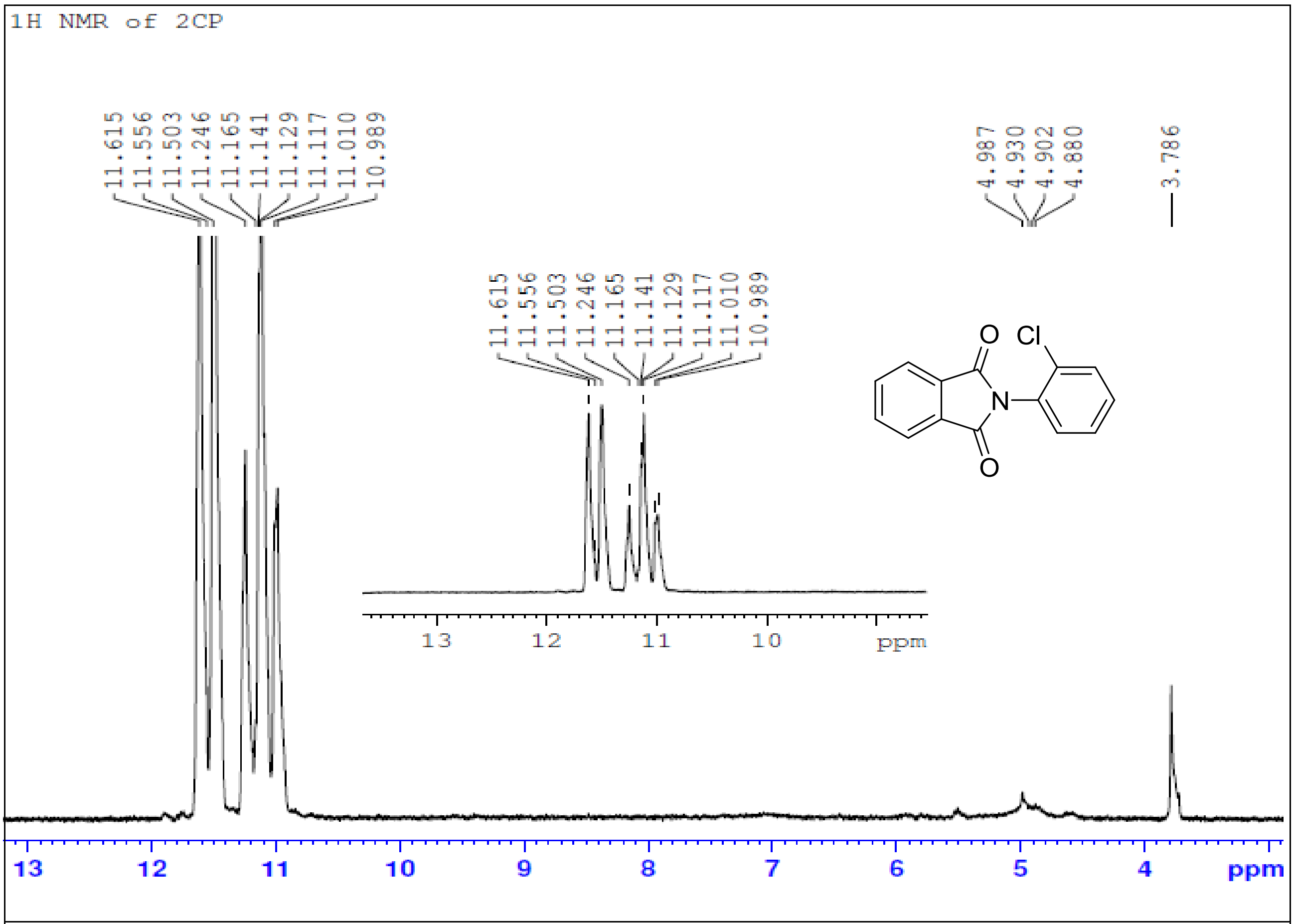
1H NMR of 2CP
11.615
11.556
11.503
11.246
11.165
11.141
11.129
11.117
11.010
10.989
4.987
4.930
4.902
4.880
3.786
O
Cl
N
O
13
12
11
10
9
8
7
6
5
4
ppm

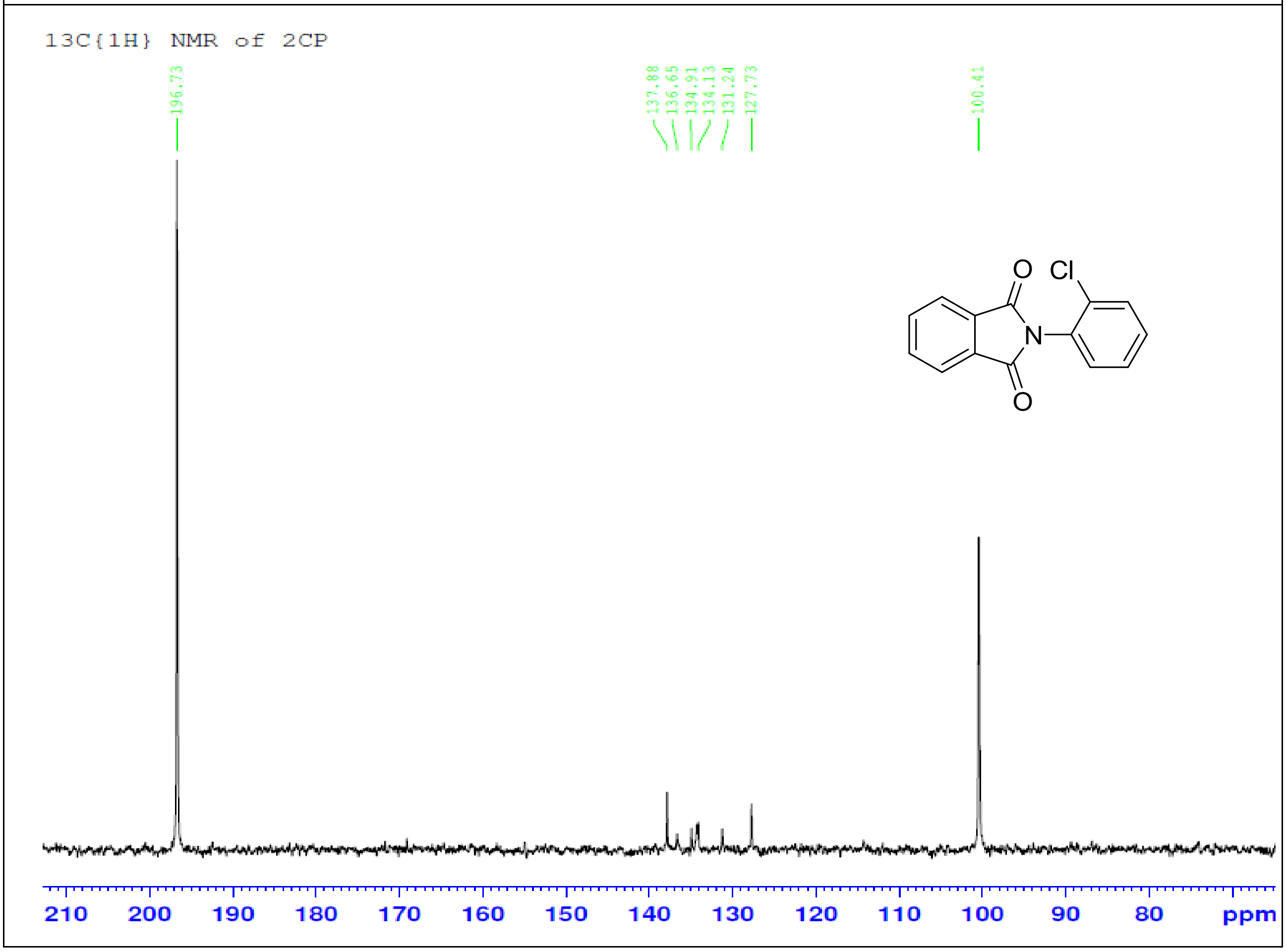
13C{1H} NMR of 2CP
196.73
137.88
136.65
134.91
134.13
131.24
127.73
100.41
O
Cl
N
O
210
200
190
180
170
160
150
140
130
120
110
100
90
80
ppm

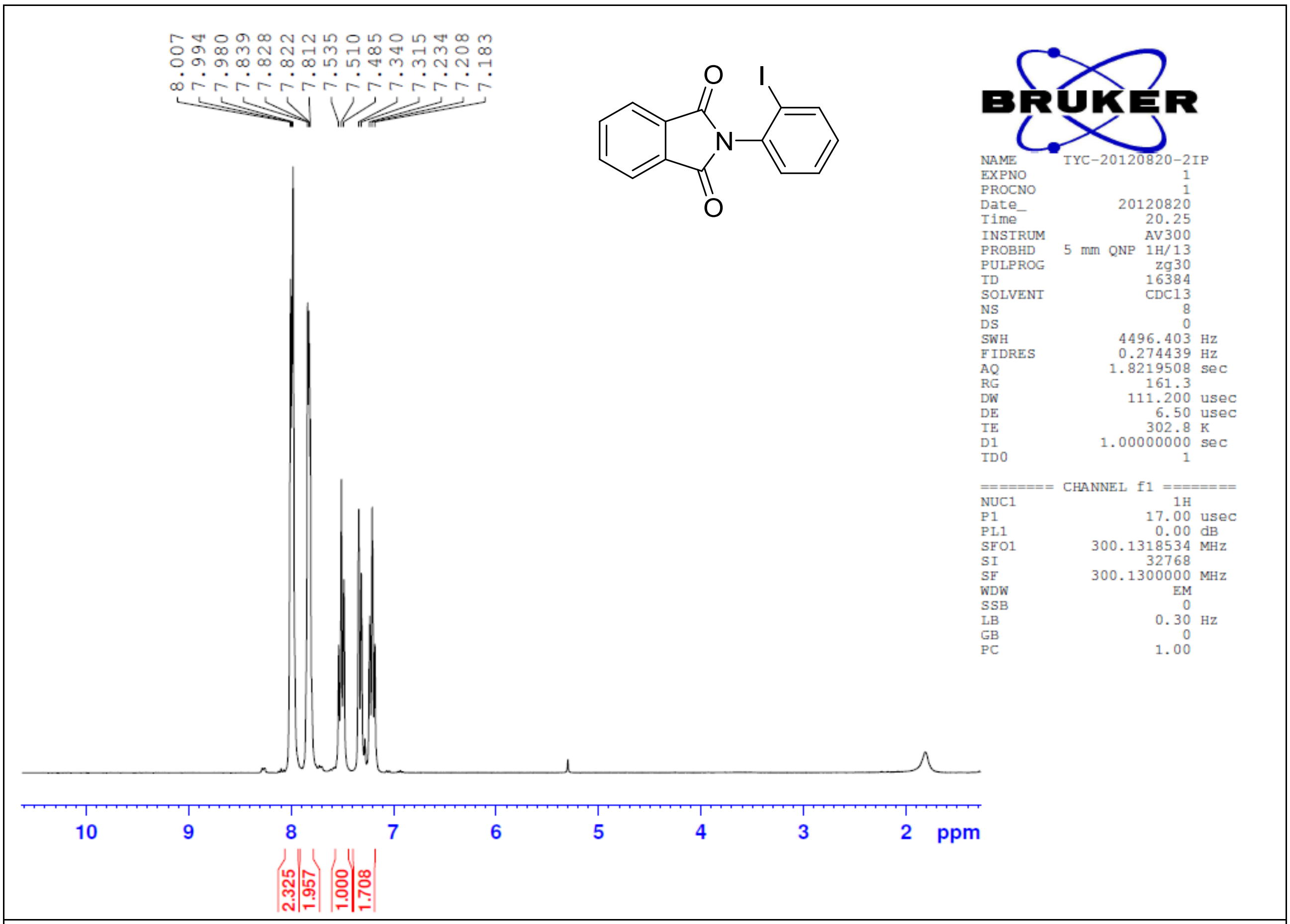
8.007
7.994
7.980
7.839
7.828
7.822
7.812
7.535
7.510
7.485
7.340
7.315
7.234
7.208
7.183
O
I
N
O
BRUKER
NAME TYC-20120820-2IP
EXPNO 1
PROCNO 1
Date_ 20120820
Time 20.25
INSTRUM AV300
PROBHD 5 mm QNP 1H/13
PULPROG zg30
TD 16384
SOLVENT CDCl3
NS 8
DS 0
SWH 4496.403 Hz
FIDRES 0.274439 Hz
AQ 1.8219508 sec
RG 161.3
DW 111.200 usec
DE 6.50 usec
TE 302.8 K
D1 1.00000000 sec
TD0 1
======== CHANNEL f1 ========
NUC1 1H
P1 17.00 usec
PL1 0.00 dB
SFO1 300.1318534 MHz
SI 32768
SF 300.1300000 MHz
WDW EM
SSB 0
LB 0.30 Hz
GB 0
PC 1.00
10 9 8 7 6 5 4 3 2 ppm
2.325
1.957
1.000
1.708

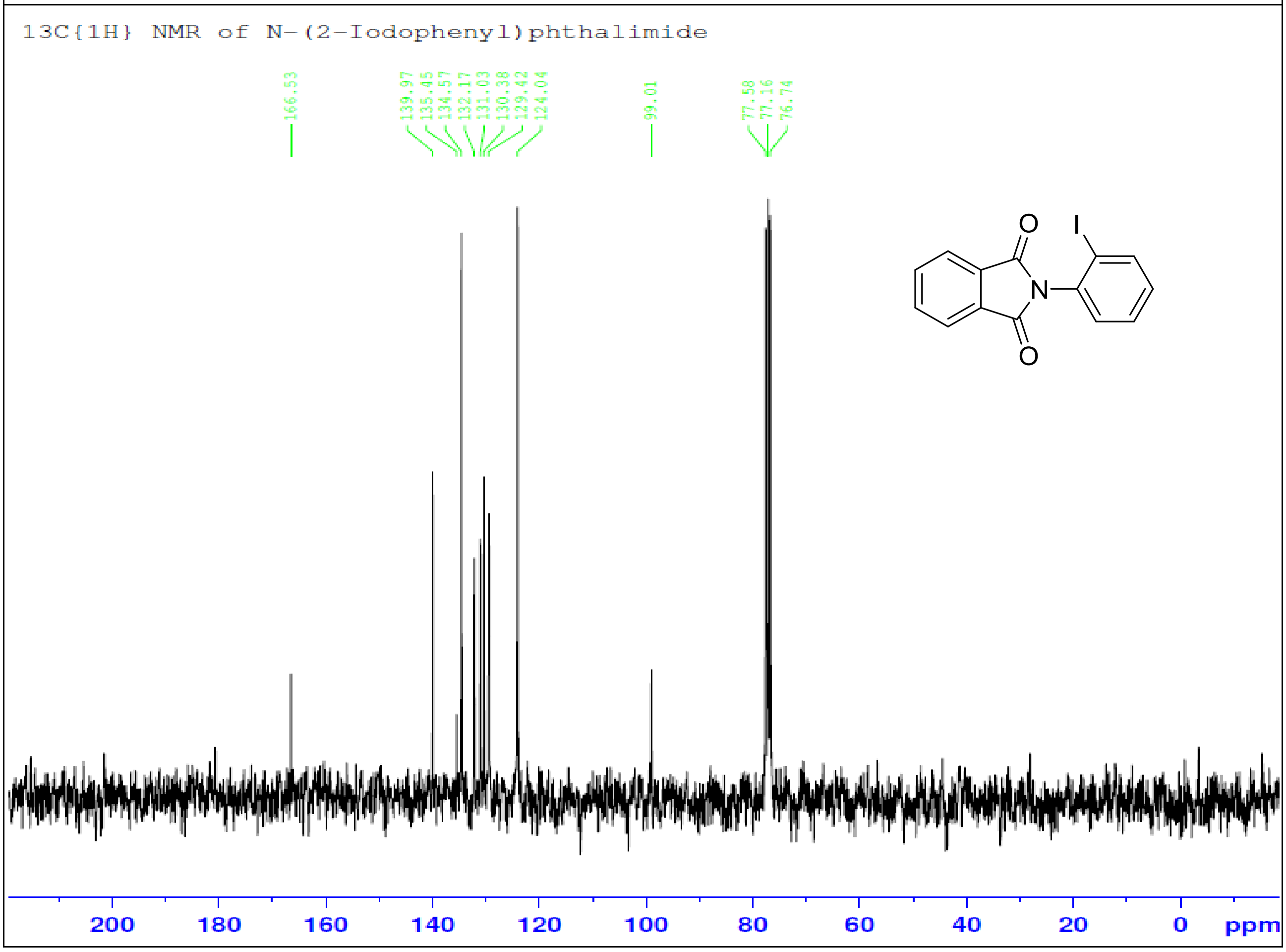
13C{1H} NMR of N-(2-Iodophenyl)phthalimide
166.53
139.97
135.45
134.57
132.17
131.03
130.38
129.42
124.04
99.01
77.58
77.16
76.74
O
I
N
O
200 180 160 140 120 100 80 60 40 20 0 ppm

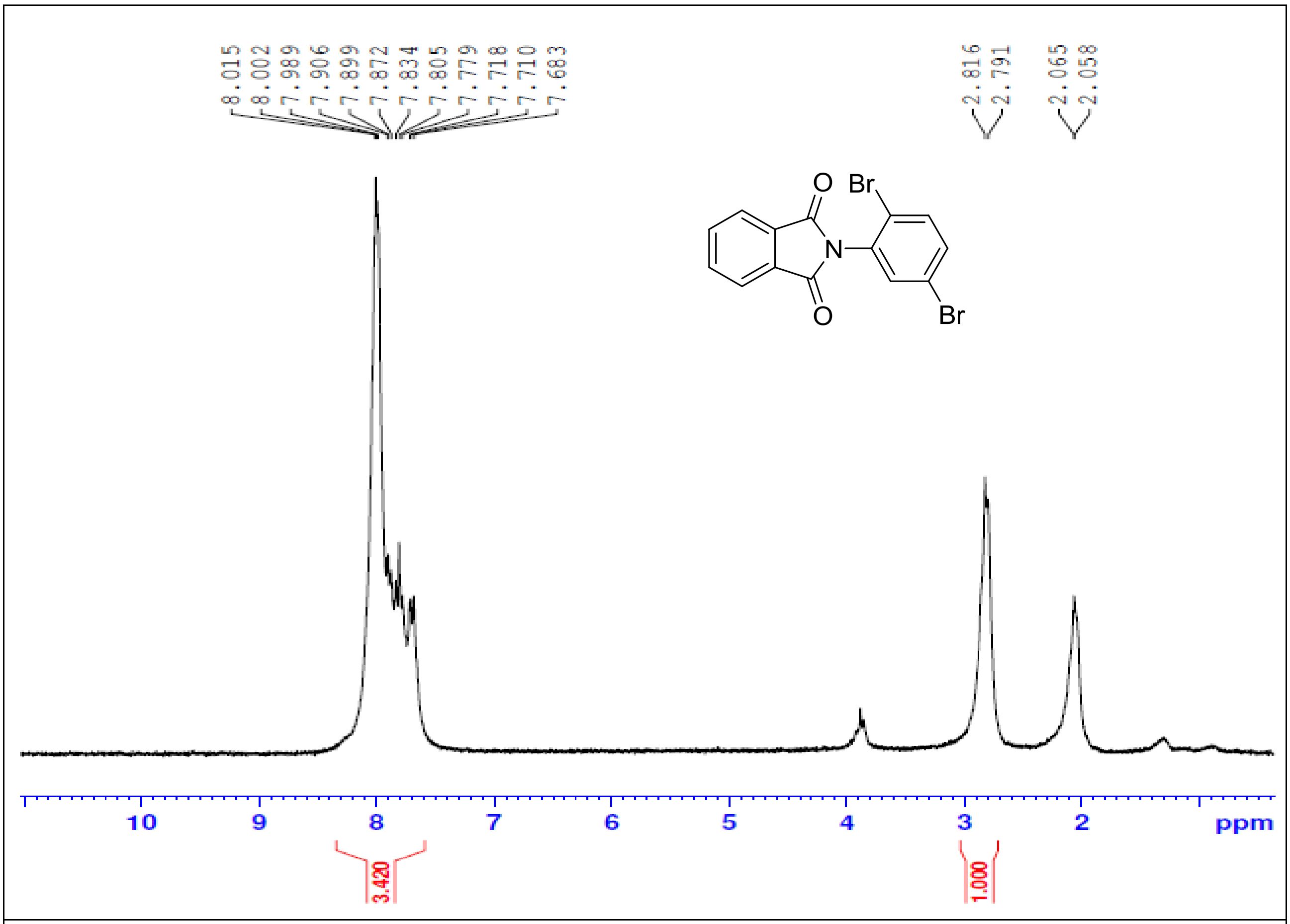
8.015
8.002
7.989
7.906
7.899
7.872
7.834
7.805
7.779
7.718
7.710
7.683
2.816
2.791
2.065
2.058
O
Br
N
O
Br
10
9
8
7
6
5
4
3
2
ppm
3.420
1.000

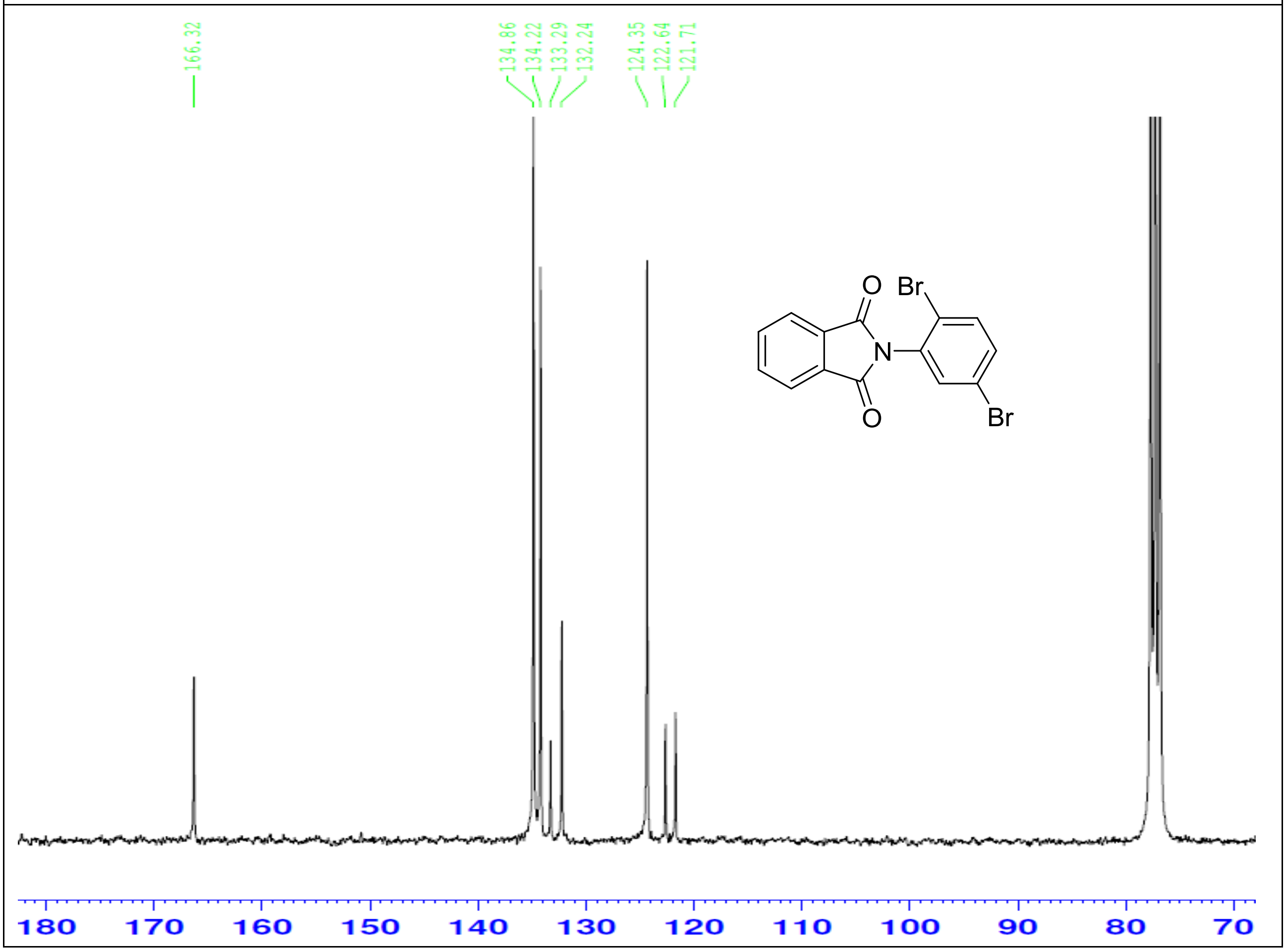
166.32
134.86
134.22
133.29
132.24
124.35
122.64
121.71
O
Br
N
O
Br
180
170
160
150
140
130
120
110
100
90
80
70

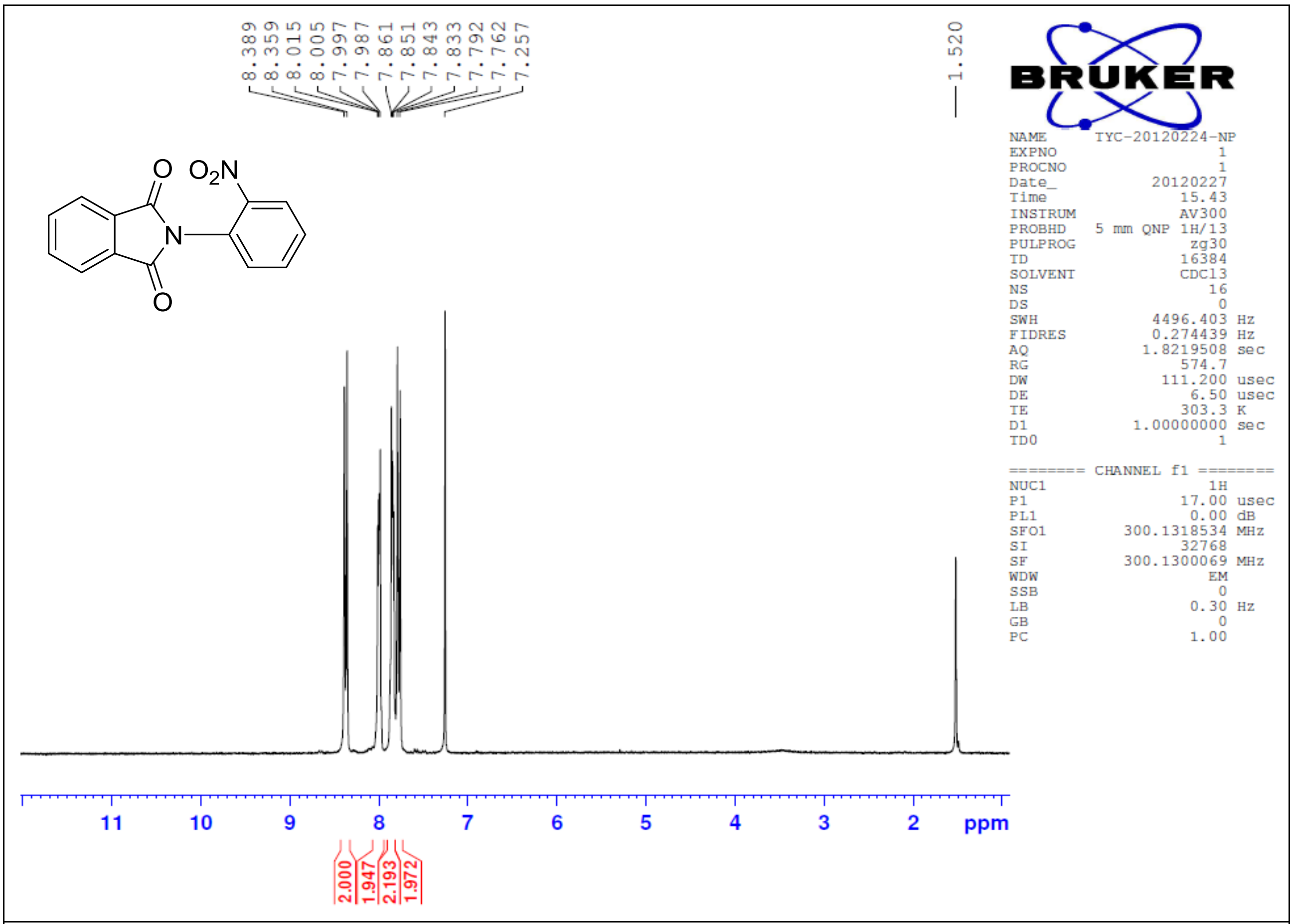
8.389
8.359
8.015
8.005
7.997
7.987
7.861
7.851
7.843
7.833
7.792
7.762
7.257
1.520
BRUKER
NAME TYC-20120224-NP
EXPNO 1
PROCNO 1
Date_ 20120227
Time 15.43
INSTRUM AV300
PROBHD 5 mm QNP 1H/13
PULPROG zg30
TD 16384
SOLVENT CDCl3
NS 16
DS 0
SWH 4496.403 Hz
FIDRES 0.274439 Hz
AQ 1.8219508 sec
RG 574.7
DW 111.200 usec
DE 6.50 usec
TE 303.3 K
D1 1.00000000 sec
TD0 1
======== CHANNEL f1 ========
NUC1 1H
P1 17.00 usec
PL1 0.00 dB
SFO1 300.1318534 MHz
SI 32768
SF 300.1300069 MHz
WDW EM
SSB 0
LB 0.30 Hz
GB 0
PC 1.00
O
O2N
N
O
11
10
9
8
7
6
5
4
3
2
ppm
2.000
1.947
2.193
1.972

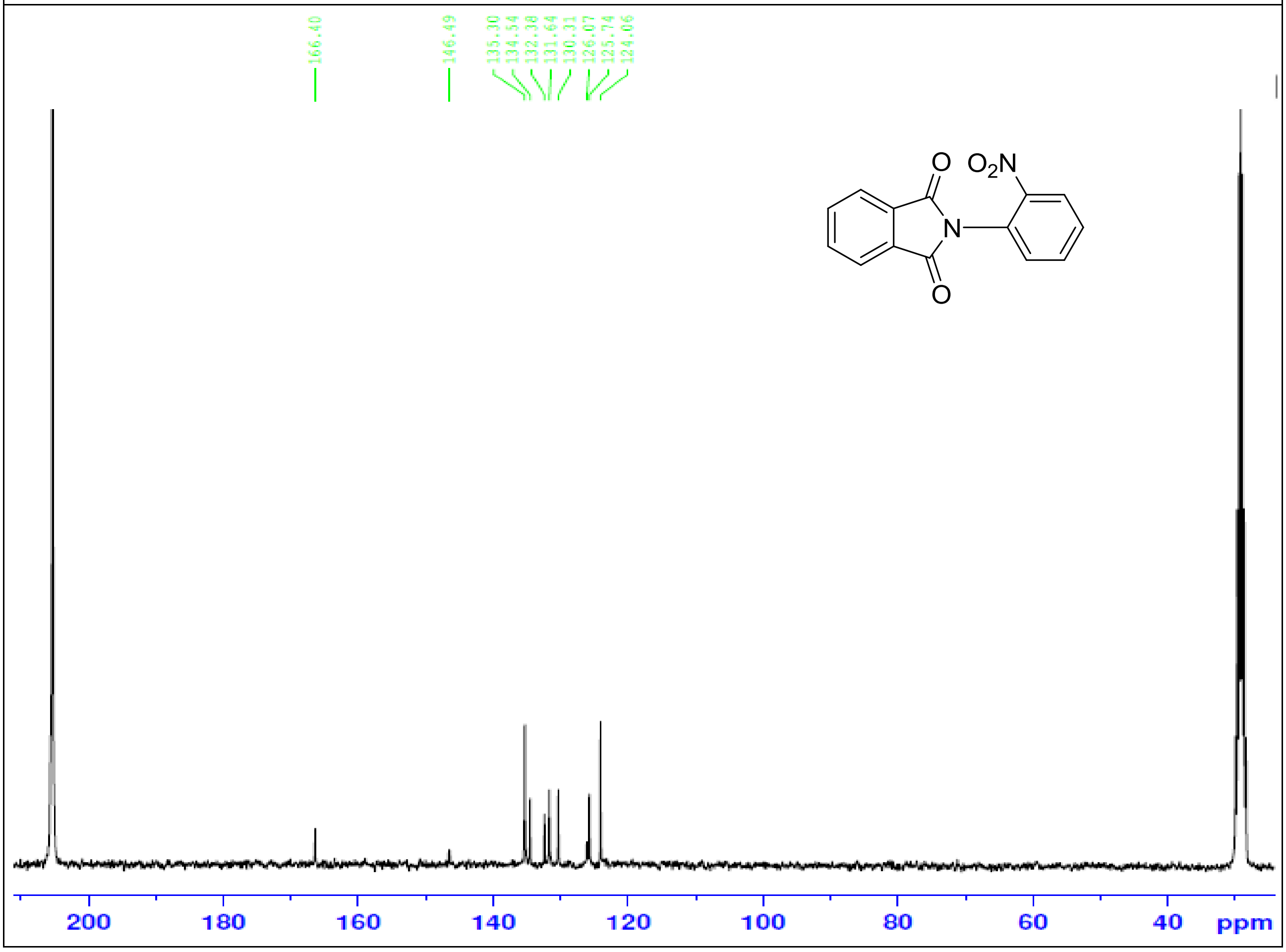
166.40
146.49
135.30
134.54
132.38
131.64
130.31
126.07
125.74
124.06
O
O2N
N
O
200
180
160
140
120
100
80
60
40
ppm

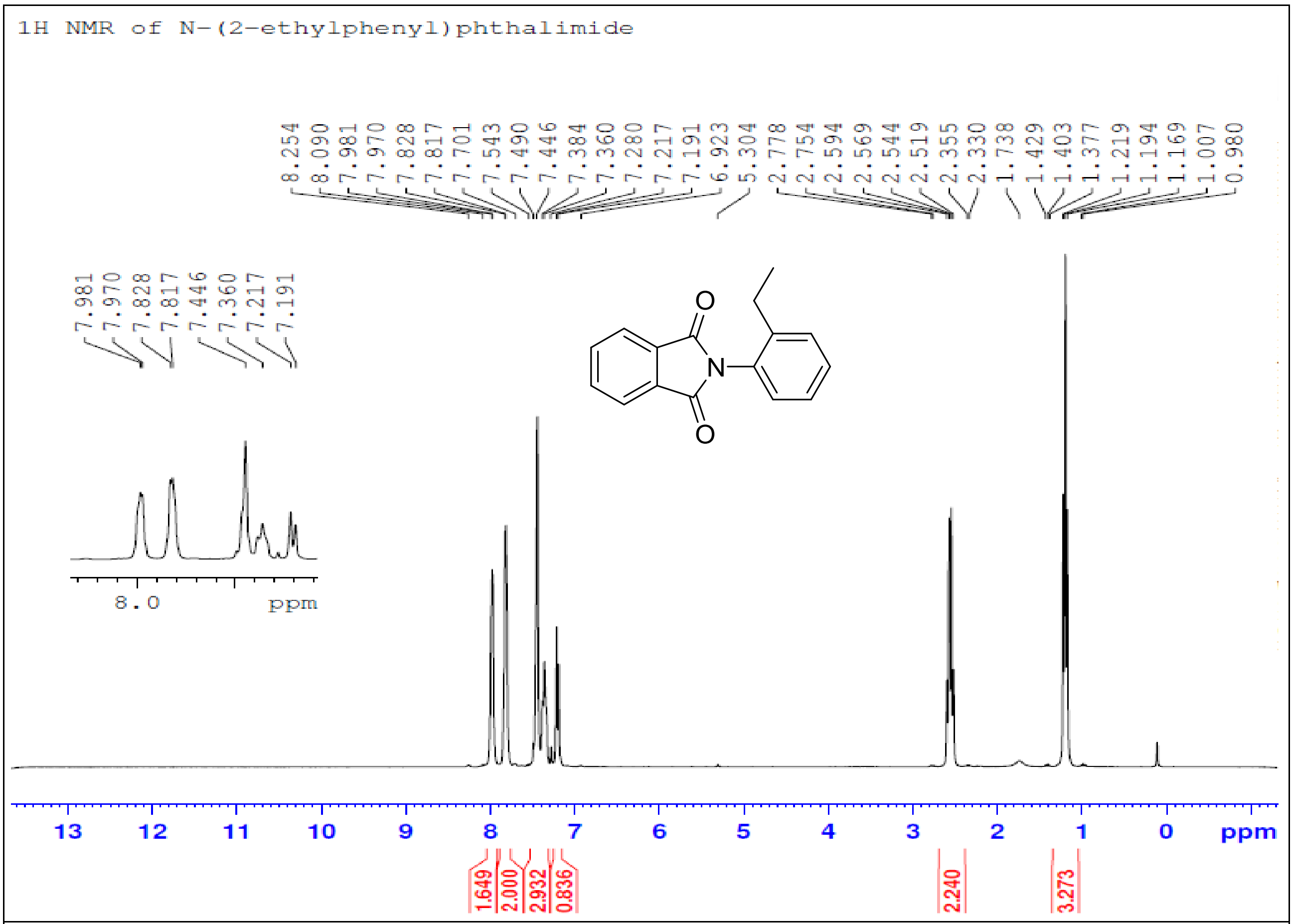
1H NMR of N-(2-ethylphenyl)phthalimide
8.254
8.090
7.981
7.970
7.828
7.817
7.701
7.543
7.490
7.446
7.384
7.360
7.280
7.217
7.191
6.923
5.304
2.778
2.754
2.594
2.569
2.544
2.519
2.355
2.330
1.738
1.429
1.403
1.377
1.219
1.194
1.169
1.007
0.980
O
N
O
8.0
ppm
13
12
11
10
9
8
7
6
5
4
3
2
1
0
ppm
1.649
2.000
2.932
0.836
2.240
3.273

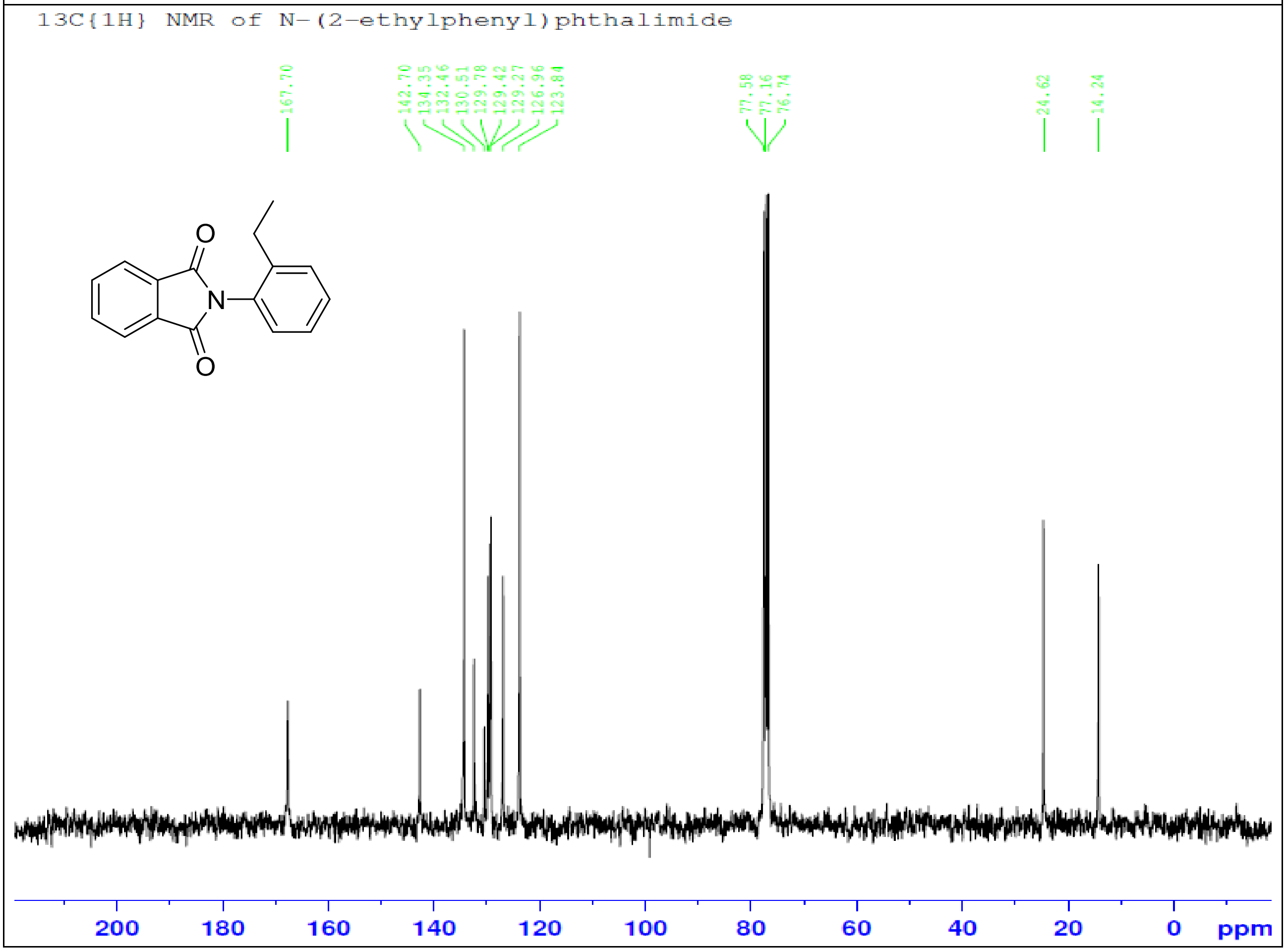
13C{1H} NMR of N-(2-ethylphenyl)phthalimide
167.70
142.70
134.35
132.46
130.51
129.78
129.42
129.27
126.96
123.84
77.58
77.16
76.74
24.62
14.24
O
N
O
200
180
160
140
120
100
80
60
40
20
0
ppm

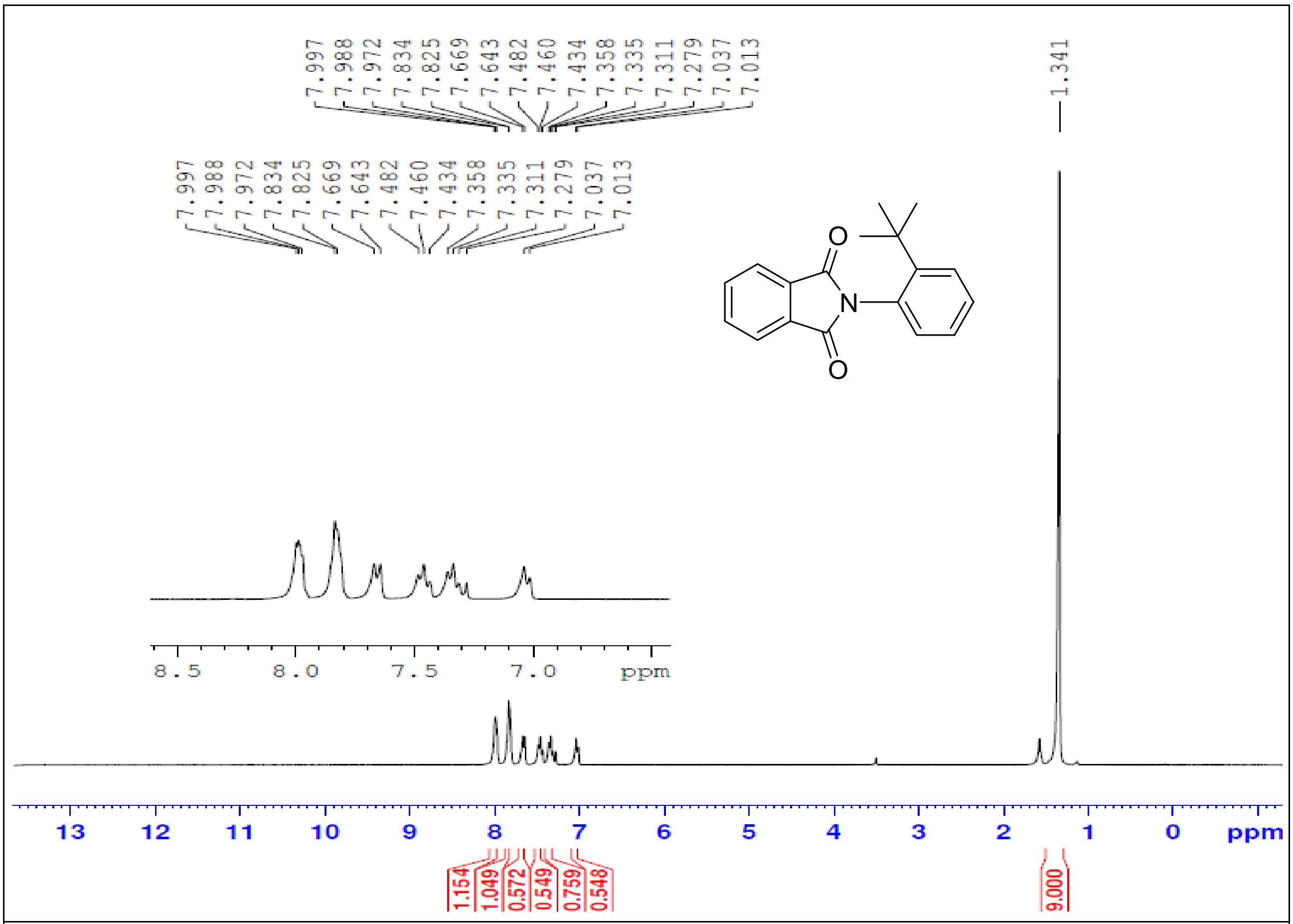
7.997
7.988
7.972
7.834
7.825
7.669
7.643
7.482
7.460
7.434
7.358
7.335
7.311
7.279
7.037
7.013
1.341
O
N
O
8.5
8.0
7.5
7.0
ppm
13
12
11
10
9
8
7
6
5
4
3
2
1
0
ppm
1.154
1.049
0.572
0.549
0.759
0.548
9.000

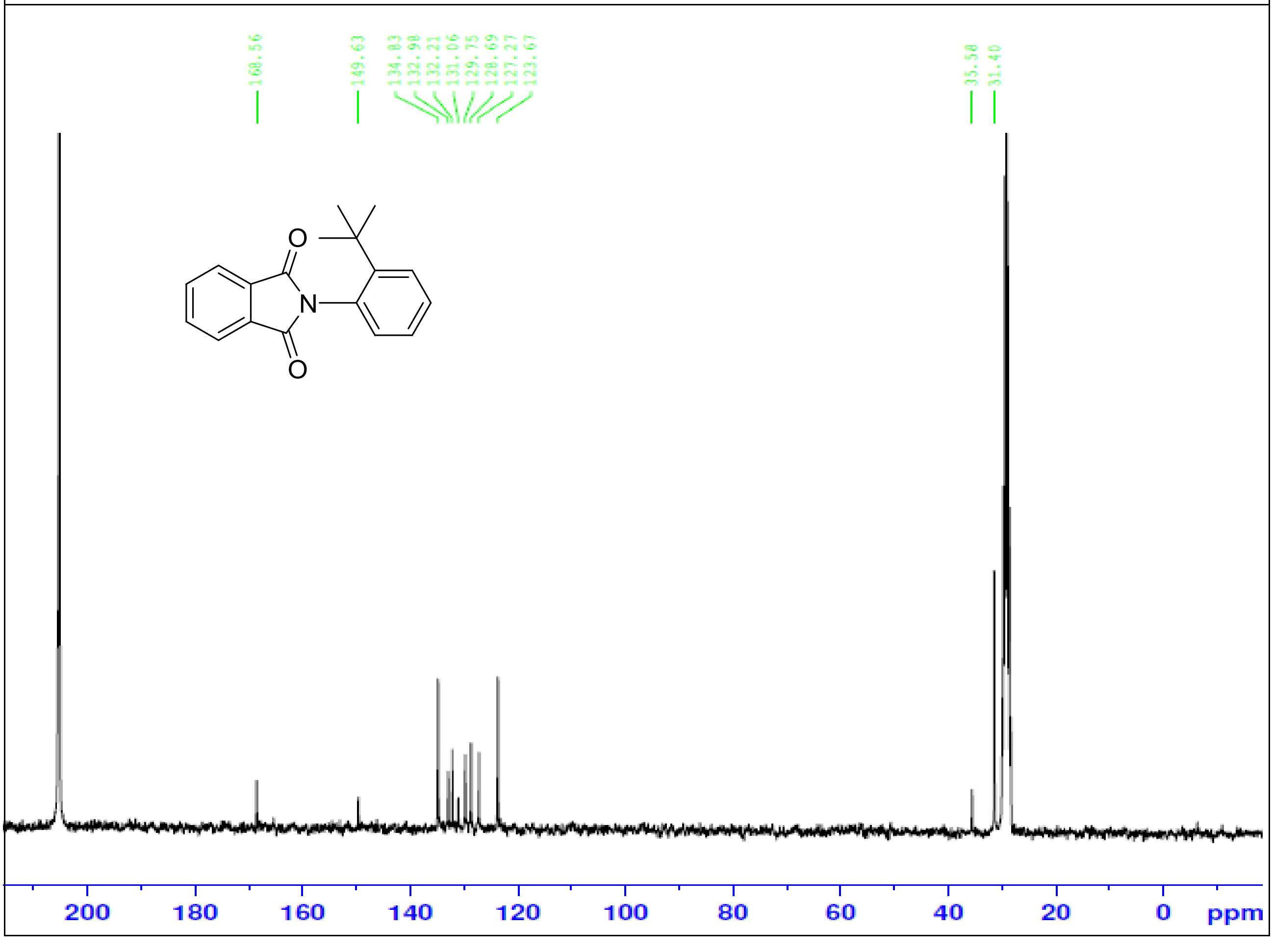
168.56
149.63
134.83
132.98
132.21
131.06
129.75
128.69
127.27
123.67
35.58
31.40
O
N
O
200
180
160
140
120
100
80
60
40
20
0
ppm

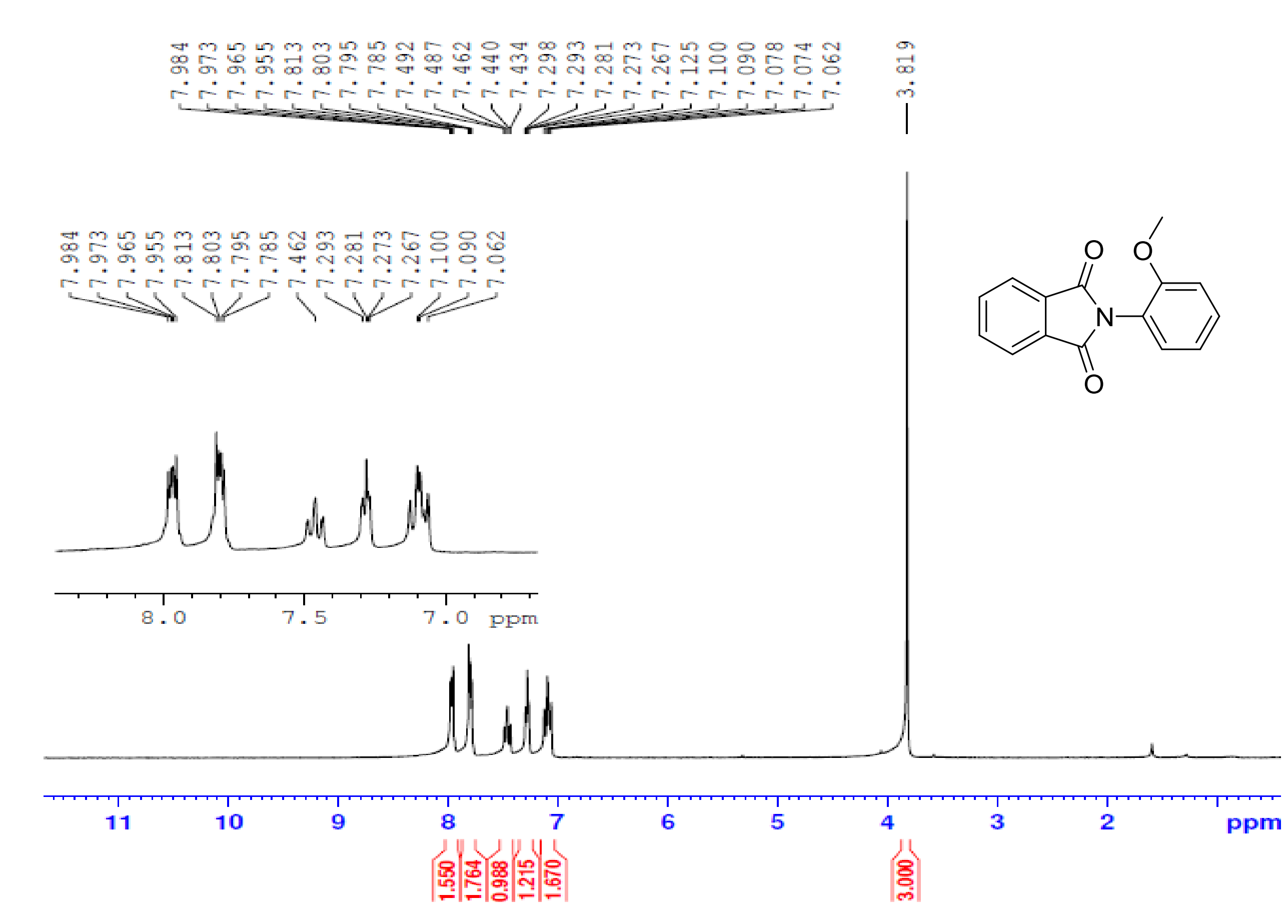

13C{1H} NMR of N-(ortho-methoxyphenyl)phthlaimide

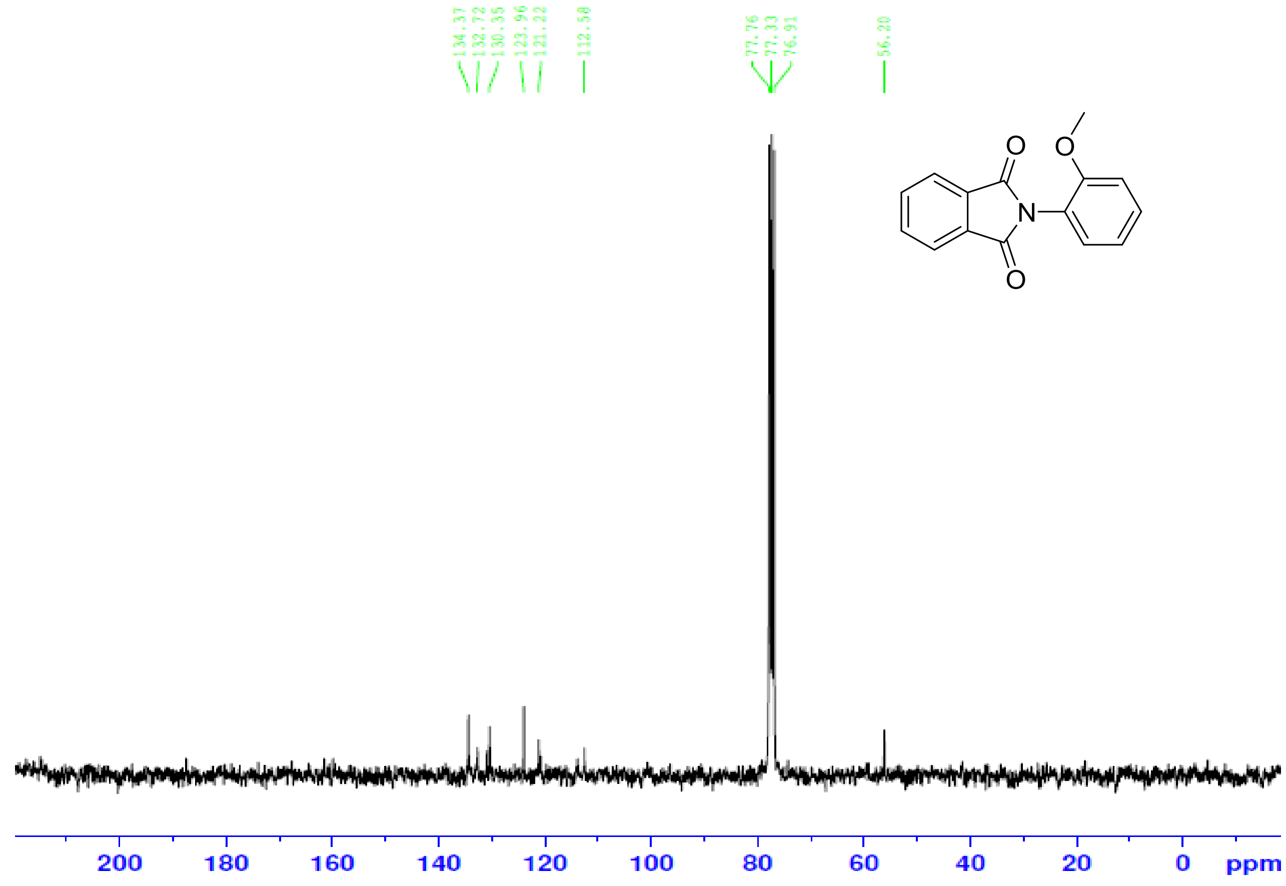

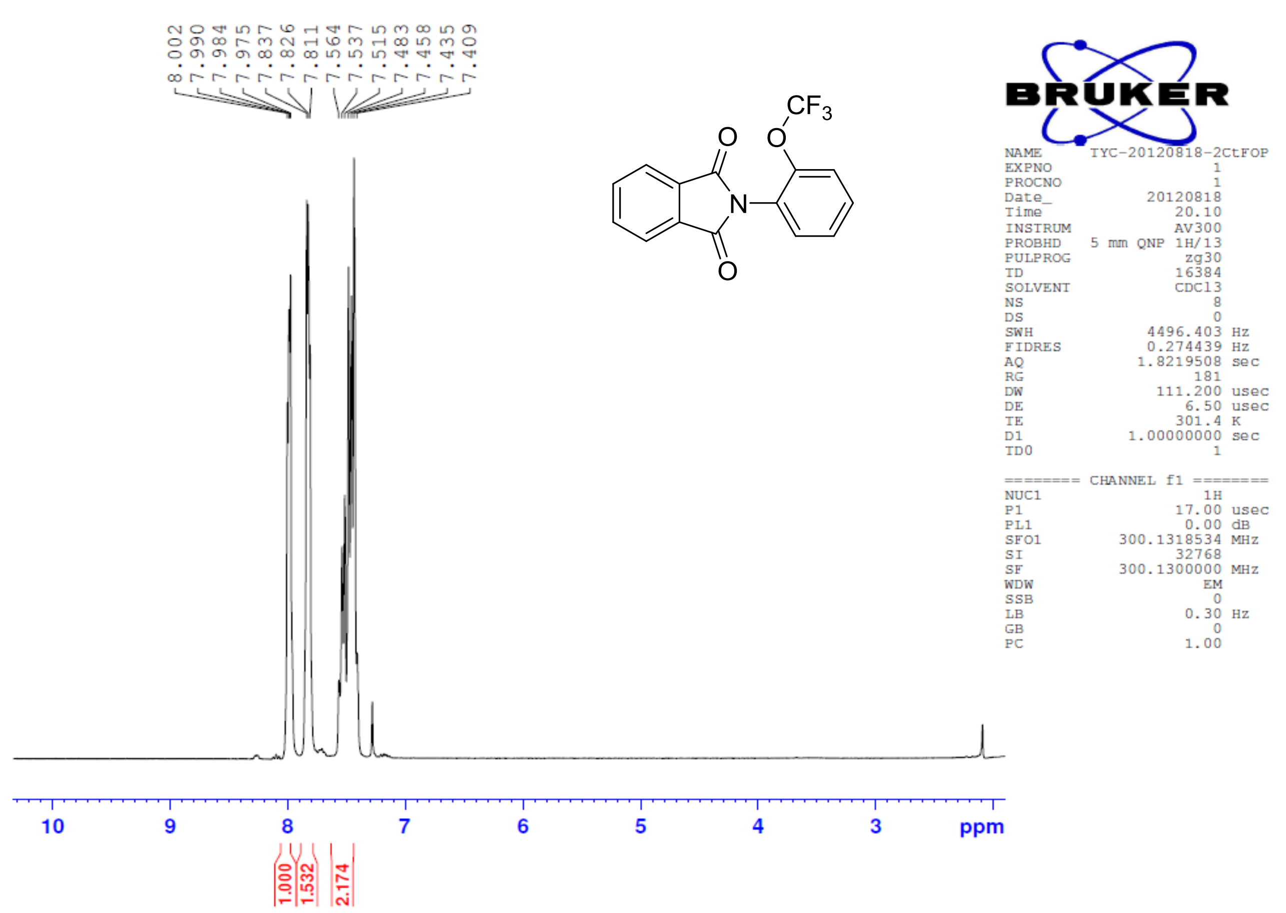

13C{1H} NMR of N-(2-trifluoromethoxyphenyl)phthalimide

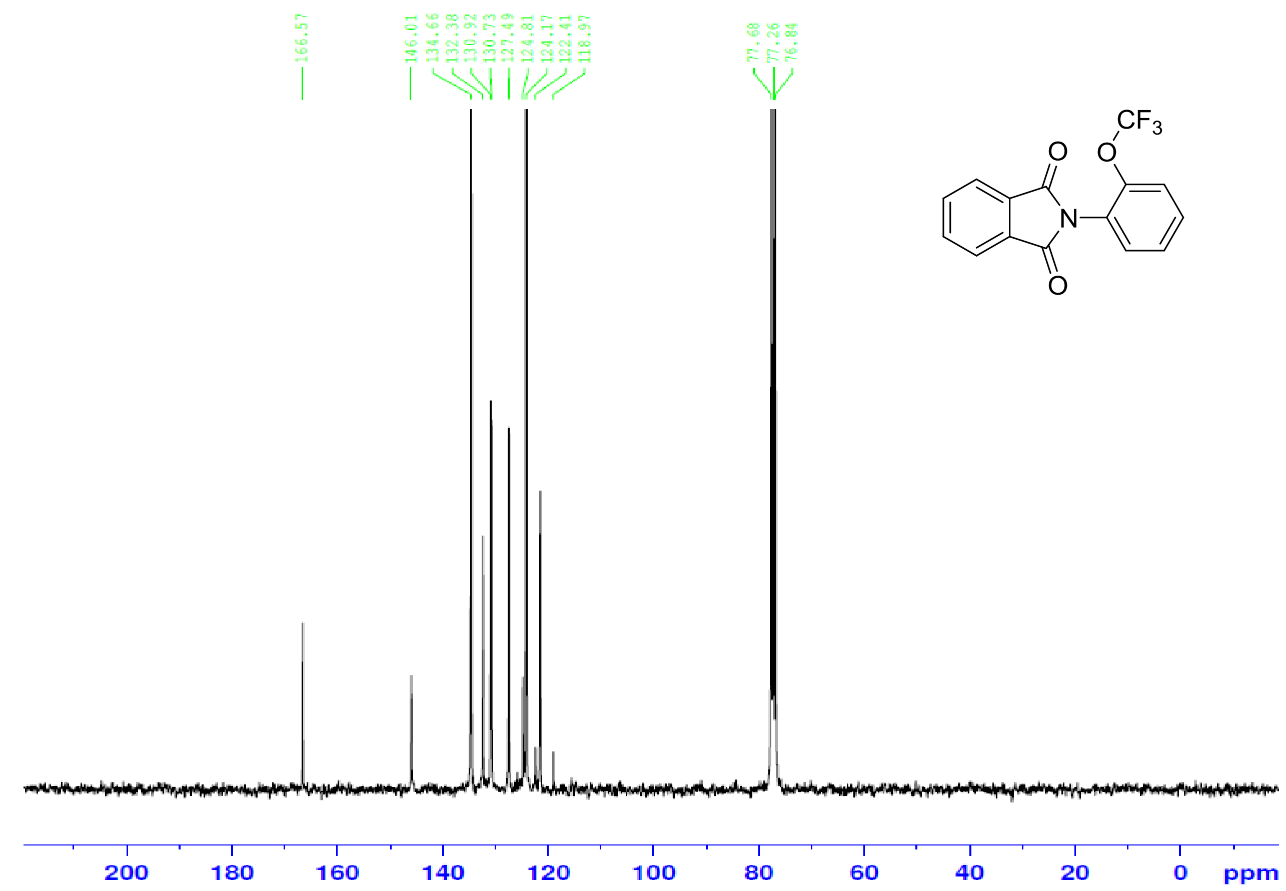

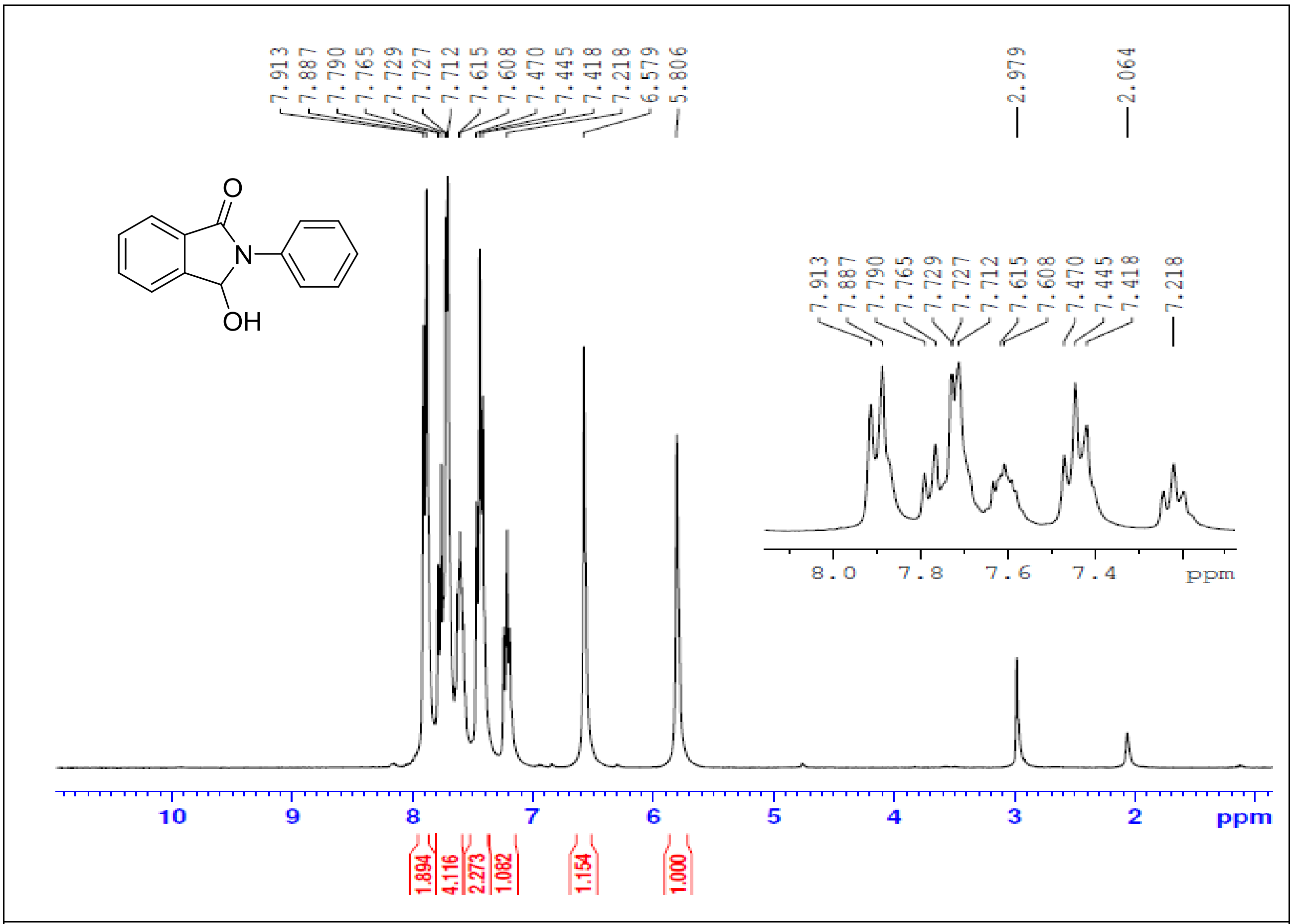
7.913
7.887
7.790
7.765
7.729
7.727
7.712
7.615
7.608
7.470
7.445
7.418
7.218
6.579
5.806
2.979
2.064
O
N
OH
8.0
7.8
7.6
7.4
ppm
10
9
8
7
6
5
4
3
2
ppm
1.894
4.116
2.273
1.082
1.154
1.000

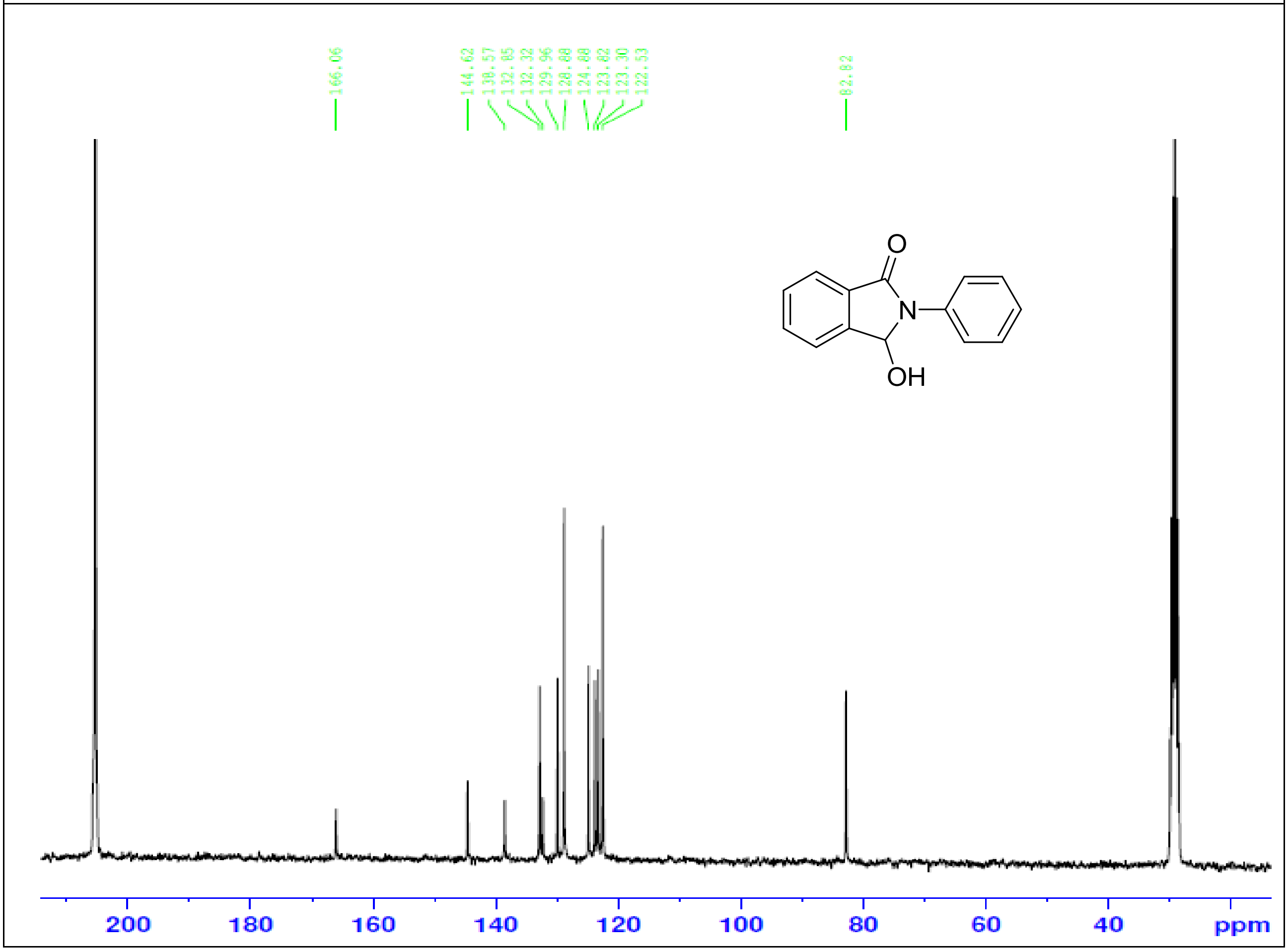
166.06
144.62
138.57
132.85
132.32
129.96
128.80
124.80
123.82
123.30
122.53
82.82
O
N
OH
200
180
160
140
120
100
80
60
40
ppm

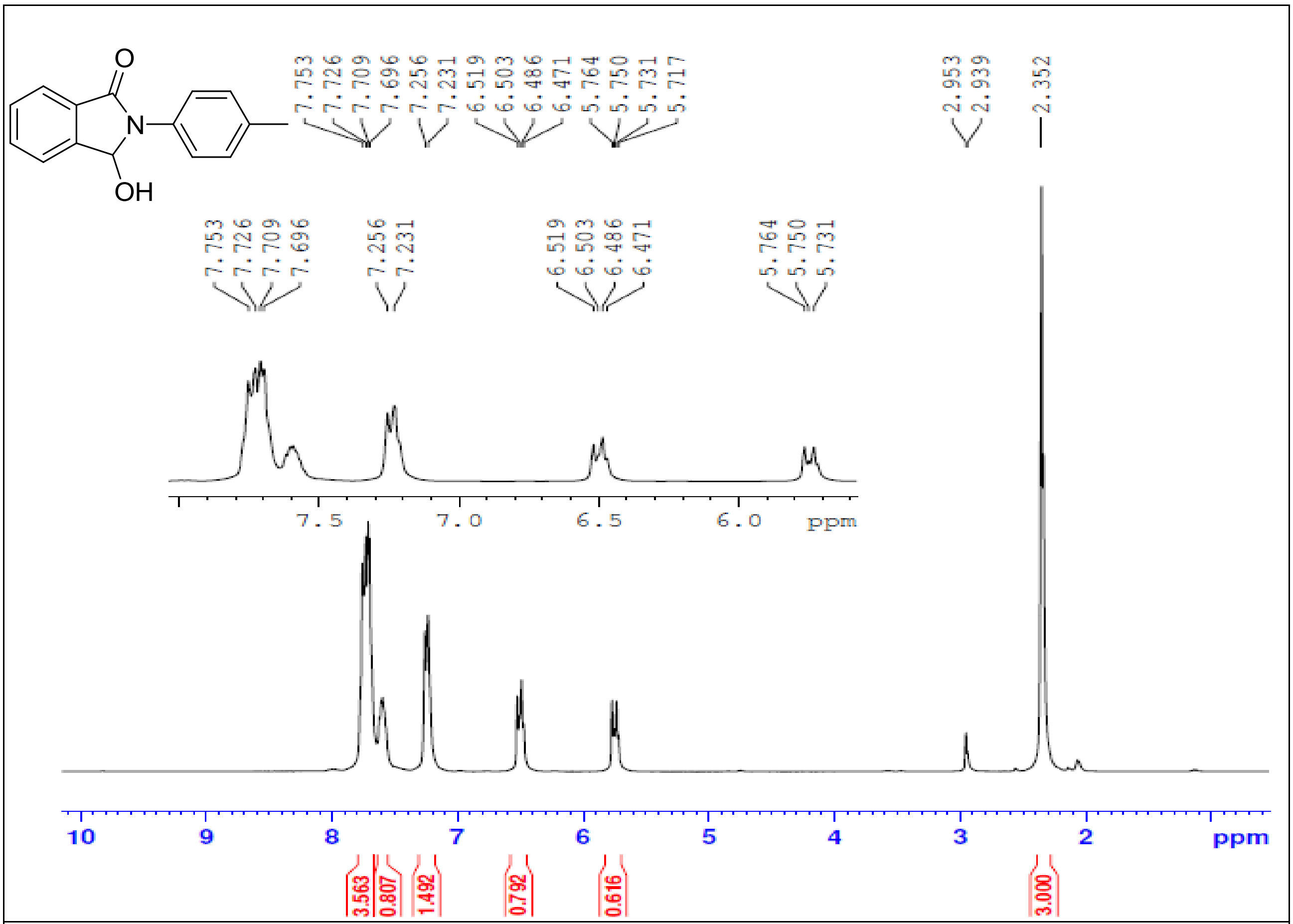
O
N
OH
7.753
7.726
7.709
7.696
7.256
7.231
6.519
6.503
6.486
6.471
5.764
5.750
5.731
5.717
2.953
2.939
2.352
7.5
7.0
6.5
6.0
ppm
10
9
8
7
6
5
4
3
2
ppm
3.563
0.807
1.492
0.792
0.616
3.000

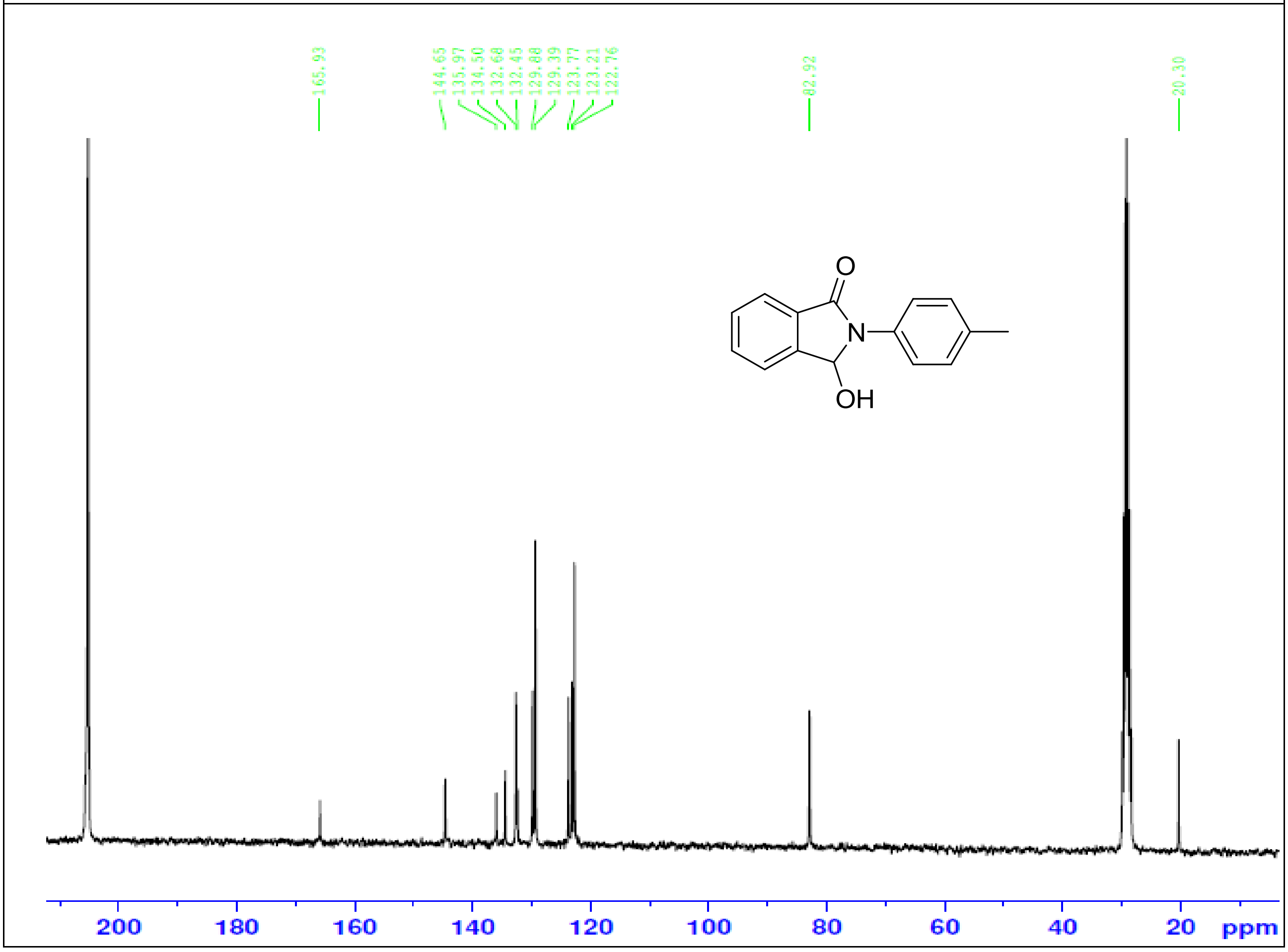
165.93
144.65
135.97
134.50
132.68
132.45
129.88
129.39
123.77
123.21
122.76
82.92
20.30
O
N
OH
200
180
160
140
120
100
80
60
40
20
ppm

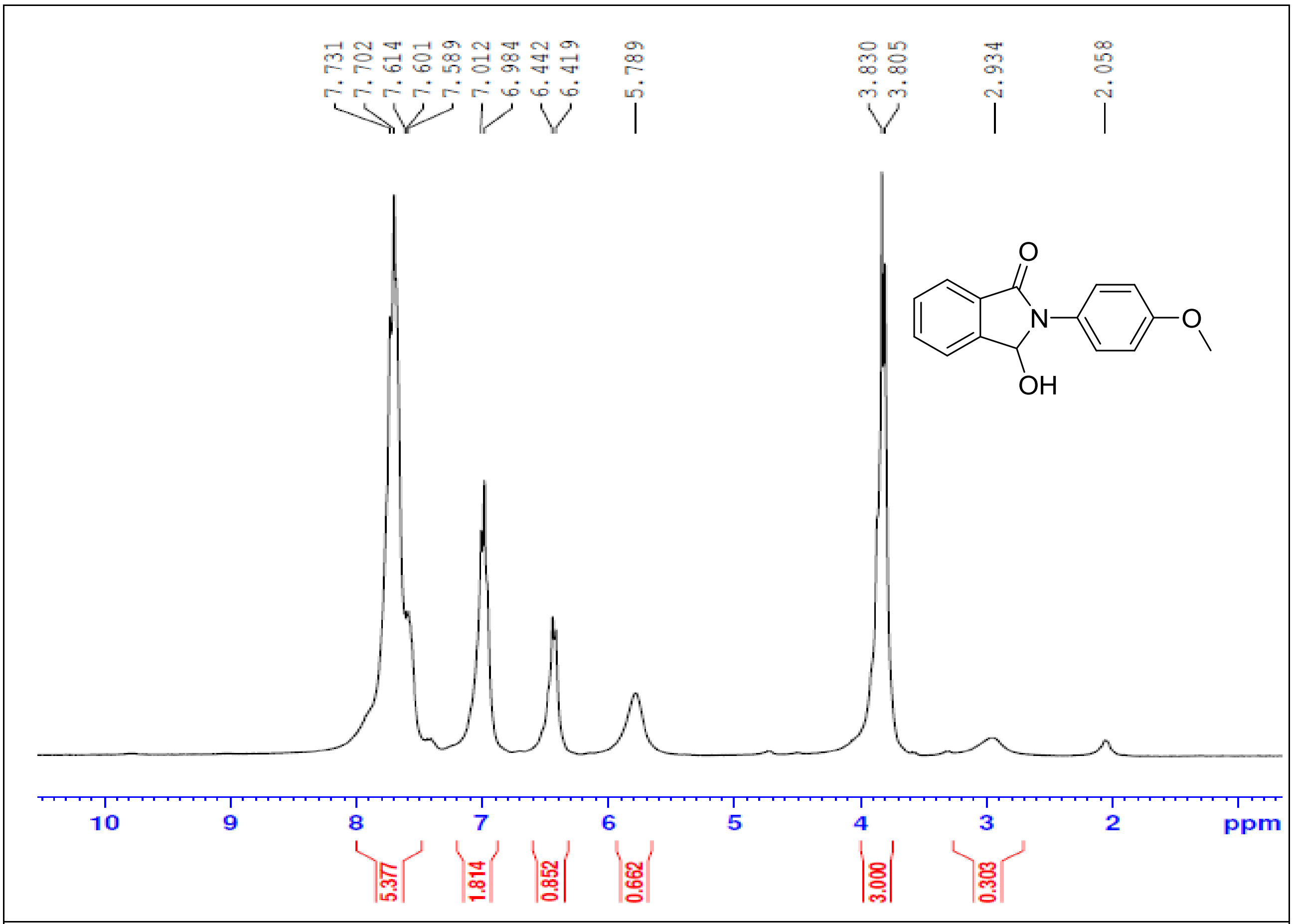
7.731
7.702
7.614
7.601
7.589
7.012
6.984
6.442
6.419
5.789
3.830
3.805
2.934
2.058
O
N
O
OH
10
9
8
7
6
5
4
3
2
ppm
5.377
1.814
0.852
0.662
3.000
0.303

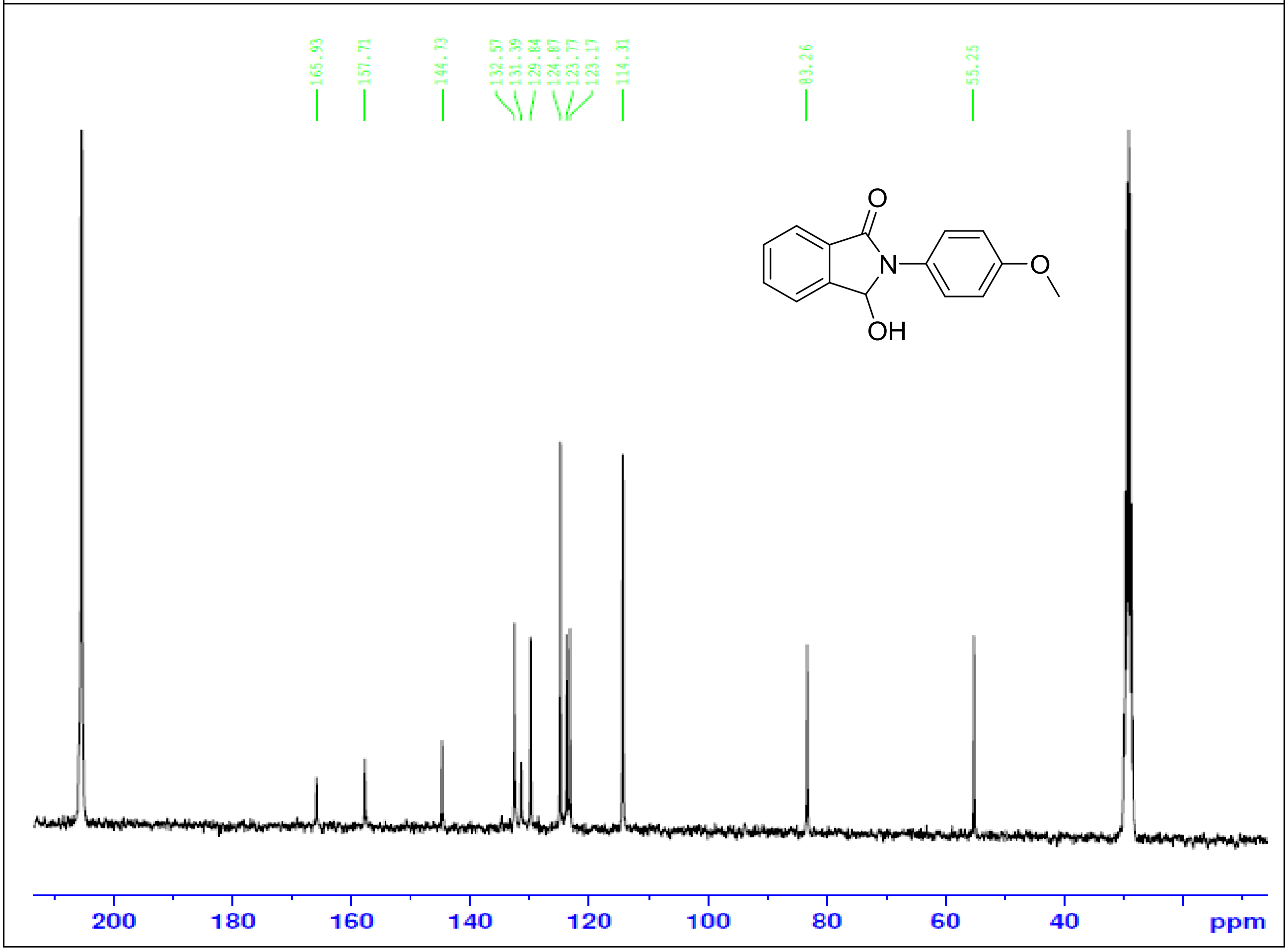
165.93
157.71
144.73
132.57
131.39
129.84
124.87
123.77
123.17
114.31
83.26
55.25
O
N
O
OH
200
180
160
140
120
100
80
60
40
ppm

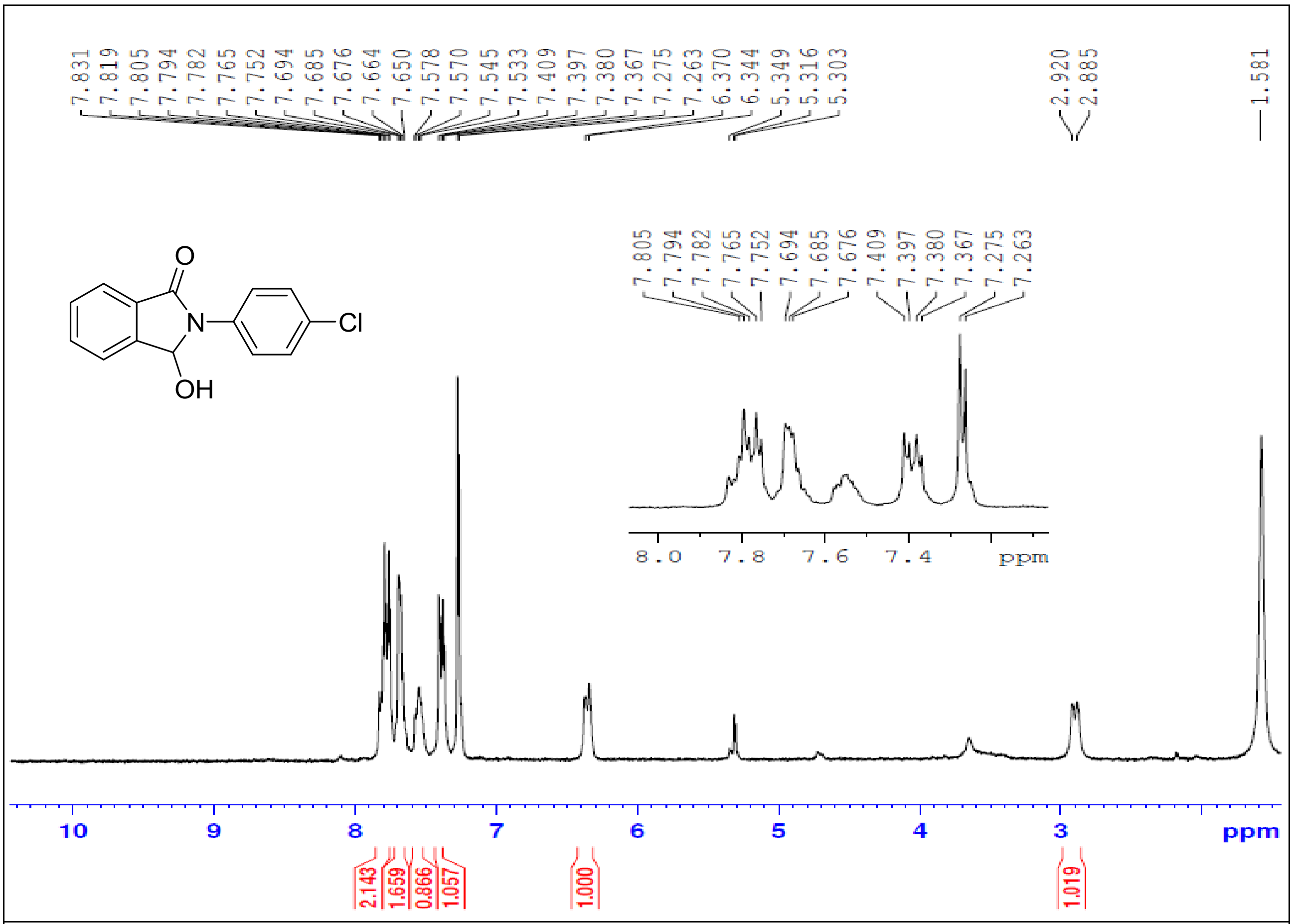
7.831
7.819
7.805
7.794
7.782
7.765
7.752
7.694
7.685
7.676
7.664
7.650
7.578
7.570
7.545
7.533
7.409
7.397
7.380
7.367
7.275
7.263
6.370
6.344
5.349
5.316
5.303
2.920
2.885
1.581
O
N
Cl
OH
8.0
7.8
7.6
7.4
ppm
10
9
8
7
6
5
4
3
ppm
2.143
1.659
0.866
1.057
1.000
1.019

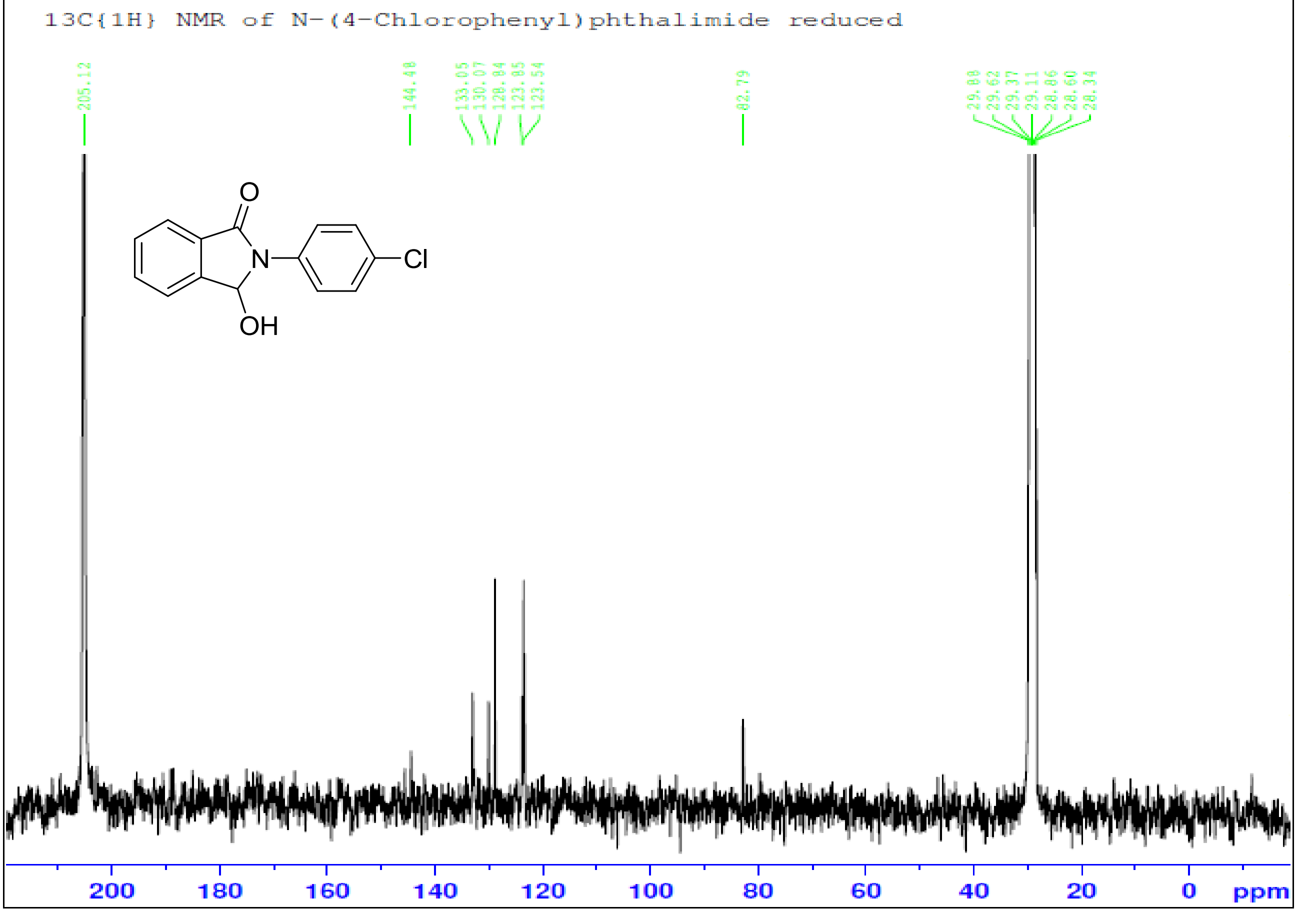
13C{1H} NMR of N-(4-Chlorophenyl)phthalimide reduced
205.12
144.48
133.05
130.07
128.94
123.85
123.54
82.79
29.88
29.62
29.37
29.11
28.86
28.60
28.34
O
N
Cl
OH
200
180
160
140
120
100
80
60
40
20
0
ppm

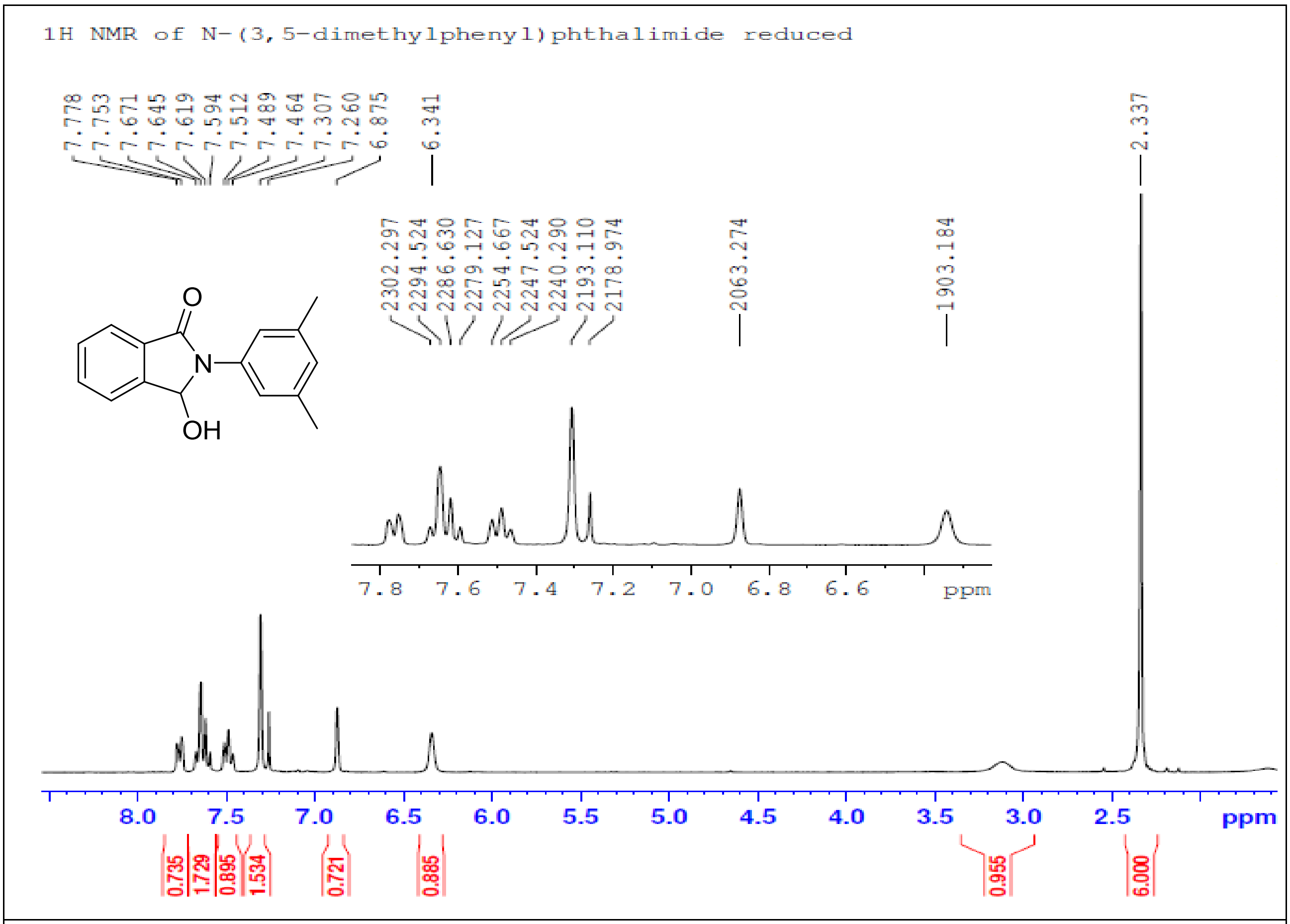
1H NMR of N-(3,5-dimethylphenyl)phthalimide reduced
7.778 7.753 7.671 7.645 7.619 7.594 7.512 7.489 7.464 7.307 7.260 6.875
6.341
2.337
2302.297 2294.524 2286.630 2279.127 2254.667 2247.524 2240.290 2193.110 2178.974
2063.274
1903.184
O
N
OH
7.8 7.6 7.4 7.2 7.0 6.8 6.6 ppm
8.0 7.5 7.0 6.5 6.0 5.5 5.0 4.5 4.0 3.5 3.0 2.5 ppm
0.735 1.729 0.895 1.534 0.721 0.885 0.955 6.000

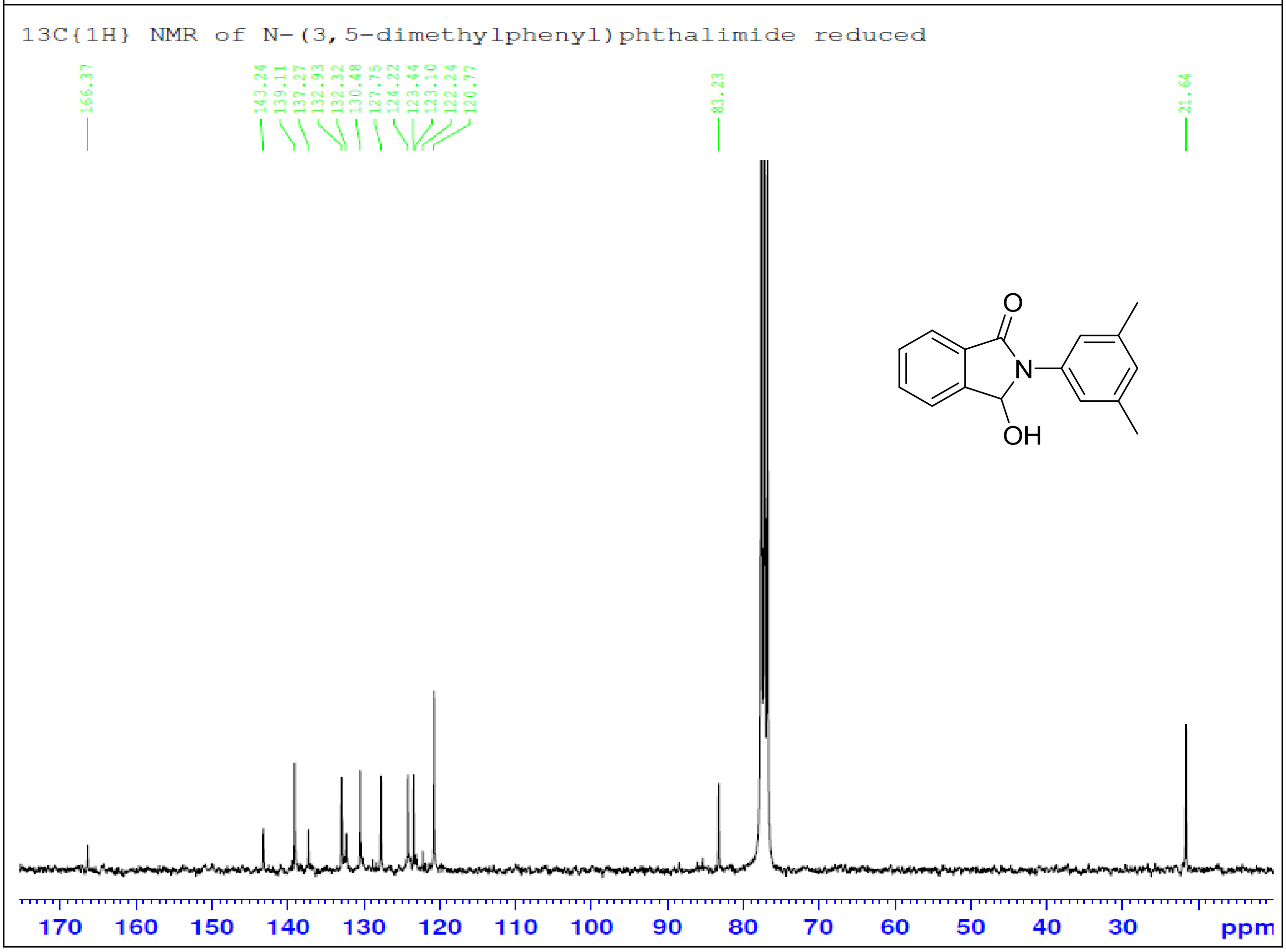
13C{1H} NMR of N-(3,5-dimethylphenyl)phthalimide reduced
166.37
143.24 139.11 137.27 132.93 132.32 130.48 127.75 124.22 123.44 123.10 122.24 120.77
83.23
21.64
O
N
OH
170 160 150 140 130 120 110 100 90 80 70 60 50 40 30 ppm

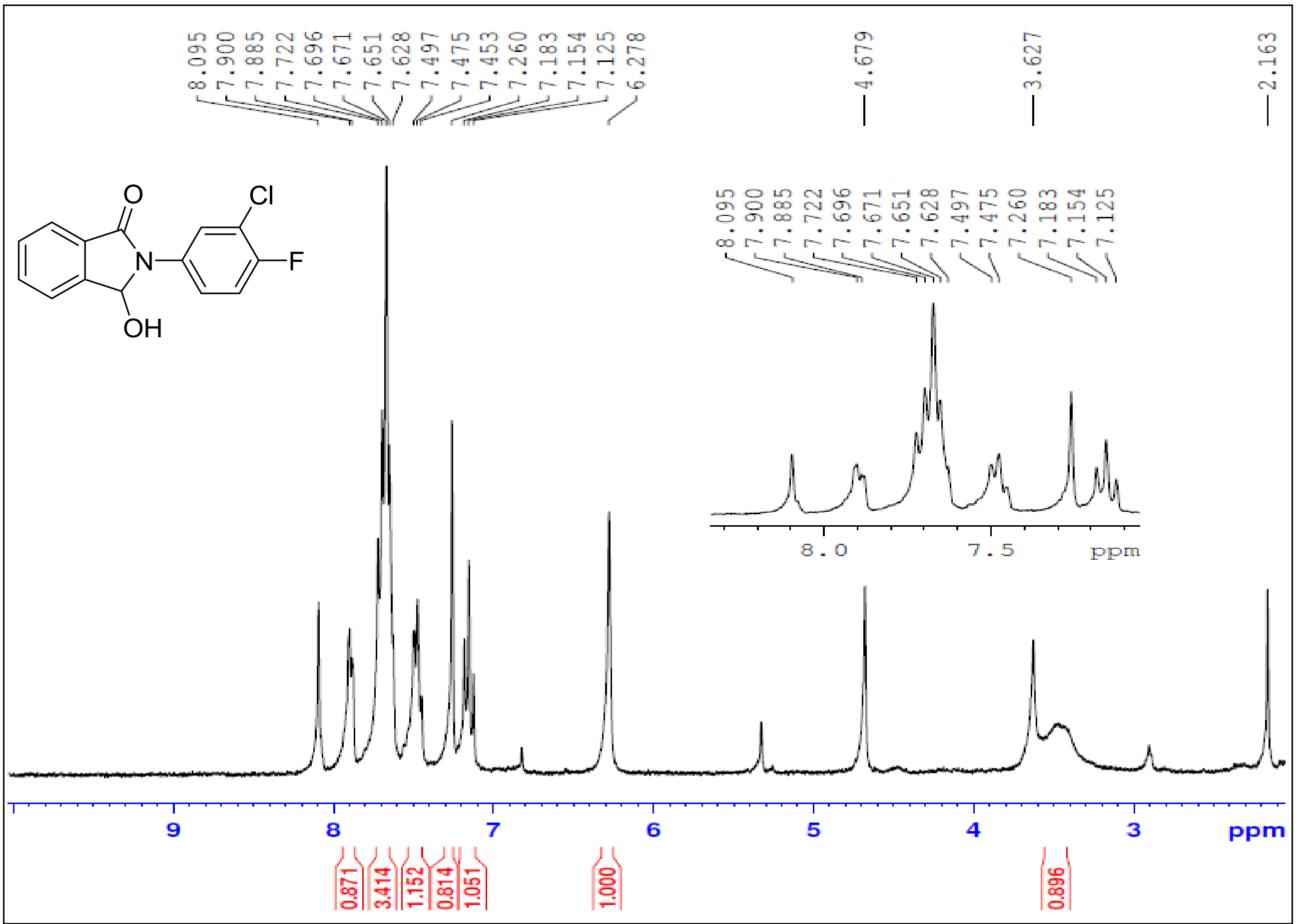
8.095 7.900 7.885 7.722 7.696 7.671 7.651 7.628 7.497 7.475 7.453 7.260 7.183 7.154 7.125 6.278
4.679
3.627
2.163
O
Cl
N
F
OH
8.0
7.5
ppm
9 8 7 6 5 4 3 ppm
0.871 3.414 1.152 0.814 1.051 1.000 0.896

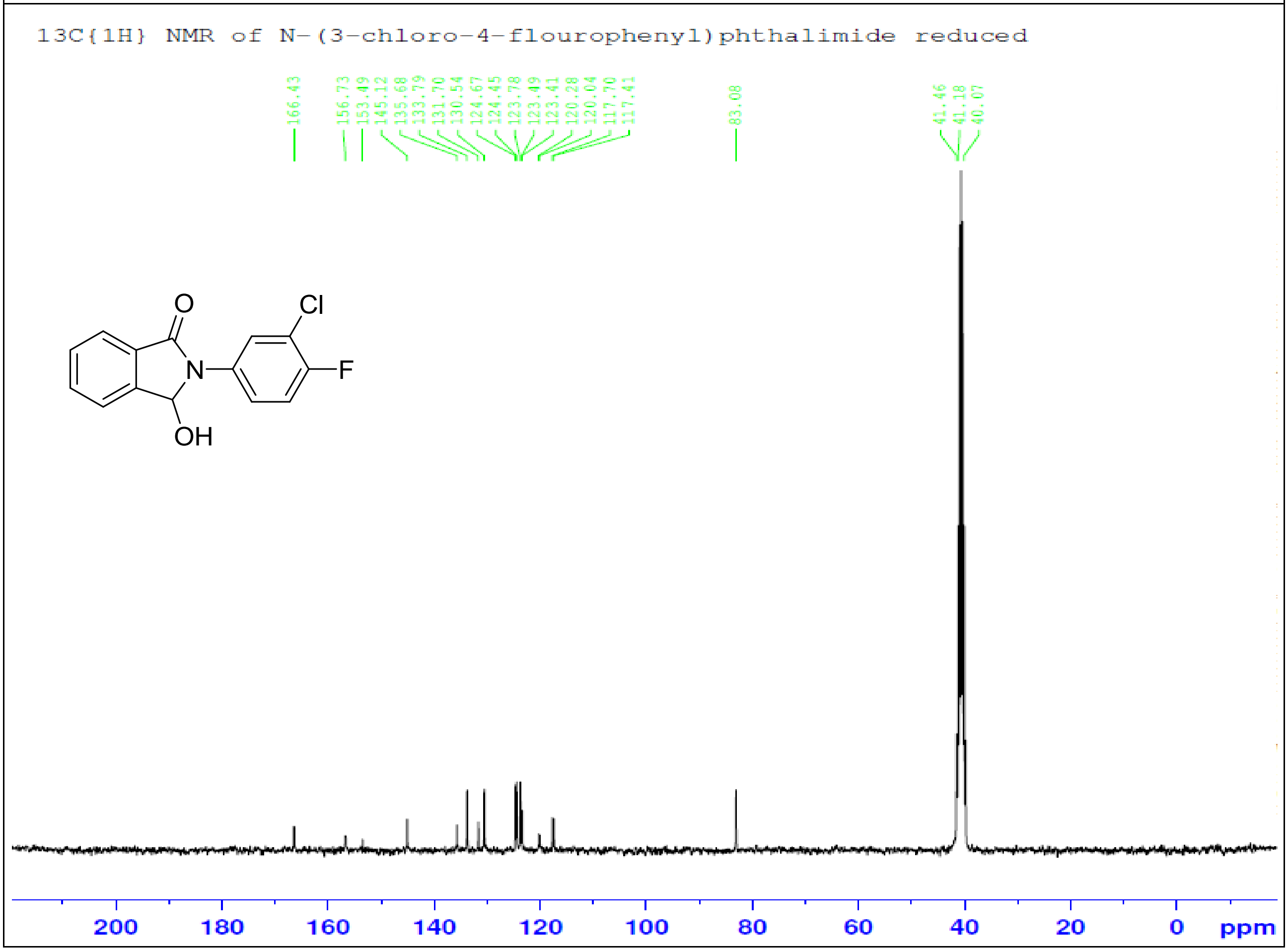
13C{1H} NMR of N-(3-chloro-4-flourophenyl)phthalimide reduced
O
Cl
N
F
OH
200 180 160 140 120 100 80 60 40 20 0 ppm

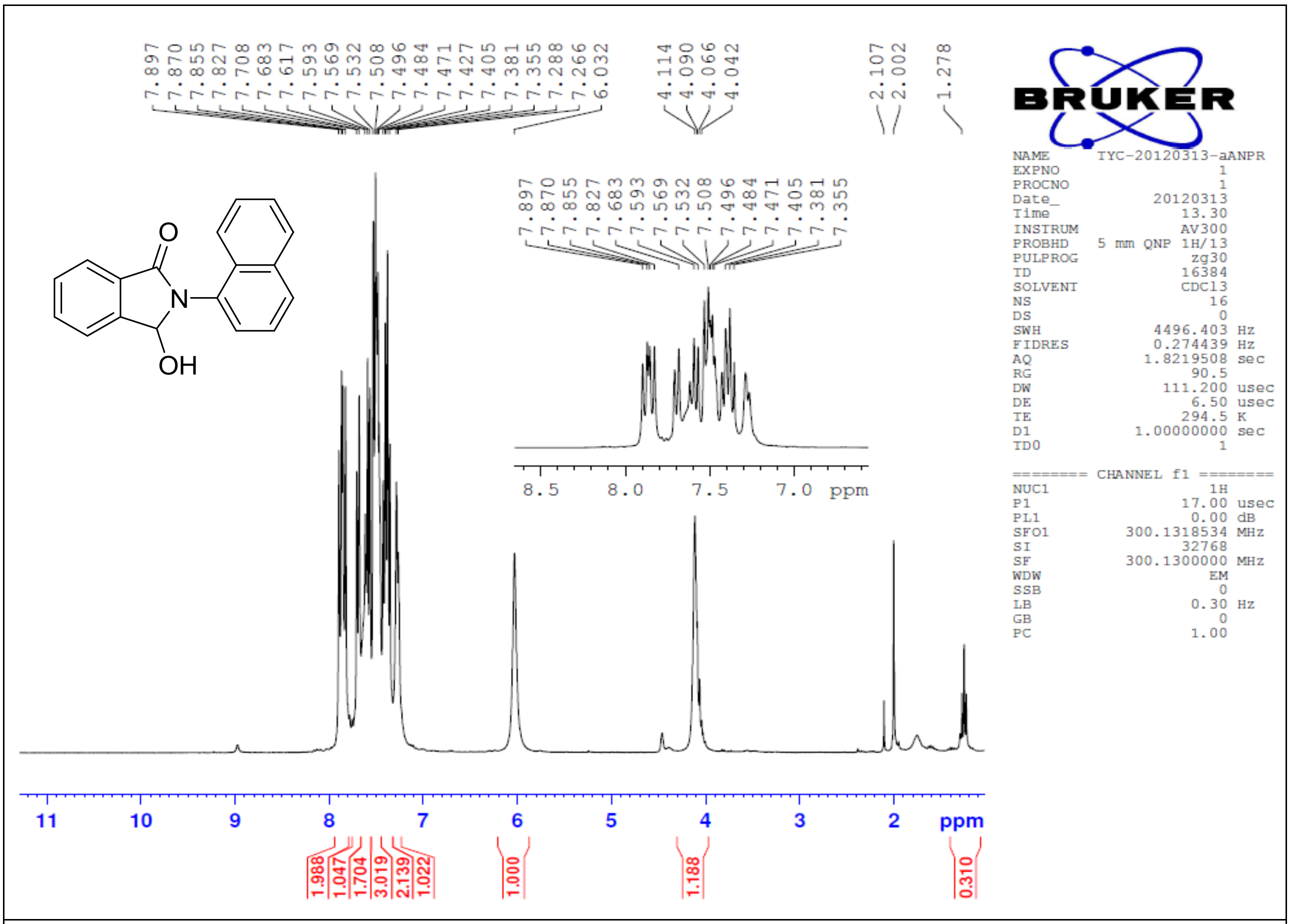
7.897 7.870 7.855 7.827 7.708 7.683 7.617 7.593 7.569 7.532 7.508 7.496 7.484 7.471 7.427 7.405 7.381 7.355 7.288 7.266 6.032
4.114 4.090 4.066 4.042
2.107 2.002
1.278
O
N
OH
BRUKER
NAME TYC-20120313-aANPR
EXPNO 1
PROCNO 1
Date_ 20120313
Time 13.30
INSTRUM AV300
PROBHD 5 mm QNP 1H/13
PULPROG zg30
TD 16384
SOLVENT CDCl3
NS 16
DS 0
SWH 4496.403 Hz
FIDRES 0.274439 Hz
AQ 1.8219508 sec
RG 90.5
DW 111.200 usec
DE 6.50 usec
TE 294.5 K
D1 1.00000000 sec
TD0 1
======== CHANNEL f1 ========
NUC1 1H
P1 17.00 usec
PL1 0.00 dB
SFO1 300.1318534 MHz
SI 32768
SF 300.1300000 MHz
WDW EM
SSB 0
LB 0.30 Hz
GB 0
PC 1.00
7.897 7.870 7.855 7.827 7.683 7.593 7.569 7.532 7.508 7.496 7.484 7.471 7.405 7.381 7.355
8.5 8.0 7.5 7.0 ppm
11 10 9 8 7 6 5 4 3 2 ppm
1.988 1.047 1.704 3.019 2.139 1.022 1.000 1.188 0.310

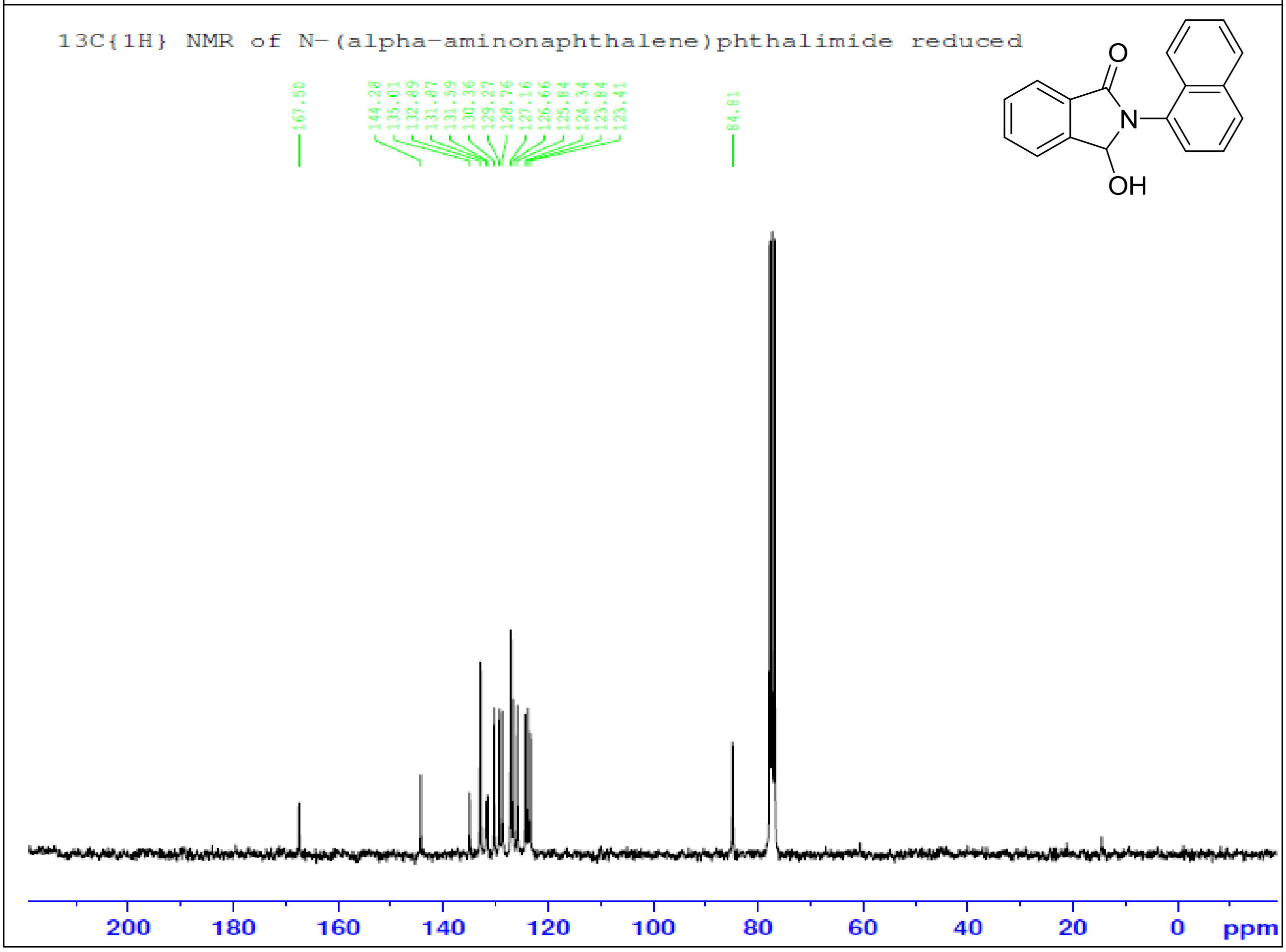
13C{1H} NMR of N-(alpha-aminonaphthalene)phthalimide reduced
167.50
144.28 135.01 132.89 131.87 131.59 130.36 129.27 128.76 127.16 126.66 125.84 124.34 123.84 123.41
84.81
O
N
OH
200 180 160 140 120 100 80 60 40 20 0 ppm

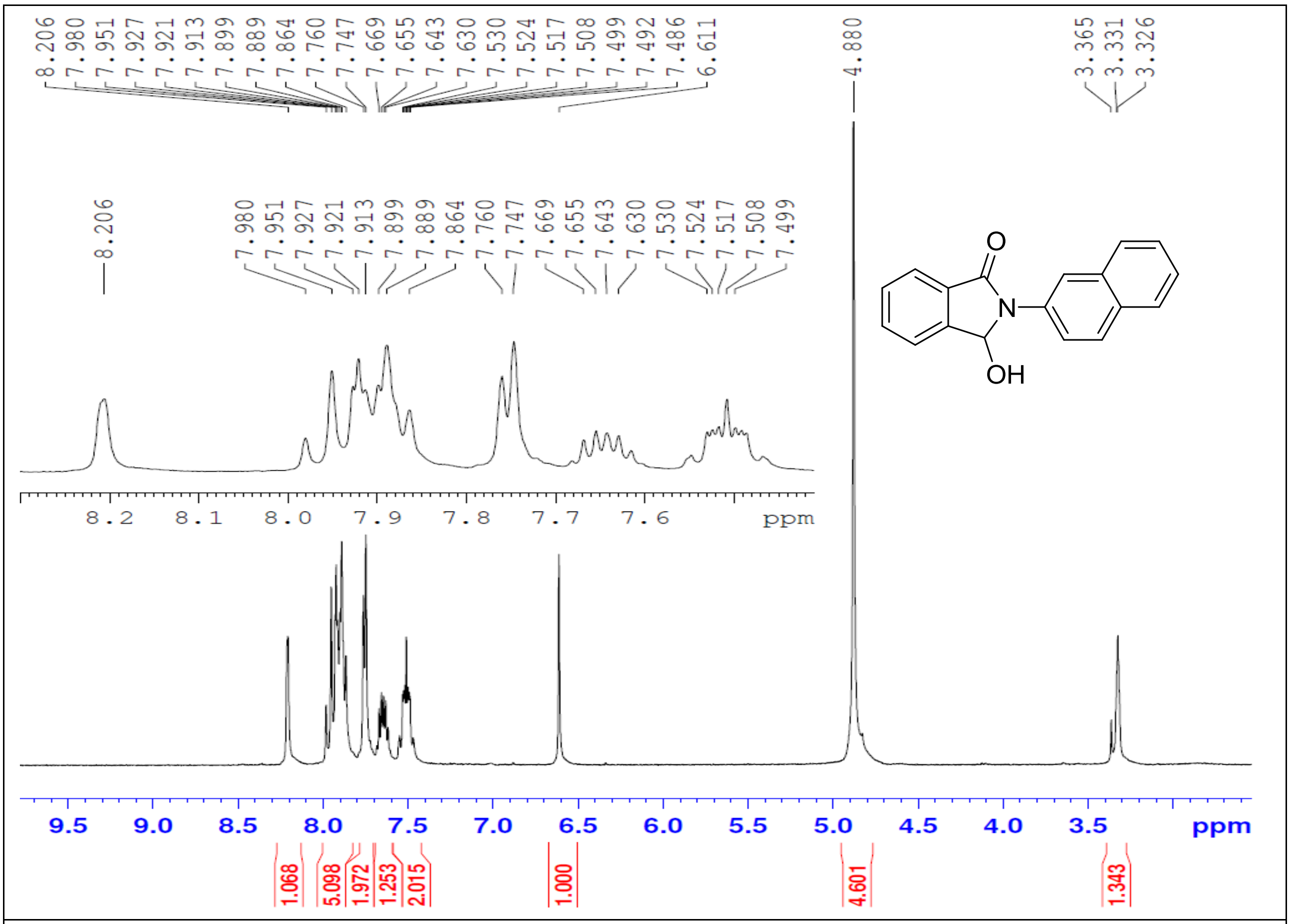
8.206 7.980 7.951 7.927 7.921 7.913 7.899 7.889 7.864 7.760 7.747 7.669 7.655 7.643 7.630 7.530 7.524 7.517 7.508 7.499 7.492 7.486 6.611
4.880
3.365 3.331 3.326
O
N
OH
ppm
1.068
5.098
1.972
1.253
2.015
1.000
4.601
1.343

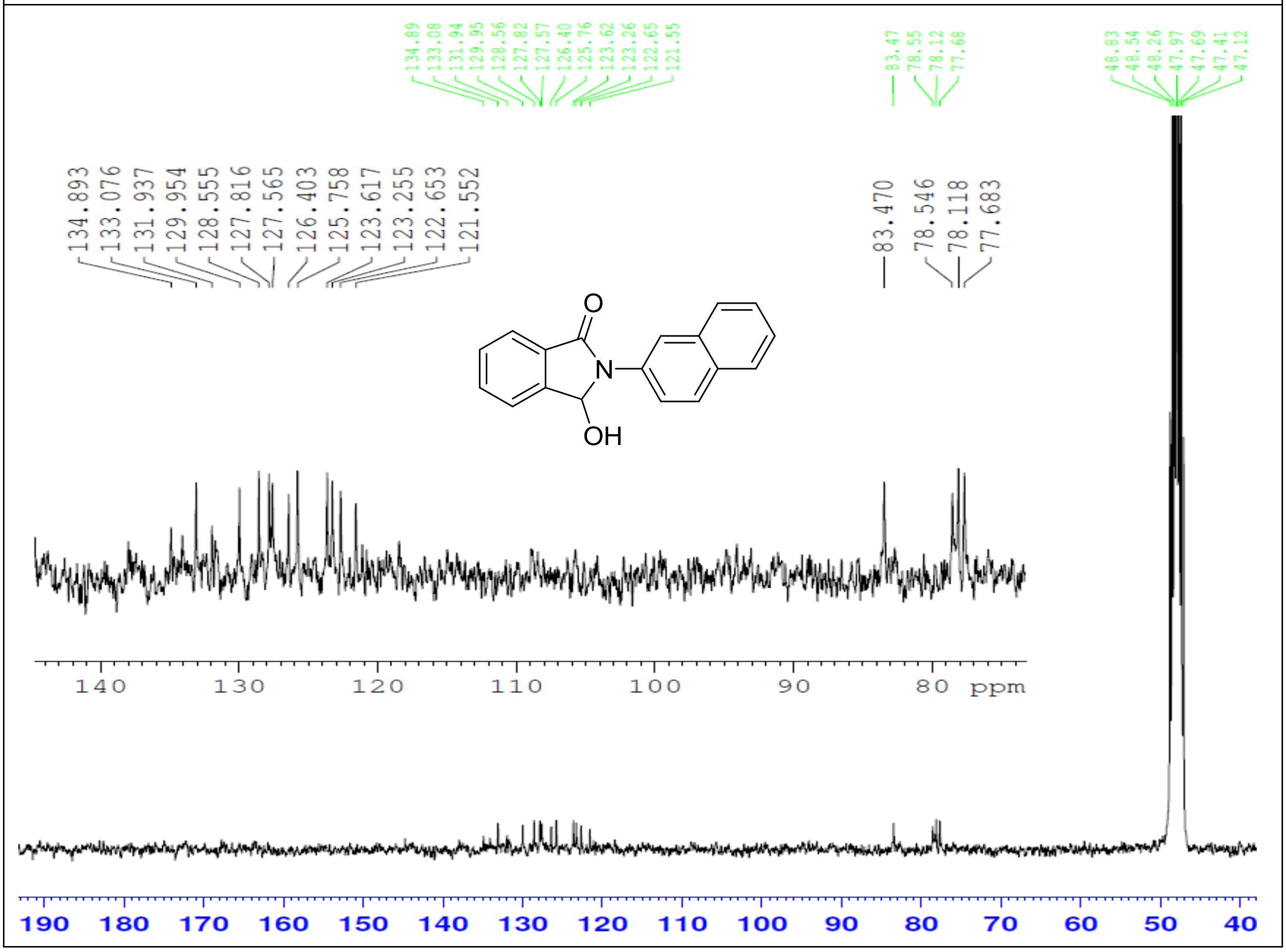
134.89 133.08 131.94 129.95 128.56 127.82 127.57 126.40 125.76 123.62 123.26 122.65 121.55
83.47 78.55 78.12 77.68
48.83 48.54 48.26 47.97 47.69 47.41 47.12
134.893 133.076 131.937 129.954 128.555 127.816 127.565 126.403 125.758 123.617 123.255 122.653 121.552
83.470 78.546 78.118 77.683
O
N
OH
ppm

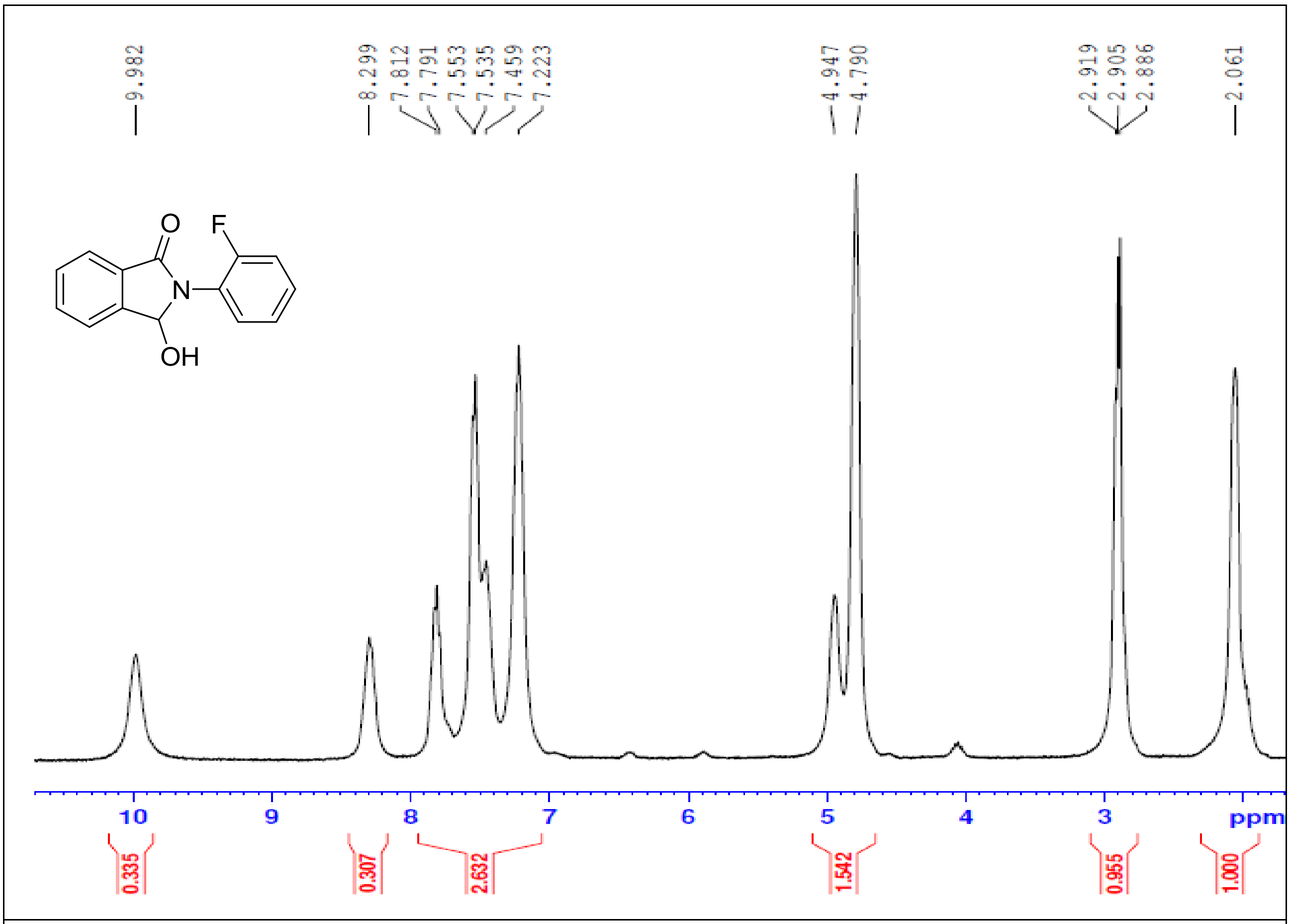
9.982
8.299
7.812
7.791
7.553
7.535
7.459
7.223
4.947
4.790
2.919
2.905
2.886
2.061
O
F
N
OH
10
9
8
7
6
5
4
3
ppm
0.335
0.307
2.632
1.542
0.955
1.000

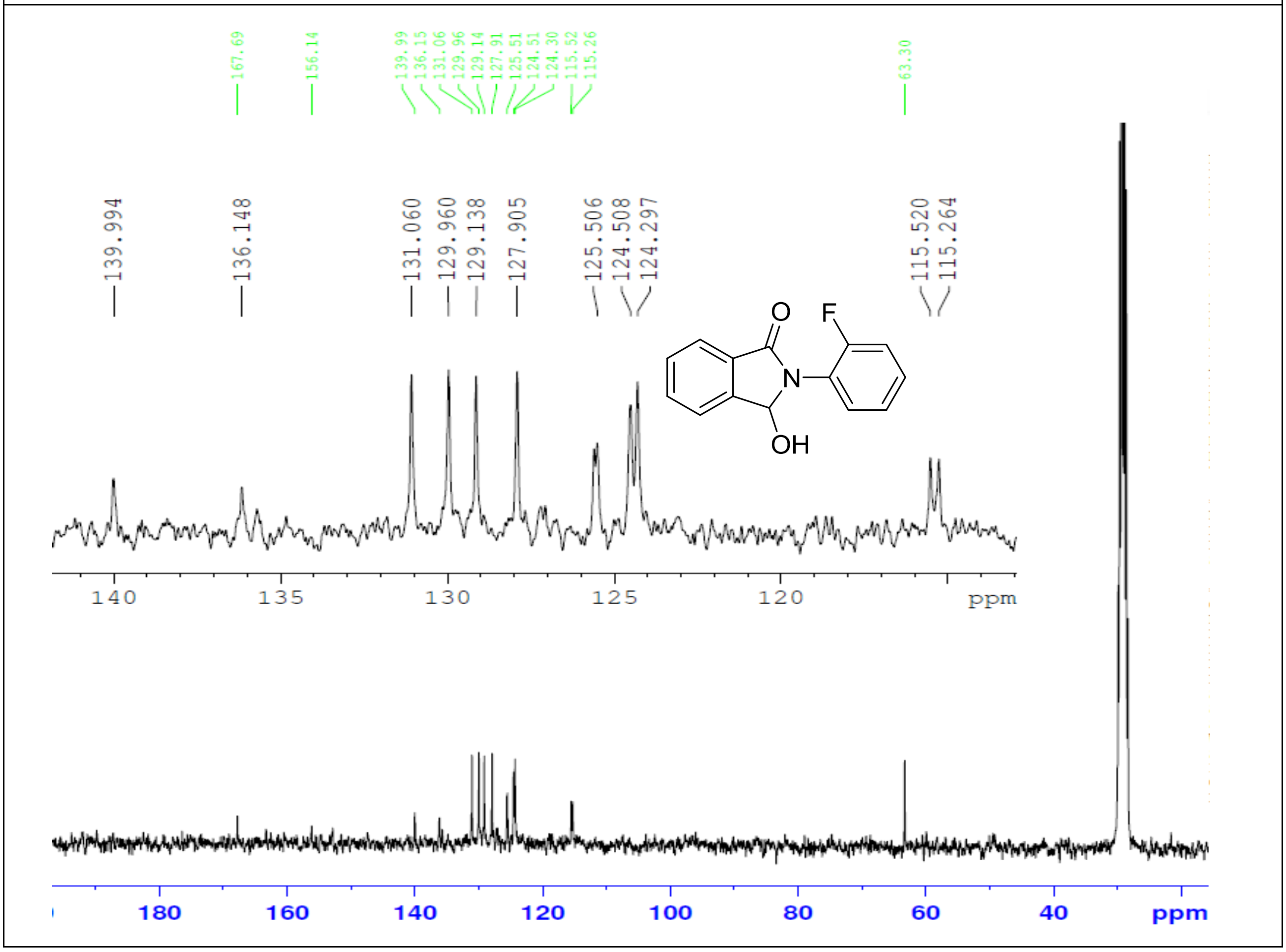
167.69
156.14
139.99
136.15
131.06
129.96
129.14
127.91
125.51
124.51
124.30
115.52
115.26
63.30
139.994
136.148
131.060
129.960
129.138
127.905
125.506
124.508
124.297
115.520
115.264
O
F
N
OH
140
135
130
125
120
ppm
180
160
140
120
100
80
60
40
ppm

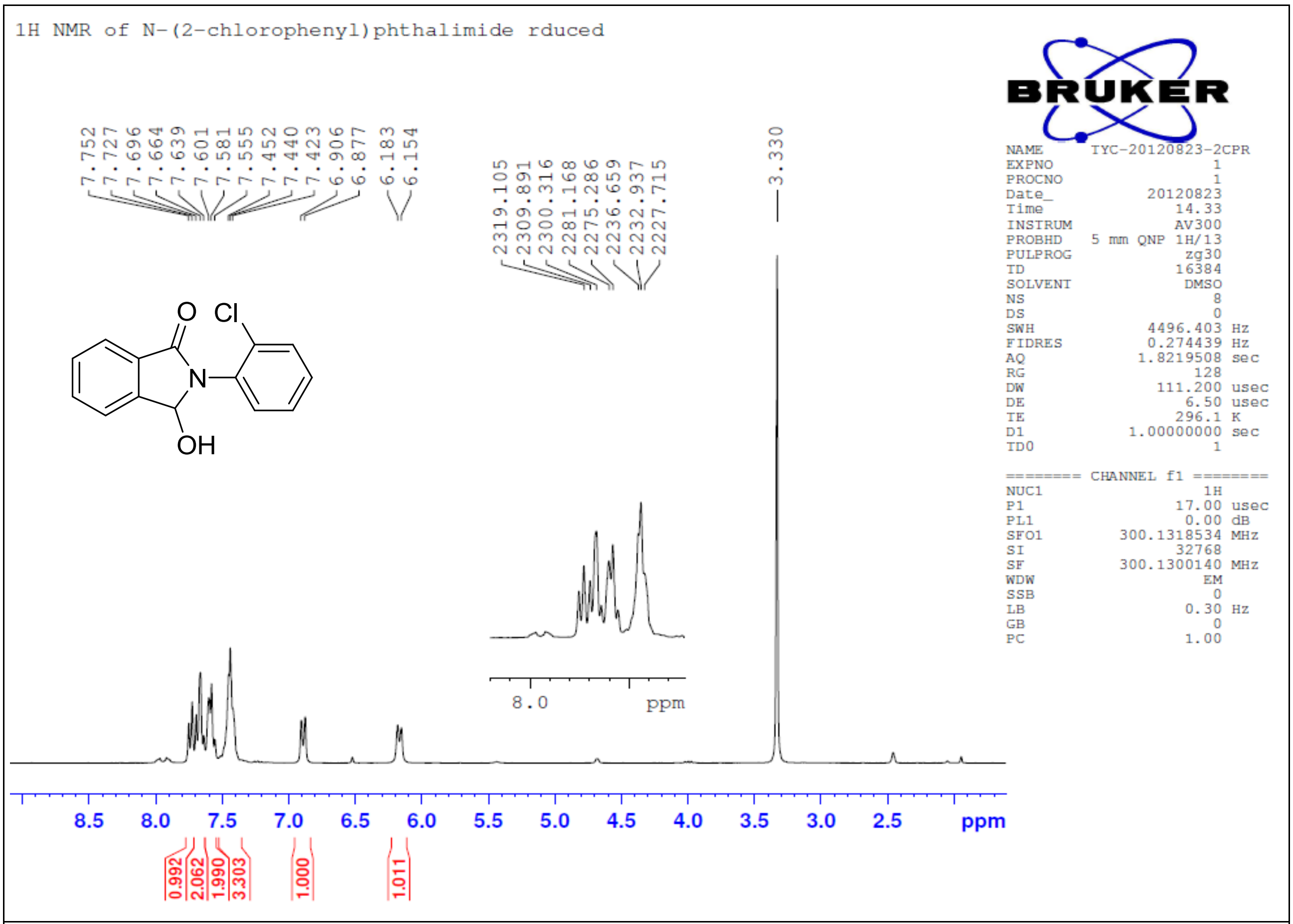

1H NMR of N-(2-chlorophenyl)phthalimide rduced
7.752
7.727
7.696
7.664
7.639
7.601
7.581
7.555
7.452
7.440
7.423
6.906
6.877
6.183
6.154
2319.105
2309.891
2300.316
2281.168
2275.286
2236.659
2232.937
2227.715
3.330
O
Cl
N
OH
BRUKER
NAME TYC-20120823-2CPR
EXPNO 1
PROCNO 1
Date_ 20120823
Time 14.33
INSTRUM AV300
PROBHD 5 mm QNP 1H/13
PULPROG zg30
TD 16384
SOLVENT DMSO
NS 8
DS 0
SWH 4496.403 Hz
FIDRES 0.274439 Hz
AQ 1.8219508 sec
RG 128
DW 111.200 usec
DE 6.50 usec
TE 296.1 K
D1 1.00000000 sec
TD0 1
======== CHANNEL f1 ========
NUC1 1H
P1 17.00 usec
PL1 0.00 dB
SFO1 300.1318534 MHz
SI 32768
SF 300.1300140 MHz
WDW EM
SSB 0
LB 0.30 Hz
GB 0
PC 1.00
8.0
ppm
8.5
8.0
7.5
7.0
6.5
6.0
5.5
5.0
4.5
4.0
3.5
3.0
2.5
ppm
0.992
2.062
1.990
3.303
1.000
1.011

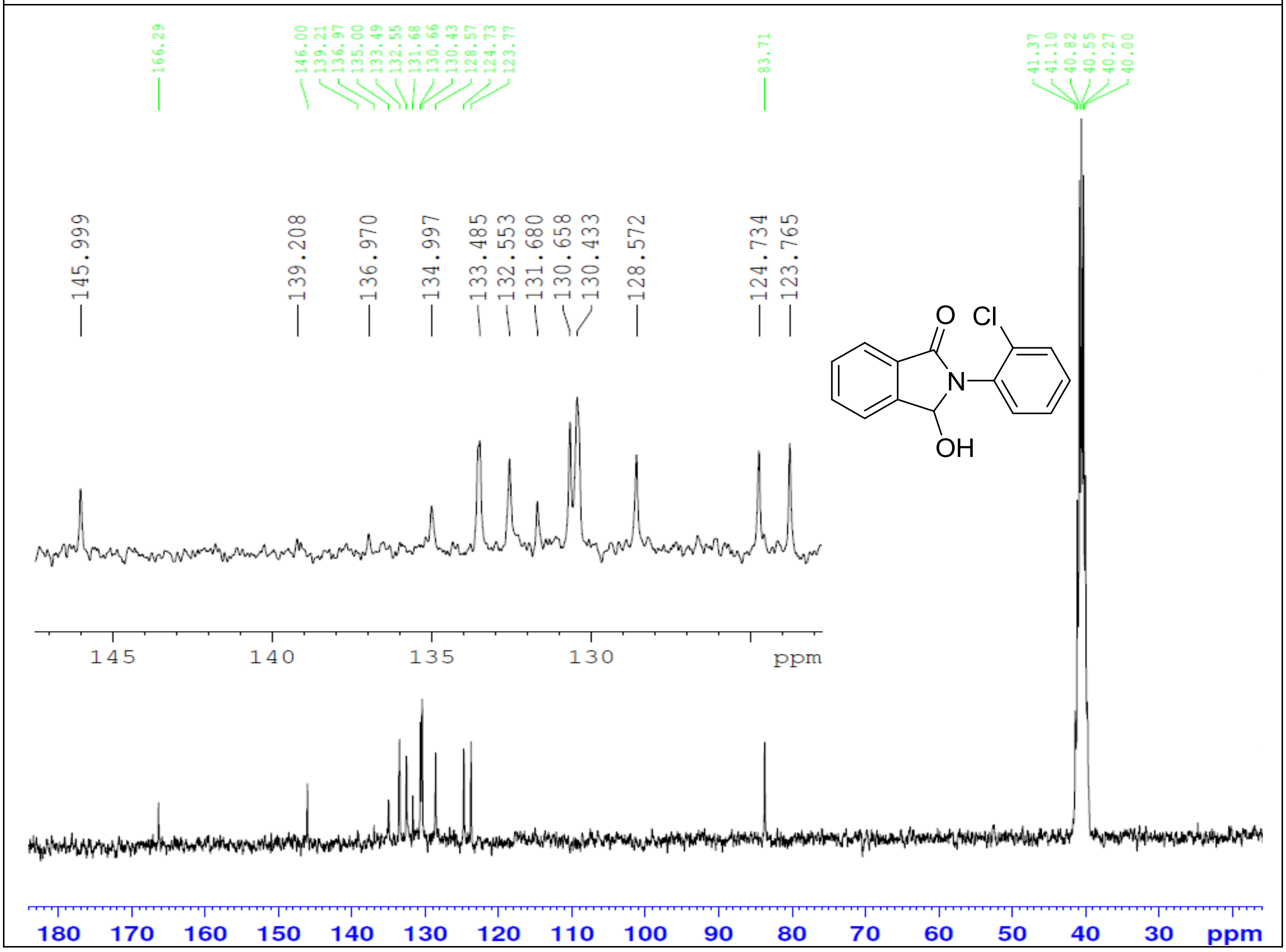

166.29
146.00
139.21
136.97
135.00
133.49
132.55
131.68
130.66
130.43
128.57
124.73
123.77
83.71
41.37
41.10
40.82
40.55
40.27
40.00
145.999
139.208
136.970
134.997
133.485
132.553
131.680
130.658
130.433
128.572
124.734
123.765
O
Cl
N
OH
145
140
135
130
ppm
180
170
160
150
140
130
120
110
100
90
80
70
60
50
40
30
ppm

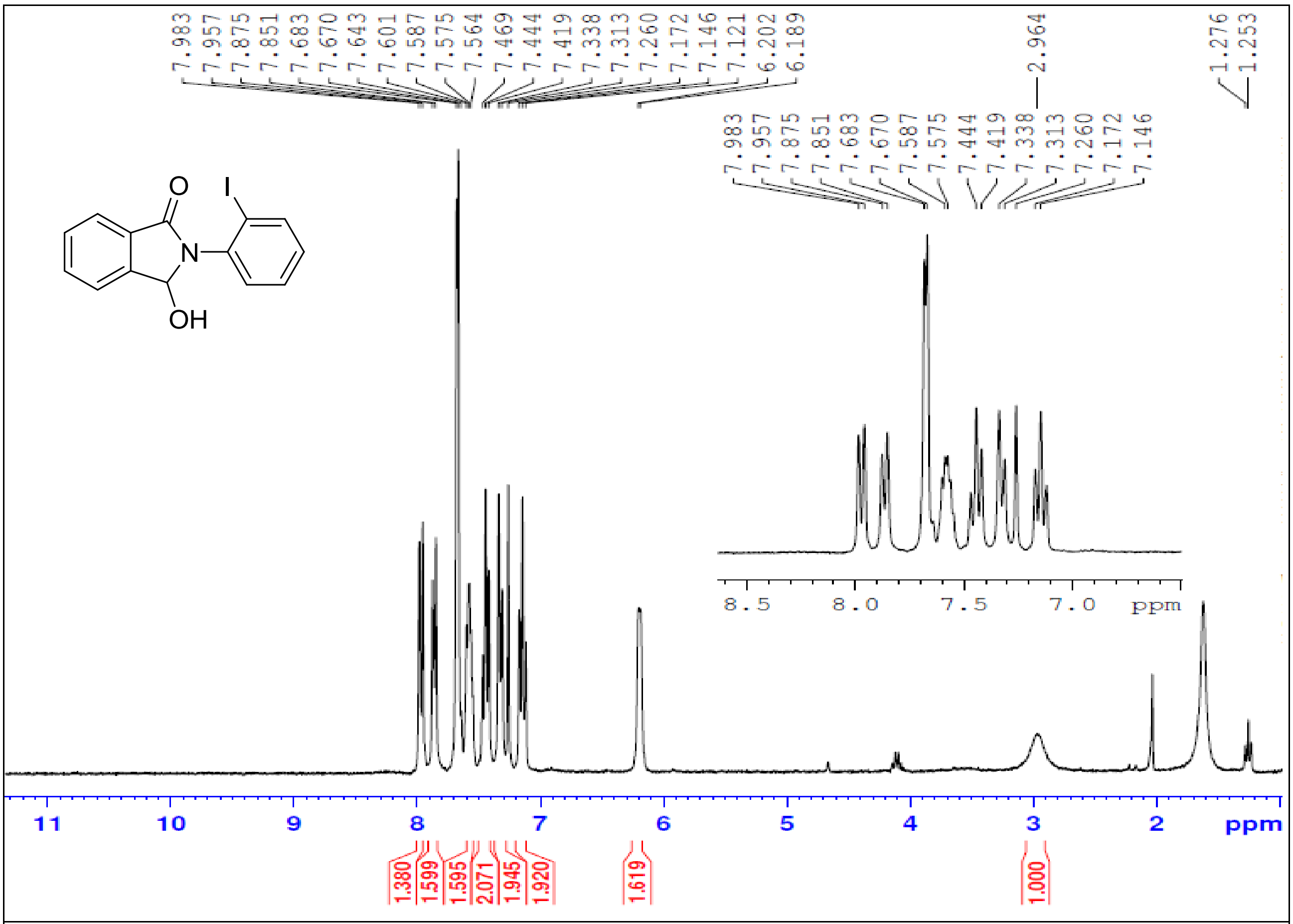
7.983
7.957
7.875
7.851
7.683
7.670
7.643
7.601
7.587
7.575
7.564
7.469
7.444
7.419
7.338
7.313
7.260
7.172
7.146
7.121
6.202
6.189
2.964
1.276
1.253
O
I
N
OH
8.5
8.0
7.5
7.0
ppm
11
10
9
8
7
6
5
4
3
2
ppm
1.380
1.599
1.595
2.071
1.945
1.920
1.619
1.000

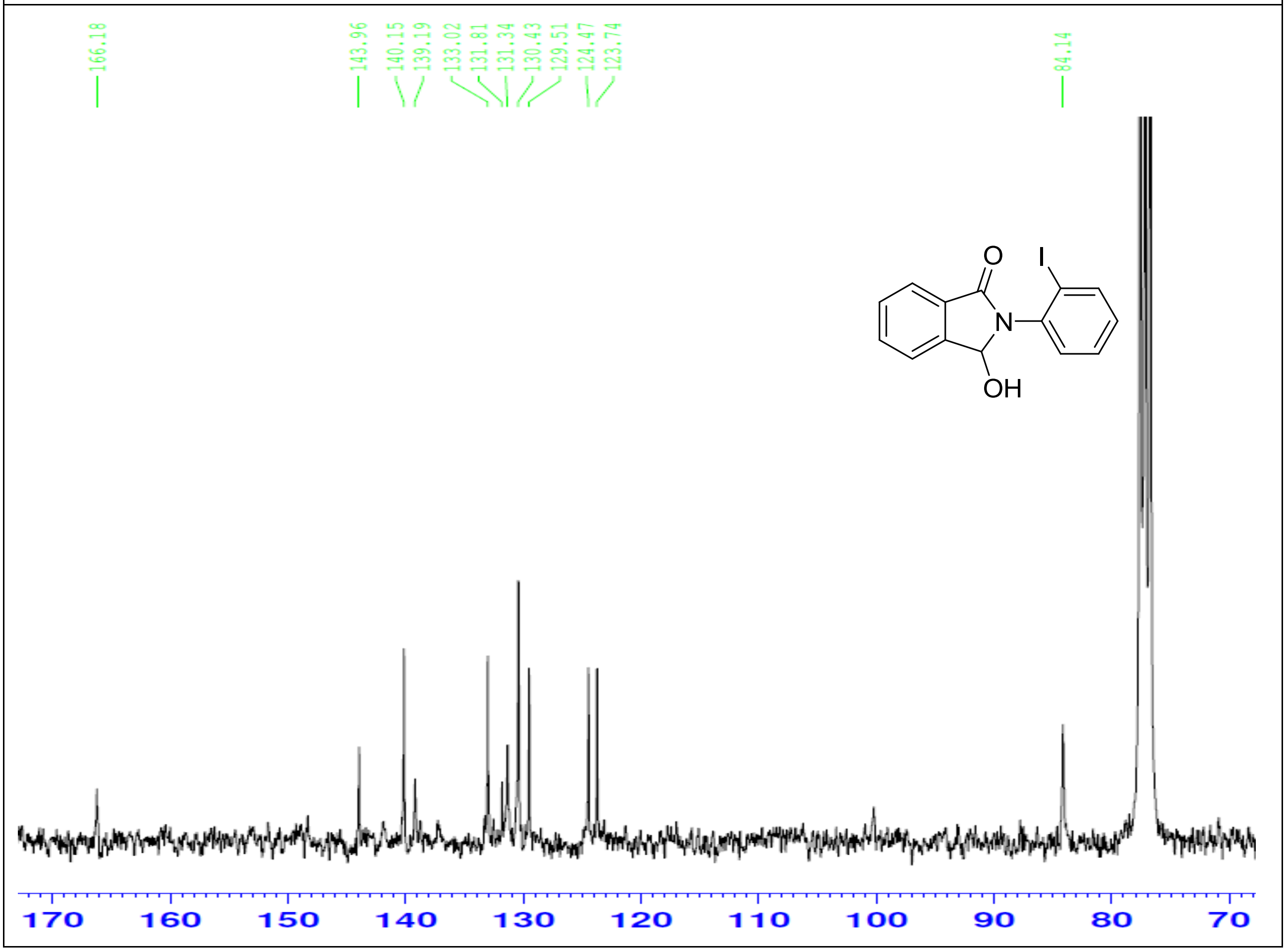
166.18
143.96
140.15
139.19
133.02
131.81
131.34
130.43
129.51
124.47
123.74
84.14
O
I
N
OH
170
160
150
140
130
120
110
100
90
80
70

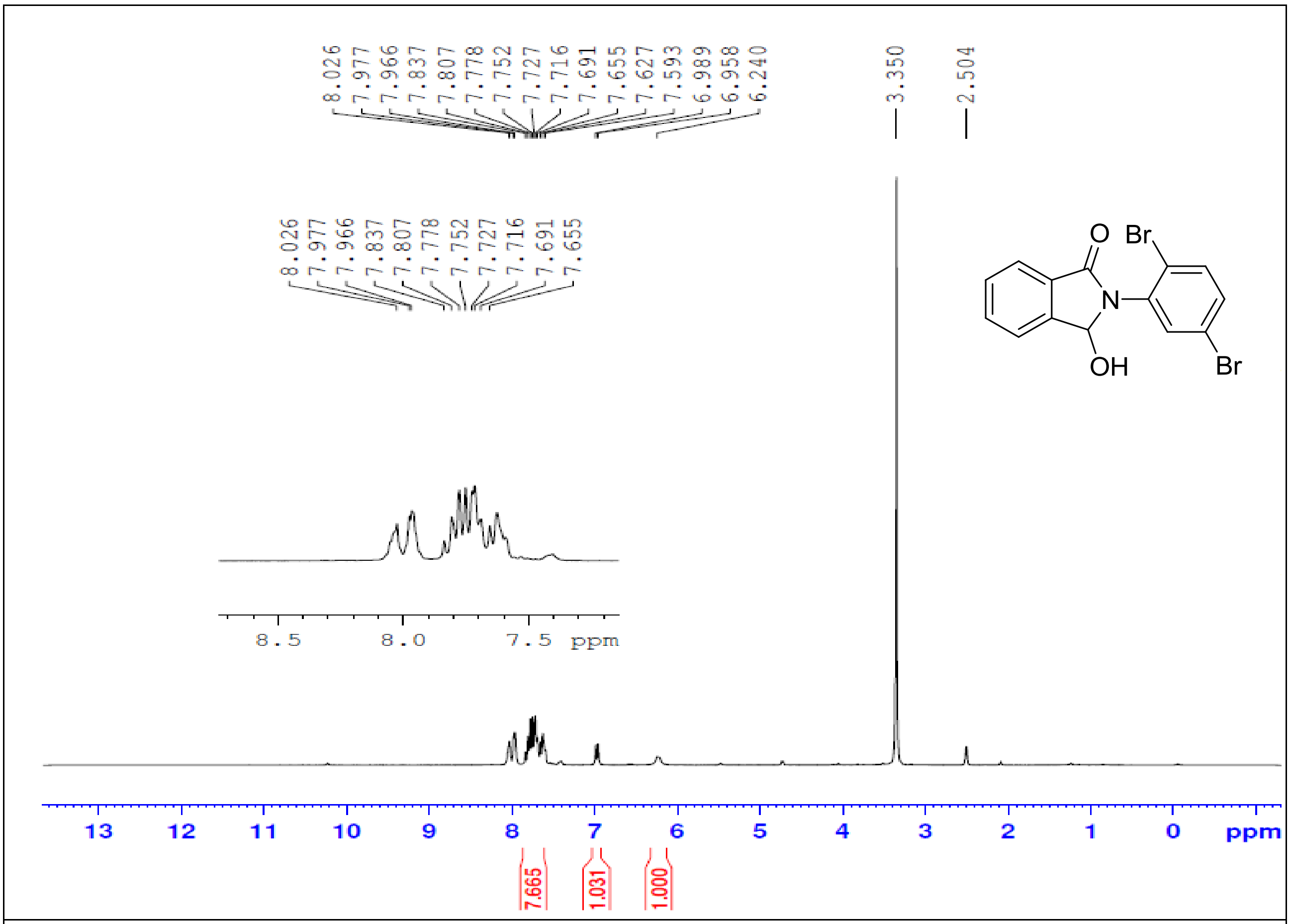

8.026
7.977
7.966
7.837
7.807
7.778
7.752
7.727
7.716
7.691
7.655
7.627
7.593
6.989
6.958
6.240
3.350
2.504
8.5
8.0
7.5
ppm
13
12
11
10
9
8
7
6
5
4
3
2
1
0
ppm
7.665
1.031
1.000
O
Br
N
OH
Br

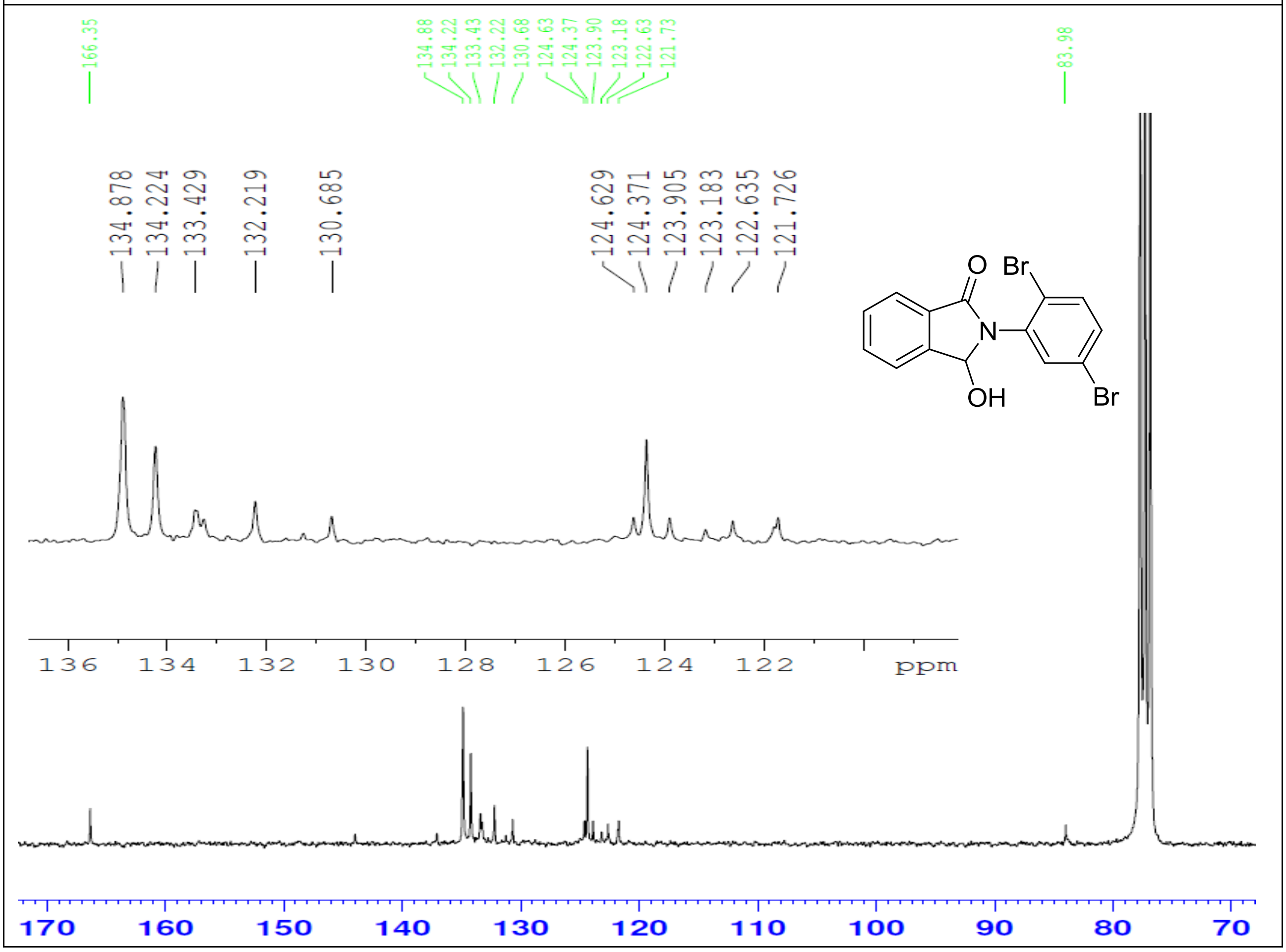

166.35
134.88
134.22
133.43
132.22
130.68
124.63
124.37
123.90
123.18
122.63
121.73
83.98
134.878
134.224
133.429
132.219
130.685
124.629
124.371
123.905
123.183
122.635
121.726
136
134
132
130
128
126
124
122
ppm
O
Br
N
OH
Br
170
160
150
140
130
120
110
100
90
80
70

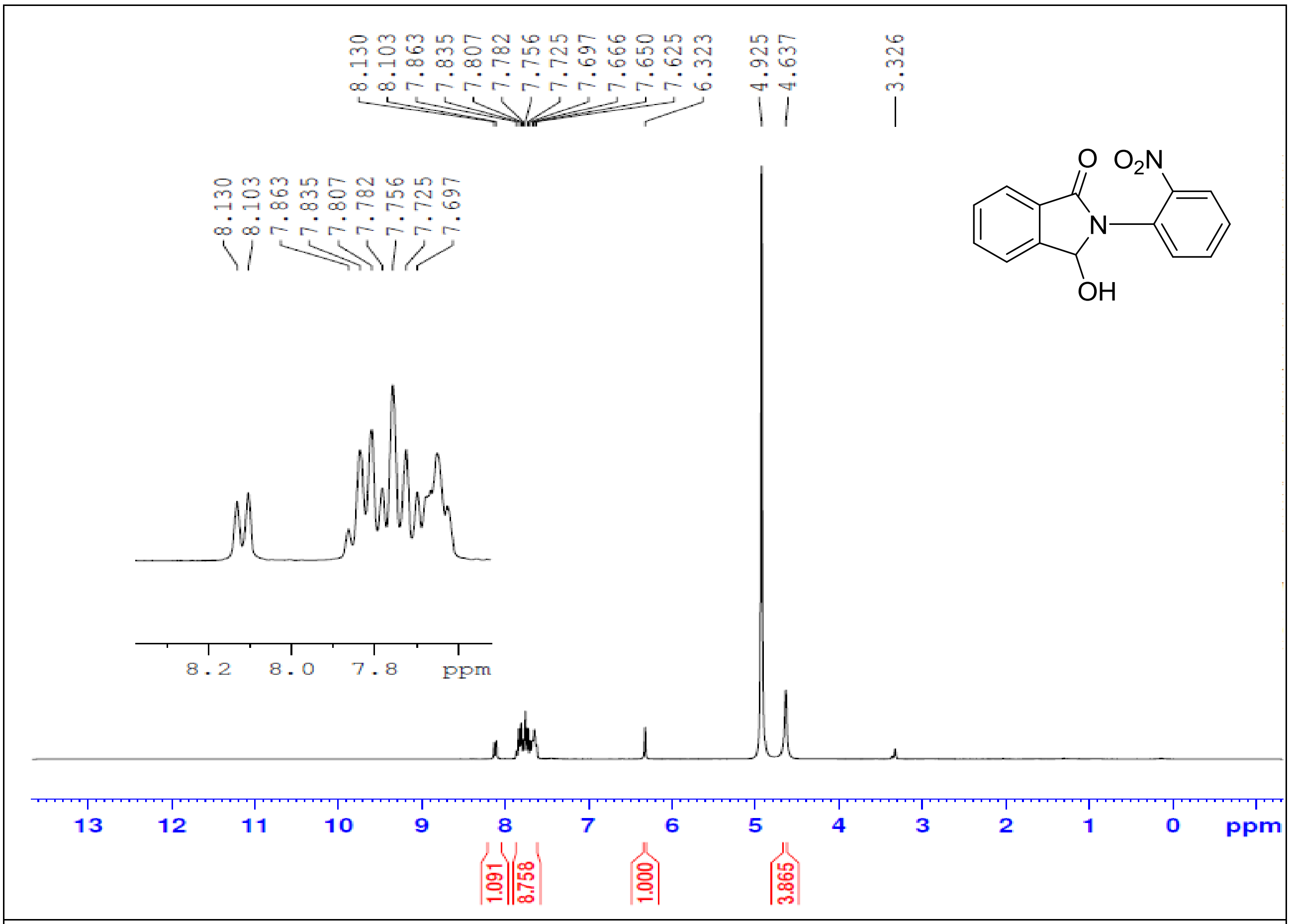
8.130
8.103
7.863
7.835
7.807
7.782
7.756
7.725
7.697
7.666
7.650
7.625
6.323
4.925
4.637
3.326
8.2
8.0
7.8
ppm
O
O2N
N
OH
13
12
11
10
9
8
7
6
5
4
3
2
1
0
ppm
1.091
8.758
1.000
3.865

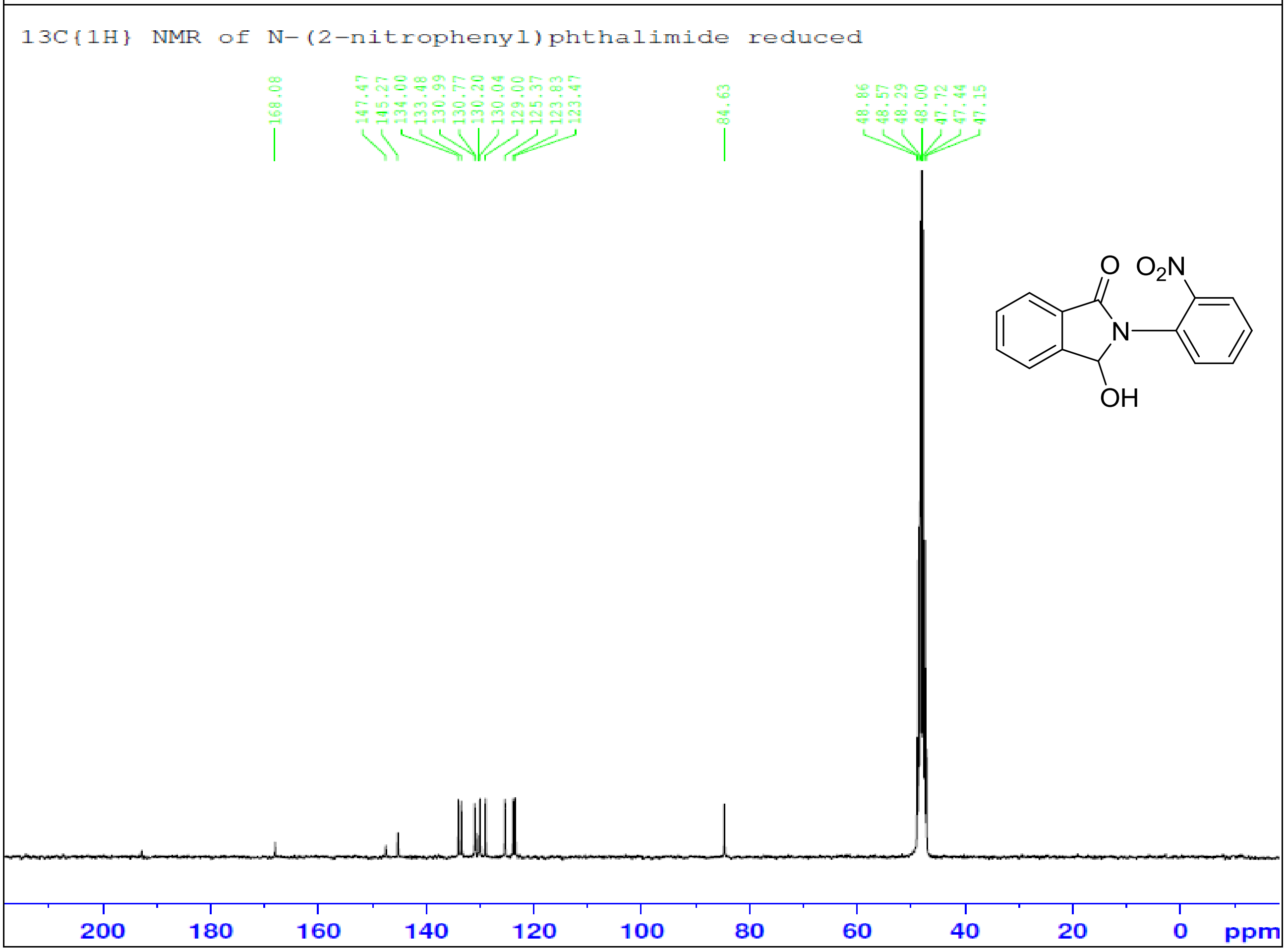
13C{1H} NMR of N-(2-nitrophenyl)phthalimide reduced
168.08
147.47
145.27
134.00
133.48
130.99
130.77
130.20
130.04
129.00
125.37
123.83
123.47
84.63
48.86
48.57
48.29
48.00
47.72
47.44
47.15
O
O2N
N
OH
200
180
160
140
120
100
80
60
40
20
0
ppm

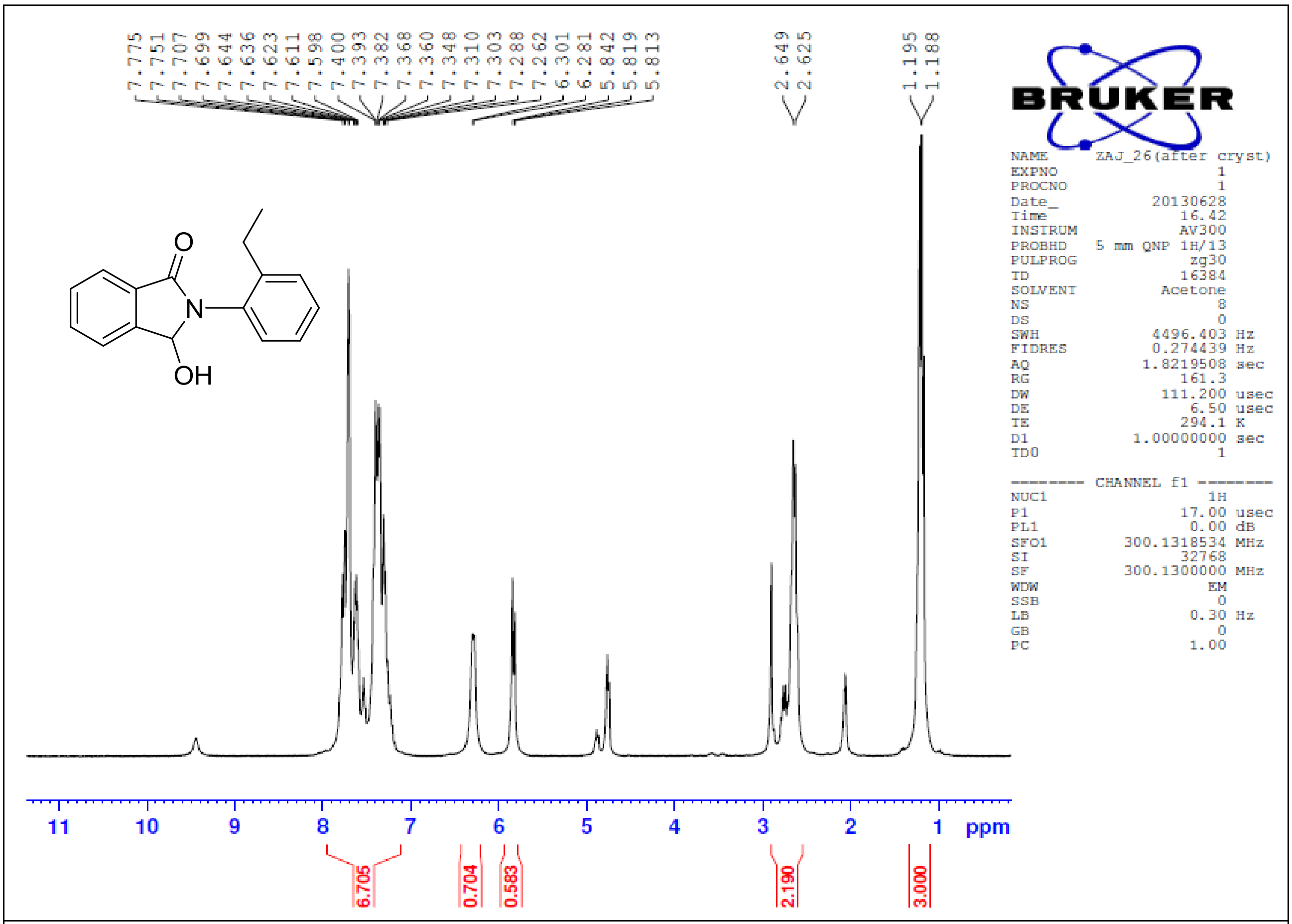
7.775
7.751
7.707
7.699
7.644
7.636
7.623
7.611
7.598
7.400
7.393
7.382
7.368
7.360
7.348
7.310
7.303
7.288
7.262
6.301
6.281
5.842
5.819
5.813
2.649
2.625
1.195
1.188
BRUKER
NAME ZAJ_26(after cryst)
EXPNO 1
PROCNO 1
Date_ 20130628
Time 16.42
INSTRUM AV300
PROBHD 5 mm QNP 1H/13
PULPROG zg30
TD 16384
SOLVENT Acetone
NS 8
DS 0
SWH 4496.403 Hz
FIDRES 0.274439 Hz
AQ 1.8219508 sec
RG 161.3
DW 111.200 usec
DE 6.50 usec
TE 294.1 K
D1 1.00000000 sec
TD0 1
======== CHANNEL f1 ========
NUC1 1H
P1 17.00 usec
PL1 0.00 dB
SFO1 300.1318534 MHz
SI 32768
SF 300.1300000 MHz
WDW EM
SSB 0
LB 0.30 Hz
GB 0
PC 1.00
O
N
OH
11
10
9
8
7
6
5
4
3
2
1
ppm
6.705
0.704
0.583
2.190
3.000

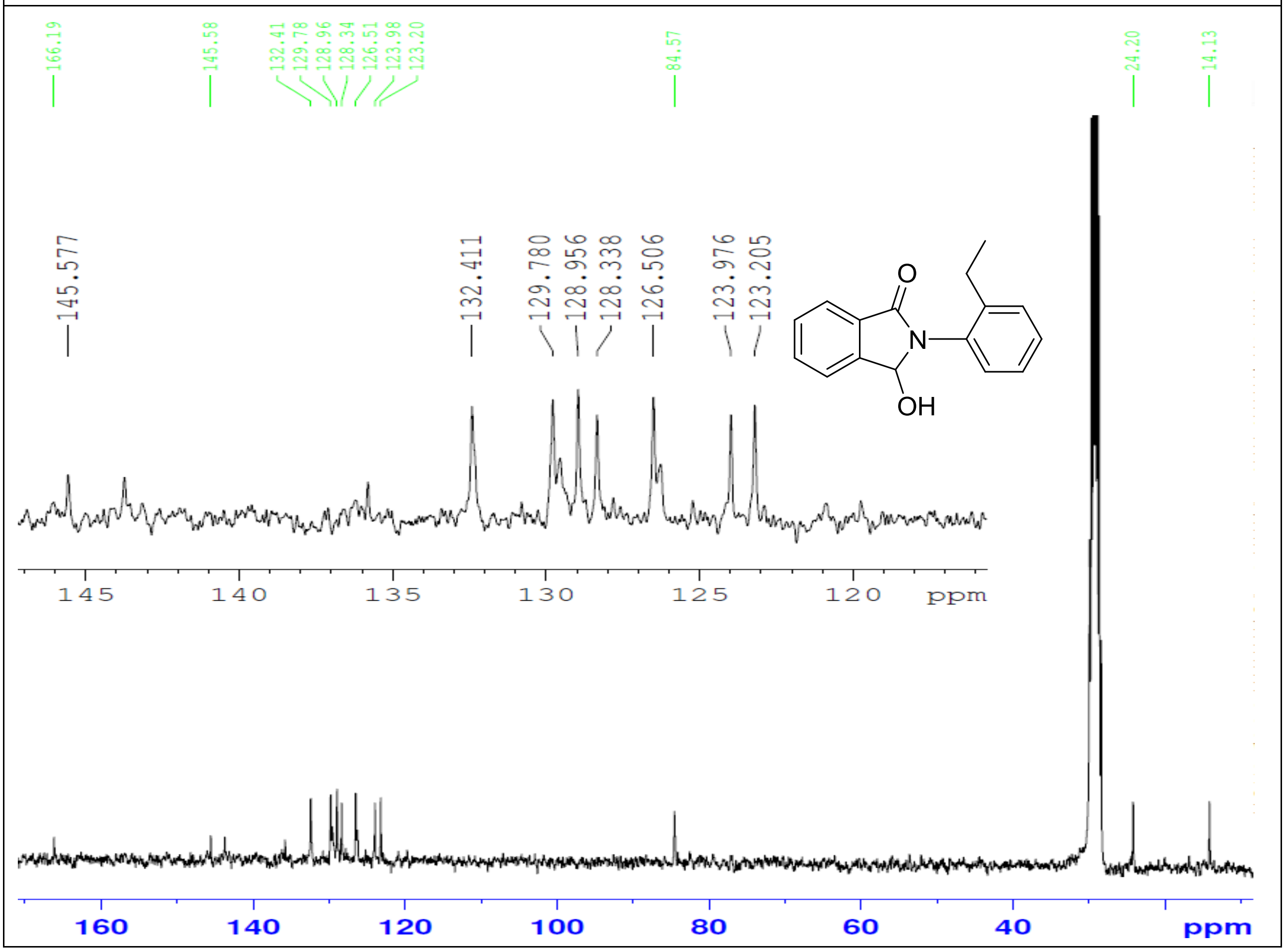
166.19
145.58
132.41
129.78
128.96
128.34
126.51
123.98
123.20
84.57
24.20
14.13
145.577
132.411
129.780
128.956
128.338
126.506
123.976
123.205
O
N
OH
145
140
135
130
125
120
ppm
160
140
120
100
80
60
40
ppm

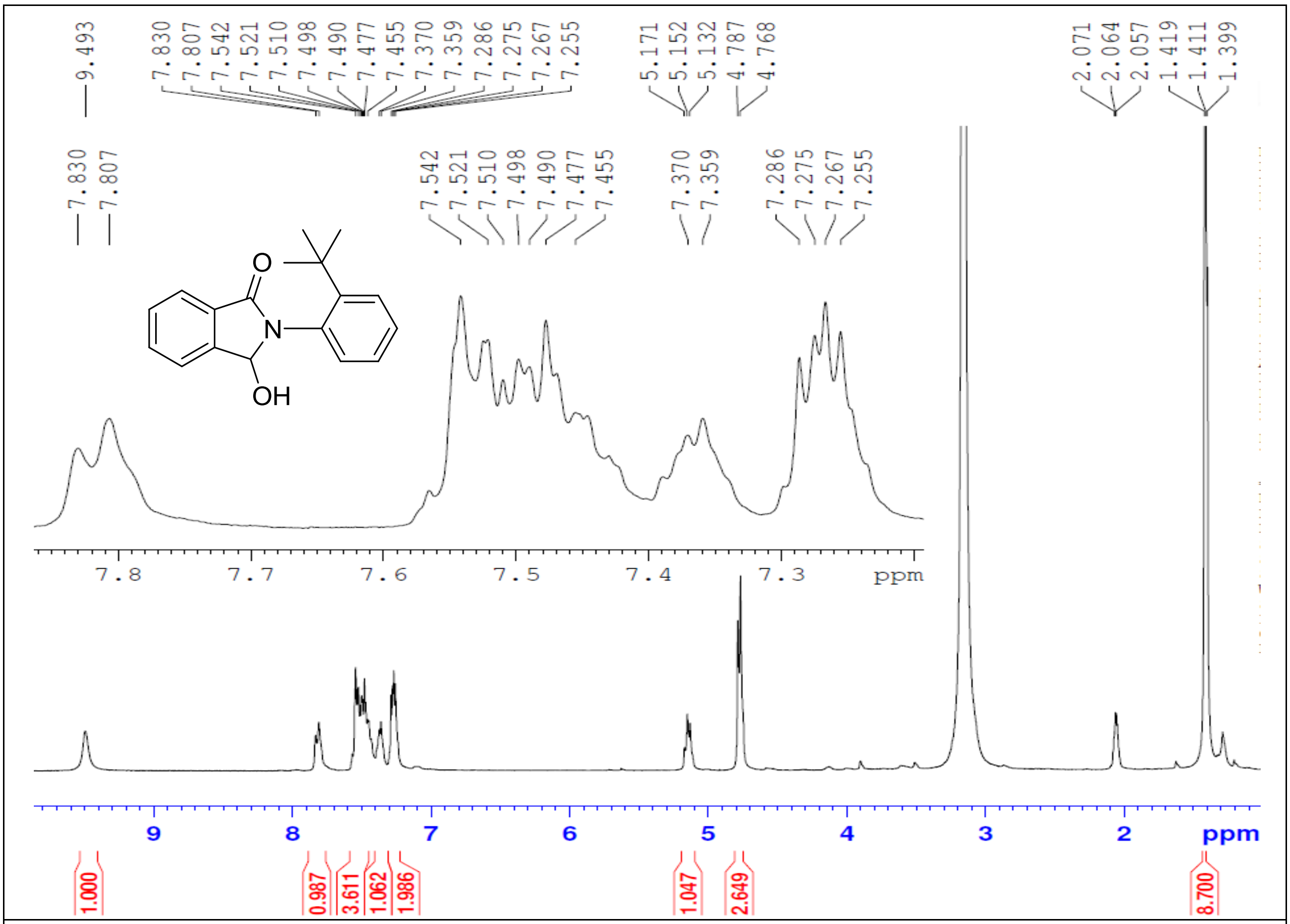

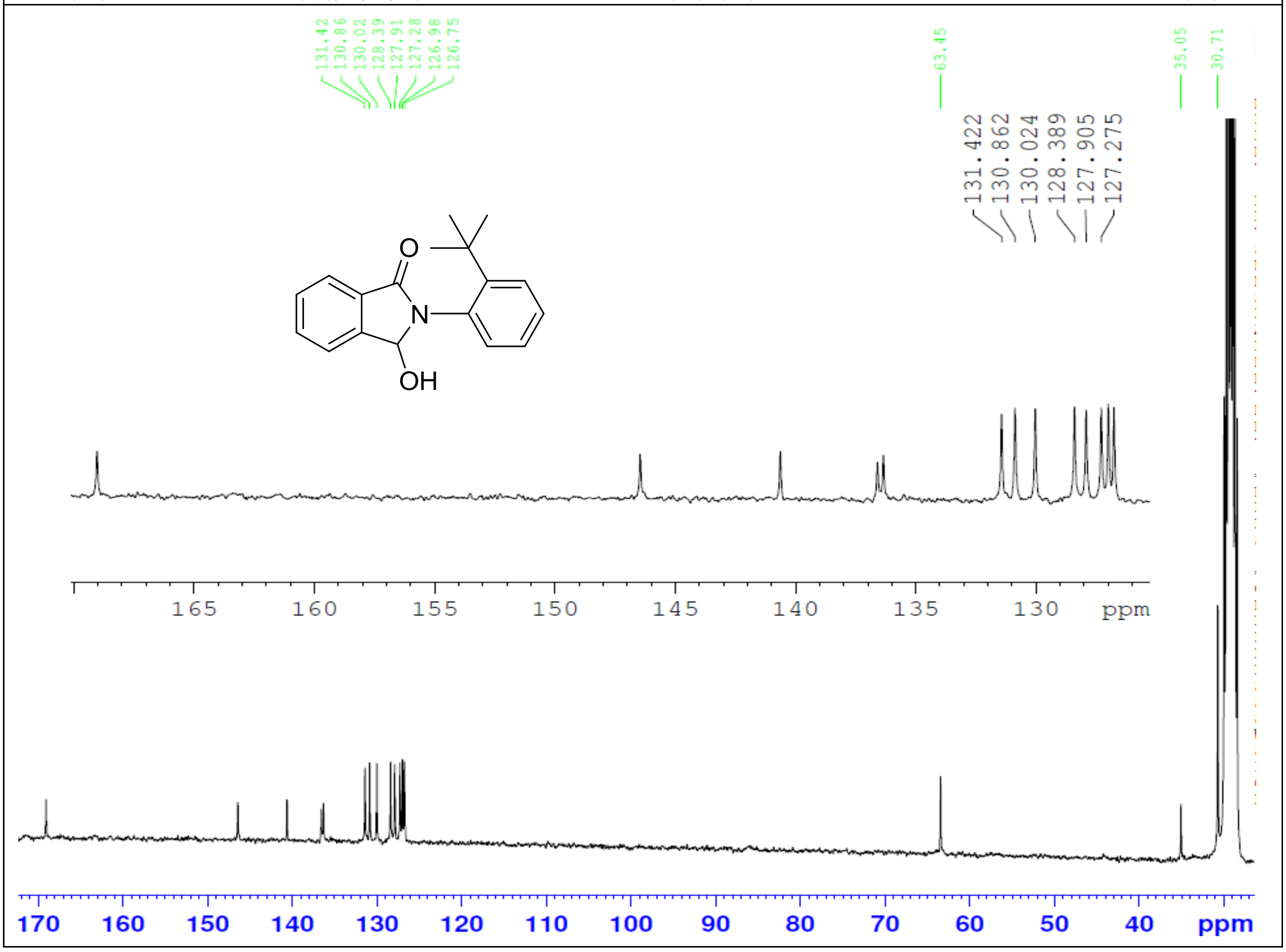

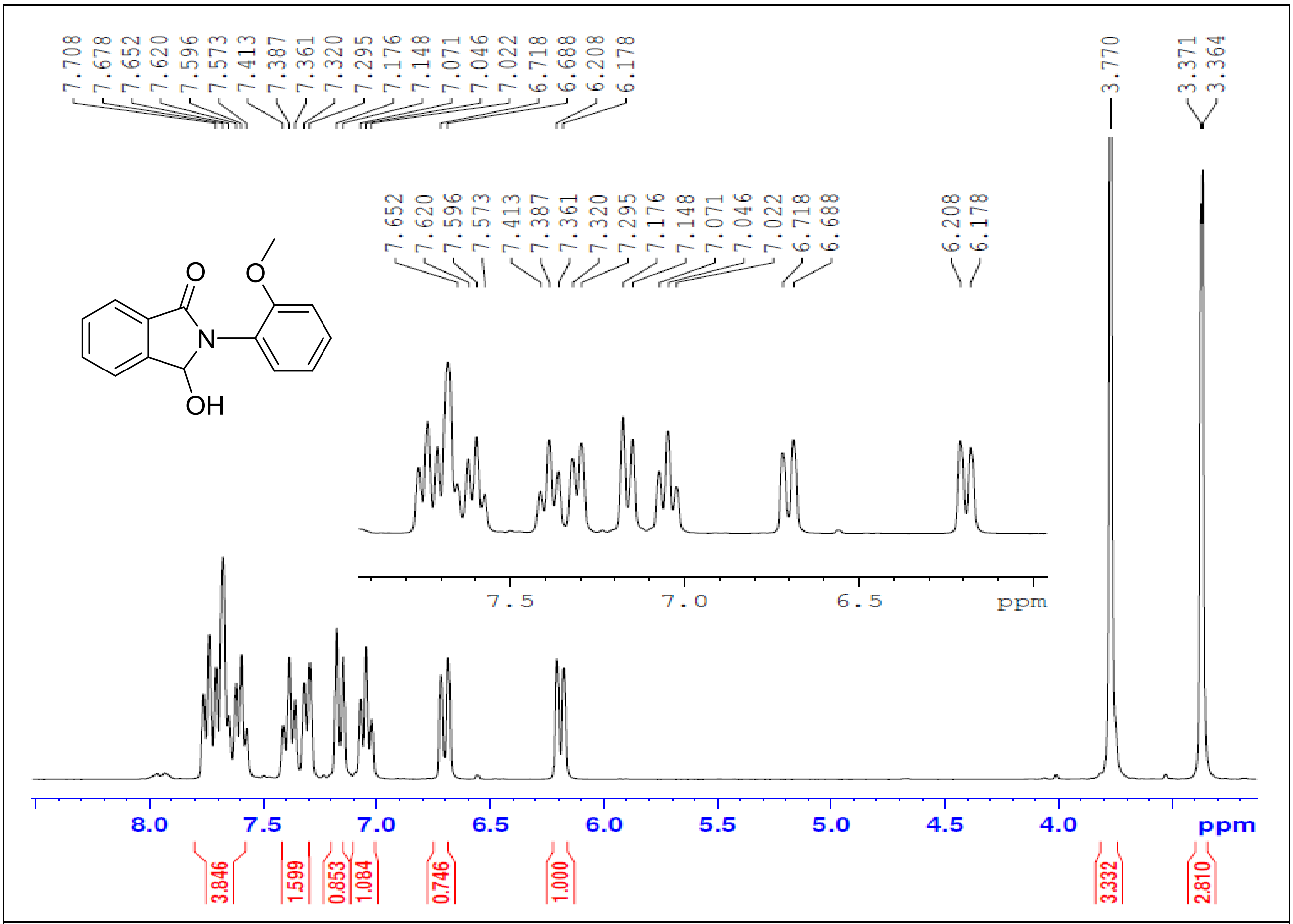
7.708 7.678 7.652 7.620 7.596 7.573 7.413 7.387 7.361 7.320 7.295 7.176 7.148 7.071 7.046 7.022 6.718 6.688 6.208 6.178
3.770
3.371 3.364
OH
ppm
3.846 1.599 0.853 1.084 0.746 1.000 3.332 2.810

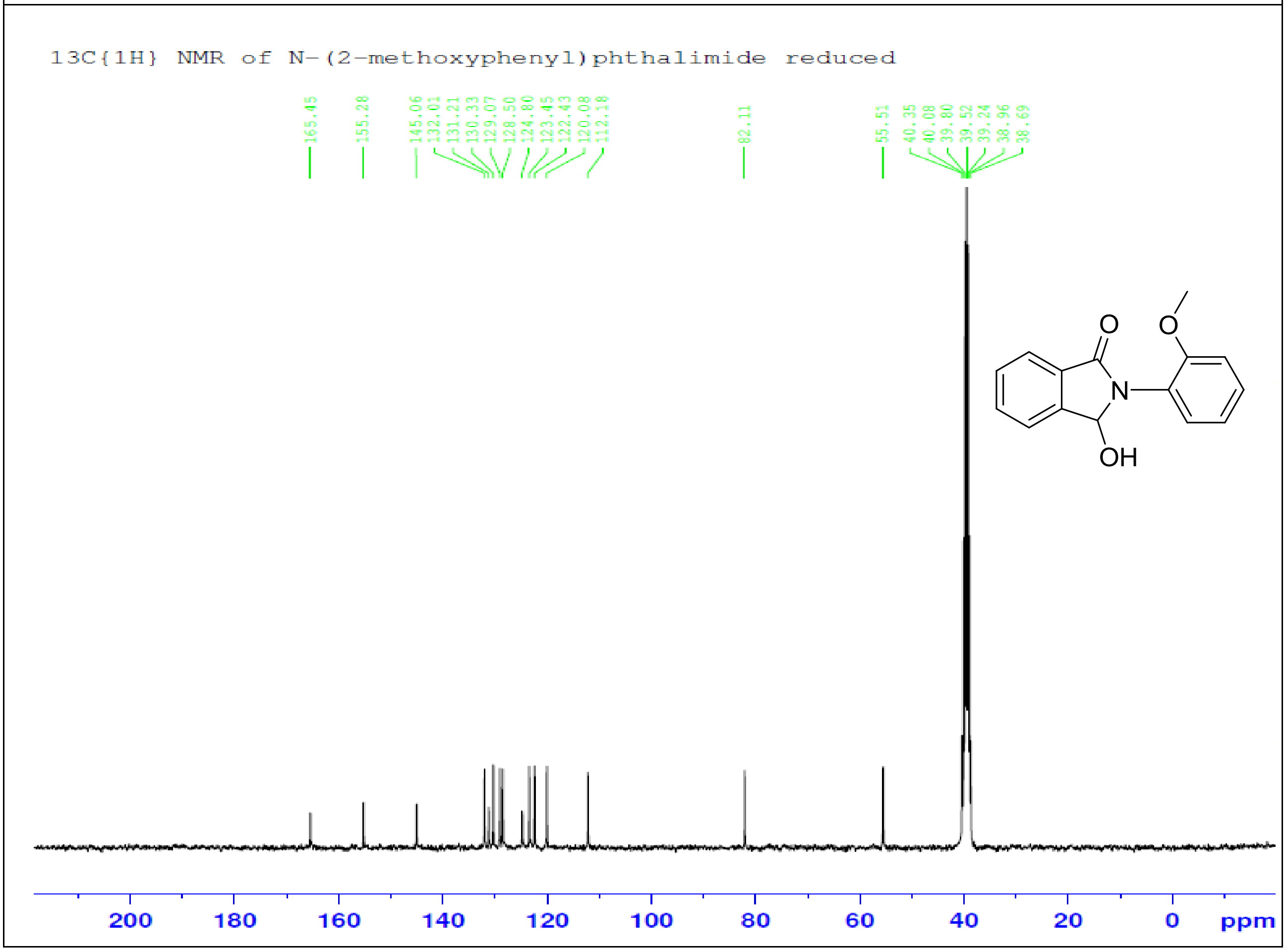
13C{1H} NMR of N-(2-methoxyphenyl)phthalimide reduced
165.45 155.28 145.06 132.01 131.21 130.33 129.07 128.50 124.80 123.45 122.43 120.08 112.18 82.11 55.51 40.35 40.08 39.80 39.52 39.24 38.96 38.69
OH
ppm

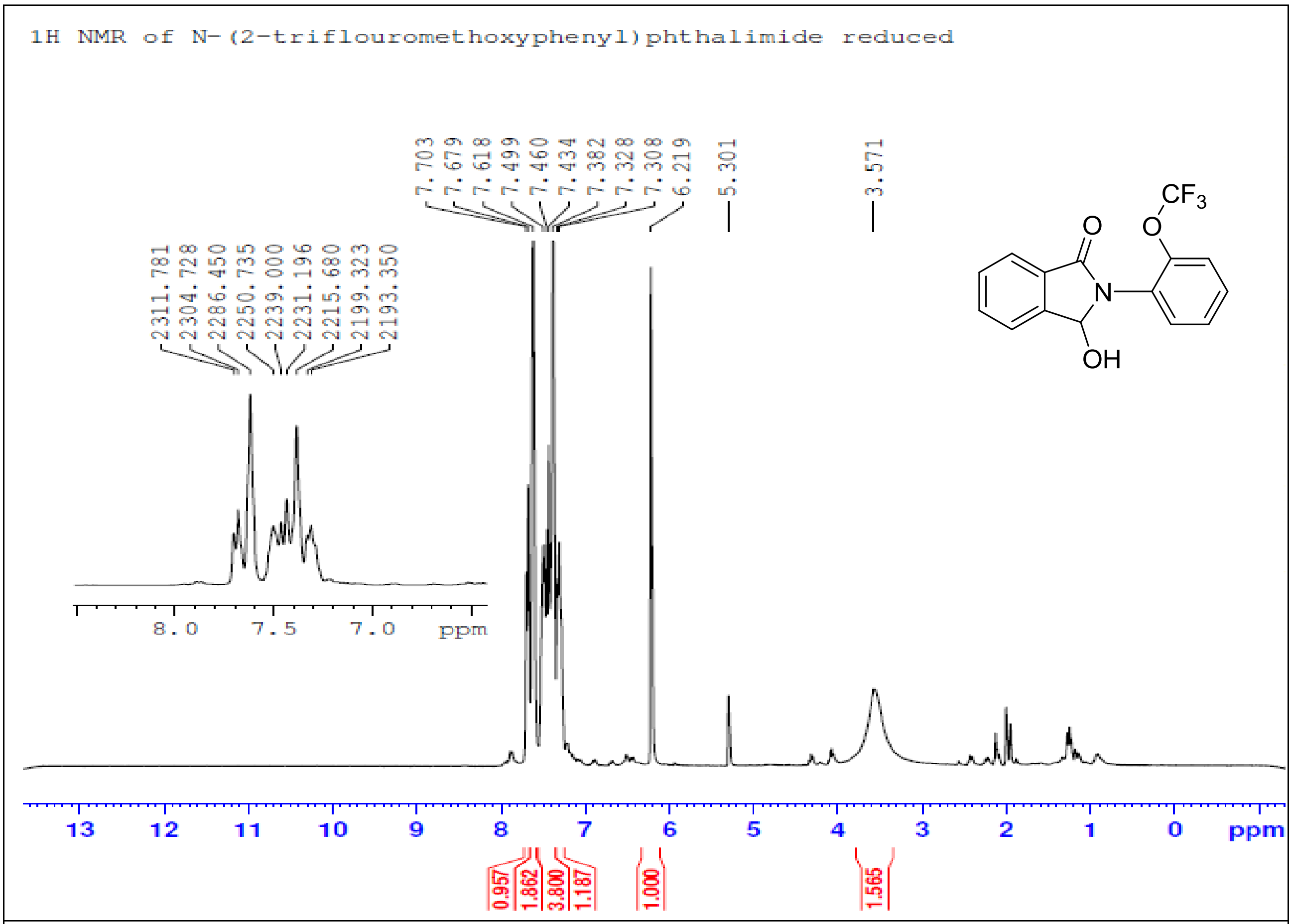
1H NMR of N-(2-triflouromethoxyphenyl)phthalimide reduced
7.703
7.679
7.618
7.499
7.460
7.434
7.382
7.328
7.308
6.219
5.301
3.571
2311.781
2304.728
2286.450
2250.735
2239.000
2231.196
2215.680
2199.323
2193.350
8.0
7.5
7.0
ppm
13 12 11 10 9 8 7 6 5 4 3 2 1 0 ppm
0.957
1.862
3.800
1.187
1.000
1.565
CF3
O
N
OH

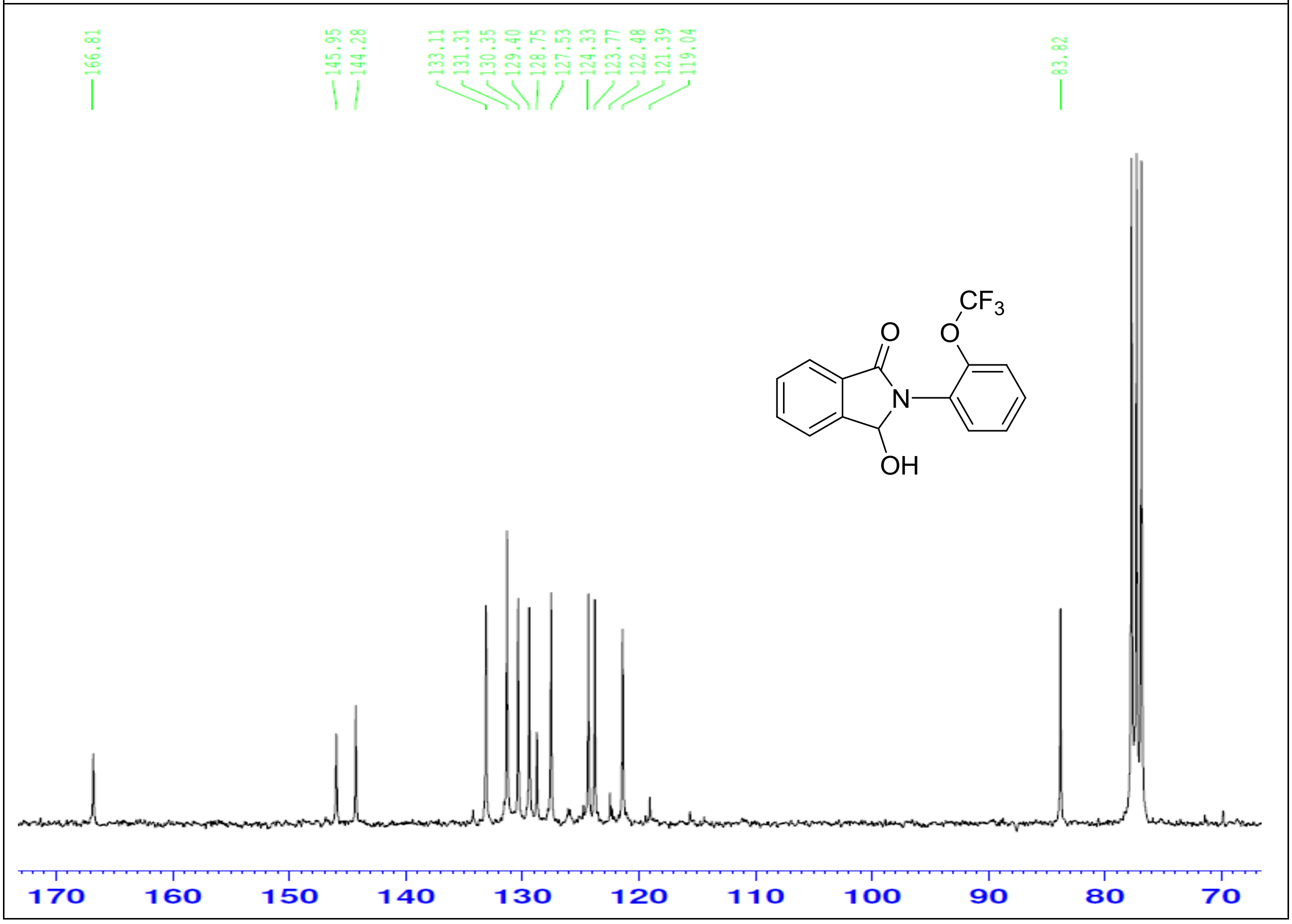
166.81
145.95
144.28
133.11
131.31
130.35
129.40
128.75
127.53
124.33
123.77
122.48
121.39
119.04
83.82
CF3
O
N
OH
170 160 150 140 130 120 110 100 90 80 70

X-Ray Crystal Structure of Compound 1b (CCDC 952236)

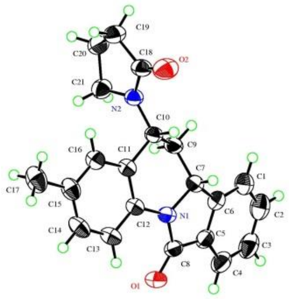

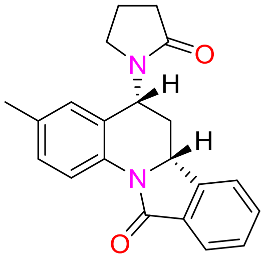

1b

Figure 1: ORTEP diagram showing crystallographic atom numbering and solid-state conformation for 3-methyl-5-(2-oxopyrrolidin-1-yl)-6,6*a*-dihydroisoindolo[2,1-*a*]quinolin-11(5*H*)-one (**1b**). The additional set of H atoms attached to the disordered CH_3 group is omitted for clarity.

A colourless needle crystal of **1b** having approximate dimensions of 0.37 × 0.19 × 0.18 mm was mounted on a glass fiber. All measurements were made on a Rigaku RAXIS RAPID imaging plate area detector with graphite monochromated Mo-Kα radiation. The structure was solved by direct methods [8] and expanded using Fourier techniques [9]. The non-hydrogen atoms were refined anisotropically. Hydrogen atoms were refined using the riding model.

The crystallographic data for **1b** are listed in Table 1, and Table 2 lists the selected bond distances and angles. CCDC 952236 for **1b** contains the supplementary crystallographic data for this paper. These data can be obtained free of charge via www.ccdc.cam.ac.uk/data_request/cif (or from the Cambridge Crystallographic Data Centre, 12, Union Road, Cambridge CB2 1EZ, UK; fax: (+44) 1223-336-033; or deposit@ccdc.cam.ac.uk).

Table 1: Crystal data and structure refinements for **1b**.

**Crystal Data**	
Empirical Formula	$C_{21}H_{20}N_2O_2$
Formula Weight	332.40
Crystal Color, Habit	colourless, needle
Crystal Dimensions	0.37 X 0.19 X 0.18 mm
Crystal System	monoclinic
Lattice Type	Primitive
Indexing Images	4 oscillations @ 180.0 seconds
Detector Position	127.40 mm
Pixel Size	0.100 mm

Lattice Parameters	a = 9.1629(2) Å
	b = 6.9694(2) Å
	c = 26.0355(8) Å
	β = 98.2109(14)°
	V = 1645.58(8) $Å^3$
Space Group	$P2_1/c$ (#14)
Z value	4
D_{calc}	1.342 g/cm^3
F_{000}	704.00
μ(MoKα)	0.870 cm^{-1}
**Intensity Measurements**	
Diffractometer	Rigaku RAXIS-UNKNOWN
Radiation	MoKα (λ = 0.71070 Å)
	graphite monochromated
Data Images	35 exposures
ω oscillation Range	20.0 - 195.0°
Exposure Rate	288.0 sec./°
Detector Position	127.40 mm
Pixel Size	0.100 mm
$2\theta_{max}$	71.1°
No. of Reflections Measured	Total: 17656; Unique: 6258 (R_{int} = 0.023)
Corrections	Lorentz-polarization
	Absorption
	(trans. factors: 0.733 - 0.984)
**Structure Solution and Refinement**	
Structure Solution	Direct Methods (SHELX97)
Refinement	Full-matrix least-squares on F
Function Minimized	Σ w (|Fo| - |Fc|)^2
Least Squares Weights	Chebychev polynomial with 3 parameters
	5.5530, 4.0059, 4.1133

$2\theta_{max}$ cutoff	52.0°
Anomalous Dispersion	All non-hydrogen atoms
No. Observations (I>3.00σ(I))	2678
No. Variables	249
Reflection/Parameter Ratio	10.76
Residuals: R (I>3.00σ(I))	0.0373
Residuals: Rw (I>3.00σ(I))	0.0476
Goodness of Fit Indicator	1.071
Max Shift/Error in Final Cycle	0.000
Maximum peak in Final Diff. Map	0.14 $e^-/Å^3$
Minimum peak in Final Diff. Map	-0.19 $e^-/Å^3$

Table 2: Selected bond lengths (Å) and angles (°) for **1b**.

**Bond Lengths (Å)**			
Atom-Atom	Distance	Atom-Atom	Distance
O(1)-C(8)	1.2274(15)	O(2)-C(18)	1.2183(15)
N(1)-C(7)	1.4734(14)	N(1)-C(8)	1.3851(14)
N(1)-C(12)	1.4165(13)	N(2)-C(10)	1.4547(13)
N(2)-C(18)	1.3518(14)	N(2)-C(21)	1.4499(14)
C(1)-C(2)	1.393(2)	C(1)-C(6)	1.3840(17)
C(2)-C(3)	1.391(2)	C(3)-C(4)	1.382(2)
C(4)-C(5)	1.3879(16)	C(5)-C(6)	1.3818(18)
C(5)-C(8)	1.4757(16)	C(6)-C(7)	1.4970(15)
C(7)-C(9)	1.5185(15)	C(9)-C(10)	1.5273(14)
C(10)-C(11)	1.5209(16)	C(11)-C(12)	1.4096(15)
C(11)-C(16)	1.3926(14)	C(12)-C(13)	1.3961(16)
C(13)-C(14)	1.3801(17)	C(14)-C(15)	1.388(2)
C(15)-C(16)	1.3856(16)	C(15)-C(17)	1.5084(19)
C(18)-C(19)	1.5124(17)	C(19)-C(20)	1.5150(18)
C(20)-C(21)	1.5235(19)		

Bond Angles (°)			
Atom-Atom-Atom	Angle	Atom-Atom-Atom	Angle
C(7)-N(1)-C(8)	112.05(8)	C(7)-N(1)-C(12)	119.75(9)
C(8)-N(1)-C(12)	127.63(9)	C(10)-N(2)-C(18)	122.01(9)
C(10)-N(2)-C(21)	124.07(9)	C(18)-N(2)-C(21)	113.68(9)
C(2)-C(1)-C(6)	117.99(14)	C(1)-C(2)-C(3)	121.27(14)
C(2)-C(3)-C(4)	120.49(12)	C(3)-C(4)-C(5)	117.93(13)
C(4)-C(5)-C(6)	121.85(12)	C(4)-C(5)-C(8)	128.68(12)
C(6)-C(5)-C(8)	109.43(9)	C(1)-C(6)-C(5)	120.44(11)
C(1)-C(6)-C(7)	130.21(12)	C(5)-C(6)-C(7)	109.35(9)
N(1)-C(7)-C(6)	102.63(9)	N(1)-C(7)-C(9)	110.17(8)
C(6)-C(7)-C(9)	117.42(9)	O(1)-C(8)-N(1)	126.53(9)
O(1)-C(8)-C(5)	127.39(9)	N(1)-C(8)-C(5)	106.06(9)
C(7)-C(9)-C(10)	107.29(8)	N(2)-C(10)-C(9)	111.74(8)
N(2)-C(10)-C(11)	113.12(8)	C(9)-C(10)-C(11)	110.38(8)
C(10)-C(11)-C(12)	120.61(8)	C(10)-C(11)-C(16)	120.36(9)
C(12)-C(11)-C(16)	119.02(10)	N(1)-C(12)-C(11)	119.58(9)
N(1)-C(12)-C(13)	121.52(10)	C(11)-C(12)-C(13)	118.90(9)
C(12)-C(13)-C(14)	120.08(12)	C(13)-C(14)-C(15)	122.27(11)
C(14)-C(15)-C(16)	117.23(10)	C(14)-C(15)-C(17)	121.74(11)
C(16)-C(15)-C(17)	121.03(12)	C(11)-C(16)-C(15)	122.49(11)
O(2)-C(18)-N(2)	125.53(10)	O(2)-C(18)-C(19)	127.01(11)
N(2)-C(18)-C(19)	107.49(10)	C(18)-C(19)-C(20)	104.75(10)
C(19)-C(20)-C(21)	104.12(10)	N(2)-C(21)-C(20)	102.94(10)

X-ray Crystal Structure of Compound 1h (CCDC 951754)

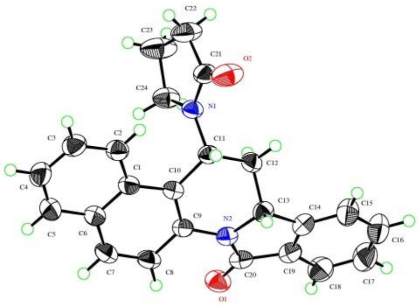

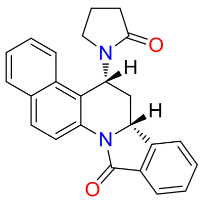

1h

Figure 2 ORTEP diagram showing crystallographic atom numbering and solid-state conformation for 7-(2-oxopyrrolidin-1-yl)-8,8*a*-dihydrobenzo[*f*]isoindolo[2,1-*a*]quinolin-13(7*H*)-one (**1h**).

A colourless needle crystal of **1h** having approximate dimensions of 0.44 × 0.37 × 0.32 mm was mounted on a glass fiber. All measurements were made on a Rigaku RAXIS RAPID imaging plate area detector with graphite monochromated Mo Kα radiation. The structure was solved by direct methods [8] and expanded using Fourier techniques [9]. The non-hydrogen atoms were refined anisotropically. Hydrogen atoms were refined using the riding model.

The crystallographic data for **1h** are listed in Table 3, and Table 4 lists the selected bond distances and angles. CCDC 951754 for **1h** contains the supplementary crystallographic data for this paper. These data can be obtained free of charge via www.ccdc.cam.ac.uk/data_request/cif (or from the Cambridge Crystallographic Data Centre, 12, Union Road, Cambridge CB2 1EZ, UK; fax: (+44) 1223-336-033; or deposit@ccdc.cam.ac.uk).

Table 3: Crystal data and structure refinements for **1h**.

**Crystal Data**	
Empirical Formula	$C_{24}H_{20}N_2O_2$
Formula Weight	368.43
Crystal Color, Habit	colourless, hexagonal needle
Crystal Dimensions	0.44 X 0.37 X 0.32 mm
Crystal System	Monoclinic
Lattice Type	Primitive
Indexing Images	4 oscillations @ 180.0 seconds
Detector Position	127.40 mm
Pixel Size	0.100 mm
Lattice Parameters	a = 10.0772(2) Å

	b = 17.5969(3) Å
	c = 10.4208(3) Å
	β = 96.3441(13)°
	V = 1836.58(7) $Å^3$
Space Group	$P2_1/a$ (#14)
Z value	4
D_{calc}	1.332 g/cm^3
F_{000}	776.00
μ(MoKα)	0.854 cm^{-1}
**Intensity Measurements**	
Diffractometer	Rigaku RAXIS-UNKNOWN
Radiation	MoKα (λ = 0.71070 Å)
	graphite monochromated
Data Images	45 exposures
ω oscillation Range	20.0 - 200.0°
Exposure Rate	300.0 sec./°
Detector Position	127.40 mm
Pixel Size	0.100 mm
$2\theta_{max}$	71.3°
No. of Reflections Measured	Total: 25005; Unique: 6970 (R_{int} = 0.021)
Corrections	Lorentz-polarization
	Absorption; (trans. factors: 0.660 - 0.973)
**Structure Solution and Refinement**	
Structure Solution	Direct Methods (SHELX97)
Refinement	Full-matrix least-squares on F
Function Minimized	Σ w (|Fo| - |Fc|)^2
Least Squares Weights	Chebychev polynomial with 3 parameters
	6.2397, 3.8659, 4.1125
$2\theta_{max}$ cutoff	54.0°
Anomalous Dispersion	All non-hydrogen atoms

No. Observations (I>3.00σ(I))	3370
No. Variables	273
Reflection/Parameter Ratio	12.34
Residuals: R (I>3.00σ(I))	0.0473
Residuals: Rw (I>3.00σ(I))	0.0569
Goodness of Fit Indicator	1.079
Max Shift/Error in Final Cycle	0.000
Maximum peak in Final Diff. Map	0.29 $e^-/Å^3$
Minimum peak in Final Diff. Map	-0.45 $e^-/Å^3$

Table 4: Selected bond lengths (Å) and angles (°) for **1h**.

**Bond Lengths (Å)**			
Atom-Atom	Distance	Atom-Atom	Distance
O(1)-C(20)	1.2203(17)	O(2)-C(21)	1.218(2)
N(1)-C(11)	1.4670(14)	N(1)-C(21)	1.3442(17)
N(1)-C(24)	1.4601(17)	N(2)-C(9)	1.4073(16)
N(2)-C(13)	1.4678(16)	N(2)-C(20)	1.3828(16)
C(1)-C(2)	1.4222(19)	C(1)-C(6)	1.4221(18)
C(1)-C(10)	1.4322(17)	C(2)-C(3)	1.369(2)
C(3)-C(4)	1.400(2)	C(4)-C(5)	1.366(2)
C(5)-C(6)	1.419(2)	C(6)-C(7)	1.4165(19)
C(7)-C(8)	1.356(2)	C(8)-C(9)	1.4227(18)
C(9)-C(10)	1.3886(16)	C(10)-C(11)	1.5258(16)
C(11)-C(12)	1.5290(17)	C(12)-C(13)	1.5186(17)
C(13)-C(14)	1.4983(18)	C(14)-C(15)	1.384(2)
C(14)-C(19)	1.3813(19)	C(15)-C(16)	1.389(2)
C(16)-C(17)	1.385(2)	C(17)-C(18)	1.382(2)
C(18)-C(19)	1.390(2)	C(19)-C(20)	1.4832(19)
C(21)-C(22)	1.512(2)	C(22)-C(23)	1.466(3)
C(23)-C(24)	1.504(2)		

Bond Angles (°)			
Atom-Atom-Atom	Angle	Atom-Atom-Atom	Angle
C(11)-N(1)-C(21)	121.76(10)	C(11)-N(1)-C(24)	123.89(10)
C(21)-N(1)-C(24)	113.93(10)	C(9)-N(2)-C(13)	118.45(10)
C(9)-N(2)-C(20)	127.65(11)	C(13)-N(2)-C(20)	112.97(10)
C(2)-C(1)-C(6)	117.46(11)	C(2)-C(1)-C(10)	122.62(11)
C(6)-C(1)-C(10)	119.92(11)	C(1)-C(2)-C(3)	121.13(12)
C(2)-C(3)-C(4)	121.00(13)	O(3)-C(4)-C(5)	119.73(14)
C(4)-C(5)-C(6)	120.81(14)	C(1)-C(6)-C(5)	119.75(12)
C(1)-C(6)-C(7)	118.77(12)	C(5)-C(6)-C(7)	121.48(12)
C(6)-C(7)-C(8)	121.18(12)	C(7)-C(8)-C(9)	120.40(12)
N(2)-C(9)-C(8)	119.18(11)	N(2)-C(9)-C(10)	119.96(10)
C(8)-C(9)-C(10)	120.80(11)	C(1)-C(10)-C(9)	118.62(10)
C(1)-C(10)-C(11)	120.16(10)	C(9)-C(10)-C(11)	121.22(10)
N(1)-C(11)-C(10)	112.10(9)	N(1)-C(11)-C(12)	108.32(9)
C(10)-C(11)-C(12)	112.63(9)	C(11)-C(12)-C(13)	109.89(9)
N(2)-C(13)-C(12)	107.82(9)	N(2)-C(13)-C(14)	102.59(10)
C(12)-C(13)-C(14)	115.36(10)	C(13)-C(14)-C(15)	130.01(13)
C(13)-C(14)-C(19)	109.35(11)	C(15)-C(14)-C(19)	120.64(13)
C(14)-C(15)-C(16)	117.87(15)	C(15)-C(16)-C(17)	121.53(16)
C(16)-C(17)-C(18)	120.45(17)	C(17)-C(18)-C(19)	118.06(15)
C(14)-C(19)-C(18)	121.44(13)	C(14)-C(19)-C(20)	109.67(11)
C(18)-C(19)-C(20)	128.86(12)	O(1)-C(20)-N(2)	127.17(12)
O(1)-C(20)-C(19)	127.50(12)	N(2)-C(20)-C(19)	105.33(10)
O(2)-C(21)-N(1)	125.26(12)	O(2)-C(21)-C(22)	127.08(14)
N(1)-C(21)-C(22)	107.65(12)	C(21)-C(22)-C(23)	106.07(13)
C(22)-C(23)-C(24)	107.60(15)	N(1)-C(24)-C(23)	103.79(13)

X-ray Crystal Structure of Compound 2b (CCDC 951755)

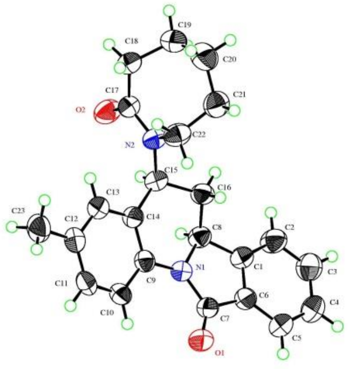

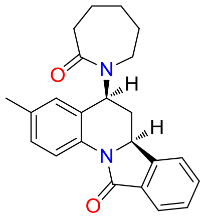

2b

Figure 3: ORTEP diagram showing crystallographic atom numbering and solid-state conformation for 3-methyl-5-(2-oxoazepan-1-yl)-6,6*a*-dihydroisoindolo[2,1-*a*]quinolin-11(5*H*)-one (**2b**).

A colourless needle crystal of **2b** having approximate dimensions of 0.41 × 0.14 × 0.11 mm was mounted on a glass fiber. All measurements were made on a Rigaku RAXIS RAPID imaging plate area detector with graphite monochromated Mo Kα radiation. The structure was solved by direct methods [8] and expanded using Fourier techniques [9]. The non-hydrogen atoms were refined anisotropically. Hydrogen atoms were refined using the riding model.

The crystallographic data for **2b** are listed in Table 5, and Table 6 lists the selected bond distances and angles. CCDC 951755 for **2b** contains the supplementary crystallographic data for this paper. These data can be obtained free of charge via www.ccdc.cam.ac.uk/data_request/cif (or from the Cambridge Crystallographic Data Centre, 12, Union Road, Cambridge CB2 1EZ, UK; fax: (+44) 1223-336-033; or deposit@ccdc.cam.ac.uk).

Table 5: Crystal data and structure refinements for **2b**.

**Crystal Data**	
Empirical Formula	$C_{23}H_{24}N_2O_2$
Formula Weight	360.45
Crystal Color, Habit	colourless, needle
Crystal Dimensions	0.41 X 0.14 X 0.11 mm
Crystal System	Monoclinic
Lattice Type	Primitive
Indexing Images	4 oscillations @ 120.0 seconds
Detector Position	127.40 mm
Pixel Size	0.100 mm

Lattice Parameters	a = 10.2484(3) Å
	b = 33.4029(12) Å
	c = 11.1621(2) Å
	β = 99.4030(14)°
	V = 3769.74(19) $Å^3$
Space Group	$P2_1/a$ (#14)
Z value	8
D_{calc}	1.270 g/cm^3
F_{000}	1536.00
μ(MoKα)	0.813 cm^{-1}
**Intensity Measurements**	
Diffractometer	Rigaku RAXIS-UNKNOWN
Radiation	MoKα (λ = 0.71070 Å)
	graphite monochromated
Data Images	36 exposures
ω oscillation Range	20.0 - 200.0°
Exposure Rate	264.0 sec./°
Detector Position	127.40 mm
Pixel Size	0.100 mm
$2\theta_{max}$	60.1°
No. of Reflections Measured	Total: 35171; Unique: 10853 (R_{int} = 0.043)
Corrections	Lorentz-polarization
	Absorption; (trans. factors: 0.591 - 0.991)
**Structure Solution and Refinement**	
Structure Solution	Direct Methods (SHELX97)
Refinement	Full-matrix least-squares on F
Function Minimized	Σ w $(\|Fo\| - \|Fc\|)^2$
Least Squares Weights	Chebychev polynomial with 3 parameters
	9.1859, 1.9202, 6.9202
$2\theta_{max}$ cutoff	52.0°

Anomalous Dispersion	All non-hydrogen atoms
No. Observations (I>3.00σ(I))	4480
No. Variables	487
Reflection/Parameter Ratio	9.20
Residuals: R (I>3.00σ(I))	0.0465
Residuals: Rw (I>3.00σ(I))	0.0582
Goodness of Fit Indicator	1.086
Max Shift/Error in Final Cycle	0.000
Maximum peak in Final Diff. Map	0.47 $e^-/Å^3$
Minimum peak in Final Diff. Map	-0.23 $e^-/Å^3$

Table 6: Selected bond lengths (Å) and angles (°) for **2b**.

**Bond Lengths (Å)**			
Atom-Atom	Distance	Atom-Atom	Distance
O(1)-C(7)	1.225(2)	O(2)-C(17)	1.234(3)
N(1)-C(7)	1.382(2)	N(1)-C(8)	1.474(2)
N(1)-C(9)	1.421(2)	N(2)-C(15)	1.461(2)
N(2)-C(17)	1.359(2)	N(2)-C(22)	1.467(3)
C(1)-C(2)	1.379(3)	C(1)-C(6)	1.376(3)
C(1)-C(8)	1.506(2)	C(2)-C(3)	1.381(3)
C(3)-C(4)	1.395(4)	C(4)-C(5)	1.375(3)
C(5)-C(6)	1.391(3)	C(6)-C(7)	1.476(2)
C(8)-C(16)	1.514(3)	C(9)-C(10)	1.397(3)
C(9)-C(14)	1.407(2)	C(10)-C(11)	1.384(3)
C(11)-C(12)	1.389(3)	C(12)-C(13)	1.390(3)
C(12)-C(23)	1.499(3)	C(13)-C(14)	1.390(2)
C(14)-C(15)	1.522(2)	C(15)-C(16)	1.529(3)
C(17)-C(18)	1.510(3)	C(18)-C(19)	1.523(3)
C(19)-C(20)	1.478(4)	C(20)-C(21)	1.512(4)
C(21)-C(22)	1.524(3)		

Bond Angles (°)			
Atom-Atom-Atom	Angle	Atom-Atom-Atom	Angle
C(7)-N(1)-C(8)	112.80(16)	C(7)-N(1)-C(9)	127.21(17)
C(8)-N(1)-C(9)	119.19(16)	C(15)-N(2)-C(17)	118.86(19)
C(15)-N(2)-C(22)	118.26(18)	C(17)-N(2)-C(22)	122.71(18)
C(2)-C(1)-C(6)	121.2(2)	C(2)-C(1)-C(8)	129.3(2)
C(6)-C(1)-C(8)	109.46(18)	C(1)-C(2)-C(3)	117.6(2)
C(2)-C(3)-C(4)	121.3(2)	C(3)-C(4)-C(5)	120.8(2)
C(4)-C(5)-C(6)	117.5(2)	C(1)-C(6)-C(5)	121.5(2)
C(1)-C(6)-C(7)	109.85(18)	C(5)-C(6)-C(7)	128.6(2)
O(1)-C(7)-N(1)	126.70(19)	O(1)-C(7)-C(6)	127.6(2)
N(1)-C(7)-C(6)	105.71(18)	N(1)-C(8)-C(1)	102.13(16)
N(1)-C(8)-C(16)	110.40(17)	C(1)-C(8)-C(16)	117.20(18)
N(1)-C(9)-C(10)	120.94(18)	N(1)-C(9)-C(14)	119.05(17)
C(10)-C(9)-C(14)	120.01(18)	C(9)-C(10)-C(11)	119.4(2)
C(10)-C(11)-C(12)	122.1(2)	C(11)-C(12)-C(13)	117.45(19)
C(11)-C(12)-C(23)	121.9(2)	C(13)-C(12)-C(23)	120.6(2)
C(12)-C(13)-C(14)	122.7(2)	C(9)-C(14)-C(13)	118.26(18)
C(9)-C(14)-C(15)	121.18(17)	C(13)-C(14)-C(15)	120.50(18)
N(2)-C(15)-C(14)	112.88(17)	N(2)-C(15)-C(16)	113.40(17)
C(14)-C(15)-C(16)	111.51(17)	C(8)-C(16)-C(15)	107.84(18)
O(2)-C(17)-N(2)	121.9(2)	O(2)-C(17)-C(18)	120.6(2)
N(2)-C(17)-C(18)	117.5(2)	C(17)-C(18)-C(19)	113.9(2)
C(18)-C(19)-C(20)	115.7(2)	C(19)-C(20)-C(21)	117.4(2)
C(20)-C(21)-C(22)	113.4(2)	N(2)-C(22)-C(21)	114.2(2)

X-ray Crystal Structure of Compound 3a (CCDC 951756)

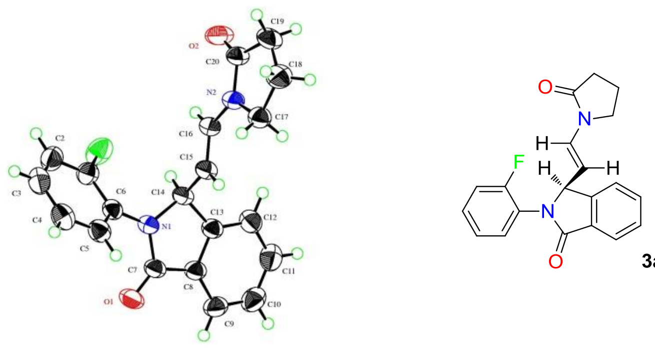

3a

Figure 4: ORTEP diagram showing crystallographic atom numbering and solid-state conformation for *E*-2-(2-fluorophenyl)-3-(2-(2-oxopyrrolidin-1-yl)vinyl)isoindolin-1-one (**3a**).

A colourless pinacoid crystal of **3a** having approximate dimensions of 0.25 × 0.24 × 0.16 mm was mounted on a glass fiber. All measurements were made on a Rigaku RAXIS RAPID imaging plate area detector with graphite monochromated Mo Kα radiation. The structure was solved by direct methods [8] and expanded using Fourier techniques [9]. The non-hydrogen atoms were refined anisotropically. Hydrogen atoms were refined using the riding model.

The crystallographic data for **3a** are listed in Table 7, and Table 8 lists the selected bond distances and angles. CCDC 951756 for **3a** contains the supplementary crystallographic data for this paper. These data can be obtained free of charge via www.ccdc.cam.ac.uk/data_request/cif (or from the Cambridge Crystallographic Data Centre, 12, Union Road, Cambridge CB2 1EZ, UK; fax: (+44) 1223-336-033; or deposit@ccdc.cam.ac.uk).

Table 7: Crystal data and structure refinements for **3a**.

**Crystal Data**	
Empirical Formula	$C_{20}H_{17}N_2O_2F$
Formula Weight	336.36
Crystal Color, Habit	colourless, pinacoid
Crystal Dimensions	0.25 X 0.24 X 0.16 mm
Crystal System	Monoclinic
Lattice Type	Primitive
Indexing Images	4 oscillations @ 300.0 seconds
Detector Position	127.40 mm
Pixel Size	0.100 mm

Lattice Parameters	a = 11.9085(3) Å
	b = 9.8202(2) Å
	c = 14.8555(5) Å
	β = 102.3677(16)°
	V = 1696.94(8) $Å^3$
Space Group	$P2_1/c$ (#14)
Z value	4
D_{calc}	1.316 g/cm^3
F_{000}	704.00
μ(MoKα)	0.933 cm^{-1}
**Intensity Measurements**	
Diffractometer	Rigaku RAXIS-UNKNOWN
Radiation	MoKα (λ = 0.71070 Å)
	graphite monochromated
Data Images	48 exposures
ω oscillation Range	20.0 - 200.0°
Exposure Rate	360.0 sec./°
Detector Position	127.40 mm
Pixel Size	0.100 mm
$2\theta_{max}$	60.1°
No. of Reflections Measured	Total: 20315; Unique: 4902 (R_{int} = 0.021)
Corrections	Lorentz-polarization
	Absorption; (trans. factors: 0.746 - 0.985)
**Structure Solution and Refinement**	
Structure Solution	Direct Methods (SHELX97)
Refinement	Full-matrix least-squares on F
Function Minimized	Σ w (|Fo| - |Fc|)^2
Least Squares Weights	Chebychev polynomial with 3 parameters
	7.2229, 5.0833, 5.6398
$2\theta_{max}$ cutoff	60.1°

Anomalous Dispersion	All non-hydrogen atoms
No. Observations (I>3.00σ(I))	3703
No. Variables	243
Reflection/Parameter Ratio	15.24
Residuals: R (I>3.00σ(I))	0.0389
Residuals: Rw (I>3.00σ(I))	0.0544
Goodness of Fit Indicator	1.041
Max Shift/Error in Final Cycle	0.000
Maximum peak in Final Diff. Map	0.28 $e^-/Å^3$
Minimum peak in Final Diff. Map	-0.14 $e^-/Å^3$

Table 8: Selected bond lengths (Å) and angles (°) for **3a**.

**Bond Lengths (Å)**			
Atom-Atom	Distance	Atom-Atom	Distance
F(1)-C(1)	1.3623(13)	O(1)-C(7)	1.2205(14)
O(2)-C(20)	1.2159(14)	N(1)-C(6)	1.4153(11)
N(1)-C(7)	1.3829(12)	N(1)-C(14)	1.4861(13)
N(2)-C(16)	1.3916(14)	N(2)-C(17)	1.4611(13)
N(2)-C(20)	1.3701(13)	C(1)-C(2)	1.3779(15)
C(1)-C(6)	1.3885(13)	C(2)-C(3)	1.3828(19)
C(3)-C(4)	1.3795(17)	C(4)-C(5)	1.3903(15)
C(5)-C(6)	1.3960(14)	C(7)-C(8)	1.4741(13)
C(8)-C(9)	1.3929(13)	C(8)-C(13)	1.3857(14)
C(9)-C(10)	1.3808(18)	C(10)-C(11)	1.3927(19)
C(11)-C(12)	1.3892(16)	C(12)-C(13)	1.3844(14)
C(13)-C(14)	1.5087(11)	C(14)-C(15)	1.5003(14)
C(15)-C(16)	1.3291(14)	C(17)-C(18)	1.5313(19)
C(18)-C(19)	1.5135(19)	C(19)-C(20)	1.5040(17)

Bond Angles (°)			
Atom-Atom-Atom	Angle	Atom-Atom-Atom	Angle
C(6)-N(1)-C(7)	123.03(8)	C(6)-N(1)-C(14)	124.17(7)
C(7)-N(1)-C(14)	112.79(7)	C(16)-N(2)-C(17)	123.59(8)
C(16)-N(2)-C(20)	121.66(8)	C(17)-N(2)-C(20)	114.30(9)
F(1)-C(1)-C(2)	117.96(9)	F(1)-C(1)-C(6)	118.62(8)
C(2)-C(1)-C(6)	123.42(10)	C(1)-C(2)-C(3)	118.89(10)
C(2)-C(3)-C(4)	119.68(11)	C(3)-C(4)-C(5)	120.52(11)
C(4)-C(5)-C(6)	121.06(9)	N(1)-C(6)-C(1)	122.03(8)
N(1)-C(6)-C(5)	121.53(8)	C(1)-C(6)-C(5)	116.42(8)
O(1)-C(7)-N(1)	125.61(9)	O(1)-C(7)-C(8)	128.15(8)
N(1)-C(7)-C(8)	106.24(8)	C(7)-C(8)-C(9)	129.16(10)
C(7)-C(8)-C(13)	109.38(8)	C(9)-C(8)-C(13)	121.46(9)
C(8)-C(9)-C(10)	117.58(11)	C(9)-C(10)-C(11)	121.15(11)
C(10)-C(11)-C(12)	121.01(11)	C(11)-C(12)-C(13)	117.94(11)
C(8)-C(13)-C(12)	120.85(8)	C(8)-C(13)-C(14)	109.95(8)
C(12)-C(13)-C(14)	129.19(9)	N(1)-C(14)-C(13)	101.61(7)
N(1)-C(14)-C(15)	112.54(8)	C(13)-C(14)-C(15)	113.19(7)
C(14)-C(15)-C(16)	120.74(8)	N(2)-C(16)-C(15)	124.67(9)
N(2)-C(17)-C(18)	103.99(9)	C(17)-C(18)-C(19)	106.38(11)
C(18)-C(19)-C(20)	106.13(10)	O(2)-C(20)-N(2)	124.52(10)
O(2)-C(20)-C(19)	127.47(10)	N(2)-C(20)-C(19)	107.99(9)

References

1. Hsieh, J. –C.; Cheng, C. -H. Nickel-catalyzed coupling of isocyanates with 1,3-iodoesters and halobenzenes: a novel method for the synthesis of imide and amide derivatives. *Chem. Commun.* **2005**, 4554-4556. doi: 10.1039/b506903c

2. Shibata, Y.; Sasaki, K.; Hashimoto, Y.; Iwasaki, S. Phenylphthalimides with tumor necrosis factor alpha production-enhancing activity. *Chem. Pharm. Bull.* **1996**, *44*, 156-162. doi: 10.1248/cpb.44.156

3. Kim, H. J.; Kim, J.; Cho, S. H.; Chang, S. Intermolecular oxidative C–N bond formation under metal-free conditions: Control of chemoselectivity between aryl sp^2 and benzylic sp^3 C–H bond imidation. *J. Am. Chem. Soc.* **2011**, *133*, 16382-16385. doi: 10.1021/ja207296y

4. Sena, V. L. M.; Srivastava, R. M.; Silva, R. O.; Lima, V. L. M. Synthesis and hypolipidemic activity of N-substituted phthalimides. Part V. *Farmaco* **2003**, *58*, 1283-1288. doi: 10.1016/S0014-827X(03)00185-X

5. Mali, R. S.; Yeola, S. N. A Novel Synthesis of N-Substituted-3-carboethoxymethylphthalimidines. *Synthesis* **1986**, 755-757. doi: 10.1055/s-1986-31765

6. Yuan, X. –H.; Zhang, M. –J.; Kang, C. –Q.; Gou, H. –Q.; Qui, X. –P.; Gao, L. –X. Efficient synthesis of aryl hydroxylactams by reducing imides with activated zinc dust. *Synth. Commun.* **2006**, *36*, 435-444. doi: 10.1080/00397910500383485

7. Li, J.; Chen, J.; Chen, H. –B.; Chen, W. –X. Low-valent titanium induced reduction of phthalimides and coupling of corresponding isoindoles. *Synth. Commun.* **1998**, *28*, 1281-1286. doi: 10.1080/00397919808005970

8. SHELX97: Sheldrick, G. M. (**1997**).

9. DIRDIF99: Beurskens, P.T.; Admiraal, G.; Beurskens, G.; Bosman, W.P.; de Gelder, R.; Israel, R.; Smits, J. M. M. (**1999**). The DIRDIF-99 program system, Technical Report of the Crystallography Laboratory, University of Nijmegen, The Netherlands.
